# Novel Tyrosine Kinase-Mediated Phosphorylation With Dual Specificity Plays a Key Role in the Modulation of *Streptococcus pyogenes* Physiology and Virulence

**DOI:** 10.3389/fmicb.2021.689246

**Published:** 2021-12-07

**Authors:** Sashi Kant, Vijay Pancholi

**Affiliations:** Department of Pathology, The Ohio State University College of Medicine, Columbus, OH, United States

**Keywords:** tyrosine kinase, Group A *Streptococcus*, *Streptococcus pyogenes*, post-translational modifications, tyrosine phosphorylation, dual-specificity tyrosine kinase

## Abstract

*Streptococcus pyogenes* (Group A *Streptococcus*, GAS) genomes do not contain a gene encoding a typical bacterial-type tyrosine kinase (BY-kinase) but contain an orphan gene-encoding protein Tyr-phosphatase (SP-PTP). Hence, the importance of Tyr-phosphorylation is underappreciated and not recognized for its role in GAS pathophysiology and pathogenesis. The fact that SP-PTP dephosphorylates Abl-tyrosine kinase-phosphorylated myelin basic protein (MBP), and SP-STK (*S. pyogenes* Ser/Thr kinase) also autophosphorylates its Tyr^101^-residue prompted us to identify a putative tyrosine kinase and Tyr-phosphorylation in GAS. Upon a genome-wide search of kinases possessing a classical Walker motif, we identified a non-canonical tyrosine kinase M5005_Spy_1476, a ∼17 kDa protein (153 aa) (SP-TyK). The purified recombinant SP-TyK autophosphorylated in the presence of ATP. *In vitro* and *in vivo* phosphoproteomic analyses revealed two key phosphorylated tyrosine residues located within the catalytic domain of SP-TyK. An isogenic mutant lacking SP-TyK derived from the M1T1 strain showed a retarded growth pattern. It displayed defective cell division and long chains with multiple parallel septa, often resulting in aggregates. Transcriptomic analysis of the mutant revealed 287 differentially expressed genes responsible for GAS pathophysiology and pathogenesis. SP-TyK also phosphorylated GAS CovR, WalR, SP-STP, and SDH/GAPDH proteins with dual specificity targeting their Tyr/Ser/Thr residues as revealed by biochemical and mass-spectrometric-based phosphoproteomic analyses. SP-TyK-phosphorylated CovR bound to P*covR* efficiently. The mutant displayed sustained release of IL-6 compared to TNF-α during co-culturing with A549 lung cell lines, attenuation in mice sepsis model, and significantly reduced ability to adhere to and invade A549 lung cells and form biofilms on abiotic surfaces. SP-TyK, thus, plays a critical role in fine-tuning the regulation of key cellular functions essential for GAS pathophysiology and pathogenesis through post-translational modifications and hence, may serve as a promising target for future therapeutic developments.

## Introduction

The post-translational modification (PTM) of proteins occurring at certain Ser, Thr, and Tyr residues subsequent to creating the polypeptide chain is an important mechanism used by all living organisms to modulate the activity of key factors that play critical roles in a variety of cellular mechanisms. Group A *Streptococcus* (GAS, *S. pyogenes*), a successful human pathogen, is associated with high morbidity and mortality (Stevens, 1995; Bisno and Stevens, 1996; Steer et al., 2002; Snider and Swedo, 2003; McDonald et al., 2004; Rodriguez-Iturbe and Musser, 2008). GAS pathogenesis is, in part, controlled by PTMs at Asp, His, Ser, and Thr residues by kinases and phosphatases of two-component as well as other phospho-relay regulatory systems activated by a variety of environmental cues (Kreikemeyer et al., 2003; McIver, 2009). In *S. pyogenes*, eukaryote-type Ser/Thr kinase (SP-STK), phosphatase (SP-STP), as well as a dual-specificity low molecular weight (LMW) protein-Tyr-phosphatase (PTP), play decisive roles in the regulation of GAS growth and metabolism, cell division, cell wall biosynthesis, virulence, and penicillin tolerance (Jin and Pancholi, 2006; Pancholi et al., 2010; Agarwal et al., 2011, 2012; Bugrysheva et al., 2011; Horstmann et al., 2014; Kant et al., 2015). GAS genomes, however, do not contain gene(s) encoding the known prokaryote-type BY-kinases (Bacterial type Tyr-kinase) (Cozzone et al., 2004; Grangeasse et al., 2007, 2012; Jadeau et al., 2012; Chao et al., 2014). Hence, it is presumed that Tyr-phosphorylation does not exist in GAS.

Tyrosine kinase-phosphorylated proteins contribute to only 1–2% of the total phosphorylated proteins in eukaryotes, yet, it plays a crucial regulatory role in a variety of cellular functions (Hunter, 2000, 2002), including the pathophysiology of sepsis, which involves dysregulation of pro-inflammatory and anti-inflammatory responses (Oberholzer et al., 2001; Hotchkiss and Karl, 2003; Abraham and Singer, 2007). However, several proteomic analyses of prokaryotes have revealed that Tyr-phosphorylation is very common in prokaryotes (Lower et al., 2004; Sun et al., 2010; Esser et al., 2012; Kennelly, 2014; Ravikumar et al., 2014). Approximately 9–10% of the total phosphorylated proteins in *S. pneumoniae* (Sun et al., 2010) and *Bacillus subtilis* (Ravikumar et al., 2014), and up to 54% in *Archaean* species, *Sulfolobus solfataricus* (Esser et al., 2012) are Tyr-phosphorylated indicating that prokaryotes, in general, rely on Tyr-phosphorylation more heavily than eukaryotes for their metabolic functions and their regulation. BY-kinases, although associated with their direct role in capsule biosynthesis in Gram-positive and Gram-negative bacteria (Cozzone et al., 2004; Grangeasse et al., 2007, 2012; Jadeau et al., 2012; Chao et al., 2014), do not regulate global prokaryotic Tyr-phosphorylation indicating that other non-canonical Tyr-kinases exist in prokaryotes (Sun et al., 2010; Esser et al., 2012; Ravikumar et al., 2014). Our recent report showing phosphorylated Tyr101 of the autophosphorylated SP-STK (Jin and Pancholi, 2006; Kant et al., 2015) prompted us to investigate the possibility of the existence of tyrosine-phosphorylation in *S. pyogenes*. Additionally, we previously demonstrated that the inhibition of Tyr-phosphatase activity of SP-PTP (i.e., indirectly enriching Tyr- kinase activity or Tyr- phosphorylation) results in a GAS phenotype exhibiting substantially reduced virulence property (Kant et al., 2015). Interestingly, our RNA-seq analysis of the M1T1ΔPTP mutant strain in comparison with the Wild-type M1T1 strain [see supplement (Kant et al., 2015)] has revealed ∼eightfold downregulation of Spy_*1476/sp-TyK*. It is, therefore, likely that SP-PTP and the newly identified SP-TyK are physiologically linked.

Bioinformatic analysis has revealed that Spy_1476 belongs to a specialized class of bacterial kinase annotated as UPF0079 (Nguyen et al., 2017). There are, so far, 13 known members of this class, including YdiB of *Bacillus subtilis* (Nguyen et al., 2017) and UbK of *Streptococcus pneumoniae* (Pelletier et al., 2019), and other Gram-positive and Gram-negative bacteria. The physiological role of these proteins other than ATP/GTP hydrolase and cell division (Nguyen et al., 2017; Pelletier et al., 2019) is presently unknown. Furthermore, the role and the potential targets of this kinase in other bacteria and their pathogenesis are still unknown. We, therefore, surmise that the elucidation of functions of SP-TyK will have a substantial impact in understanding the role of this class of proteins in the pathogenesis and metabolism of Gram-positive pathogens (GPPs) in general and *S. pyogenes* in particular.

In the present manuscript, therefore, we have characterized the Spy_1476/SP-TyK protein and corresponding GAS mutant to understand the contribution of this protein to GAS pathogenesis. We have also identified its potential targets predicted on the basis of global transcriptome profile of the GAS mutant lacking SP-TyK vs. the parent wild-type GAS strain. Our key findings indicated that SP-TyK-mediated post-translational modifications play an essential role in GAS pathophysiology and pathogenesis.

## Materials and Methods

### Bacterial Strains, Reagents, and Cell Lines

The wild-type *S. pyogenes* strain M1T1 5448 (Agarwal et al., 2011) and its corresponding mutant and complemented strains were grown at 37°C in Todd-Hewitt broth (Sigma-Aldrich Co., St Louis, MO, United States) supplemented with 0.5% (W/V) yeast extract (Difco, BD, NJ, United States) with or without spectinomycin (500 μg/ml). The *E. coli* strains DH5α, BL21 (λDE3) pLysS, and MC1061 were grown in Luria–Bertani (L.B.) broth or agar at 37°C. MC1061 strains and chloramphenicol (5 μg/ml) were used to select the complemented strain. All enzymes for DNA manipulations were obtained from New England Bio Lab (Ipswich, MA, United States). All other chemicals, unless otherwise mentioned, were obtained from Sigma-Aldrich or ThermoFisher Co. Human lung epithelial A549 cell lines (CCL-185) were obtained from American Type Culture Collection (ATCC, Manassas, VA, United States) and were maintained in DMEM with 10% FBS at 37°C in a humidified CO_2_ incubator as described previously (Jin and Pancholi, 2006; Agarwal et al., 2011; Kant et al., 2015).

### Recombinant Proteins, Anti-*Streptococcus pyogenes*-Tyrosine Kinase Antibodies, and Purification

Recombinant 6XHis-tag SP-TyK was produced by cloning the *spy*_*1476* (*sp*-*TyK*) gene in the pET14B vector using primer pairs HisSP-TyK-F/R (Primers # 1/2; Supplementary Table 1), and by expressing it in pLysS BL21 strain under 1 mM IPTG induction at 30°C for 12 h, as described previously (Kant et al., 2015). rHis-SP-TyK was purified by Ni^+2^-NTA-based affinity column chromatography followed by Superdex-200 FPLC size column chromatography as previously described (Kant et al., 2015). We employed similar strategies to produce recombinant, SP-PTP (Spy_0036, Primers # 11/12) (Kant et al., 2015), CovR (Spy_0282, Primers # 13/14), VicR/WalR (Spy_0435, Primers # 15/16) (Agarwal et al., 2011), SP-STP (Spy_1336, Primers # 17/18) (Jin and Pancholi, 2006; Agarwal et al., 2012), SDH (Spy_0233, Primers # 19/20) (Jin et al., 2005), and SEN (Spy_0556, Primers # 21/22) (Pancholi et al., 2003; Derbise et al., 2004) as described. Bovine Myelin Basic-Protein (MBP, Cat # M1891) was obtained from Sigma-Aldrich. High titer rabbit antibody against SP-TyK was custom-raised using the 50-day expressed protocol (Lampire Biologicals Labs, Everett, PA, United States).

### Immunoblot Analysis

Test samples from GAS culture supernatants and cell lysates were resolved by gradient 12% precast SDS-PAGE gels (GenScript), Western blotted onto PVDF membranes, and analyzed by Coomassie stain followed by antibody staining. The purified SP-TyK and its tyrosine phosphorylation detection were carried out by staining with primary antibodies [anti-SP-TyK or Anti-TyrP (Cell Signaling)], and corresponding alkaline phosphatase-conjugated antibody and visualized by the chromogenic method (Agarwal et al., 2011, 2012).

### Mutation Strategies, Complementation, and Growth Curve

*Streptococcus pyogenes* mutant lacking the *spy*_*1476* gene was derived from Type M1 M1T1 5448 strain using the suicide pFW6 (Spectinomycin^*R*^) vector (Podbielski et al., 1996; Agarwal et al., 2011) and by employing the allelic replacement method as detailed in Supplementary Methods 1. Supplementary Table 1 describes the sequences of the oligonucleotides used for different constructs. Briefly, the 1,021-bp upstream and 1,044-bp downstream regions of the *spy_1476* gene were cloned in the multiple cloning sites (MCS)-I and MCS-II, respectively, of pFW6, and the resulting construct (pFW6Δ1476) was used to transform M1T1 5448 strain to obtain the M1T1ΔTyK mutant strain. A parallel control with empty plasmid (M1T1-WTspc) was created by introducing the *aad9* gene between *spy*_*1477* and the start of the *spy_1476* promoter-containing intergenic region. The genetic integrity of the mutant and the control strains was confirmed as described in Supplementary Methods. The pDC123 complementation vector (Chaffin and Rubens, 1998) was used to obtain M1T1ΔTyK::*tyk* complemented strain by inserting the wild-type *spy_1476 gene* along with its native promoter and ribosomal binding site (RBS). Growth patterns of the mutant, control, and complemented strains in THY broth were then compared with that of the wild-type strain using Polar Star Galaxy spectrofluorometer under the absorbance mode as described in the Supplementary Methods (Jin and Pancholi, 2006; Agarwal et al., 2011; Kant et al., 2015). GAS strains were subjected to cell fractionation as described in the Supplementary Methods. The specific distribution of SP-TyK in individual cell fractions of different GAS strains was identified by the Western Immunoblotting method using Anti-SP-TyK IgG antibodies.

### Microscopy

The early log phase (A_600_ = 0.4) cultures of M1T1-WT, M1T1-WTspc, mutant M1T1ΔTyK, and complemented M1T1ΔTyK::tyk strains were stained by FL-Van (BODIPY^®^ FL-conjugated vancomycin, 3 μg/ml, Invitrogen/Thermo) for 2 h. The stained bacteria were spread on the slides, air-dried, and observed under the Nikon Eclipse E600 fluorescence microscope attached with DS-Fi1C Nikon camera. The results were analyzed using NIS-Element viewer software v4.2.

Additionally, the late log phase cultures (A_600_ = 0.8) were washed with PBS, bacterial pellets were Gram- stained or fixed with freshly made fixative (2.5% glutaraldehyde and 4% paraformaldehyde in 0.1 M cacodylate buffer, pH 7.4) and subjected for field-emission gun-equipped scanning (FEI Nova NanoSEM 400) or Cryo-capable transmission electron microscopy (Technai G2 Spirit, EFI). The sample processing for SEM and TEM electron microscopy after fixation was done at the Ohio State University Microscopy Shared Resource (MSR) as described previously (Jin and Pancholi, 2006; Agarwal et al., 2011; Kant et al., 2015).

### *In vitro* Tyrosine-Phosphorylation/Dephosphorylation Assays

Autophosphorylation of SP-TyK (1 μg) was carried out in 30 μl of the phosphorylation reaction buffer (50 mM Tris/HCl pH 7.5 buffer, 10 mM MgCl_2_ or 10 mM MnCl_2_, 0.1 mM EDTA, 2 mM DTT, 0.01% Brij 35, and 10.0 μCi γ^32^P-ATP) at room temperature for 60 min in the presence or absence of poly-L-lysine (1.0 mM) (Jin and Pancholi, 2006; Kant et al., 2015). Poly-L-Lysine was added to increase the efficiency of the conventional autophosphorylation reaction condition (Fujita-Yamaguchi et al., 1989; Kyriakis and Avruch, 1990; Nguyen et al., 2017). Phosphorylated forms of SP-TyK were then resolved by SDS-PAGE and visualized by autoradiography and anti-TyrP antibodies in immunoblot analysis.

### Substrate Identification

The kinase activity of SP-TyK was studied as follows: Purified SP-STP, SP-PTP, CovR, WalR, SDH, SEN, and MBP proteins (1 μg each) were subjected to ^32^P-ATP-based phosphorylation reaction using the modified buffer containing 1 mM poly-L-lysine and 5 mM MnCl_2_ along with other contents of phosphorylation buffer in the presence and absence of SP-TyK (0.6 μg/reaction) as described above. After 60 min of incubation, the reaction was stopped by adding 4 × SDS-PAGE sample buffer. The phosphorylated proteins were resolved by SDS-PAGE using 15% gradient precast gel (GenScript), and subsequently Western blotted on PVDF membranes. The dried Coomassie-stained PVDF membranes were then subjected to autoradiography. The phosphorylation reaction buffer without SP-TyK for each targeted protein served as corresponding negative controls. Furthermore, confirmation of phosphorylation was carried out by LC-MS/MS mass-spectrometry analysis as described below.

### *In vivo* Phosphorylation

For *in vivo* phosphorylation, the mutant M1T1ΔPTP (Kant et al., 2015), lacking low molecular weight protein tyrosine phosphatase (SP-PTP), was complemented individually with the wild-type genes encoding His-WalR and HisCovR using pDC123 as described above. Briefly, these genes were separately cloned between KpnI and BamHI sites in pDC123 to create pDC-*His-walR* and pDC-*His-covR*. The *walR and covR*-complemented M1T1ΔPTP mutant strains were grown in THY broth containing 5 μg/ml chloramphenicol to a late log phase (O.D._620_ 0.8), and centrifuged. The resulting bacterial pellets were subjected to phage lysin treatment (Kant et al., 2015) to obtain whole cell lysates, which were then dialyzed against 50 mM Tris/HCl buffer pH 8.0 (2,000 M.W. cutoff). Individual dialyzed whole cell lysates were subjected to Ni-NTA column chromatography to purify putatively *in vivo* phosphorylated His-WalR and His-CovR. These preparations, thus obtained, were then subjected to mass spectrometry analysis to determine the *in vivo* phosphorylated residues of WalR and CovR as described below.

### Mass-Spectrometric Analysis of *Streptococcus pyogenes*-Tyrosine Kinase Protein and Phosphorylated Substrates

For mass spectrometry analyses, the autophosphorylation and phosphokinase reactions were carried out in cold ATP instead of radioactive ^32^P-ATP. Gel slices of both Coomassie-stained phosphorylated and non-phosphorylated SP-TyK and other substrates (SP-STP, CovR, WalR, and SDH) were subjected to LC-MS/MS mass-spectrometry analysis. Essentially, the mass spectrometry analysis was performed at OSU Comprehensive Cancer Center (CCC) Proteomic Shared Resource (PSR) as described in the Supplementary Methods. Peptides with a *p* value less than 0.02 with a score less than 20 were filtered. Protein identification was verified manually. Proteins with a *p*-value <0.05 and a Mascot score of ≤ 50 with a minimum of two unique peptides from one protein with a*-b*, or *–y* ion sequence tag of five residues or better were accepted. Furthermore, phosphoproteomic analysis of these data was carried out using MassMatrix software as described and manually validated (Xu and Freitas, 2007, 2008, 2009).

### Electrophoretic Mobility Shift Assay

Electrophoretic mobility shift assay (EMSA) experiments were performed using a 266-bp DNA probe (P*covR*) containing the promoter element downstream of the *spy_0281* and upstream of the *covR* gene (*spy*_*0282*) and a 454-bp DNA probe (P*ntpI*) encompassing the promoter element between the downstream of *spy*_*0124* and upstream of *spy*_*0125* genes. These probes were PCR-amplified using specific primer pairs, P*covR* Primers # 33/34, and P*ntpI* Primers # 35/36 (Supplementary Table 1). Briefly, the binding of the *in vitro* SP-TyK-phosphorylated, non-phosphorylated, CovR and VicR/WalR with the P*covR*, and P*ntpI* was carried out at the final volume of 40 μl of reaction mixer containing the probe (10 nM) with different concentrations (0.09–1.35 μM) of purified WalR and WalR-P, or CovR and CovR-P in the EMSA buffer [2 mg/ml poly dI-dC (Sigma), 10 mM Tris pH 7.5, 35 mM KCl, 1 mM EDTA pH 7.5, 1 mM DTT, 6% glycerol and 1 mM MgCl_2_]. The phosphorylated and non-phosphorylated WalR/VicR and CovR proteins were incubated with poly dI-dC containing reaction buffer for 5 min at room temperature, followed by the addition of other reaction components and further incubation for 25 min at room temperature. Protein-bound and free probes in the reaction mixtures after 30 min of incubation were then resolved by 6% non-denaturing polyacrylamide gel electrophoresis (200 V × 90 min) using 0.5 × TBE buffer. The gels were stained by 1:10,000 diluted SYBR^®^ Green EMSA nucleic acid gel stain (Thermo Fisher) in 1 × TBE buffer for 30 min and visualized using an LED (470 nm) transilluminator (LB-16^1^). The image obtained was then reverted by Photoshop software and subjected to densitometric analysis of the DNA band by the ImageJ software. The ratios of the density of free/non-shifted and the bound/shifted P*covR* DNA probe bands were calculated, and the line graph was plotted using GraphPad Prism-6 software.

### RNA-seq-Based Transcriptome Analysis

Analysis of the RNA-seq-based transcriptomes of the late-log phase grown cultures of the GAS strains (M1T1 WT, M1T1ΔTyK) was carried out essentially as described previously (Jin and Pancholi, 2006; Agarwal et al., 2011; Kant et al., 2015) and detailed in Supplementary Methods. Briefly, DNase I-treated high-quality total RNA from the bacterial lysates was extracted using the RNA purification kit (Norgen, Canada) per the instructions of the manufacturer, confirmed by an Agilent 2100 Bioanalyzer (Agilent Technologies, Palo Alto, CA, United States), and subsequently subjected to RNA-seq analysis in the commercial facility of Novogene Corp. Inc., (Durham, NC, United States). Libraries were sequenced using Illumina Hiseq 2000 using 100-bp sequencing. High-quality total RNA (20 μg) preparations obtained from three biological repeats from wild-type M1T1 and M1T1ΔTyK were subjected for RNA-seq transcriptome analysis. The data were analyzed using a popular-alignment tool Bowtie2 (Ver 2.4.2) (Langmead et al., 2009, 2019; Langmead and Salzberg, 2012). Differentially expressed genes (DEG) were determined based on fold changes (up- or downregulated) log_2_ ± 1, and p-values and corrected p-values (FDR/q-values/adjusted p-values) < 0.05. FDR value estimation was based on multiple hypothesis testing. Graphical representation of these values was presented in the form of a Volcano plot and cluster heat diagram. GO-enrichment analysis was carried out employing ClueGO analysis version 2.5.8 (Bindea et al., 2009, 2013) in Cytoscape version 3.8.2 (Shannon et al., 2003; Assenov et al., 2008).

### Quantitative Determination of TNF-α and IL-6

Secreted forms of IL-6 and TNF-α were quantitatively detected in the supernatants human lung epithelial A549 cell lines (CCL-185, American Type Culture Collection, ATCC) co-cultured with or without GAS strains for 3 and 4 h, using IL-6 (Cat#6050, D6RandD systems, Minneapolis, MN, United States) and TNF-α (BD OptEIA^TM^, cat#550610, B.D. Biosciences, Franklin Lakes, NJ, United States) ELISA-based detection kits per the instructions of the manufacturers. Purified IL-6 and TNF-α provided by the manufacturers were used as internal standards to obtain standard curves for quantitative analysis. Values obtained from uninfected cells were subtracted from the test samples. Data obtained from three independent experiments with duplicate samples were statistically analyzed by unpaired non-parametric Student’s t-test using GraphPad Prism-6 software. Values of p of mean ± S.D. less than 0.05 were treated as a significant difference.

### Mouse Infection Studies

Virulence of the M1T1-WT, M1T1ΔTyK mutant, and the *tyk*-complemented GAS strains was determined in the mouse peritonitis model (10–12 mice/group). CD-1 Swiss mice (5 weeks, 20–22 g Charles River Laboratories) were injected intraperitoneally with 5 × 10^8^ CFU of individual GAS strains. Morbidity and mortality in the infected animals were monitored twice daily for 10 days. All animal experiments were performed per the recommendations of the National Institute of Health Guide for the Care and Use of Laboratory Animals. The animal experiment protocol (2008A0222R4) was approved by the Ohio State University Institutional Animal Care and Use Committee (OSU-IACUC). These experiments were once repeated for reproducibility. Percentage survival in each group was combined and statistically evaluated by the Log Rank test (Kaplan–Meier) using GraphPad Prism 6 software. A value of *p* < 0.05 was treated as a significant difference.

### Bacterial Adherence and Invasion

Assays for GAS adherence to and invasion of A549 cells were carried out essentially as described previously (Jin and Pancholi, 2006; Agarwal et al., 2011; Kant et al., 2015). The confluent cultures of A549 cells, maintained in 12-well tissue culture plates, were co-cultured with M1T1 5448 wild-type, control strains, and the derived ΔTyK mutant and *tyk*-complemented GAS strains (multiplicity of Infection, MOI∼100: 1::bacteria: cell) for 3 h and adherent bacteria were enumerated employing the plate dilution method. Parallel to this assay, duplicate plates containing adherent bacteria of each strain were treated with penicillin (100 μg/ml) and gentamycin (50 μg/ml) for 90 min. The surviving bacteria were enumerated in the cell lysate as described above. The experiments were performed twice in four independent wells. The results were statistically analyzed, and the p-value significance was determined by an unpaired non-parametric t-test (Mann–Whitney test) using the GraphPad Prism 6 software.

### Biofilm Formation

The ability to form biofilms by GAS strains was determined using the crystal violet stain method. Briefly, the overnight cultures of control M1T1 wild-type, ΔTyK mutant, and *tyk*-complemented GAS strains were washed with sterile PBS and adjusted to O.D. 1.0 (∼5 × 10^8^ CFU/ml). Biofilm assays were carried out in 12-well tissue culture plates in a final volume of fresh 2.0 ml of THY broth seeded with an equal number of each GAS strain (1:100 dilution of O.D. 1.0 culture) and incubated for 72 h. At the end of incubation, the culture medium from each well was removed, and stained with 1% Crystal violet for 5 min, washed, and air-dried. The stained biofilms were then extracted and dissolved in 500 μl of 33% acetic acid. The aliquot (100 μl) from each extract was spectrophotometrically (λ = 492 nm) analyzed using a microtiter plate reader (BMG Polar Star Galaxy). The test samples were analyzed after subtracting the background values obtained from wells containing only the medium. All results were obtained from three independent experiments, each with four technical replicates. The results were statistically analyzed by unpaired non-parametric *t*-test using the GraphPad Prism 6 software as described above. A value of *p* < 0.05 was treated as a significant difference.

## Results

### Spy_1476 Is the First Reported *Streptococcus pyogenes* Putative Tyrosine Kinase

**FIGURE 1:**
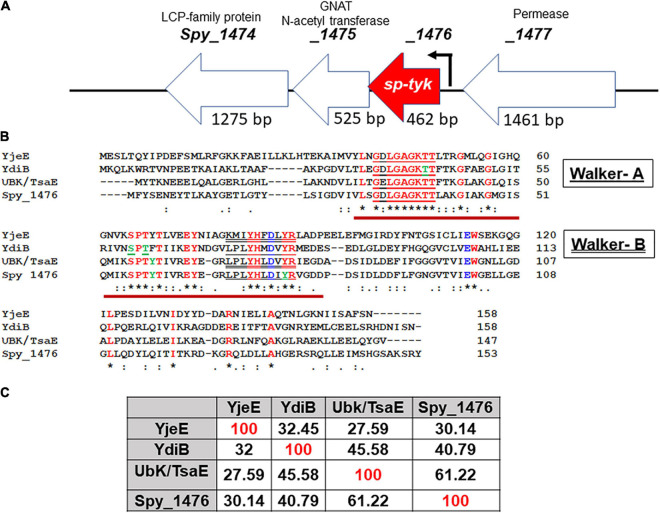
Genomic background of the *spy_1476*/*sp-tyk* gene and its comparison with other homologs. **(A)** The genomic environment of Spy_1476 (462 bp) and flanking genes. (see the conserved operon in gram-positive and gram-negative bacteria Supplementary Table 7). The flanking genes upstream *spy_1477* (1,461 bp) and downstream genes *spy_1475* (525 bp) and *spy_1474* (1,275 bp), respectively, encode permease, GNAT *N*-Acetyltransferase, and Lytr-Cps2A-Psr (LCP)-family protein. Arrow indicates transcription start site and the predicted promoter. **(B)** Protein sequence of Spy_1476 and its comparison with other homologs. Yje, YdiB, and UbK/TsaE were identified in *Haemophilus influenzae*, *Bacillus subtilis*, and *Streptococcus pneumoniae*, as indicated. Bold letters in red fonts are the conserved regions in these proteins. Letters in green fonts indicate the identified phosphorylated residues. Brown underline indicates catalytic site represented by Walker-A [red underline, GX(4)GKT(S)] and Walker–B (double underline, (RK)-X(3)-G-X(3)-L-h(3)D) motifs, where “h” stands for hydrophobic residues. Symbol “*” denotes identical conserved residues, and “**:**” denotes similar conserved residues. **(C)** Percent sequence identity of Spy_1476 with other homologs.

By bioinformatic analysis and motif/homology search within the MGAS5005 *S. pyogenes* genome, we predicted *spy_1476* and its homologs in other *S. pyogenes* genomes (annotated as protein-encoding gene with unknown function or a GTP/ATP hydrolase) as a putative 17 kDa Tyr-kinase. Its genome environment indicted that the gene *spy_1476* co-transcribed with the *spy_1475* gene encoding acetyltransferase, as their respective 3′ and 5′ 26 nucleotides overlap with each other (Figure 1A). Based on the protein sequence of the YjeE ATPase protein from *Haemophilus influenzae* (Teplyakov et al., 2002), we initially predicted that a putative Tyr- kinase would likely have one or both of two typical kinases-related nucleotide-binding Walker A [G-X(4)-GKT(S)] motif and also the divalent cation-binding Walker-B motif [(R.K.)-X(3)-G-X(3)-LhhhD] (Figure 1B). Recent characterization of the tyrosine kinase activity of *B. subtilis*, YdiB, (a Spy_1476/SP-TyK homolog) (Nguyen et al., 2017) revealed that this class of proteins belonged to a novel class of UbK (ubiquitous bacterial kinase) kinases, annotated as UPF0079 possessing Walker-B like motif [X(4)YHXDXYR]. Amino acid sequence comparison analysis of the Spy_1476 protein with *H. influenzae* YjeE, *Bacillus subtilis* YdiB, and *S. pneumoniae* UbK/TsaE proteins revealed 30%–61% identity with these homologs (Figure 1C).

### Spy_1476 Is an Autophosphorylating Tyrosine Kinase

**FIGURE 2:**
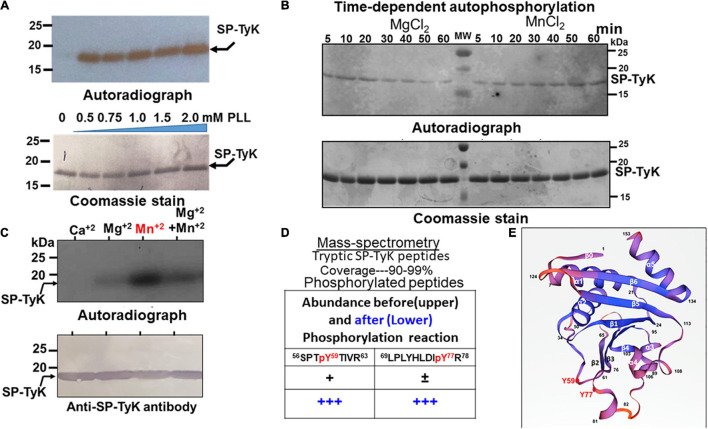
Autophosphorylating properties of Spy_1476. **(A)** Autoradiograph showing ^32^P-ATP –based autophosphorylation of the Ni-NTA affinity-purified recombinant 6XHis Spy_1476 (SP-TyK) carried out in the presence and absence of different concentrations of poly–L-lysine. **(B)** Poly-L-lysine and ^32^P-ATP -based autophosphorylation reaction in the presence and absence of 5 mM MgCl_2_ or MnCl_2_ at different time-points up to 60 min. Time-dependent phosphorylation reaction denotes MnCl_2_ as a preferred divalent cation cofactor and 60 min as the optimal incubation time. **(C)** The efficiency of autophosphorylation reactions in the presence of different cations employing an optimal reaction condition as described in panel **(B)**. In panels **(A–C)**, the upper panels denote autoradiographs, and except in panel **(C)**, the lower panels are the corresponding Coomassie-stained gel serving as loading controls. The lower panel in panel **(C)** is the corresponding Western immunoblot of the upper panel stained with Protein-A column purified Anti-SP-TyK rabbit polyclonal IgG and corresponding Alk-phosphatase-labeled conjugate antibody as visualized by using a colorimetric substrate. **(D)** Mass-spectrometric analysis of the tryptic peptides of *in vitro* autophosphorylated and non-phosphorylated Spy_1476. “±” and “**+”** indicate low abundance of phosphorylation as revealed by the manual examination of phosphorylated residues (>50 scores and *p* > 0.05). Residues in the red font denote phosphorylated residues (Tyr59 and Tyr77). **(E)** Three dimension structures of *Streptococcus pyogenes*-tyrosine kinase (SP-TyK) based on *H. influenzae* YjeE using SWISS port (PDB:1FL9) (Teplyakov et al., 2002). The structure shows seven stranded mixed β-sheet in the following order of residues: 1-5β0→7-18α1→25-30β1→37-48α2→(Y59)-61-65β2→71-75β3-(Y77)→78-80α3→89-92α4→97-101β4→103-110α 5→114-121β5→126-133β6→135-147α6. The phosphorylated Y59 and Y77 residues near the two adjacent loops (beginning of β2 and end of the β3 sheets) are highlighted with red closed circles.

Homologs of Spy_1476 in *B. subtilis* (Nguyen et al., 2017) and *S. pneumoniae* (Pelletier et al., 2019), previously identified as YdiB and UbK (universal bacterial kinase), respectively, have been found to display autophosphorylating activity both *in vivo* and *in vitro*. Recombinant Spy_1476 was, therefore, subjected to autophosphorylate *in vitro*. Spy_1476 was poorly autophosphorylated in radioactive ^32^γP-ATP-based autophosphorylation assays. Since the phosphorylation reaction of certain kinases is enhanced in the presence of poly-L-lysine (Fujita-Yamaguchi et al., 1989; Kyriakis and Avruch, 1990; Nguyen et al., 2017) and typically carried out between 30 and 60 min, similar reactions with Spy_1476 in the presence of different concentrations of poly-L-lysine (PLL, 0.5–2.0 mM) (Figure 2A) revealed autophosphorylation activity in a time-dependent manner with a peak activity between 50 and 60 min (Figure 2B). While this time-dependent phosphorylation reaction depended on Mn^+2^ or Mg^+2^ (Figure 2B), it was more efficient in the presence of Mn^+2^ than in Mg^+2^ divalent cations. The autophosphorylation of Spy_1476 decreased gradually after 30 min of incubation in the presence of 5 mM MgCl_2_, while it was continually increased in the presence of 5 mM MnCl_2_. Since certain tyrosine kinase, such as PYK2 is calcium-dependent (Matsui et al., 2007), the influence of CaCl_2_ on SP-TyK activity was examined. However, unlike MnCl_2_ and MgCl_2_, the Spy_1476 did not autophosphorylate in the presence of CaCl_2_, as confirmed by purified anti-Spy_1476/SP-TyK-specific rabbit polyclonal IgG antibody in Western blot analysis (Figure 2C). Subsequent mass-spectrometric-based phosphoproteomic analysis of the recombinant Spy_1476 protein after autophosphorylation reaction revealed two tyrosine residues, Tyr59 and Tyr77 (score < 20, *p* < 0.02), as the only phosphorylated residues of the autophosphorylated Spy_1476 (Figure 2D). The manual mass-spectrometry data evaluation of Spy_1476 before autophosphorylation revealed the phosphorylated Tyr59 residue in low abundance (score 52 and 54 in repeat experiment, shown as “+”). Similarly, Tyr77 was observed once at a score of 78 but not in the repeat experiment (shown as “±”), indicating that the *in vivo* autophosphorylation of SP-TyK likely occurred while expressing it in *E. coli*. The latter events that occurred before the *in vitro* phosphorylation reaction were, therefore, treated as non-significant. The observed *in vitro* dual phosphorylation at Tyr59 and Tyr77 might be influenced by their location in close proximity (before the start of β2-sheet and at the end of β3-sheet) as revealed from the simulated three dimensional seven-stranded mixed β-sheet ribbon structure (Figure 2E and Supplementary Table 5A). This information concluded that the protein Spy_1476 was the *bona fide S. pyogenes* tyrosine kinase, hence termed SP-TyK.

### *Streptococcus pyogenes*-Tyrosine Kinase Regulates Group A *Streptococcus* Cell Division, Morphology, and Chain Formation

The successfully derived and sequence-verified *S. pyogenes* (GAS) mutant lacking SP-TyK (M1T1ΔTyK) and control strains obtained with the empty plasmid met the necessary criteria of their genetic integrity without any polar effects (see Supplementary Method and Results and Supplementary Figures 1A–D). Western Immunoblot analyses with anti-SP-TyK IgG showing the absence of SP-TyK in supernatant fractions of the wild-type, wild-type control, and complemented GAS strains and all fractions of (supernatant, cell wall, cytoplasm, and membrane particulate) of the mutant as well as its presence essentially in the cytoplasmic and cell wall fractions of the wild-type, control wild-type, and complemented GAS strains confirmed that SP-TyK, which does not possess a signal sequence, is essentially a cytoplasmic protein (Supplementary Figure 1E). These results also revealed that the absence of SP-TyK adversely affected an overall pattern of the mutant’s growth. These findings suggested that the adversely affected growth pattern might be an outcome of directly or indirectly affected cell division processes and cell morphology.

**FIGURE 3:**
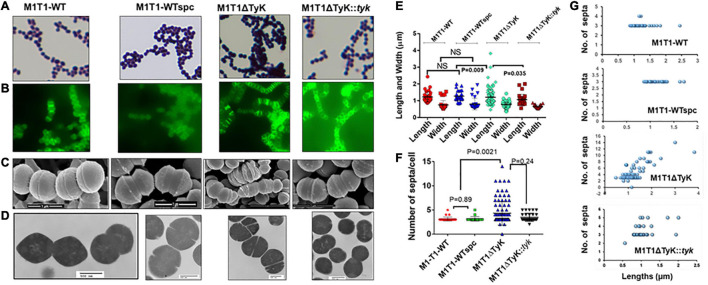
Microscopy of the M1T1-WT, M1T1ΔTyK mutant, and M1T1ΔTyK::*tyk* complemented Group A *Streptococcus* (GAS) strains. **(A–D)** GAS strains as observed by **(A)** Gram’s stain, **(B)** Van-Fl stain (Fluorescence microscopy), **(C)** scanning electron microscopy, and **(D)** Transmission electron microscopy. Scale bar in electron micrograph as indicated. **(E)** Quantitative analysis of the lengths and widths and **(F)** septa of individual GAS strains based on multiple fields. **(G)** Length vs. the number of septa of each GAS strain’s abnormal and normal individual bacteria based on multiple microscopic fields scanned during scanning electron microscopy. Scattered plots with horizontal bars show the median size in μm ± S.D. as determined using GraphPad Prism 6. *p* < 0.05 was treated as a significant difference and determined using non-parametric unpaired Student’s *t*-test.

Initial microscopic examination of Gram-stained M1T1ΔTyK revealed the formation of aggregation and long-chain formation (Figure 3A). These changes became more apparent by staining GAS strains with fluorescence-labeled vancomycin (Van-FL) (Figure 3B) as well as scanning (Figure 3C and Supplementary Figure 2) and transmission electron microscopy (Figure 3D). Together, these examinations revealed aggregated forms with long chains and irregularly spaced multiple parallel septa. About 10% of abnormal cells showed elongated tube-like or bulging structures with 10–15 parallel or irregular multidirectional septa. The resulting abnormal morphology was distinct from that of the wild-type and complemented strains, which showed two to three distinct, well-formed septa and cell walls between and around two cells of the chain. Quantitative morphological analyses based on multiple fields (15–30) with randomly selected three to four clusters of bacteria revealed that the mean lengths, widths, and the number of septa of the wild-type, M1T1-WTspc and complemented strains did not show significant differences from those of the mutant except in the number of septa per cell (Figures 3E,F). In this regard, the mutant displayed 43.4 ± 5.0% of abnormal cells vs. 2.0% ± 0.4% of wild-type control strains and 7.0% ± 2.3% of complemented strains (*p* < 0.009) (Figures 3E,F), and the number of septa per M1T1ΔTyK mutant was proportional to the individual bacterial cell length (Figure 3G). Together, these results indicated that SP-TyK plays a crucial role in the homeostasis of the GAS cell division process, and its absence resulted in abnormal shapes and sizes and increased parallel and non-directional septa formation in GAS.

### *Streptococcus pyogenes*-Tyrosine Kinase Differentially Regulates the Expression of a Variety of Metabolic-,Cell Division-, and Virulence-Related Genes

The enhanced cell division activity and reduction in growth in the absence of SP-TyK suggested that the expression of corresponding cell division- and cell metabolism-related genes might be differentially regulated. The RNA-seq-based transcriptome analysis of the M1T1ΔTyK mutant vs. M1T1 wild-type strain covering ∼99% genes (1,846 of 1,865 ORFs) (Supplementary Table 2) revealed a total of 287 differentially expressed genes (DEGs) of which 146 genes (51%, Table 1 and Supplementary Table 3) were upregulated and 141 genes (49%, Table 2 and Supplementary Table 4) were downregulated.

**TABLE 1:** Transcript abundance of the major upregulated clustered genes in *Streptococcus* (*S.) pyogenes*. in the absence of SP-TyK.

Gene_id	Locus	Protein name	log2-Fold	Linear fold	*p*-Val	*p*-Adj
	**Cell division genes**					
**Spy0308**	**scpA**	**Segregation and condensation protein A**	**1.1273**	**2.184495**	**0.00237**	**0.021435**
**Spy0309**	**scpB**	**Segregation and condensation protein B**	**1.0119**	**2.016565**	**0.00676**	**0.04535**
**Spy0531**	**ftsE**	**Cell division ATP-binding protein**	**1.2094**	**2.312414**	**0.00053**	**0.007268**
**Spy0532**	**ftsX**	**Cell division protein**	**0.97367**	**1.96383**	**0.00327**	**0.027264**
Spy1244	divIVAS	Cell division initiation protein	0.80863	1.751547	**0.01702**	0.083883
**Spy1249**	**ftsZ**	**Cell division protein FtsZ**	**1.0243**	**2.033972**	**0.00368**	**0.02901**
**Spy1250**	**ftsA**	**Cell division protein**	**1.4569**	**2.745179**	**3.21E−05**	**0.000992**
**Spy1866**	**parB**	**Chromosome partitioning protein**	**0.93487**	**1.911718**	**0.00561**	**0.039333**
	**F_1_-F_0_-ATPase**					
Spy0575	atpE	ATP synthase F0F1 subunit C	0.31794	1.24655	0.3292	0.55129
**Spy0577**	**atpF**	**ATP synthase F0F1 subunit B**	**1.2454**	**2.37084**	**0.00033**	**0.005208**
Spy0578	atpH	ATP synthase F0F1 subunit delta	0.66233	1.58264	**0.03874**	0.1469
**Spy0579**	**atpA**	**ATP synthase F0F1 subunit alpha**	**1.723**	**3.30122**	**9.09E−07**	**6.48E−05**
**Spy0580**	**atpG**	**ATP synthase F0F1 subunit gamma**	**1.614**	**3.06099**	**4.65E−06**	**0.00025**
**Spy0581**	**atpD**	**ATP synthase F0F1 subunit beta**	**1.7284**	**3.31360**	**9.23E−07**	**6.48E−05**
**Spy0582**	**atpC**	**ATP synthase F0F1 subunit epsilon**	**1.5044**	**2.83707**	**1.87E−05**	**0.000681**
	**Virulence-related genes**					
**Spy1145**	**sodA**	**Superoxide dismutase**	**1.3862**	**2.61389**	**2.91E−05**	**0.00092192**
**Spy1735**	**speB**	**Exotoxin B**	**1.1591**	**2.23318**	**0.0004729**	**0.0068463**

*Bold numbers and corresponding genes are significantly differentiated (up or downregulated) (log2 = ≥ 1 or ≤ −1).*

**TABLE 2:** Transcript abundance of the major down-regulated clustered genes in *S. pyogenes* in the absence of SP-TyK.

Gene_id	Locus	Protein name	log2-fold	Linear fold	*p*-val	*p*-adj
**PTS-transport system**						
Spy0149	-	PTS system 3-keto-L-gulonate specific transporter subunit IIB	**−2.5998**	**−6.06203**	**0.02112**	0.097536
**Spy0150**	**-**	**PTS system 3-keto-*L*-gulonate specific transporter subunit IIA**	**−2.4934**	**−5.63103**	**0.00146**	**0.015194**
**Spy0519**	**agaD**	**PTS system *N*-acetylgalactosamine-specific transporter subunit IID**	**−1.121**	**−2.17498**	**0.00769**	**0.048627**
Spy1079	-	PTS system cellobiose-specific transporter subunit IIC	**−0.8157**	**−1.76015**	**0.03304**	0.13248
**Spy1083**	**-**	**PTS system, mannitol (cryptic)-specific IIA component**	**−1.1493**	**−2.21806**	**0.00297**	**0.025409**
Spy1662	ulaA	PTS system ascorbate-specific transporter subunit IIC	**−1.147**	**−2.21453**	**0.02441**	0.1086
**Spy1744**	**-**	**PTS system cellobiose-specific transporter subunit IIC**	**−1.3317**	**−2.51699**	**0.00057**	**0.007597**
Spy1745	-	PTS system cellobiose-specific transporter subunit IIB	**−1.8132**	**−3.51421**	**0.04485**	0.162
**Spy1746**	**-**	**PTS system cellobiose-specific transporter subunit IIA**	**−1.7089**	**−3.26911**	**0.00201**	**0.019124**
**Spy1399**	**-**	**PTS system galactose-specific transporter subunit IIC**	**−3.5653**	**−11.8376**	**0.00233**	**0.021278**
**Spy1400**	**-**	**PTS system galactose-specific transporter subunit IIB**	**−3.4328**	**−10.7988**	**0.00331**	**0.027264**
**Spy1401**	**-**	**PTS system galactose-specific transporter subunit IIA**	**−3.2836**	**−9.73783**	**0.00752**	**0.048447**
**Spy1542**	**scrA**	**PTS system sucrose-specific transporter subunit IIABC**	**−2.0758**	**−4.21578**	**0.00025**	**0.004466**
Spy1633	lacE	PTS system lactose-specific transporter subunit IIBC	**−3.2071**	**−9.23492**	**0.0129**	0.071094
Spy1634	lacF	PTS system lactose-specific transporter subunit IIA	**−2.7073**	**−6.53098**	**0.01083**	0.062301
Spy1664	-	PTS system, mannitol (cryptic)-specific IIA component	**−1.4414**	**−2.71584**	**0.02409**	0.10771
**Spy1692**	**-**	**PTS system glucose-specific transporter subunit IIABC**	**−1.187**	**−2.27679**	**0.00082**	**0.009789**
**Spy1784**	**-**	**PTS system, trehalose-specific IIBC component**	**−3.3758**	**−10.3805**	**2.49E−17**	**2.27E−14**
**Vo-V1 Na^+^—ATPase**						
**Spy0126**	**ntpI**	**V-type ATP synthase subunit I**	**−2.5024**	**−5.66627**	**0.00136**	**0.014628**
**Spy0127**	**ntpK**	**V-type ATP synthase subunit K**	**−**2**.4527**	**−5.4744**	**0.00065**	**0.008203**
**Spy0128**	**ntpE**	**V-type sodium ATP synthase subunit E**	**−2.7724**	**−6.83244**	**0.00053**	**0.007268**
**Spy0129**	**ntpC**	**V-type ATP synthase subunit C**	**−2.9325**	**−7.63432**	**0.00459**	**0.034141**
**Spy0130**	**ntpF**	**V-type ATP synthase subunit F**	**−2.6003**	**−6.06413**	**0.00064**	**0.008131**
**Spy0131**	**ntpA**	**V-type ATP synthase subunit A**	**−2.4719**	**−5.54774**	**0.00258**	**0.022735**
**Spy0132**	**ntpB**	**V-type ATP synthase subunit B**	**−2.4977**	**−5.64784**	**0.0041**	**0.031426**
**Spy0133**	**ntpD**	**V-type ATP synthase subunit D**	**−2.7435**	**−6.69693**	**0.00306**	**0.026053**
**Lactose metabolism and transport**						
**Spy1395**	**lacD.1**	**Tagatose 1,6-diphosphate aldolase**	**−4.0745**	**−16.8479**	**0.00341**	**0.027554**
**Spy1396**	lac.1*	**Tagatose-6-phosphate kinase**	**−4.1343**	**−17.561**	**0.00243**	**0.021743**
**Spy1397**	**lacB.1**	**Galactose-6-phosphate isomerase subunit LacB**	**−4.0835**	**−16.9534**	**0.00257**	**0.022735**
**Spy1398**	**lacA.1**	**Galactose-6-phosphate isomerase subunit LacA**	**−3.8108**	**−14.0335**	**0.00194**	**0.01875**
**Spy1399**	**PTS-IIC**	**PTS system galactose-specific transporter subunit IIC**	**−3.5653**	**−11.8376**	**0.00233**	**0.021278**
**Spy1400**	**PTS-IIC**	**PTS system galactose-specific transporter subunit IIB**	**−3.4328**	**−10.7988**	**0.00331**	**0.027264**
**Spy1401**	**PTS-IIA**	**PTS system galactose-specific transporter subunit IIA**	**−3.2836**	**−9.73783**	**0.00752**	**0.048447**
**Spy1402**	lacR.1	Lactose phosphotransferase system repressor	**−1.5411**	**−2.91016**	**0.02947**	0.12272
Spy1632	lacG	6-phospho-beta-galactosidase	**−3.1083**	**−8.62366**	**0.00867**	0.05255
Spy1633	lacE	PTS system lactose-specific transporter subunit IIBC	**−3.2071**	**−9.23492**	**0.0129**	0.071094
Spy1634	LacF	PTS system lactose-specific transporter subunit IIA	**−2.7073**	**−6.53098**	**0.01083**	0.062301
Spy1635	lacD.2	Tagatose 1,6-diphosphate aldolase	**−2.3671**	**−5.15903**	**0.01165**	0.065405
**Spy1636**	**lacC.2**	**Tagatose-6-phosphate kinase**	**−1.7235**	**−3.30237**	**0.00153**	**0.015543**
Spy1637	lacB.2	Galactose-6-phosphate isomerase subunit LacB	**−1.7958**	**−3.47208**	**0.01445**	0.076593
Spy1638	lacA.2	Galactose-6-phosphate isomerase subunit LacA	**−1.8966**	**−3.72335**	**0.01878**	0.08973
Spy1639	lacR.2	Lactose phosphotransferase system repressor	**−**0.7302	**−**1.658869	0.18997	0.40212
***N*-acetyl neuraminate transport**						
**Spy0213**	-	***N*-acetylneuraminate-binding protein**	**−1.5166**	**−2.86116**	**0.00013**	**0.002885**
**Spy0214**	-	***N*-acetylneuraminate transporter permease**	**−1.9678**	**−3.91171**	**1.12E−05**	**0.000474**
**Spy0215**	-	***N*-acetylneuraminate transporter permease**	**−2.098**	**−4.28115**	**4.64E−07**	**3.98E−05**
**Spy0216**	-	**Hypothetical protein M5005_Spy_0216**	**−2.2867**	**−4.87939**	**2.28E−07**	**2.60E−05**
**Spy0217**	nanH	***N*-acetylneuraminate lyase**	**−2.1783**	**−4.5262**	**2.27E−06**	**0.000138**
**Ferrichrome transport**						
Spy0321	fhuG	**ferrichrome transporter permease**	**−1.3179**	**−2.49303**	**0.00086**	**0.010212**
Spy0322	fhuB	**ferrichrome transporter permease**	**−1.1354**	**−2.19679**	**0.00487**	**0.03547**
Spy0323	fhuD	**Ferrichrome-binding protein**	**−1.0591**	**−2.08363**	**0.00773**	**0.048648**
Spy0323	fhuA	**Ferrichrome ABC transporter ATP-binding protein**	**−1.164**	**−2.24078**	**0.011777**	**0.065895**
**Virulence-related genes**						
Spy0139	nga	NAD glycohydrolase	**−**1.036	**−2.05053**	**0.0545**	0.18547
Spy0140	-	Hypothetical protein M5005_Spy_0140	**−**1.2788	**−2.42637**	**0.01825**	0.088288
**Spy0141**	**slo**	**Streptolysin O**	**−1.4722**	**−2.77445**	**0.00729**	**0.04787**
**Spy0351**	**spyA**	**C3 family ADP-ribosyltransferase**	**−1.059**	**−2.08349**	**0.00311**	**0.026272**
**Spy0562**	**sagA**	**Streptolysin S**	**−1.1917**	**−2.28422**	**0.0005**	**0.006941**
**Spy0668**	**mac**	**IgG-degrading protease**	**−2.607**	**−6.09235**	**0.00153**	**0.015543**
Spy1169	spd3	Streptodornase	**−**0.8225	**−**1.7684679	**0.02474**	0.10899
Spy1684	ska	Streptokinase	**−**0.8729	**−**1.8313404	**0.01577**	0.079648
Spy1318	rocA	**Sensory transduction protein kinase**	**−**0.9818	**−1.97493**	**0.01527**	0.078922
Spy1714	-	**Cell surface protein**	**−1.0042**	**−2.00583**	**0.04635**	0.16478
Spy1715	scpA	**C5A peptidase**	**−1.0364**	**−2.0511**	0.19829	0.4143
**Spy1718**	**sic1.01**	**Inhibitor of complement protein**	**−1.5132**	**−2.85442**	**3.60E−05**	**0.001046**
Spy1719	emm1.0	M protein	**−1.1325**	**−2.19238**	**0.04401**	0.15987
Spy1720	mga	*Trans-*acting positive regulator	**−0**.7263	**−**1.6543907	**0.03105**	0.12725
Spy1851	hasA	Hyaluronan synthase	**−**2.2063	**−4.6149**	**0.0384**	0.14621
Spy1852	hasB	UDP-glucose 6-dehydrogenase	**−**1.2283	**−2.34291**	**0.02577**	0.11168

*P values in Bold numbers and corresponding genes are significantly differentiated (up or down regulated) (log2 = ≥ 1 or ≤ **−**1).*

**FIGURE 4:**
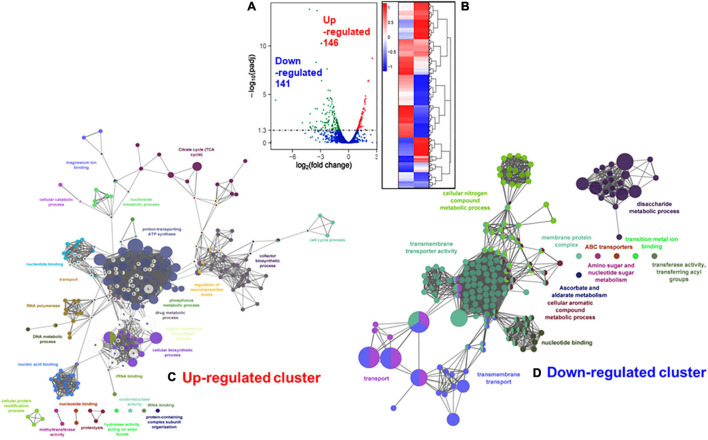
Comparison with RNA-seq based differentially expressed genes (DEGs) in M1T1ΔTyK mutant vs. M1T1 wild-type GAS strain. **(A)** Volcano plot for DEGs. The X-axis shows the fold change in gene expression between different samples, and Y-axis shows the statistical significance of the differences [-log_10_.(padj/q-value/FDR)]. Significantly up and Downregulated genes are highlighted in red and green dots, respectively. Blue dots denote genes showing no significant differential expression. **(B)** Cluster Heat MAP of DEGs in M1T1-WT and M1T1ΔTyK. The adjacent scale shows up or downregulated DEGs in Log_2_ values. Statistical evaluation of the RNA-seq data was performed as described in the “Materials and Methods” section. ClueGo- and Cytoscape-based analysis of the clustered **(C)** upregulated and **(D)** downregulated genes.

The ClueGO and Cytoscape cluster analysis of the Volcano-plotted (Figure 4A) and Heat-MAP (Figure 4B) analyzed 287 DEGs revealed noticeable changes in the expression of transport-related genes. In particular, this analysis revealed upregulated genes of ATP pump/ATPase FoF1 pathway (*spy_576-spy_582*) and lipid metabolism-related genes *(spy_1489-spy_1492, spy_1344-spy_1346*) (Figure 4C, Table 1, and Supplementary Figure 3). Among 13 two-component regulatory systems, only the *ciaHR* genes (*spy_947-spy_948*) were found to be upregulated. No substantial changes in the transcript abundance of virulence-related genes were observed except the upregulation of *speB* (exotoxin B, *spy_1735*), and *sodA* (superoxide dismutase, *spy_1145*). Two other clusters of highly up-regulated genes belonged to ribosomal biosynthesis (*spy_0049—spy_0069*) and cell division processes (*ftsE-spy_0531, ftsX-spy_0532, ftsZ-spy_1249, ftsA-spy_1250*, ParB/chromosomal partitioning protein-encoding gene *spy_1866*). Similarly, the downregulated genes included several genes belonging to (i) V-ATP synthase (*spy_125-spy_133*), (ii) ferrichrome transport system (*spy_321-spy_323*), (iii) lactose/galactose transport/metabolism genes (*spy_1395-spy_1401, spy_1636*), (iv) PTS systems responsible for various sugar transport (11 genes), (v) *N*-acetylneuraminate metabolism (*spy_0213-spy_0218*), (vi) trehalose transport (*spy_1783-spy_1784*), and (vii) cellobiose/sorbitol transport (*spy_1744-spy_1750*) (Table 2, Figure 4D and Supplementary Figure 4). The downregulated genes also included four virulence–related genes, *spyA* (C3 family ADP-ribosyl transferase, *spy_0351*), *slo* (Streptolysin O, *spy_0141*), and *sagA* (Streptolysin S, *spy_0562*), and one cell wall-associated surface protein-encoding gene. Together, these results indicated that the bacterial cell division process was enhanced in the absence of SP-TyK-mediated post-translational modifications. Still, at the same time, the GAS metabolic fitness was compromised.

### *Streptococcus pyogenes*-Tyrosine Kinase Is Not Dephosphorylated by *Streptococcus pyogenes* Protein Tyrosine Phosphatase, a Low Molecular Weight Tyrosine Phosphatase

**FIGURE 5:**
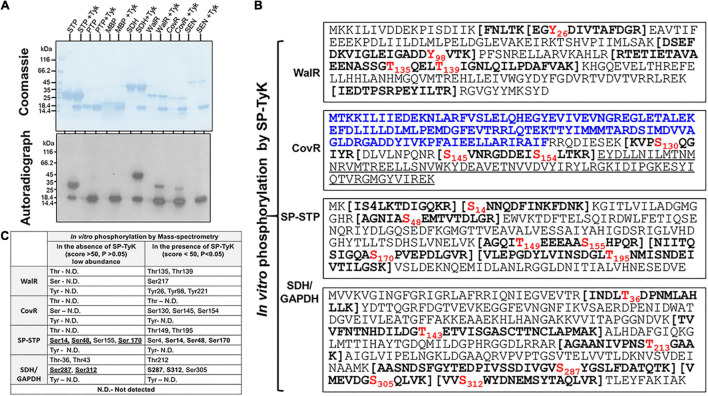
SP-TyK-targeted *in vitro* substrates and their specific amino acid residues identification and biological relevance. **(A)** Identification of substrates by examining kinase activity of SP-TyK. SDS-PAGE-resolved individual proteins (SP-STP, SP-PTP, MBP, SDH, WalR, CovR, and SEN 1 μg each) and corresponding autoradiographs show *in vitro* phosphorylation (as described in Figure 2) in the presence and absence of SP-TyK (Tyk, 0.6 μg/reaction). Coomassie-stained SDS-PAGE gel with resolved protein bands depicts loading control for each protein. The corresponding autoradiograph shows the phosphorylated substrates only in the presence of SP-TyK. SP-TyK is autophosphorylated. SP-STP, SDH, CovR, and WalR show robust phosphorylation. SP-STP, *S. pyogenes* eukaryote-type Ser/Thr phosphatase; SP-PTP, *S. pyogenes*protein tyrosine phosphatase; MBP, myelin basic-protein; SDH, streptococcal surface dehydrogenase; WalR, cell wall metabolism response regulator; CovR, control of virulence regulator; SEN (streptococcal surface enolase). **(B)** Mass-spectrometry analysis of the SP-TyK-phosphorylated substrates was carried out as described in the “Materials and Methods” section. Peptide sequences with bold letters within the bracket(s) indicate tryptic peptides identified with one or more phosphorylated residues (shown in Red font). The N-terminal sequence of CovR in blue fonts (the N-terminal half) is a regulator-CovRR, and the underlined sequence in its C-terminal half is a DNA-binding region-CovRB). **(C)** Mass-spectrometry analysis of the WalR, CovR, SP-STP, and SDH/GAPDH after the phosphorylation reaction carried out in the absence and presence of SP-TyK enzyme using cold ATP. Detection of certain phosphorylated residues in the absence of SP-TyK at low abundance and at high score (>50) low probability (*p* > 0.05) are treated as physiologically non-significant/background noise. Residues in bold fonts represent their detection before and after SP-TyK treatment.

Based on our previous study on the dual-specificity of orphan phosphatase, SP-PTP (Kant et al., 2015), the dephosphorylation of SP-TyK by SP-PTP was examined in one-step (together with SP-TyK and SP-PTP) and two-steps (autophosphorylation followed by dephosphorylation) reactions conditions. Irrespective of the design of the experiment, only one phosphorylated band (Figure 5A, lanes 3 and 4) of SP-TyK was detected by autoradiography. Because of the co-migratory nature of SP-PTP (145 aa, MW 16,954 Da) and SP-TyK (153 aa, MW 17,042 Da), the phosphorylated and/or dephosphorylated status of SP-PTP and SP-TyK could not be discerned. Hence, the above reversible phosphorylation reaction was carried out in the presence of cold ATP and subjected to Mass spectrometric analysis. Only SP-TyK was found to be phosphorylated at Tyr59 and Tyr77 residues as described above (Figure 2D). Identification of Tyr59 and Tyr77 residues in SP-TyK and the absence of any phosphorylated residues within the entire sequence of SP-PTP indicated that SP-PTP does not serve as a cognate phosphatase or a substrate of SP-TyK.

### Kinase Activity of *Streptococcus pyogenes*-Tyrosine Kinase and *in vitro* Identification of *Streptococcus pyogenes*-Tyrosine Kinase-Targeted Substrates

Based on the global transcriptome analysis of the mutant vs. wild-type, we predicted physiologically relevant potential targets of SP-TyK. The expression levels of specific carbohydrate metabolism-related genes and virulence genes such as *speB*, *SagA*, and *Slo*, as described above, are regulated by the two-component regulator, CovR (Levin and Wessels, 1998; Heath et al., 1999; Graham et al., 2002). CovR also regulates the expression of Spy*_0019/CdhA/SibA* (Pancholi et al., 2010), *gapdh/plr/sdh* (Pancholi and Fischetti, 1992), and *ciaRH* (Graham et al., 2002). CdhA/SibA (cell division plane-recognizing, and chain-forming cell wall hydrolase) is also regulated by SP-STK/SP-STP-modified WalR two-component regulator in GAS (Pancholi et al., 2010; Agarwal et al., 2011), as well as its homologs in *S. aureus* (Howell et al., 2003; Dubrac et al., 2007, 2008; Delaune et al., 2011). We, therefore, hypothesized that SP-TyK might target CovR and/or WalR, and their phosphorylation and post-translationally modified forms might regulate the expression of CovR/WalR-regulated genes. To test this hypothesis, the CovR and WalR regulators were considered as potential substrate targets of SP-TyK. In addition, many bacterial glycolytic enzymes also serve as substrates for serine/threonine kinases (Dannelly et al., 1989; Lomas-Lopez et al., 2007; Pietack et al., 2010). We, therefore, surmised that upregulated GAPDH and SEN/Enolase might serve as potential substrates or if not, can serve as negative control(s). Similarly, certain Ser/Thr phosphatases are also phosphorylated by tyrosine kinases (Vogel et al., 1993; Dohadwala et al., 1994). We, thus, examined two known phosphatases SP-PTP and SP-STP, along with CovR, WalR, GAPDH, and Enolase (SEN), as potential substrates for SP-TyK *in vitro* phosphorylation. MBP (myelin basic protein) was included as a non-specific positive control protein.

The *in vitro* phosphorylation of these purified recombinant proteins, carried out in the presence and absence of SP-TyK, revealed robust phosphorylation of SP-STP, CovR, WalR, GAPDH but not SP-PTP and Enolase (Figure 5A). Interestingly, the non-specific protein MBP was not phosphorylated by SP-TyK, indicating that SP-TyK does not target non-specific substrates. The mass-spectrometry analyses of individual SP-TyK-treated and untreated (control) SP-STP, CovR, WalR, and GAPDH (Figure 5B and Supplementary Tables 5B–E) revealed only WalR with tyrosine phosphorylation at its residues Tyr26, Tyr98, and Tyr221. WalR, as well as all other proteins, displayed either Ser and/or Thr residues. While both Thr and Ser (Thr135, Thr139, and Ser 217) residues were phosphorylated in WalR, only three Ser residues (Ser130, Ser145, and Ser154 residues) belonging to the DNA-binding sites were phosphorylated in CovR. CovR or WalR did not show phosphorylation at any residues in the absence of SP-TyK. While the SP-TyK-phosphorylated SP-STP revealed six phosphorylated residues (Ser4, Ser14, Ser48, Thr149, Ser170, and Thr195), SDH/GAPDH showed four phosphorylated residues (Thr212, Ser305, Ser287, Ser312). The phosphorylated amino acid residues of SP-STP and SDH in the absence of SP-TyK were detected with low probability (score > 50 and *p*-values > 0.05) (Figures 5B,C), and hence, were treated as non-specific or background phosphorylation that might have occurred during their expression in *E. coli*. Together, these results indicated that SP-TyK exhibited tyrosine kinase, as well as Ser/Thr kinase activities, although its autophosphorylation activity was exclusively directed to Tyr residues.

### *In vivo* Phosphorylation of Cell Wall Metabolism Response Regulator and Control of Virulence Regulator

**FIGURE 6:**
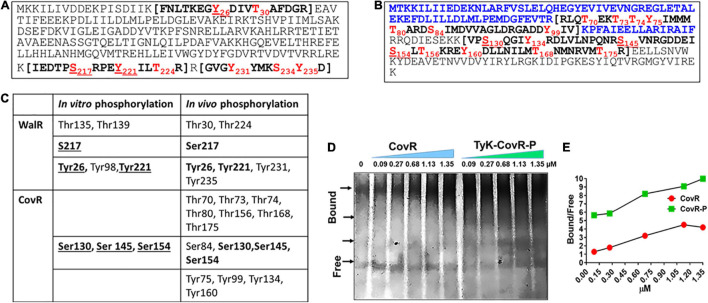
*In vivo* phosphorylation of WalR and CovR. **(A,B)** Mass-spectrometry analysis of the *in vivo* phosphorylated WalR **(A)** and CovR **(B)**. *In vivo* phosphorylation of His-WalR and His-CovR was achieved by purifying Ni-NTA affinity column-purified His-WalR and His-CovR from the whole-cell extract of the M1T1ΔSP-PTP::*His-walR* or M1T1ΔSP-PTP::*His-covR* strain constructed by complementing M1T1ΔPTP mutant with the *His-walR* or *His-covR* gene using pDC123 plasmid. Phosphorylated residues are shown in red fonts The N-terminal sequence of CovR shown in blue fonts (the N-terminal half) is a regulator-CovRR, and the sequence in the bracket are the trypsin fragments detected by Mass-spectromtery. (see also Supplementary Table 6 for the detailed mass spectrometry analysis). **(C).** Comparison with *in vitro* and *in vivo* phosphorylated residues of WalR and CovR. Residues in bold fonts depict their presence both *in vivo* and *in vitro* under the provided conditions. **(D)** Electrophoretic Mobility Shift Assay (EMSA), showing the binding of purified non-phosphorylated and phosphorylated CovR with the promoter *covR*(P*covR*) at different concentrations as indicated. Free and differentially migrated bound forms of the P*covR* DNA probe (266 bp) bands were resolved on 6% native gel and visualized using SYBR Green stain as described in the “Materials and Methods” section (see Supplementary Figure 4). Arrows indicate the positions of differentially migrated bands of the bound DNA probe with and without CovR and SP-TyK-phosphorylated CovR-P. The image is the PhotoShop software-converted revert image of the original image of the SYBR green-stained native gel (see Supplementary Figure 5). **(E)** Bound and free bands were quantitatively analyzed by spot densitometric analysis using the ImageJ software. Each data point in the line graph shows a ratio of Bound vs. Free arbitrary densitometric units of P*covR* preincubated with or without different concentrations (0.09–1.35 μM) of non-phosphorylated CovR and SP-TyK-phosphorylated CovR-P.

Mass-spectrometric analysis of the purified His-tagged WalR and CovR expressed in the GAS mutant lacking SP-PTP revealed that in addition to *in vitro*-phosphorylated Tyr26, Ser217, and Tyr221 residues of WalR, additional three residues Thr224, Tyr231, and Tyr235, were also found to be *in vivo* phosphorylated (Figures 6A,C and Supplementary Table 6). Similarly, while *in vitro* phosphorylation of CovR revealed phosphorylation at only three residues (Ser130, Ser145, Ser154) belonging to the DNA-binding domain (CovRB), *in vivo* phosphorylation of CovR revealed additional 12 phosphorylated residues spanning both response (CovRR), as well as DNA binding (CovRB) domains. These different phosphorylated amino acids included seven Thr-residues (Thr70, Thr73, Thr74, Thr80, Thr156, Thr168, and Thr175), one Ser residue (Ser84), and four Tyr residues (Tyr75, Tyr99, Tyr134, and Tyr160) (Figures 6B,C and Supplementary Table 6). Discrepancies and similarities of *in vitro* vs. *in vitro* phosphorylated residues of WalR, and CovR (Figure 6C) indicated that unlike static *in vitro* conditions, the dynamic conformational changes occurring in the protein in different *in vivo* physiological conditions might affect the accessibility of specific residues to be phosphorylated.

The location of phosphorylated residues of CovR and WalR in their respective DNA-binding C-terminal half regions indicated that the SP-TyK phosphorylation might influence the binding ability of CovR and WalR to their respective promoters. As much as the information on GAS WalR regulon is minimal (Liu et al., 2006; Vega et al., 2016) to none, the transcript abundance of its predicted target genes, e.g., *SibA/cdhA/spy_0019* (Liu et al., 2006; Pancholi et al., 2010), and an operon that encodes putative mannose/fructose EII (Spy_1058-Spy_1060) (Liu et al., 2006) was also found to be unaltered by RNA-seq analysis. Since the transcript abundance of V-type ATP pump encoding genes was highly downregulated in the mutant, we examined by the binding affinity of purified WalR and SP-TyK-phosphorylated WalR-P for P*ntpI* containing a putative WalR-binding motif similar to “TGTWAHTGTWAH” (Dubrac et al., 2007, 2008). However, we could not find any difference in binding pattern (data not shown). On the other hand, in similar EMSA experiments of CovR binding to its promoter (Gusa and Scott, 2006) revealed ∼fourfold higher P*covR*-binding capacity of the SP-TyK-phosphorylated CovR-P compared to the control non-phosphorylated CovR at their various concentrations (0.09–1.35 μM) as revealed by densitometric analysis (Figures 6D,E and Supplementary Figure 5). Thus, a 7- to 14-fold higher concentration of non-phosphorylated CovR (0.68–1.35 μM) vs. 0.09 μM of CovR-P was required to attain the equivalent binding to P*covR* (Figures 6D,E and Supplementary Figure 5). Together, these results indicated that SP-TyK-mediated phosphorylation of CovR potentially influenced the expression of specific CovR-regulated genes as observed by RNA-seq analysis.

### *Streptococcus pyogenes*-Tyrosine Kinase Plays a Critical Role in the Modulation of *Streptococcus pyogenes* Virulence

**FIGURE 7:**
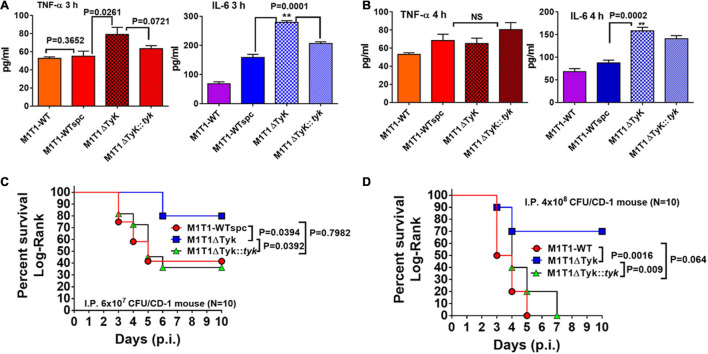
Determination SP-TyK in GAS virulence. **(A)** Quantitative analyses of the secretion of TNF-α and IL-6 using the supernatant of A549 cell lines co-cultured with various GAS strains for 3 and 4 h in CO_2_ incubator at 37°C, employing the sandwich ELISA method-based TNF-(B.D. optiEIA) and IL-6 (RandD) Detection kits. The supplied purified TNF and IL-6 as part of the kits were used as respective internal standards for the final quantification of the TNF and IL-6 per the instructions of the manufacturer. The bar diagrams represent average values ± S.D. obtained from three independent experiments with duplicate technical repeats. *p* < 0.05 indicates a significant difference. **(B)** The key role played by SP-TyK in virulence as determined by *in vivo* mouse intraperitoneal (i.p.) infection model. Percent survival of CD1 mice (10–12 mice/group) infected i.p with M1T1-WT, M1T1ΔTyK, and M1T1ΔTyK::*tyk* GAS strains using two different infection doses [**(C)** 6 × 10^7^ CFU/mouse, **(D)** 4 × 10^8^ CFU]. Sham-infected (PBS only) and GAS strain-infected mice were observed for 10 days post-infection (p.i.). All sham-infected mice survived and the mice infected with control M1T1-WT, M1T1-WTspc, and M1T1-WTspc/pDC strains (4 × 10^8^ CFU per mice, *N* = 5) died within 4–5 days (Data not shown). Percent survival was evaluated statistically by the Log-Rank test using GraphPad Prism 6 software. **p < 0.05 was treated as a significant difference.

Based on our previous report on the GAS mutant lacking SP-PTP (enrichment of Tyr-phosphorylation) showing attenuation of virulence (Kant et al., 2015), we predicted that the lack of SP-TyK (enriched Tyr-phosphatase activity) might reciprocally result in increased virulence. However, RNA-sequence-based DEGs indicated that in the absence of SP-TyK, GAS metabolic fitness and cell division processes were compromised, and the expression of many well-established virulence factors did not change. The increased transcript abundance of *speB* and *slo in* M1T1ΔTyK mutant, as revealed by RNA-seq analysis, could contribute to increased virulence. In support of this finding, the levels of secreted pro-inflammatory markers, TNF-α and IL-6, were detected from A549 human lung epithelial cell lines co-cultured with and without various GAS strains for 3 h (Figure 7A) and 4 h at 37°C (Figure 7B). The mutant strain, as compared with the control wild-type strains and the complemented strain, revealed significantly higher concentrations of TNF-α (*p* = 0.0261) and IL-6 (*p* = 0.0001) in the culture supernatants at 3 h. The relative concentration of secreted IL-6, in comparison with TNF-α, was found to be significantly higher (*p* = 0.0002) in the culture supernatants of the mutant-infected cells compared with other GAS-strains despite IL-6 concentration *per se* decreased at 4 h. These results indicated that the SP-TyK could impact potential virulence properties by influencing host inflammatory responses during GAS infection.

To further examine the global impact on *in vivo* virulence properties of GAS lacking SP-TyK, the intraperitoneal mouse septicemia infection model was employed. In the initial experiments, all mice infected with three different control GAS strains (M1T1-WT, M1T1-WTspc, and M1T1-WTspc/pDC strains, 4 × 10^8^ CFU/mouse, *N* = 5) died within the initial 5 days, while none of the sham-infected mice died during the observation period (Data not shown). Therefore, in the subsequent experiments, only the M1T1-WT, M1T1ΔTyK, and M1T1ΔTyK::*tyk* GAS strains were examined for their virulence properties by infecting CD-1 mice (*N* = 10) with two separate different doses (6 × 10^7^ CFU/mouse and 4 × 10^8^ CFU/mouse, Figures 7C,D). Control mice challenged with the lower dose of wild-type and complemented strains displayed 70% mortality within 5–6 days, and 30% of mice survived throughout the remaining observation period. The group of mutant strain-infected mice showed significantly low mortality (20%, *p* = 0.039). At a higher infecting dose, all mice of the M1T1-WT and M1T1ΔTyK::*tyk* groups died by 5–7 days, indicating that the complementation with wild-type *sp-tyk* restored virulence trait. Even at the higher dose, the mutant strain caused significantly lower mortality (30% mortality, *p* = 0.0016), as was observed in the mice challenged with the lower infecting dose of GAS. Together these results showed that SP-TyK played a crucial role in the maintenance of the GAS virulence.

### The Absence of *Streptococcus pyogenes*-Tyrosine Kinase Adversely Impacts Group A *Streptococcus* Ability to Adhere to and Invade Host Cells and Form Biofilms

**FIGURE 8:**
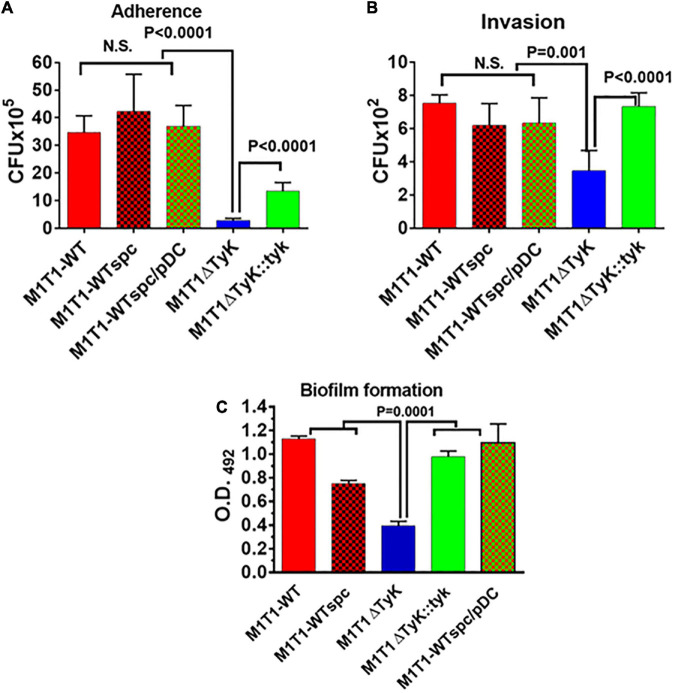
Impact of the absence of SP-TyK on the GAS ability to adhere to and invade the host cells and to form *in vitro* biofilms. **(A)** Adherence to and **(B)** Invasion of human lung epithelial cell lines (A549) by M1T1 wild-type control, M1T1ΔTyK mutant and M1T1ΔTyK::*tyk* complemented GAS strains [multiplicity of infection (MOI)-100:1::bacteria:cell] was observed after 3 h of co-culture in CO_2_ incubator at 37°C using six-well tissue culture plates. Invasion assays were carried out in separate duplicate 12-well plates, after adherence assays and penicillin/gentamicin treatment for 90 min as described in the “Materials and Methods” section. The adherent bacteria and antibiotic treatment-protected internalized bacteria in these two assays were enumerated from the respective whole cell lysates employing the plate dilution method as CFU isolated on blood agar plates. Individual bars in the graphs represent average CFUs/ml ± S.D. A value pf *p* < 0.05 was treated as a significant difference. **(C)** Polystyrene 12-well tissue culture plate-based assays of M1T1-wild-type control, M1T1ΔTyK mutant, M1T1ΔTyK::*tyk* complemented GAS strains to determine biofilm formation. Equal numbers of bacteria (∼5 × 10^6^ CFU/ml/well) were seeded in 2 ml THY broth using 12-well polystyrene tissue culture plates, incubated for 72 h, and further processed as described in the description in the “Materials and Methods” section. The washed biofilms were dried and stained with Coomassie stain and extracted using 33% (V/V) acetic acid. The abundance of individual biofilms was measured spectrophotometrically in the form of Optical density at 490 nm. Individual bars in the graphs represent the average O.D. ± S.D. obtained from four independent experiments, each with three technical replicates. Values of *p* < 0.05 were treated as significant difference and determined by unpaired Student’s *t*-test with Welch correction using GraphPad Prism 6 software.

The results described above showing *in vitro* ability of the mutant to induce inflammatory responses and at the same time *in vivo* mutant’s inability to cause GAS disease, indicated that additional mechanisms were involved in the attenuation process. In the conventional tissue-culture plate-based bacterial adherence and gentamycin-protection-based invasion assays revealed significantly fewer numbers of M1T1ΔTyK adhered to A549 cells (*p* < 0.0001) (Figure 8A). The complemented strain significantly recovered adherence capacity, yet it did not reach the wild-type control strains level. The adherence of all control strains was comparable and with no significant differences among them. Subsequently, parallel invasion assays also revealed the compromised ability of the mutant strain to invade the host cells (*p* = 0.001) (Figure 8B). The complemented strain recovered its capacity to invade the A549 cell lines similar to control wild-type and other control GAS strains. In the control wells without cells, the input of the bacterial count at the end of 3 h of incubation remained essentially the same as at starting t_0_-time point. These results thus indicated that the innate capacity of the mutant to adhere and invade the host cells was severely compromised, which was not influenced by the growth defect or the number of bacteria (Figures 8A,B).

The increased expression of SpeB and downregulated PTS and other transport system-related genes in M1T1ΔTyK mutant (Table 2) indicated that *S. pyogenes* biofilm formation might adversely affect as reported recently in a time and stage-dependent proteomic and transcriptomic analyses of *S. pyogenes* biofilm formation revealing the importance of several differentially expressed genes encoding for carbohydrate, lipid, and transport metabolism, cell division, chromosome partitioning, and cell wall biogenesis (Freiberg et al., 2016). The increased expression of *speB* in the M1T1ΔTyK mutants (Table 1) also indicated possible inhibition of adherence and *S. pyogenes* biofilms as previously reported (Kansal et al., 2000; Connolly et al., 2011; Anderson et al., 2014). The observed compromised ability of the mutant to adhere to host cells indicated that the lack of SP-TyK might adversely impact GAS biofilm formation. In polystyrene tissue culture plate-based assays, M1T1-WT and control strains showed the robust formation of biofilms after 48 h of incubation (O.D._490_ = 1.12). However, the M1T1ΔTyK mutant showed significantly decreased (*p* = 0.015) biofilms (Figure 8C). The complemented strain regained this lost function (*p* < 0.0001) and formed biofilms similar to the wild-type M1T1-WT strain. These results supported our hypothesis and indicated that SP-TyK-mediated post-translational modification-modulated functions played a critical role in GAS adherence, invasion, and biofilm formation, which together impacted its virulence.

## Discussion

Tyrosine phosphorylation, a relatively rare event in eukaryotes, is heavily relied upon by prokaryotes. The present study highlights the previously unrecognized and rarely studied post-translational tyrosine phosphorylation-mediated modifications of protein and its modulatory roles in critical cellular metabolic activities in *S. pyogenes*. In particular, we have provided the direct proof of *in vitro* and *in vivo* tyrosine phosphorylation in *S. pyogenes* by characterizing SP-TyK as a member of a novel class of bacterial kinases, UbK (ubiquitous bacterial kinase) (Nguyen et al., 2017). Furthermore, besides possessing tyrosine kinase activity on its own, SP-TyK also phosphorylates physiologically relevant targets at their Tyr/Ser/Thr residues and displays a global regulatory role. UbK enzymes were earlier identified as ATPase units of the adenosine-threonyl carbamoyltransferase complex (ATP hydrolase) (Mangat and Brown, 2008; Thiaville et al., 2015). In this work, we focused on providing information on the functions of this protein in the bacterial physiology and virulence of *S. pyogenes*.

**FIGURE 9:**
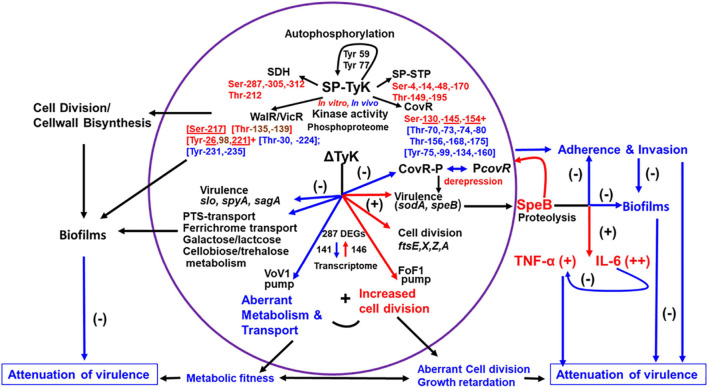
Schematic diagram showing the role of SP-TyK in GAS pathophysiology. SP-TyK, belonging to a class of ubiquitous bacterial kinase (UbK) enzymes, shows a tyrosine kinase activity during autophosphorylation targeting its Tyr59 and Tyr77 residues. However, during kinase activity on specific substrates, it shows dual specificity targeting Ser and Thr residues, as indicated and revealed based on Mass-spectrometric-based phosphoproteomic analysis. The residues in red and blue fonts represent *in vitro* and *in vivo* phosphorylated residues. The underlined residues in red fonts were detected both *in vitro* and *in vivo* conditions. The residues in brown fonts were detected only *in vitro* phosphorylation. The selection of SP-TyK substrates was based on RNA-seq-based transcriptome analysis and certain precedence described in the “Result” section. This analysis, as shown, identified a nearly equal proportion of down- (−) and upregulated (+) genes indicated with blue and red arrows, respectively. As shown, downregulated many types of transport and carbohydrate metabolism genes and some virulence genes result in compromised metabolic fitness. The upregulated cell division genes in conjunction with compromised metabolic fitness results in abnormal morphology and growth retardation. The observed differential virulence gene regulation results in attenuation of virulence properties of the mutant despite increased expression of *speB*. SpeB, being an important virulence factor, can play a dual role in regulating *S. pyogenes* virulence. As shown, SpeB, on the one hand, induces proinflammatory responses increasing TNF-α and IL-6 secretion. The sustained release of IL-6 dampens the expression of TNF-α, thus mitigating its adverse effect on the host. The increased expression of *speB* and, hence, the increased translated SpeB protein as an outcome of derepression of non-phosphorylated CovR in the absence of SP-TyK-mediated phosphorylation results in attenuation of the mutant’s virulence. This attenuation is likely due to the SpeB-mediated non-specific proteolytic activities on the bacterial surface proteins involved in adherence and invasion processes and biofilm formation and, in turn, can result in attenuation of virulence. As shown, in addition to the various functions of CovR, the functions of other proteins/regulators modulated by SP-TyK–mediated post-translational modifications can potentially contribute to *S. pyogenes* virulence. The virulence of *S. pyogenes* and its disease outcomes are multifactorial host-parasite relationships, and bacterial metabolism plays a critical regulatory role. SP-TyK, as depicted, plays an essential modulatory role in fine-tuning this regulation of pathophysiological processes that govern *S. pyogenes* pathogenesis.

Despite structural similarities among UbK enzymes, SP-TyK showed distinct differences from pneumococcal and *B. subtilis* UbK enzymes (Nguyen et al., 2017; Pelletier et al., 2019). Unlike *S. pneumoniae* UbK (Pelletier et al., 2019) and *Bacillus* YdiB (Nguyen et al., 2017), SP-TyK is not an essential enzyme for bacterial survival as the slow growth of the M1T1ΔTyK mutant reached an appreciable optical density after an extended lag period (Supplementary Figure 2). GAS SP-TyK, unlike *Bacillus* YdiB (Nguyen et al., 2017) and *S. pneumoniae* UbK (Pelletier et al., 2019), phosphorylated more efficiently in the presence of Mn^+ 2^ instead of Mg^+2^. Further, the autophosphorylation of SP-TyK was observed at two conserved Tyr residues, Tyr59 and Tyr77, while in *S. pneumoniae*, UbK has been shown to be autophosphorylated only at the conserved Tyr58 residue. In *Bacillus* YidB, this conserved residue is replaced with Phe63. The autophosphorylated YidB phosphorylates at Tyr82, Ser60, Thr42, and Thr62 residues, but a point mutation at Tyr82Ala (SP-TyK Tyr77 equivalent) does not entirely remove its kinase activity (Nguyen et al., 2017). Even though these Ser/Thr residues are conserved in *S. pneumoniae* UbK and *S. pyogenes* SP-TyK (Figure 1), they are not targeted during the autophosphorylation reaction. Thus, YdiB autophosphorylates primarily at its Ser60, Thr62, and Thr42 [Figure 1, (Nguyen et al., 2017)]. As summarized in Figure 9, the identification of four physiologically relevant substrates of SP-TyK, based on the global transcriptome analysis of M1T1ΔTyK mutant in the present study, contributes significantly to help better understand the role of SP-TyK in GAS pathogenesis. The absence of signal sequence in this protein makes this protein essentially cytoplasmic (Supplementary Figure 1E). The ability of SP-Tyk to target only Tyr residues of its own and Tyr and/or Ser/Thr residues of four identified substrates *in vitro* and/or *in vivo* conditions makes this enzyme a versatile dual-specificity tyrosine kinase.

The cell division defects observed in the SP-TyK mutant seem similar to those reported in *S. pneumoniae* (Nguyen et al., 2017), *Bacillus* (Pelletier et al., 2019), as well as *Haemophilus influenza* (Teplyakov et al., 2002). In the GAS and other bacterial genomes, acetyltransferase, and LCP (Lytr-Cps2A-Psr) proteins-encoded genes are adjacent to the *sp-tyk* gene (Supplementary Table 7). These proteins play a critical role in the last step of translocating the lipid conjugate to peptidoglycan during cell wall biosynthesis and capsule biosynthesis in certain Gram-positive bacteria (Schoenemann and Margolin, 2017). Hence SP-TyK, like other UbK enzymes, may directly or indirectly influence cell wall biosynthesis and cell division processes. The feature of altered cell division patterns of M1T1ΔTyK mutant in this study overlaps in part with that of mutants lacking SP-STK (Boel et al., 2005; Jin and Pancholi, 2006), SP-STP (Agarwal et al., 2011) or SP-PTP (Kant et al., 2015) in *S. pyogenes* and other Gram-positive bacteria (Manuse et al., 2015; Mijakovic et al., 2016). These common features indicate that these kinases target conserved substrates and target residues, and thus directly or indirectly modulating the cell division process. The mechanism by which UbK enzymes in general regulate bacterial cell division is, however, presently unknown and requires experimental verification. Thus, despite having sequence similarities, the unique genomic environment in different bacterial groups (Supplementary Table 7) indicates that these seemingly subtle yet essential differences reflect functional diversities and the unique nature of individual UbK enzymes that fulfill particular bacterial species’ physiological needs to perform unique sets of cellular activities.

The general knowledge gap in understanding the modulatory role of UbK enzymes is partly due to their recent identification, lack of information on their global regulatory functions, and possible target(s) for post-translational modifications. However, as described above, although the precise mechanism underlying the observed cell division defects in the absence of SP-TyK is presently unknown, the mutant displaying the upregulated cell-division-related genes (*ftsZ*, *ftsX*, *ftsE*, *ftsA*, and cell partitioning gene) (Table 1) indicate the recruitment and over-accumulation of the FtsZ protein at the division site to form multiple and frequent septal rings (Margolin, 2005, 2020; Schoenemann and Margolin, 2017; Figure 3). Similarly, the downregulation of lactose/galactose- (*spy_1395-spy_1401*) and *N*-acetylneuraminate metabolism (*spy_0213–spy_0218*)-related genes as well as genes encoding for several PTS systems (Table 2), transporters, permeases, and V-type ATP synthase pump (*spy_0125*–*spy_0133*) can adversely impact metabolic fitness and resulting slow bacterial growth (Supplementary Figures 1C,D). Together, this non-equilibrium may result in multiple septa within irregularly and undivided or swollen bulged cells (Figure 3).

The downregulated tagatose-6-pathway in the mutant for lactose/galactose metabolism and transport (Loughman and Caparon, 2006; Table 2) signifies inability of the mutant to metabolize non-glucose carbohydrate contributing to adversely affected metabolic fitness. The concomitant increased expression of *speB* expression is possibly due to the downregulated unique negative transcriptional regulator, *lacD.1/spy*_*1395* (aldolase), as reported earlier in the *emm*14 strain (Loughman and Caparon, 2006) and may, in part, contribute to enhancing the virulence property of the mutant. However, based on certain published reports, the secretion of SpeB in the absence of *lacD.1* could be a strain-dependent or serotype-dependent phenomenon as the deletion of the *lacD.1* gene in *emm1* (strain A20) does not affect the *speB* transcription (Chiang-Ni et al., 2016). Future studies on the role SP-TyK-mediated post-translational modifications of this regulator and corresponding modulated functions may reveal the precise mechanistic role of SP-TyK in modulating metabolic fitness and virulence.

The detection of *in vitro* and *in vivo* Tyr as well as Ser/Thr phosphorylation in WalR and CovR concur with almost fourfold binding efficiency of SP-TyK-phosphorylated CovR to *PcovR* (Figures 6, 9). These results support the observed upregulation of the *speB* expression in the absence SP-TyK as an outcome of derepression of CovR regulated genes, including *speB* (Graham et al., 2002; Gusa and Scott, 2006). To our knowledge, the tyrosine phosphorylation of WalR and CovR either *in vitro* or *in vivo* assay has not been reported for any organism. Given the precedent that many tyrosine kinases phosphorylate phosphatases and enhance their activities in eukaryotes (Vogel et al., 1993; Dohadwala et al., 1994), it is intriguing that SP-TyK also phosphorylated SP-STP, a cognate phosphatase of SP-STK (Jin and Pancholi, 2006). Whether SP-TyK alters the phosphatase activity *per se* and possible proapoptotic activity of SP-STP on eukaryotic cells (Agarwal et al., 2011, 2012) require further investigation, especially knowing the vital role of SP-STP in *S. pyogenes* virulence (Agarwal et al., 2011).

Because of their secretory nature, many bacterial tyrosine phosphatases have a direct role in perturbing host immune and cellular systems, thereby contributing to bacterial virulence or the pathogenic processes in the host (Whitmore and Lamont, 2012; Sajid et al., 2015). However, the literature on bacterial tyrosine kinase as a virulence factor is very minimal (Cozzone, 2005; Chao et al., 2014; Kohler et al., 2020). UbK enzymes, including SP-TyK, unlike the secreted *Helicobacter* CtkA tyrosine kinase (Tenguria et al., 2014), are essentially cytoplasmic (Supplementary Figure 1E) as they lack signal sequence. Hence, examining their direct roles in modulating innate host responses is either not logical or technically challenging. However, the global transcriptome analysis of M1T1ΔTyK provides the clue that the absence of SP-TyK impacts the expression of several innate bacterial virulence properties. In addition to the CovR-regulated *speB* gene along with other differentially up and downregulated virulence-related genes (Tables 1, 2), clearly indicate that SP-TyK-mediated phosphorylation modulates GAS virulence. The increased secretion of TNF-α and IL-6 from A549 cells co-cultured with the M1T1ΔTyK mutant strain shows increased expression of *speB* (Figures 7A,B) concurs with the previous report showing the ability of SpeB to induce TNF-α and its physiological impact on the host cell (Tamura et al., 2004). The observed attenuation of the mutant’s virulence properties could be partly due to sustained and significantly higher IL-6 secretion compared with TNF-α from the co-culture with the M1T1ΔTyK mutant, as the increased IL-6 suppresses TNF-α production resulting in protection against septic shock by *S. pyogenes* (Diao and Kohanawa, 2005). The downregulation of other essential virulence-related genes encoding SpyA (Hoff et al., 2011), Slo (Mozola and Caparon, 2015), SagA (Datta et al., 2005), Sic-01 (Frick et al., 2003), Mac-1 (Agniswamy et al., 2006) and other *mga*-regulated virulence genes (Hondorp et al., 2013; Table 2) may also contribute directly or indirectly to *S. pyogenes* virulence.

As such, *S. pyogenes* virulence is multifactorial (Walker et al., 2014) and dynamically regulated by the host and bacterial interaction (Casadevall and Pirofski, 2009). Although tyrosine phosphorylation plays a role in the biosynthesis of extracellular polysaccharide capsule formation, biofilm formation, and virulence (Whitmore and Lamont, 2012; Chao et al., 2014; Kohler et al., 2020), UbK enzyme-mediated tyrosine phosphorylation in such a process is presently unknown. However, several lines of evidence as summarized in Figure 9, including downregulation of carbohydrate, lipid metabolism, cell division, and cell wall biosynthesis genes in the M1T1ΔTyK mutant in association with its inability to form biofilms, support previous reports on the role of metabolism and transport in biofilm formation (Stipp et al., 2013; Fiedler et al., 2015; Freiberg et al., 2016; Young et al., 2016). Additionally, the increased expression of SpeB correlates with the reduced ability of the mutant to adhere to and invade host cells (Figures 8A,B). The reduced adherence, in turn, is likely to contribute to significantly reduced formation of biofilms (Figure 8C) as reported previously (Connolly et al., 2011). A potentially reduced biofilm-like niche in the host can result in virulence attenuation (Figures 7C,D). Although not yet explored, the lack of tyrosine phosphorylation of WalR may also adversely impact the ability of M1T1ΔTyK mutant to form biofilms through the control of WalR regulon, including cell wall hydrolase production, cell division, and wall biosynthesis-associated proteins (Dubrac et al., 2007).

In conclusion, our study revisits the long-standing unsupported presumption that Tyr-phosphorylation does not exist in *S. pyogenes* and has no relevant physiological significance. The present study resolves this knowledge gap by providing critical information on the SP-TyK enzyme as a genuine tyrosine kinase with dual specificity targeting Tyr and/or Ser and Thr residues of important virulence factors and regulators *in vitro* and *in vivo*. The present study emphasizes that SP-TyK-mediated post-translational modifications play an essential role in the differential expression of genes related to the GAS glycolytic pathway, oxidative phosphorylation, cell division, cell wall biosynthesis, PTS transport, and virulence through proteomic and genomic analyses. Together, this regulation influences *S. pyogenes* cell division, metabolic fitness, adherence to host cells, biofilm formation, host inflammatory response mediators, and ultimately bacterial virulence (Figure 9). SP-TyK, thus, can serve as a potential therapeutic target to develop novel anti-virulence therapeutic agents.

## Data Availability Statement

The datasets presented in this study can be found in online repositories. The names of the repository/repositories and accession number(s) can be found in the article/Supplementary Material.

## Ethics Statement

The animal study was reviewed and approved by the Ohio State University IACUC.

## Author Contributions

SK and VP contributed to the experimental designs, performed the biological experiments, critically analyzed the data, and wrote the manuscript. Both authors have read and approved the final manuscript.

## Conflict of Interest

The authors declare that the research was conducted in the absence of any commercial or financial relationships that could be construed as a potential conflict of interest.

## Publisher’s Note

All claims expressed in this article are solely those of the authors and do not necessarily represent those of their affiliated organizations, or those of the publisher, the editors and the reviewers. Any product that may be evaluated in this article, or claim that may be made by its manufacturer, is not guaranteed or endorsed by the publisher.

## Footnotes

1
Maestrogen.com
