# Supplementary material for: Novel Tyrosine Kinase-Mediated Phosphorylation With Dual Specificity Plays a Key Role in the Modulation of Streptococcus pyogenes Physiology and Virulence
Source: Front Microbiol. 2021 Dec 7;12:689246. doi: 10.3389/fmicb.2021.689246 (PMC8689070; doi:10.3389/fmicb.2021.689246)
Supplement: Supplementary file 1 [file Data_Sheet_1.PDF]

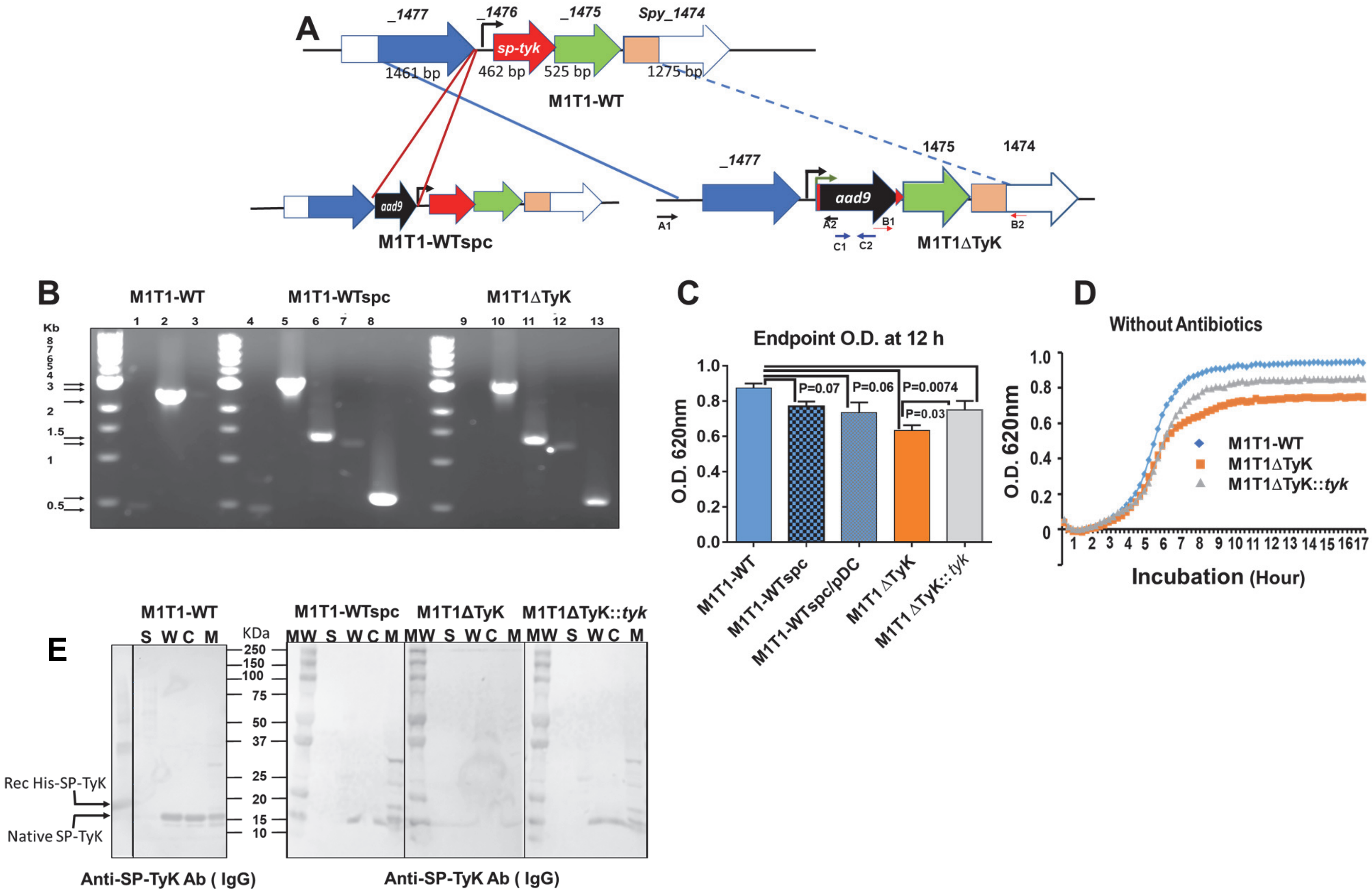

MW- Molecular weight marker; **S**- Culture supernatant; **W**- Cell wall fraction; **C**-cytoplasm; **M**-membrane particulate fraction

Fig. S1

Fig. S1. Derivation and characterization of the mutant lacking the *spy_1476* gene. (A) An allelic replacement strategy was employed to replace *spy_1476 /sp-tyk* from the wild-type *S. pyogenes* M1T1 5448 (Upper panel) strain with the *aad9* (spectinomycin resistance as a selection marker) gene using the pFW6 suicide vector. The lower left panel shows the strategy to create M1T1-WTspc control strain by introducing the *aad9* gene between the end *spy_1477* and the beginning of the Promoter region-containing intergenic region before *spy_1476*. The lower right panel shows a strategy to create the M1T1ΔTyK mutant as described in Materials and Methods. A1/A2 (Primers # 7/28), B1/B2 (Primers#27/8), and C1/C2 (Primers # 25/26) denote the location of the primers for determining the integrity of the correct insertion of the gene as determined by PCR and sequencing (See Table-S1). **(B)** Ethidium bromide-stained 1% agarose DNA Gel showing each line with PCR-amplified product of different sizes, primer pairs, and genomic DNA of the M1T1-WT M1T1-WTspc and M1T1ΔTyK mutant as a template. Lanes 1, 4, and 9 depict the presence or absence of *spy-1476/sp-tyk*-specific PCR product obtained with Primers #1/2 (Table-S1). Lanes 2, 5, 10 show PCR amplified products using flanking primers (A1/B2). Lanes 3, 8, 13 depict PCR products obtained with *aad9*-specific primers (C1/C2). Lanes 6 and 11 illustrates PCR products obtained with primers A1/A2. Lanes 7 and 12 depicts PCR product obtained with primers B1/B2. **(C)** Endpoint O.D. of M1T1-WT, M1T1-WTspc, M1T1-WTspc/pDC, M1T1ΔTyK, and M1T1ΔTyK::*tyk* measured at 12 h by growing these strains in THY media without any antibiotics. Results are based on average $O.D._{620nm}$ obtained with three independent cultures ± S.D. P-values <0.05 were treated as a significant difference and were obtained using the paired parametric *t*-test using GraphPad Prism 6. P-value <0.05 is treated as a significant difference. **(D)** Growth curves of M1T1-WT, M1T1ΔTyK, and M1T1ΔTyK::*tyk* were obtained by growing them in THY broth for a period of 16 h at 37 ^{o}C. Each data point represents an average optical density (λ620nm) ± S.D. of the four independent cultures at different time points, as indicated. **(E)** Cell fractionation and Western blot analysis of Wild-Type, wild-type control, ΔTyK mutant and ΔTyK::*tyk* complemented M1T1 GAS strains using Protein-A/G-purified anti-SP-TyK antibodies. **MW**- Molecular weight marker; **S**- Culture supernatant; **W-** Cell wall fraction; **C-**cytoplasm; **M**-membrane particulate fraction. Differential migration of Native and His-Rec SP-TyK is due to additional His-tag in the recombinant protein.
