# Supplementary material for: Novel Tyrosine Kinase-Mediated Phosphorylation With Dual Specificity Plays a Key Role in the Modulation of Streptococcus pyogenes Physiology and Virulence
Source: Front Microbiol. 2021 Dec 7;12:689246. doi: 10.3389/fmicb.2021.689246 (PMC8689070; doi:10.3389/fmicb.2021.689246)

Fig-S1 Representative SEM pictures of GAS strains

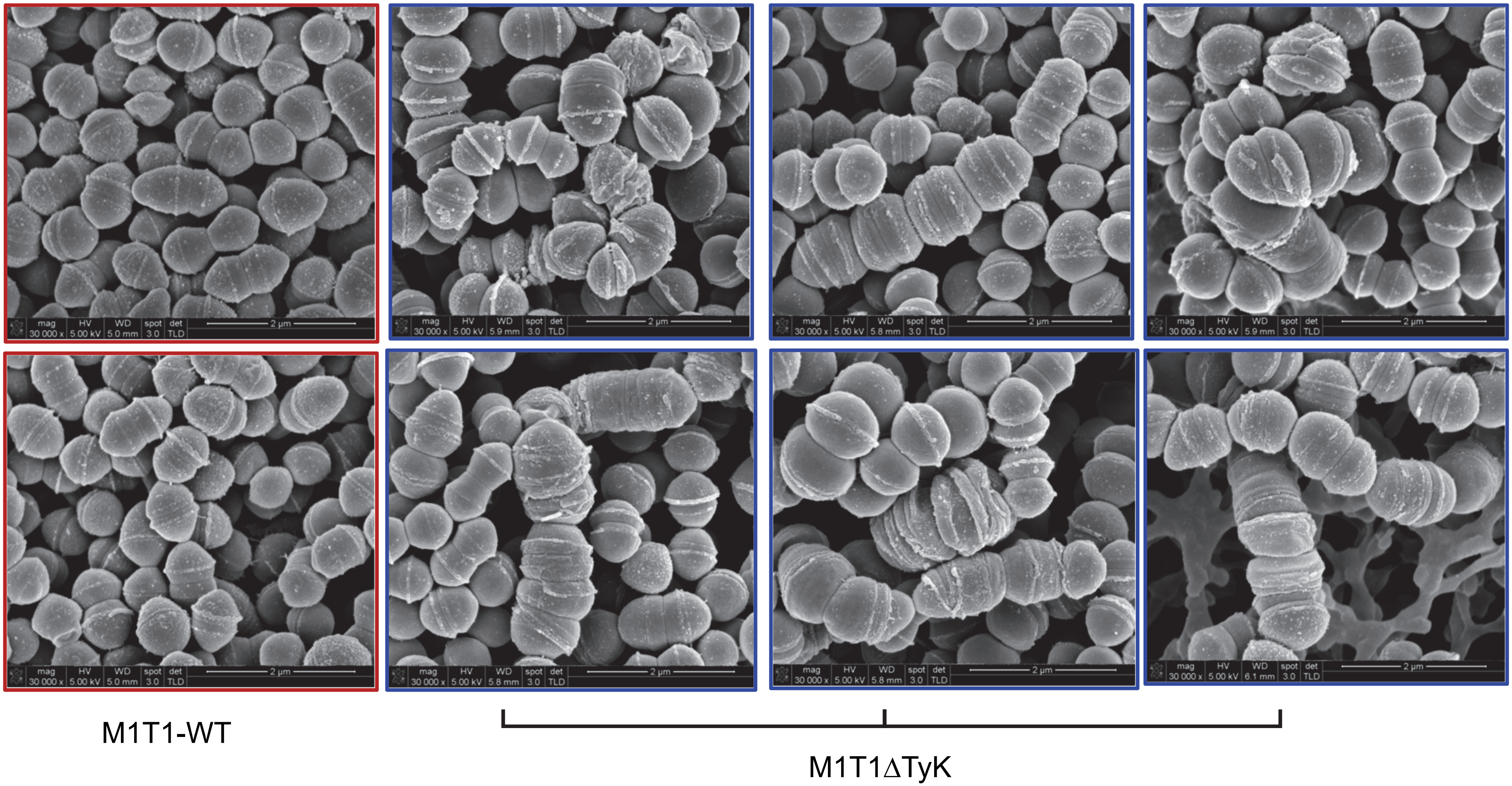

Conti….Fig-S2 Representative SEM pictures of GAS strains

M1T1ΔTyK::*tyk*

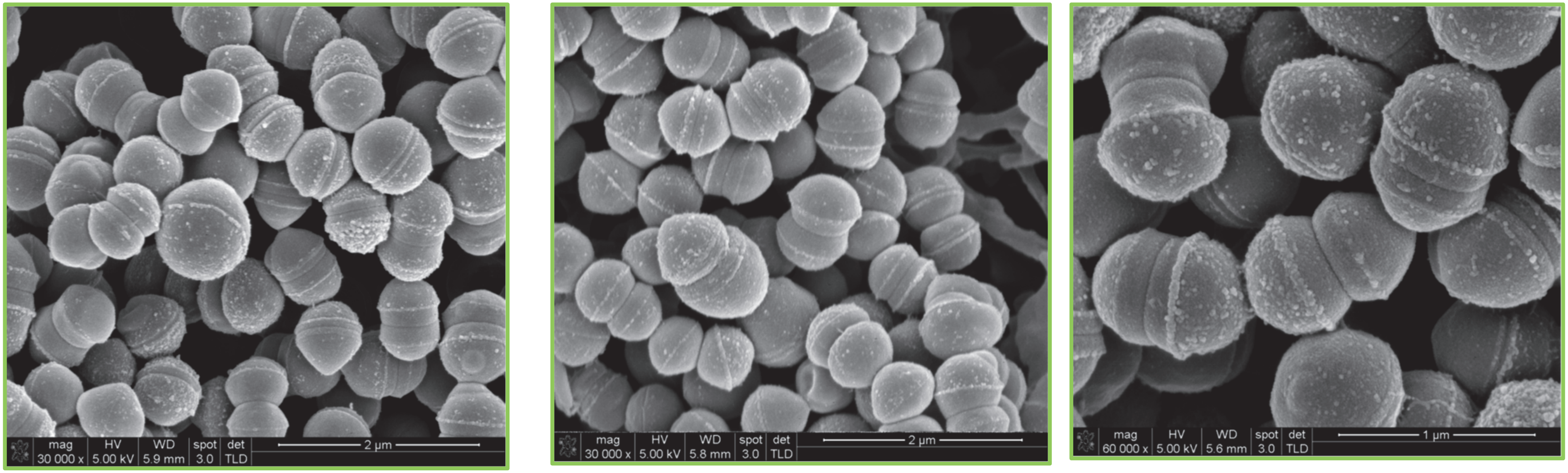

Supplement: Supplementary file 2 [file Data_Sheet_2.PDF]
