# Supplementary figures and images for: Novel Tyrosine Kinase-Mediated Phosphorylation With Dual Specificity Plays a Key Role in the Modulation of Streptococcus pyogenes Physiology and Virulence
Source: Front Microbiol. 2021 Dec 7;12:689246. doi: 10.3389/fmicb.2021.689246 (PMC8689070; doi:10.3389/fmicb.2021.689246)

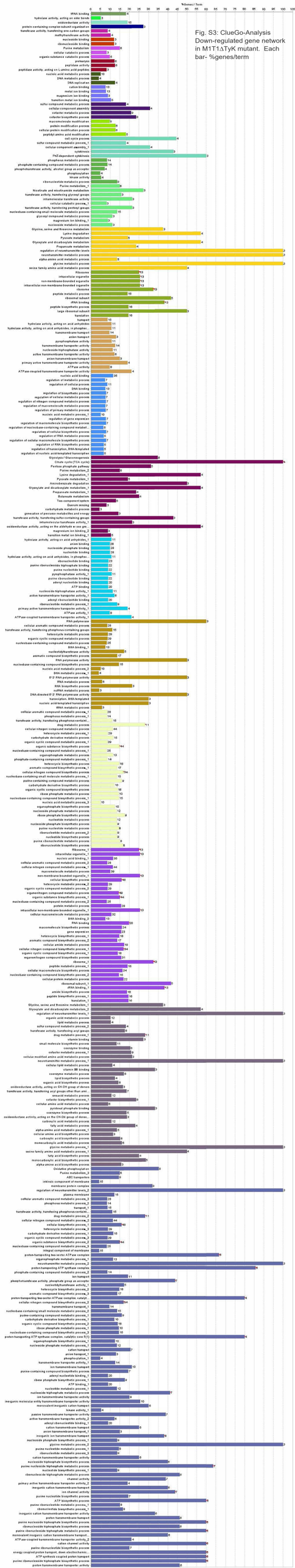

Supplement: Supplementary file 3 [file Data_Sheet_3.PDF]

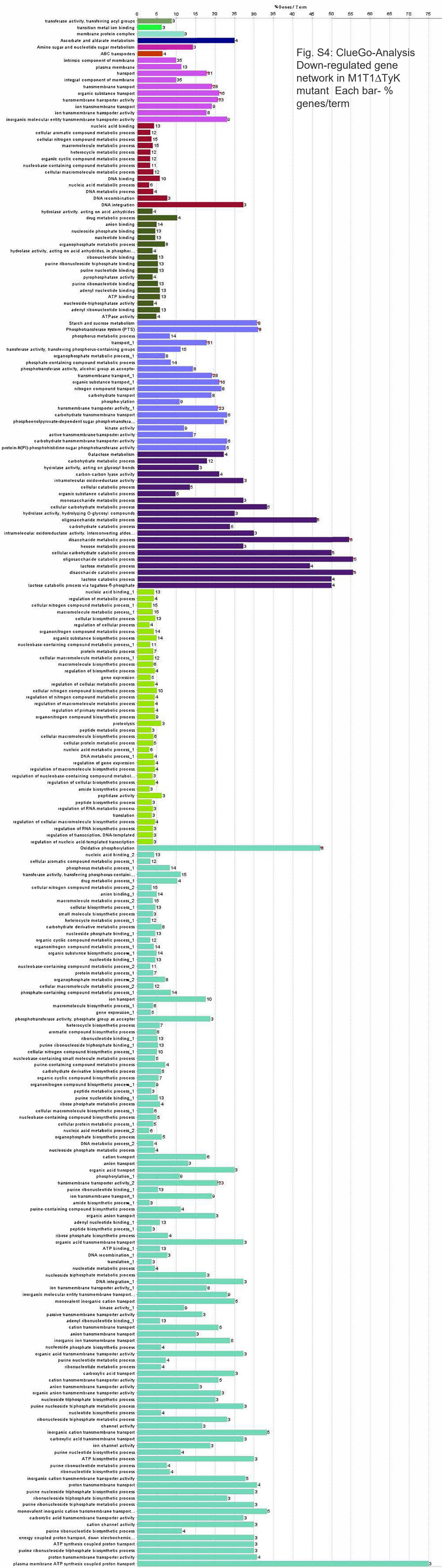

Fig. S4: ClueGo-Analysis  
Down-regulated gene  
network in M1T1ΔTyK  
mutant Each bar- %  
genes/term

Supplement: Supplementary file 7 [file Data_Sheet_7.PDF]
