# Supplementary material for: Novel Tyrosine Kinase-Mediated Phosphorylation With Dual Specificity Plays a Key Role in the Modulation of Streptococcus pyogenes Physiology and Virulence
Source: Front Microbiol. 2021 Dec 7;12:689246. doi: 10.3389/fmicb.2021.689246 (PMC8689070; doi:10.3389/fmicb.2021.689246)
Supplement: Supplementary file 4 [file Data_Sheet_4.PDF]

Supplemental Methods and results:

A. Creation of Mutants and Complemented strains and Validation of their Genetic Integrity

A.1 Mutation Strategies, Complementation and Growth Curve

S. pyogenes mutant lacking the *spy_1476* gene was derived from Type M1 M1T1 5448 strain using the suicide pFW6 (Spectinomycin^R) vector (Agarwal *et al.*, 2011, Podbielski *et al.*, 1996). Supplementary Table-S1 describes the sequences of the oligonucleotides used for different constructs. The upstream (1021 bp including the first 21nt of the Spy_*1476*) and downstream (1044 bp including the last 23 nt of the *spy_1476* gene) fragments of the *spy_1476* gene were PCR-amplified, using the primer pair # 3/4 and primer pair# 5/6 respectively. These fragments were then cloned in the BamH1/SacI and SphI/XmaI restricted MCS I and MCS II of pFW6 vector, respectively. The resulting construct, pFW6ΔTyK was subsequently used to transform M1T1 5448 electrocompetent cells. A similar strategy was used to create a parallel control with empty plasmid (M1T1-WTspc) by introducing the *aad9* gene between *spy_1477* and the start of the *spy_1476* promoter-containing intergenic region by sequential cloning of the PCR products obtained with primer pairs #29/30 and #31/32 respectively in MCS-I and MCS-II (Fig. S1). The transformants obtained on spectinomycin (500 µg/ml)-containing blood agar plates were screened for the gene replacement by PCR assay using the flanking primer pairs (Primers # 7/8,) in combination with the internal primer pairs of the amplification of the *aad9* (primers #25/26, 27/28) and *spy_1476 (*Primers *#1/2*) genes (Table-S1 and Fig. S1), as well as followed by DNA sequence, and genome sequence analysis (Novagen Corp Inc. Durham, NC).

To recover optimal expression of the *spy_1476* gene and obtain selectable resistance marker other than spectinomycin resistance, the complementation vector, pDC123 (Chloramphenicol resistance), was used (Chaffin & Rubens, 1998). The entire 622 bp region (462 bp of the sp-*tyk* ORF along with upstream 160 nucleotides containing the native promoter, P*tyk*) was PCR-amplified using a primer pair (Primers # 9/10) and cloned between XmaI/BamHI restriction sites in the pDC123 as previously described (Agarwal *et al.*, 2011) (Table-S1). The resulting plasmid pDC-*tyk* was used to complement the M1T1ΔTyK mutant GAS strain. A similar strategy was used to create M1T1-WTspc/pDC strain by transforming the M1T1-

WTspc strain with an empty pDC123 plasmid as described above. The resulting M1T1ΔTyK::*tyk* complemented strain and M1T1-WTspc/pDC were selected on chloramphenicol (5 µg/ml)-containing blood agar plates.

Overnight cultures of the control wild-type, mutant, and complemented GAS strains were washed and adjusted to O.D.=1.0 (620nm) (~5 x 10^8 CFU/ml). A fresh THY broth was inoculated with 1:1000 dilution of O.D. 1.0 culture and was further incubated for 12 h, and the endpoint O.D. was first measured in three independent cultures of each GAS strain. Subsequently, in separate experiments, optical densities of each culture were measured at every 1h in quadruplet samples for 16h, using 96-well tissue culture plates and using spectrofluorometer (PolarStar Gaalxy, BMJ) under the absorbance mode as described (Agarwal *et al.*, 2011, Jin & Pancholi, 2006, Kant *et al.*, 2015).

A.2 Cell fractionation and location of SP-TyK in GAS strains

Overnight culture of the Wild-type, Wild-type Control, mutant and the complemented GAS strains were pelleted and the 5 ml supernatant of each strains was precipitated with TCA at its 20% of final concentration. The precipitates were washed twice with 100 ethanol with 1% Na_2CO_3 and finally suspended in volume to obtain 1/50 of the original volume. Similarly, the bacterial pellets were suspended in 1/50 volume of the original culture volume in 30% (W/V) raffinose buffer (Tris/HCL pH8.0 with 5mM EDTA) and digested with phage lysin (Kant *et al*., 2015) for 90 min and centrifuged to obtain cell wall fraction in the supernatant. The pelleted protoplasts were then lysed in the hypotonic lysis buffer (10mM TRIS/HCl, pH8.0 containing 5mM$MgCl_2$, 5µg/ml DNAse,, 10 µg/ml RNase, protease and phosphatase inhibitor cocktail, and 0.1% Triton-X100). The resulting lysates were sonicated for 2 min (60% efficiency of pulse for 10 sec and 20 sec intervals). The total protoplast lysates were centrifuged (14,000 rpm 4°C for 10 min) to obtain supernatants as the cytoplasmic fraction and pellets as an unpurified membrane particulate fraction. Each fractions were suspended in the same volume and the proteins were resolved by SDS-PAGE followed by electrotransfer on PVDF membranes. Membranes were probed with Protein-A/G column-purified Rabbit anti-SP-TyK IgG antibodies (1: 1000) and corresponding alkaline phosphatase-

conjugated antibodies (1:2,500). The reactive protein bands were visualized by the chromogenic method as described (Kant *et al.*, 2015).

A.3 Validation of the Genetic Integrity of the Mutant Strains

S. pyogenes (GAS) mutant lacking SP-TyK (M1T1ΔTyK) from the Type M1 M1T1 5448 GAS strain was derived by an allelic replacement method utilizing the suicide pFW6 vector, as described previously (Agarwal *et al.*, 2011, Podbielski *et al.*, 1996). The pFW6 vector, unlike pFW5, lacks a transcription terminator at the end of the *aad9* gene (antibiotic resistance marker), avoiding potential polar effects. The design and creation of the control strain by introducing the *aad9* gene at the end of *spy_1477* and the beginning of the promoter region-containing intergenic region upstream of *spy_1476*/*sp-tyk* (M1T1-WTspc) is shown in Fig. S1A (lower left panel). The *spy_1476*/*sp-tyk* gene was replaced with *aad9* (spectinomycin-resistance/SpcR), keeping the co-transcribing (26 residues overlapping region) gene *spy_1475* intact to derive M1T1ΔTyK strain, (Fig. S1A lower right panel). The genetic integrity of the mutant and wild-type strains by PCR using various primers (Fig. S1B, Table-S1) and gene sequencing described in the Materials and methods. M1T1-WT and M1T1-WTspc, but not the M1T1ΔTyK strains, showed the presence of the *sp-tyk* gene. The mutant and M1T1-WTspc, but not the M1T1-WT, strains showed the presence of the *aad9* gene (Fig. S1B lanes 3, 8, and 13). The mutant showed the absence of the *sp-tyk* gene (Fig. S1B lanes 1, 4, and 9). Further, the flanking primers yielded expected 2609 bp, 3695 bp, and 3301 bp PCR-product from the M1T1-WT, M1T1-WTspc, and M1T1ΔTyK, respectively (Fig, S1B arrows, lanes 2, 5, 10). As described later in the RNA seq analysis (Table-S2-S4), the transcript abundance of overlapping genes *spy_1475* and spy_*1474* in the mutant did not alter when compared to the wild-type strain indicating that the employed allelic replacement strategy did not cause any polar effects. The genomic analysis of the mutant and wild-type did not reveal any changes other than the deletion of the gene *sp-tyk* and its replacement with the *aad9* gene. The mutant was subsequently complemented with the wild-type *sp-tyk* gene, including the native promoter and ribosomal binding site (RBS) (M1T1ΔTyK::*tyk*). A control wild-type strain, M1T1-WTspc complemented with pDC123 plasmid, served as a control complemented wild-type strain (M1T1-WTspc/pDC).

No significant difference was observed amongst the peak endpoint O.D. (measured after 12 h) of M1T1-WT (0.85±0.03) vs. M1T1-WTspc (0.75 ± 0.07) vs. M1T1-WTspc/pDC (0.8 ± 0.05) grown in THY broth ($P>0.05$, Fig. 3C). However, the growth of M1T1ΔTyK mutant (peak O.D.= 0.65 ± 0.03) in the absence of SP-TyK was significantly retarded ($P=0.0074$) (Fig. S1C). Hence, we obtained growth curves of M1T1-WT, M1T1ΔTyK, and M1T1ΔTyK::*tyk* for 16h (Fig. S1D). Our results revealed that the log phase growth pattern of the M1T1ΔTyK mutant strain, compared to the wild-type and complemented strains, was retarded by 2 h and 1.5 h, respectively, indicating that the *sp-tyk*-complemented GAS strain recovered the wild-type growth pattern (Fig. S1C and S1D). Nevertheless, the mutant still grew to an appreciable abundance and obtained a sustained plateau phase with no decrease in O.D. during the 16 h of the observation period. Together, these results suggested that the presence of SP-TyK influences the overall GAS growth pattern.

B. Mass-Spectrometric Analysis of SP-TyK protein and Phosphorylated Substrates

For mass spectrometry analyses, the autophosphorylation and phosphokinase reactions were carried out in cold ATP instead of radioactive ^{32}P-ATP. Gel slices of both Coomassie-stained phosphorylated and nonphosphorylated SP-TyK and other substrates (SP-STP, CovR, WalR, and SDH) were subjected to LC-MS/MS mass-spectrometry analysis. Essentially, the mass spectrometry analysis was performed at OSU Comprehensive Cancer Center (CCC) Proteomic Shared Resource (PSR).

Briefly, the stained gels slices were digested using sequence grade trypsin (Promega, Madison WI) using the Montage In-Gel digestion kit from Millipore (Bedford, MA) following manufacturers' recommended protocol 0.1M ammonium bicarbonate buffer pH 8.4. The peptides of trypsin digests were extracted with 50% acetonitrile-5% formic acid and then subjected to capillary-liquid chromatography-nanospray tandem mass spectrometry (nano-LC/MS/MS) using the Thermo Finnigan LTQ-Fusion orbitrap mass spectrometer instrument equipped with a nanospray source operated in a positive ion mode. Data were acquired in LTQ with a resolution of full scan set at 30000 to achieve high mass accuracy MS determination. Sequence information from MS/MS data was processed by converting the raw data files into merged files (.mgf) using the in-house proGram, RAW2MZXML_n_MGF_batch (merge. pl, a Perl script). The resulting mgf files were searched using the MASCOT Daemon software (MatrixScience Ver

2.2.1, Boston, MA), and the data obtained were searched against the bacterial NCBI database. The mass accuracy of the precursor ion was set to 2.0 Da. The data were acquired on an ion trap mass analyzer, and the fragment mass accuracy was set to 0.8 Da. The mass accuracy of the precursor ions was set to 1.2 Da for LTQ-Orbitrap, and the fragment mass accuracy was set to 0.8 Da. Considered modifications (variable) were methionine oxidation, carbamido methyl cysteine, and phosphorylation. Peptides with a P value less than 0.02 with a score less than 20 were filtered. Protein identification was checked manually. Proteins with a P value less than 0.05 and a Mascot score of 50 or higher with a minimum of two unique peptides from one protein having *a*, *-b,* or *–y* ion sequence tag of five residues or better were accepted. Data were also searched with MassMatrix (Xu & Freitas, 2007, Xu & Freitas, 2008, Xu & Freitas, 2009) against the protein sequence for the investigation of phosphorylation. Possible hits from MassMatrix were validated manually.

C. RNA-Seq-based Transcriptome Analysis

Briefly, the GAS strains (M1T1 WT, M1T1ΔTyK) were first treated with phage lysin as described previously (Agarwal *et al.*, 2011, Jin & Pancholi, 2006, Kant *et al.*, 2015) to obtain whole bacterial cell lysate. Total RNA from the resulting lysates was first extracted by TRIZOL™ and treated with RNase-free DNase. High quality total RNA (RIN>7.0, 260/280 and 260/230 ratios >1.8, Nanodrop, Qubit) was purified from these extracts using the RNA purification kit (Norgen, Canada) per manufacturer's instructions and as confirmed by an Agilent 2100 Bioanalyzer (Agilent Technologies, Palo Alto, CA).

The DNase I-treated high-quality total RNA samples were subjected to RNA-seq analysis in the commercial facility of Novogene Corp. Inc. (Durham, NC). rRNA was removed from the total RNA samples using the Ribo-zero kit. cDNA was then made using mRNA as a template. The double-stranded cDNAs were end-repaired, adenylated, ligated with adapter sequences, and size selected using AMPure XP beads. After agarose gel electrophoresis, the suitable fragments were selected for the PCR amplification. ABI bioanalyzer and ABI StepOnePlus real-time PCR system were used for all quality control steps required for quantification and quality of sample library. Libraries were sequenced using Illumina Hiseq

2000 using 100 bp sequencing. High-quality total RNA (20 µg) preparations obtained from three biological repeats from wild-type M1T1 and M1T1ΔTyK were subjected for RNA-seq transcriptome analysis.

C.1 Data Analysis Primary sequencing data produced by Illumina Hiseq 2000, called "raw reads," were subjected to quality control (QC) to determine if a re-sequencing step was needed. After QC, raw reads were filtered into clean reads, which were then aligned to reference sequence *Streptococcus pyogenes* MGAS5005 NC_007297.1 (Sumby *et al.*, 2005) with a popular-alignment tool Bowtie2 (Ver 2.4.2) (Langmead *et al.*, 2009, Langmead *et al.*, 2019, Langmead & Salzberg, 2012). Gene expression level was measured by transcript abundance. It was estimated based on FPKM (fragments per kilobase of transcript sequence per million), actual gene expression level as well as proportional to the gene length and sequencing depth and pair sequenced (Trapnell *et al.*, 2010). HTSEq software was used to analyze the gene expression levels in this experiment (Anders *et al.*, 2015). For biological replicates, the mean FPKM values were used. Differentially expressed genes (DEG) were determined based on fold changes (Up- or down-regulated) $\log_2 \pm 1$, and P values and corrected P values (FDR/ q-values/ adjusted P values) < 0.05. FDR value estimation was based on multiple hypothesis testing. Graphical representation of these values was presented in the form of a Volcano plot and cluster heat diagram.

C.2 ClueGo analysis

For this analysis, differentially expressed genes (DEG) from the M1T1ΔTyk mutant vs. M1T1-WT were filtered based on fold changes [(Up- or down-regulated) log2 ±1, and P values and corrected P values (FDR/ q-values/ adjusted P values) < 0.05], and that were analyzed via pathway analysis with ClueGO (Bindea *et al.*, 2009, Bindea *et al.*, 2013) version 2.5.8 in Cytoscape (Assenov *et al.*, 2008, Shannon *et al.*, 2003) version 3.8.2. Before analysis, the gene loci of DEGs of *S. pyogenes* M1T15448 (ORF annotated using MGAS5005 terminology) were converted into *S. pyogenes* M1 476 gene locus. The following *S. pyogenes* M1 476 ontologies were referenced in the pathway analysis: KEGG_11.03.2020, GO_BiologicalProcess-Custom-GOA_06.03.2020, GO_Molecular Function-Custom-GOA_06.03.2020, GO_CellularComponent-Custom-GOA_06-03-2020, and GO_ImmuneSystemProcess-Custom-GOA_06-03-2020. Only pathways that had Bonferroni step down-corrected Enrichment/Depletion (Two-sided hypergeometric test) with mid-*P* values less than or equal to 0.05 were displayed. Gene ontology (GO)

levels 3 to 8 were used. Larger nodes represent more significant terms, while node shading is proportional to the percentage of genes represented for that term. Grouping of nodes was done with a perfuse-directed layout based on a kappa score of 0.4. Group terms are based on the node with the highest percentage of genes per term compared to the cluster. Any groups sharing 50% of genes or 50% of terms were merged.

Legend:

Fig. S1. Derivation and characterization of the mutant lacking the *spy_1476* gene. (A) An allelic replacement strategy was employed to replace *spy_1476 /sp-tyk* from the wild-type *S. pyogenes* M1T1 5448 (Upper panel) strain with the *aad9* (spectinomycin resistance as a selection marker) gene using the pFW6 suicide vector. The lower left panel shows the strategy to create M1T1-WTspc control strain by introducing the *aad9* gene between the end *spy_1477* and the beginning of the Promoter region-containing intergenic region before *spy_1476*. The lower right panel shows a strategy to create the M1T1ΔTyK mutant as described in Materials and Methods. A1/A2 (Primers # 7/28), B1/B2 (Primers#27/8), and C1/C2 (Primers # 25/26) denote the location of the primers for determining the integrity of the correct insertion of the gene as determined by PCR and sequencing (See Table-S1). **(B)** Ethidium bromide-stained 1% agarose DNA Gel showing each line with PCR-amplified product of different sizes, primer pairs, and genomic DNA of the M1T1-WT M1T1-WTspc and M1T1ΔTyK mutant as a template. Lanes 1, 4, and 9 depict the presence or absence of *spy-1476/sp-tyk*-specific PCR product obtained with Primers #1/2 (Table-S1). Lanes 2, 5, 10 show PCR amplified products using flanking primers (A1/B2). Lanes 3, 8, 13 depict PCR products obtained with *aad9*-specific primers (C1/C2). Lanes 6 and 11 illustrates PCR products obtained with primers A1/A2. Lanes 7 and 12 depicts PCR product obtained with primers B1/B2. **(C)** Endpoint O.D. of M1T1-WT, M1T1-WTspc, M1T1-WTspc/pDC, M1T1ΔTyK, and M1T1ΔTyK::*tyk* measured at 12 h by growing these strains in THY media without any antibiotics. Results are based on average $O.D._{620nm}$ obtained with three independent cultures ± S.D. P-values <0.05 were treated as a significant difference and were obtained using the paired parametric *t*-test using GraphPad Prism 6. P-value <0.05 is treated as a significant difference. **(D)** Growth curves of M1T1-WT, M1T1ΔTyK, and M1T1ΔTyK::*tyk* were obtained by growing them in THY broth for a period of 16 h at 37 ^{o}C. Each data point

represents an average optical density (λ620nm) ± S.D. of the four independent cultures at different time points, as indicated. **(E)** Cell fractionation and Western blot analysis of Wild-Type, wild-type control, ΔTyK mutant and ΔTyK::*tyk* complemented M1T1 GAS strains using Protein-A/G-purified anti-SP-TyK antibodies. **MW**- Molecular weight marker; **S**- Culture supernatant; **W-** Cell wall fraction; **C-**cytoplasm; **M**-membrane particulate fraction. Differential migration of Native and His-Rec SP-TyK is due to additional His-tag in the recombinant protein.
