# Supplementary material for: Novel Tyrosine Kinase-Mediated Phosphorylation With Dual Specificity Plays a Key Role in the Modulation of Streptococcus pyogenes Physiology and Virulence
Source: Front Microbiol. 2021 Dec 7;12:689246. doi: 10.3389/fmicb.2021.689246 (PMC8689070; doi:10.3389/fmicb.2021.689246)
Supplement: Supplementary file 5 [file Data_Sheet_5.PDF]

Table-S1. Primers and their nucleotide sequences used in the present study

SR #	Primer	Primer sequence	Purp ose
1	HisSP-TyK-nde-F	AAA*CATATG*TTTTATAGTGAAAATGAA	Forward primer for recombinant 6Xhis Sp-TyK Using pET14B NdeI site
2	HisSP-TyK-BamHI-R	AAA*GGATCC*TCAATAACGAGACTTTGC	Reverse primer for recombinant 6Xhis Sp-TyK Using pET14B BamHI site
3	Up-SP-TyK-BamHI-F	AAA*GGATCC*TGCTATTTTTGGTTTACT	Forward and reverse primer upstream of the Spy1476 for cloning into MCSI of pFW6 plasmid
4	Up-SP-TyK-Sac-R	AAA*GAGCTC*AGCAACATTATATCAGAA	
5	Dn-SP-TyK-sphI-F	AAA*GCATGC*TTCTGGAGATAATGTCAC	Forward and reverse primer downstream of the Spy1476 for cloning into MCSII of pFW6 plasmid
6	Dn-SP-TyK-Xma-R	AAA*CCCGGG*TCACAAGATCAATAAGGC	
7	Scrn-SP-TyK-F	GACGCCAATGGTGGAGTTGTA	Forward and reverse primer in the flanking region of the genome respective to upstream and down steam region of *Spy1476* used for the cloning in pFW6 plasmid
8	Scrn-SP-TyK-R	GCAACAGTAGCTTGATATTCAGGTTCG	
9	Comp-Prom SP-Tyk-Xma- F	AAA*CCCGGG*CTTATAACCAAATAATTT	Forward and reverse primers encompassing the entire region encompassing the spy1476 and it upstream promoter.
10	Comp-SP-TyK-Bam-R	AAA*GGATCC*TCAATAACGAGACTTTGC	
11	PTP-1F	GGAAT*CTCGAG*ATGAAAAAGTATGTTTTGT CTG	Forward primer for recombinant 6Xhis SP-PTP using pET14B Xho1 site
12	PTP-2R	CAAGT*GGATCC*TCAATACTCTTTACTCATTA AAC	Reverse primer for recombinant 6x His SP-PTP using pET14B BamH1site
13	CovR-F	CCGGC*CATATG*ACAAAGAAAATTTTAATTAT TG	Forward and reverse primers for recombinant 6XHis SP-

Table-S1. Primers and their nucleotide sequences used in the present study

14	CovR-R	CCGC*GGATCC*TTATTTCTCACGAATAACGT	WalR and SP-CovR using pET14B WalR and pET14BCovR. Reference:Agarwal et al (2011) J Biol Chem. 286:41368-41380.
15	WalR-F	TGAT*CTCGAGA*TGAAAAAAATACTTATTGTGGA	
16	WalR-R	CATA*GGATCC*CTAGTCATAAGATTTCATGTA	
17	SP-STP-nde-F	CTAG*CATATG*AAGATTTCATTAAAAACAGACATTGGGC	Forward and reverse primers for recombinant 6Xhis SP-STP. Reference: Jin,H. and Pancholi,V. (2006). J. Mol. Biol.357, 1351–1372.
18	.SP-STP-Bam R	CGC*GGATCC*TCATTCGACGTCCTCACTTTCGTTATG	
19	SDH-Nde-F	CTAG*CATATG*ATGGTAGTTAAAGTTGGTATTAACGG	Forward and reverse primers for recombinant 6XHis SDH
20	SDH-Bam-R	CGC*GGATCC*TTATTTAGCAATTTTTGCGAAGTACTC	
21	SEN-Nde-F	CTAG*CATATG*TCAATAATAACTGATGTGTATGCTCG	Forward and reverse primers for recombinant 6XHis SEN
22	SEN-Bam-R	CGC*GGATCC*TTAAGTTATAGAATGATTTGATCC	
23	Comp-His-WalR-KpnI-F	GTAC*GGTACC*GAGGTATAGCATGAAAAAAATACTTATTGTGGA	Forward and reverse primer to clone *his-walR* along with its ribosomal binding site in pDC123 between KpnI and Bam H1 sites.
24	Comp-His-WalR-BamHI-R	CATA*GGATCC*CTAGTCATAAGATTTCATGTA	
25	*aad9*-F	TTGGATCAGGAGTTGAGAGTGGAC	Internal Forward and reverse primers integrity for the validation of the mutant
26	*aad9*-R	TCCAAGATAACTACGAACTGCTAACAA	
27	*aad9-Dn-F*	CTCCATTAGAACATAGGGAGAGAA	
28	*aad9-Up-R*	CAAACATGTAAGTACCAATAAGGTTAT	
29	1477-BamHI-F	AAA*GGATCC*TGCTATTTTTGGTTTACT	PCR fragment to be inserted into the MCS-I of pFW6
30	*1477*-SacI-*R*	AAA*GAGCTC*TTATAAGATAGTTAATATG	
31	P*tyk-PstI-F-*	AAA*CTGCAC*CCAAATAATTTACTTATTTTG	PCR fragment to be inserted in to MCS-II of pFW6 to create a control WT-type strain with empty plasmid and the *aad9*
32	*1476-Dn-R-*	AAA*CCCGGG*TCACAAGATCAATAAGGC	

Table-S1.   Primers and their nucleotide sequences used in the present study

			gene is thus inserted between the end of *Spy_1477* and starting of the promoter of Spy1476
33	P*covR*-F	AACTGAATATTAAAGATGTCT	166 bp PCR product to be used for EMSA for the binding of CovR and TyK-phosphorylated CovR-P_{ser}
34	P*covR*-R	TTATACCAACCCTTATCCTCT	
35	P*ntpI*-F	TAAACATCTTTTTGACAGGAC	464 bp PCR product to be used for EMSA for the binding with WalR and Tyk-phosphorylated WalR-$P_{Tyr/Ser}$
36	P*ntpI*-R	CACATATCAACTCCT TATCTT	
