# Supplementary material for: Novel Tyrosine Kinase-Mediated Phosphorylation With Dual Specificity Plays a Key Role in the Modulation of Streptococcus pyogenes Physiology and Virulence
Source: Front Microbiol. 2021 Dec 7;12:689246. doi: 10.3389/fmicb.2021.689246 (PMC8689070; doi:10.3389/fmicb.2021.689246)
Supplement: Supplementary file 6 [file Data_Sheet_6.PDF]

Table- S2: Unfiltered differentially regualted genes in M1T1ΔTyK mutant vs. M1T1-WT

Gene_id	Locus	Start	Stop	Length	Protein nar	readcount_	readcount_	log2FoldCh	pval	padj	Linear fold	significant
Spy0001	dnaA	202	1557	451	chromoson	3746.471	4995.097	-0.41498	0.25356	0.48496	-1.33328	FALSE
Spy0002	dnaN	1712	2848	378	DNA polym	6863.529	9470.505	-0.46449	0.21162	0.43346	-1.37983	FALSE
Spy0003	-	2923	3120	65	hypothetica	251.2707	247.6009	0.021226	0.90264	0.96409	1.014822	FALSE
Spy0004	ychF	3450	4565	371	GTP-depen	4017.973	6490.232	-0.6918	0.052888	0.18133	-1.6153	FALSE
Spy0005	pth	4635	5204	189	peptidyl-tR	339.9078	349.716	-0.04104	0.92747	0.98183	-1.02886	FALSE
Spy0006	trcF	5207	8710	1167	transcriptic	3379.807	2698.284	0.3249	0.3702	0.59458	1.252578	FALSE
Spy0007	-	8872	9144	90	heat shock	161.4633	129.2329	0.32123	0.58207	0.76846	1.249395	FALSE
Spy0008	divIC	9131	9502	123	cell divisior	663.6648	423.0554	0.64961	0.080059	0.23477	1.568744	FALSE
Spy0009	-	9499	9624	41	hypothetica	NA	NA	NA	NA	NA	NA	Na
Spy0010	-	9637	10923	428	beta-lactan	2938.348	2059.477	0.51273	0.12611	0.31295	1.426747	FALSE
Spy0011	tilS	10920	12206	428	tRNA(Ile)-ly	2467.597	1970.798	0.32433	0.33379	0.55602	1.252083	FALSE
Spy0012	-	12211	12753	180	hypoxanthi	2764.467	2088.984	0.4042	0.25068	0.4831	1.323355	FALSE
Spy0013	ftsH	12775	14754	659	cell divisior	27833.12	23366.8	0.25234	0.46533	0.68354	1.191138	FALSE
Spy0014	-	15079	16470	463	amino acid	2141.552	5024.201	-1.2302	0.000637	0.008131	-2.346	TRUE
Spy0015	-	16811	16945	44	hypothetica	NA	NA	NA	NA	NA	NA	Na
Spy0016	-	30106	30249	47	transposas	NA	NA	NA	NA	NA	NA	Na
Spy0017	-	128461	128856	131	LysR family	NA	NA	NA	NA	NA	NA	Na
Spy0017	sibA	31134	32330	398	secreted pr	26255.96	34916.41	-0.41126	0.51682	0.71971	-1.32985	FALSE
Spy0018	-	128952	129371	139	LysR family	NA	NA	NA	NA	NA	NA	Na
Spy0018	prsA.2	32583	33545	320	ribose-pho	8896.623	8840.955	0.009056	0.92991	0.98271	1.006297	FALSE
Spy0019	recO	33731	34486	251	DNA repair	347.034	245.8856	0.49709	0.24718	0.48015	1.411364	FALSE
Spy0020	plsX	34589	35596	335	glycerol-3-p	2341.842	1717.033	0.44773	0.16576	0.36738	1.363893	FALSE
Spy0021	acpP.2	35589	35831	80	acyl carrier	23.51888	23.71951	-0.01226	1	1	-1.00853	FALSE
Spy0022	-	35952	36686	244	phosphorib	107.7216	149.0243	-0.46824	0.34712	0.56989	-1.38342	FALSE
Spy0023	-	36762	40535	1257	phosphorib	1280.826	2159.335	-0.75351	0.028033	0.11844	-1.68589	FALSE
Spy0024	purF	40696	42150	484	amidophos	565.0215	991.0213	-0.81061	0.026548	0.11395	-1.75395	FALSE
Spy0025	purM	42178	43200	340	phosphorib	387.6892	631.7909	-0.70455	0.061272	0.19993	-1.62964	FALSE
Spy0026	purN	43368	43922	184	phosphorib	269.4723	470.664	-0.80456	0.040768	0.1516	-1.74661	FALSE
Spy0027	purH	44106	45653	515	bifunctiona	1098.495	1910.18	-0.79818	0.024765	0.10899	-1.73891	FALSE
Spy0028	-	45711	46835	374	autolysin	56.0372	71.13314	-0.34414	0.58644	0.77288	-1.26939	FALSE
Spy0029	purD	46998	48353	451	phosphorib	770.4759	1069.381	-0.47295	0.17178	0.37479	-1.38794	FALSE
Spy0030	purE	48511	49122	203	phosphorib	249.3047	332.5812	-0.4158	0.29817	0.52855	-1.33404	FALSE
Spy0031	purK	49106	50182	358	phosphorib	701.7615	1018.776	-0.53778	0.12542	0.31208	-1.45174	FALSE
Spy0032	-	50209	51852	547	hypothetica	1191.123	1731.504	-0.5397	0.14269	0.338	-1.45367	FALSE
Spy0033	purB	51870	53162	430	adenylosuc	2358.997	3337.593	-0.50063	0.16717	0.36959	-1.41483	FALSE
Spy0034	-	53294	54205	303	transcriptic	201.641	444.4681	-1.1403	0.009448	0.055886	-2.20427	FALSE

Spy0035	ruvB	54431	55429	332	Holliday jur	6322.969	7245.031	-0.19639	0.59318	0.77839	-1.14583	FALSE
Spy0036	-	55567	56004	145	protein tyr	1191.049	1124.982	0.08233	0.83475	0.92615	1.058727	FALSE
Spy0037	-	56027	56428	133	hypothetica	1186.383	940.2197	0.3355	0.33842	0.55964	1.261815	FALSE
Spy0038	-	56425	58200	591	acyltransfe	9388.7	10184.33	-0.11735	0.75761	0.87249	-1.08474	FALSE
Spy0039	adh2	58509	61151	880	bifunctiona	24213.18	37412.53	-0.62773	0.26058	0.49152	-1.54513	FALSE
Spy0040	adhA	61403	62419	338	alcohol deh	9073.527	9554.995	-0.07459	0.9744	0.99967	-1.05306	FALSE
Spy0041	-	62807	63778	323	Na+ driven	2435.242	2815.905	-0.20953	0.49414	0.70195	-1.15631	FALSE
Spy0042	-	63783	64100	105	Na+ driven	NA	NA	NA	NA	NA	NA	Na
Spy0043	rpsJ	64301	64609	102	30S ribosor	10140.18	15668.22	-0.62776	0.066892	0.21183	-1.54516	FALSE
Spy0044	rplC	64825	65451	208	50S ribosor	15081.71	11927.61	0.3385	0.32945	0.55129	1.264441	FALSE
Spy0045	rplD	65475	66098	207	50S ribosor	13667.26	9047.53	0.59513	0.10014	0.27261	1.510609	FALSE
Spy0046	rplW	66098	66394	98	50S ribosor	2698.7	1685.708	0.67891	0.062617	0.20262	1.60093	FALSE
Spy0047	rplB	66412	67245	277	50S ribosor	30495.87	15002.93	1.0234	0.004412	0.033252	2.032704	TRUE
Spy0048	rpsS	67384	67662	92	30S ribosor	9114.265	3736.451	1.2865	0.000313	0.005092	2.439355	TRUE
Spy0049	rplV	67678	68022	114	50S ribosor	4172.216	1666.566	1.3239	0.000332	0.005217	2.503419	TRUE
Spy0050	rpsC	68035	68688	217	30S ribosor	23509.5	9612.993	1.2902	0.000282	0.004786	2.44562	TRUE
Spy0051	rplP	68692	69105	137	50S ribosor	11591.79	5358.257	1.1133	0.002387	0.021446	2.163399	TRUE
Spy0052	rpmC	69115	69321	68	50S ribosor	1008.804	504.3775	1.0001	0.012016	0.066616	2.000139	FALSE
Spy0053	rpsQ	69347	69607	86	30S ribosor	4117.253	1909.091	1.1088	0.002943	0.02532	2.156662	TRUE
Spy0054	rplN	69632	70000	122	50S ribosor	13725.91	6270.17	1.1303	0.001487	0.01524	2.189043	TRUE
Spy0055	rplX	70079	70384	101	50S ribosor	9707.891	4678.561	1.0531	0.00369	0.02901	2.074984	TRUE
Spy0056	rplE	70408	70950	180	50S ribosor	26731.75	10708.66	1.3198	0.0002	0.003806	2.496315	TRUE
Spy0057	rpsN	70966	71151	61	30S ribosor	7000.443	3127.43	1.1625	0.001053	0.011931	2.23845	TRUE
Spy0058	rpsH	71302	71700	132	30S ribosor	26739.86	11687.42	1.194	0.000692	0.008475	2.287862	TRUE
Spy0059	rplF	71903	72439	178	50S ribosor	31366.46	13290.6	1.2388	0.000385	0.005846	2.360021	TRUE
Spy0060	rplR	72544	72900	118	50S ribosor	13519.14	7205.457	0.90784	0.010536	0.061008	1.876234	FALSE
Spy0061	rpsE	72919	73413	164	30S ribosor	17948.06	9469.82	0.92242	0.007705	0.048627	1.895292	TRUE
Spy0062	rpmD	73428	73610	60	50S ribosor	6657.192	3835.747	0.79541	0.019806	0.093109	1.735571	FALSE
Spy0063	rplO	73824	74264	146	50S ribosor	17897.57	11249.56	0.6699	0.058895	0.19532	1.590963	FALSE
Spy0064	secY	74281	75585	434	preprotein	66293.25	50791.59	0.38427	0.25082	0.4831	1.305199	FALSE
Spy0065	adk	75735	76373	212	adenylate k	3834.186	1830.118	1.067	0.00236	0.021419	2.095072	TRUE
Spy0066	infA	76491	76709	72	translation	9070.007	5193.783	0.80432	0.013816	0.07478	1.746322	FALSE
Spy0067	rpmJ	76735	76851	38	50S ribosor	29.45234	15.6337	0.91372	0.3886	0.60729	1.883897	FALSE
Spy0068	rpsM	76869	77234	121	30S ribosor	6037.923	3185.77	0.92241	0.00787	0.049326	1.895279	TRUE
Spy0069	rpsK	77252	77635	127	30S ribosor	11669.02	6227.342	0.906	0.008082	0.049748	1.873843	TRUE
Spy0070	rpoA	77681	78619	312	DNA-direct	33538.76	17903.98	0.90555	0.007394	0.048204	1.873259	TRUE
Spy0071	rplQ	78634	79020	128	50S ribosor	15886.19	9421.196	0.75379	0.026206	0.113	1.686217	FALSE
Spy0072	-	79618	79752	44	hypothetica	NA	NA	NA	NA	NA	NA	Na

Spy0073	-	86264	86449	61	hypothetica	123.7969	98.39657	0.3313	0.58811	0.77405	1.258147	FALSE
Spy0074	-	87087	87224	45	4-diphosph	NA	NA	NA	NA	NA	NA	Na
Spy0075	-	87235	87393	52	4-diphosph	NA	NA	NA	NA	NA	NA	Na
Spy0076	-	87499	87660	53	4-diphosph	NA	NA	NA	NA	NA	NA	Na
Spy0077	adcR	87770	88213	147	MarR famil	933.426	736.44	0.34197	0.35255	0.57656	1.267486	FALSE
Spy0078	adcC	88217	88936	239	high-affinit	1365.756	1169.574	0.22372	0.5625	0.7522	1.167741	FALSE
Spy0079	adcB	88929	89744	271	high-affinit	2204.41	2487.04	-0.17404	0.6098	0.79387	-1.12821	FALSE
Spy0080	-	89784	90167	127	bis(5\'-nucl	67.15583	163.3572	-1.2824	0.019788	0.093109	-2.43243	FALSE
Spy0081	tyrS	90218	91474	418	tyrosyl-tRN	1774.707	3325.488	-0.90599	0.015807	0.079648	-1.87383	FALSE
Spy0082	pbp1b	91566	93878	770	multimodu	9490.984	7941.788	0.25709	0.45723	0.67583	1.195066	FALSE
Spy0083	rpoB	94142	97708	1188	DNA-direct	41481.14	16468.14	1.3328	0.000167	0.003507	2.518911	TRUE
Spy0084	rpoC	97799	101440	1213	DNA-direct	78787.15	36521.08	1.1092	0.001424	0.015017	2.15726	TRUE
Spy0085	-	101592	101957	121	DNA bindin	2184.58	685.1	1.673	7.47E-06	0.00034	3.18877	TRUE
Spy0086	comYA	102050	102988	312	competenc	44.311	60.13922	-0.44064	0.63798	0.81583	-1.35721	FALSE
Spy0087	comYB	102867	103958	363	competenc	34.56546	56.18619	-0.70088	0.42034	0.63709	-1.6255	FALSE
Spy0088	comYC	103960	104286	108	competenc	0.696267	2.626812	-1.9156	0.72211	0.85775	-3.77271	FALSE
Spy0089	-	104360	104689	109	competenc	NA	NA	NA	NA	NA	NA	Na
Spy0090	-	104646	104930	94	hypothetica	0	1.913082	#NAME?	0.6686	0.82936	NA	FALSE
Spy0091	comYD	104923	105357	144	competenc	4.954397	11.70671	-1.2406	0.50872	0.71103	-2.36297	FALSE
Spy0092	-	105341	105667	108	competenc	5.371368	8.084801	-0.58992	0.73132	0.85953	-1.50516	FALSE
Spy0093	-	105765	106718	317	adenine-sp	2360.998	1078.596	1.1302	0.001389	0.014818	2.188891	TRUE
Spy0094	ackA	106777	107973	398	acetate kin	4884.356	2987.985	0.709	0.038223	0.14585	1.634671	FALSE
Spy0095	-	108160	108468	102	hypothetica	35.19494	92.45358	-1.3934	0.030562	0.12622	-2.62697	FALSE
Spy0096	proC	108551	109321	256	pyrroline-5	4821.748	3861.011	0.32058	0.37946	0.60098	1.248833	FALSE
Spy0097	pepA	109369	110436	355	glutamyl ar	3963.616	3093.831	0.35742	0.32499	0.54887	1.281133	FALSE
Spy0098	-	110546	110710	54	hypothetica	394.6749	721.0111	-0.86936	0.069492	0.21593	-1.82685	FALSE
Spy0099	-	110892	111185	97	hypothetica	286.3768	350.1964	-0.29025	0.72972	0.85871	-1.22285	FALSE
Spy0100	-	111182	111499	105	thioredoxir	162.1882	169.5253	-0.06383	0.97247	0.99931	-1.04524	FALSE
Spy0101	-	111517	112143	208	tRNA-bindi	2056.606	2009.307	0.033567	0.84523	0.93436	1.02354	FALSE
Spy0102	-	112295	112690	131	single-strar	23.88163	50.37073	-1.0767	0.16985	0.37204	-2.10921	FALSE
Spy0103	-	112950	113591	213	deoxyaden	3310.273	3054.021	0.11624	0.78204	0.88985	1.083906	FALSE
Spy0104	-	113611	114588	325	tRNA-dihyd	3190.793	3372.196	-0.07977	0.74293	0.86481	-1.05685	FALSE
Spy0105	hslO	114575	115447	290	heat shock	2004.871	2112.385	-0.07536	0.77125	0.88331	-1.05363	FALSE
Spy0106	rofA	115594	117087	497	transcriptic	26851.03	24953.83	0.10572	0.70839	0.85091	1.076031	FALSE
Spy0107	-	117335	119623	762	fibronectin	44612.28	41143.64	0.11677	0.76824	0.88186	1.084305	FALSE
Spy0108	-	119616	120173	185	signal pepti	4015.788	1935.754	1.0528	0.020965	0.097057	2.074552	FALSE
Spy0109	-	120206	121228	340	fibronectin	15231.05	10503.2	0.53619	0.14794	0.34596	1.450138	FALSE
Spy0110	eftLSL.B	121238	121951	237	hypothetica	10185.79	8210.529	0.31101	0.33007	0.55183	1.240576	FALSE

Spy0111	-	121973	122620	215	hypothetica	9815.345	8901.888	0.14093	0.6396	0.81583	1.102616	FALSE
Spy0112	-	122807	124129	440	transposase	238.5358	462.5198	-0.95531	0.02364	0.10626	-1.939	FALSE
Spy0113	-	124370	124720	116	transposase	1.896902	29.3809	-3.9532	0.004743	0.035025	-15.4893	TRUE
Spy0114	-	125024	125779	251	sortase	157.3588	378.4107	-1.2659	0.29478	0.52646	-2.40477	FALSE
Spy0115	-	125973	126638	221	hypothetica	16.33114	282.5548	-4.1128	3.34E-05	0.000999	-17.3012	TRUE
Spy0116	atoE	126987	128393	468	short-chain	33.88572	73.22298	-1.1116	0.49576	0.7022	-2.16085	FALSE
Spy0119	-	129492	130676	394	acetyl-CoA	33.36892	63.53082	-0.92895	0.21603	0.4383	-1.90389	FALSE
Spy0120	atoD.2	130688	131347	219	acetate Co/	15.89078	34.73637	-1.1283	0.2289	0.4558	-2.18601	FALSE
Spy0121	-	131350	131997	215	acetyl-CoA:	24.44417	48.97001	-1.0024	0.21459	0.43586	-2.00333	FALSE
Spy0122	-	132119	132799	226	DNA-bindir	616.5197	1060.618	-0.78269	0.043801	0.15987	-1.72034	FALSE
Spy0123	-	132973	133338	121	translation	206.5768	656.1411	-1.6673	0.017246	0.084632	-3.1762	FALSE
Spy0124	sloR	133375	134394	339	transcriptic	899.9337	2892.837	-1.6846	5.24E-06	0.000273	-3.21451	TRUE
Spy0125	-	134849	135169	106	hypothetica	74.76288	332.2723	-2.152	0.00049	0.006881	-4.44443	TRUE
Spy0126	ntpI	135159	137180	673	V-type ATP	401.2813	2273.741	-2.5024	0.001363	0.014628	-5.66627	TRUE
Spy0127	ntpK	137182	137661	159	V-type ATP	84.78071	464.123	-2.4527	0.000648	0.008203	-5.4744	TRUE
Spy0128	ntpE	137729	138313	194	V-type sodi	112.2967	767.2457	-2.7724	0.000534	0.007268	-6.83244	TRUE
Spy0129	ntpC	138329	139327	332	V-type ATP	213.1269	1627.026	-2.9325	0.00459	0.034141	-7.63432	TRUE
Spy0130	ntpF	139324	139644	106	V-type ATP	83.97805	509.2421	-2.6003	0.000635	0.008131	-6.06413	TRUE
Spy0131	ntpA	139845	141620	591	V-type ATP	658.8528	3655.079	-2.4719	0.00258	0.022735	-5.54774	TRUE
Spy0132	ntpB	141621	143036	471	V-type ATP	466.7573	2636.163	-2.4977	0.004101	0.031426	-5.64784	TRUE
Spy0133	ntpD	143081	143707	208	V-type ATP	203.9623	1365.942	-2.7435	0.003057	0.026053	-6.69693	TRUE
Spy0134	-	143827	145089	420	tellurite res	4086.514	4412.177	-0.11062	0.69877	0.8459	-1.07969	FALSE
Spy0135	-	145102	145980	292	hypothetica	1153.811	906.3725	0.34823	0.33619	0.55797	1.272998	FALSE
Spy0136	purA	146418	147710	430	adenylosuc	11342.69	14691.19	-0.37319	0.27143	0.50365	-1.29521	FALSE
Spy0137	-	148037	149080	347	nucleoside-	9100.194	11605.39	-0.35083	0.32797	0.55129	-1.27529	FALSE
Spy0138	nusG	149253	149792	179	transcriptic	1865.429	4117.726	-1.1423	0.000921	0.010774	-2.20733	TRUE
Spy0139	nga	150154	151518	454	NAD glycoh	239.7354	491.5945	-1.036	0.054502	0.18547	-2.05053	FALSE
Spy0140	-	151523	152008	161	hypothetica	65.0761	157.8995	-1.2788	0.018248	0.088288	-2.42637	FALSE
Spy0141	slo	152032	153747	571	streptolysir	497.654	1380.708	-1.4722	0.007291	0.04787	-2.77445	TRUE
Spy0142	-	154002	154433	143	hypothetica	24.12658	107.7292	-2.1587	0.001231	0.013521	-4.46512	TRUE
Spy0143	-	154619	154855	78	hypothetica	6.680452	71.51179	-3.4202	0.000408	0.006051	-10.7049	TRUE
Spy0144	-	155265	155432	55	hypothetica	1.113237	34.38028	-4.9487	0.000698	0.008492	-30.8821	TRUE
Spy0145	-	155607	155894	95	hypothetica	2.939843	12.80303	-2.1227	0.26682	0.49887	-4.35508	FALSE
Spy0146	metB	156335	157525	396	cystathioni	7139.719	7519.689	-0.07481	0.87794	0.94867	-1.05322	FALSE
Spy0147	leuS	157736	160237	833	leucyl-tRNA	18008.96	17305.57	0.057479	0.90579	0.96504	1.040646	FALSE
Spy0148	ulaA	160544	161977	477	PTS system	211.3537	317.8131	-0.58852	0.18442	0.3943	-1.5037	FALSE
Spy0149	-	162048	162326	92	PTS system	6.104757	37.00771	-2.5998	0.021122	0.097536	-6.06203	FALSE
Spy0150	-	162449	162934	161	PTS system	14.39887	81.07803	-2.4934	0.001458	0.015194	-5.63103	TRUE

Spy0151	ulaD	163025	163687	220	3-keto-L-gu	32.0974	111.6872	-1.7989	0.005739	0.040109	-3.47955	TRUE
Spy0152	-	163692	164555	287	L-xylulose 5	31.12359	143.0004	-2.1999	0.000336	0.005236	-4.59447	TRUE
Spy0153	araD	164557	165261	234	L-ribulose-5	44.03449	218.8993	-2.3136	2.12E-05	0.000738	-4.97122	TRUE
Spy0154	-	165319	165465	48	hypothetica	1.772381	11.55346	-2.7046	0.20101	0.41855	-6.51877	FALSE
Spy0155	-	165586	167232	548	BigG family	3040.574	3333.742	-0.1328	0.67321	0.832	-1.09642	FALSE
Spy0156	-	167485	168576	363	L-ascorbate	1949.435	2330.421	-0.25753	0.48898	0.69953	-1.19543	FALSE
Spy0157	opuAA	169064	170260	398	glycine beta	10664.34	29370.1	-1.4616	0.001195	0.013213	-2.75414	TRUE
Spy0158	opuABC	170276	172003	575	glycine beta	26690.33	72719.87	-1.446	0.001164	0.012946	-2.72452	TRUE
Spy0159	polA	172134	174776	880	DNA polym	12350.7	27317.53	-1.1452	0.12797	0.31447	-2.21177	FALSE
Spy0160	-	174963	175418	151	CoA binding	1528.611	2902.088	-0.92487	0.11482	0.30091	-1.89851	FALSE
Spy0161	perR	175470	175937	155	ferric uptak	3269.298	5441.571	-0.73504	0.045825	0.16389	-1.66444	FALSE
Spy0162	vlg	176094	176393	99	trans-acting	2057.559	3519.275	-0.77434	0.12537	0.31208	-1.71041	FALSE
Spy0163	-	176615	177949	444	3\'-phosph	9366.896	16382.02	-0.80647	0.059837	0.19736	-1.74893	FALSE
Spy0164	-	177942	178472	176	parB-like nu	4585.92	8948.917	-0.9645	0.004881	0.03547	-1.95139	TRUE
Spy0165	-	178519	178761	80	transposase	NA	NA	NA	NA	NA	NA	Na
Spy0166	-	178801	179016	71	transposase	4.599688	4.361195	0.076813	1	1	1.054686	FALSE
Spy0167	-	179056	179625	189	transposase	0	0	NA	NA	NA	NA	NA
Spy0168	-	179658	179873	71	transposase	0	0	NA	NA	NA	NA	NA
Spy0169	-	180029	181339	436	malonate p	359.9821	306.844	0.23042	0.58181	0.76846	1.173176	FALSE
Spy0170	nadC	181563	182435	290	nicotinate-(	51.74195	322.8073	-2.6413	0.045933	0.16396	-6.23894	FALSE
Spy0171	-	182735	183541	268	transposase	8.410594	8.212619	0.034365	1	1	1.024106	FALSE
Spy0173	-	184163	185026	287	hypothetica	2516.612	4304.617	-0.7744	0.15	0.34724	-1.71048	FALSE
Spy0174	-	185063	185221	52	hypothetica	NA	NA	NA	NA	NA	NA	Na
Spy0175	tgt	185245	186387	380	queuine tR	6614.148	9639.063	-0.54334	0.11405	0.30018	-1.45734	FALSE
Spy0176	-	186604	186915	103	hypothetica	71.17179	521.0295	-2.872	0.021828	0.10004	-7.32079	FALSE
Spy0177	-	186919	187458	179	bioY protei	257.7594	1887.412	-2.8723	0.006215	0.042455	-7.32232	TRUE
Spy0178	-	187598	188377	259	metal-depe	1606.427	1720.456	-0.09894	0.7465	0.86725	-1.07098	FALSE
Spy0179	-	188377	188892	171	tRNA-speci	1657.439	1566.533	0.081381	0.85804	0.93942	1.05803	FALSE
Spy0180	-	189506	190726	406	S-layer prot	309.1228	401.5142	-0.37727	0.38777	0.60659	-1.29888	FALSE
Spy0181	-	190780	190887	35	hypothetica	NA	NA	NA	NA	NA	NA	Na
Spy0182	speG	191138	191842	234	exotoxin ty	1198.341	2258.757	-0.91449	0.011016	0.063186	-1.8849	FALSE
Spy0183	-	191927	192046	39	hypothetica	NA	NA	NA	NA	NA	NA	Na
Spy0185	pgi	192298	193647	449	glucose-6-p	11176.93	7947.687	0.49192	0.1281	0.31447	1.406315	FALSE
Spy0186	-	193996	195504	502	transcriptio	167.0779	254.1499	-0.60516	0.2356	0.46611	-1.52115	FALSE
Spy0187	-	196059	196763	234	transposase	NA	NA	NA	NA	NA	NA	Na
Spy0188	-	196666	197181	171	transposase	NA	NA	NA	NA	NA	NA	Na
Spy0189	-	197165	197290	41	hypothetica	49.09352	37.18382	0.40086	0.67203	0.83161	1.320295	FALSE
Spy0190	-	197371	197478	35	hypothetica	NA	NA	NA	NA	NA	NA	Na

Spy0191	-	197506	198177	223	rhomboid f	1220.358	1388.69	-0.18642	0.66373	0.82698	-1.13794	FALSE
Spy0192	hasC.2	198276	199175	299	UTP-glucos	8481.017	8415.493	0.011189	0.87007	0.94509	1.007786	FALSE
Spy0193	-	199208	199390	60	glycerol-3-p	NA	NA	NA	NA	NA	NA	Na
Spy0194	gpsA	199408	200223	271	glycerol-3-p	7469.337	7339.389	0.02532	0.88228	0.95223	1.017705	FALSE
Spy0195	-	200521	200970	149	MarR famil	963.0807	1424.42	-0.56465	0.23625	0.46637	-1.47903	FALSE
Spy0196	-	200963	202669	568	multidrug r	4470.178	6999.648	-0.64695	0.37027	0.59458	-1.56585	FALSE
Spy0197	-	202672	204456	594	multidrug r	7830.24	12304.51	-0.65206	0.26053	0.49152	-1.57141	FALSE
Spy0198	-	204574	205341	255	hypothetica	686.3653	1334.511	-0.95926	0.083863	0.24103	-1.94431	FALSE
Spy0199	dut	205451	205897	148	deoxyuridir	862.7758	1218.986	-0.49862	0.20069	0.41835	-1.41286	FALSE
Spy0200	radA	205978	207339	453	DNA repair	2452.695	3388.659	-0.46635	0.17969	0.38774	-1.38161	FALSE
Spy0201	-	207528	208025	165	carbonic ar	1698.36	1729.638	-0.02633	0.9761	0.99967	-1.01842	FALSE
Spy0202	-	208156	208866	236	hypothetica	5705.179	9741.325	-0.77185	0.11633	0.30267	-1.70746	FALSE
Spy0203	gltX	209048	210493	481	glutamyl-tR	15306.08	13388.33	0.19313	0.54268	0.73663	1.143241	FALSE
Spy0204	fasB	210888	212234	448	sensory tra	2915.402	2451.531	0.25001	0.47303	0.68695	1.189215	FALSE
Spy0205	fasC	212231	213514	427	sensory tra	2433.814	1874.58	0.37665	0.32039	0.54758	1.298324	FALSE
Spy0206	fasA	213518	214258	246	response re	2299.077	1470.685	0.64457	0.075005	0.22613	1.563273	FALSE
Spy0207	rnpA	214798	215157	119	ribonucleas	408.2669	326.3027	0.3233	0.40646	0.62301	1.251189	FALSE
Spy0208	-	215141	215950	269	hypothetica	4060.7	2701.306	0.58807	0.080589	0.23519	1.503234	FALSE
Spy0209	-	215962	216876	304	jag protein	9710.95	6648.142	0.54666	0.09878	0.27032	1.4607	FALSE
Spy0210	-	216961	217083	40	hypothetica	NA	NA	NA	NA	NA	NA	Na
Spy0211	rpmH	217191	217325	44	50S ribosor	901.012	2559.356	-1.5062	2.93E-05	0.000922	-2.84061	TRUE
Spy0212	-	217599	218303	234	N-acetylma	702.5104	1135.42	-0.69263	0.074427	0.22476	-1.61623	FALSE
Spy0213	-	218352	219671	439	N-acetylne	781.7747	2236.805	-1.5166	0.000128	0.002885	-2.86116	TRUE
Spy0214	-	219774	220661	295	N-acetylne	189.07	739.5853	-1.9678	1.12E-05	0.000474	-3.91171	TRUE
Spy0215	-	220674	221504	276	N-acetylne	183.2137	784.3603	-2.098	4.64E-07	3.98E-05	-4.28115	TRUE
Spy0216	-	221661	222323	220	hypothetica	119.0719	580.9971	-2.2867	2.28E-07	2.60E-05	-4.87939	TRUE
Spy0217	nanH	222335	223249	304	N-acetylne	191.7364	867.8203	-2.1783	2.27E-06	0.000138	-4.5262	TRUE
Spy0218	-	223271	224209	312	N-acetylma	217.4005	1015.769	-2.2241	2.49E-07	2.64E-05	-4.67219	TRUE
Spy0219	-	224320	225150	276	RpiR family	1265.524	1207.379	0.067856	0.81482	0.91292	1.048158	FALSE
Spy0220	tatD	225402	226226	274	sec-indepe	1315.788	1466.905	-0.15685	0.68766	0.84238	-1.11485	FALSE
Spy0221	-	226198	226788	196	ribonucleas	1346.937	1415.676	-0.07181	0.89467	0.95824	-1.05103	FALSE
Spy0222	ksgA	226902	227774	290	dimethylad	3235.891	3474.454	-0.10262	0.80791	0.90853	-1.07372	FALSE
Spy0223	-	228198	229070	290	ribosome-a	2863.384	2288.995	0.32301	0.36332	0.58802	1.250938	FALSE
Spy0224	rpe	229080	229742	220	ribulose-ph	2308.538	1907.038	0.27565	0.48587	0.69672	1.210539	FALSE
Spy0225	-	229735	230367	210	thiamin pyr	2068.619	1384.642	0.57915	0.1222	0.30888	1.493969	FALSE
Spy0226	-	230369	231640	423	rmuC famil	7260.157	5026.866	0.53034	0.14564	0.3419	1.44427	FALSE
Spy0227	cbf	231630	232568	312	CMP-bindir	5952.547	4666.972	0.35102	0.32062	0.54758	1.275462	FALSE
Spy0228	purR	232835	233674	279	pur operon	4404.793	2218.64	0.9894	0.004605	0.034141	1.985359	TRUE

Spy0229	prgA	233665	236286	873	surface exc	28417.42	16940.33	0.74631	0.032897	0.13248	1.677497	FALSE
Spy0230	rpsL	236494	236907	137	30S riboson	7736.222	9745.855	-0.33316	0.34085	0.56264	-1.25977	FALSE
Spy0231	rpsG	236928	237398	156	30S riboson	14547.31	18243.9	-0.32666	0.33487	0.5572	-1.25411	FALSE
Spy0232	fus	237765	239843	692	elongation	99887.41	80201.66	0.31667	0.36908	0.59458	1.245452	FALSE
Spy0233	plr	240191	241201	336	glyceraldeh	170262.1	92897.33	0.87405	0.0074	0.048204	1.832801	TRUE
Spy0234	-	241427	241543	38	hypothetica	NA	NA	NA	NA	NA	NA	Na
Spy0235	-	241685	242425	246	amino acid	2612.282	1895.458	0.46276	0.15662	0.35488	1.378176	FALSE
Spy0236	-	242418	243986	522	amino acid	4325.599	2844.729	0.60461	0.071129	0.21915	1.520568	FALSE
Spy0237	-	244184	246082	632	hypothetica	2936.05	3468.733	-0.24053	0.41742	0.63402	-1.18143	FALSE
Spy0238	uppP	246148	246987	279	undecapren	1420.998	1889.739	-0.41128	0.23765	0.46811	-1.32987	FALSE
Spy0239	mecA	247133	247894	253	adaptor pro	1805.998	2045.286	-0.17951	0.64215	0.81627	-1.1325	FALSE
Spy0240	-	248045	249070	341	undecapren	3084.988	3576.635	-0.21334	0.54168	0.73641	-1.15937	FALSE
Spy0241	rgpG	249040	249165	41	hypothetica	NA	NA	NA	NA	NA	NA	Na
Spy0242	-	249192	249962	256	ABC transp	4188.044	1686.365	1.3124	0.00021	0.003951	2.483543	TRUE
Spy0243	-	250057	251319	420	ABC transp	9125.842	4096.341	1.1556	0.001053	0.011931	2.22777	TRUE
Spy0244	nifS3	251350	252576	408	cysteine de	9538.024	4810.408	0.98753	0.004186	0.031946	1.982787	TRUE
Spy0245	nifU	252563	253042	159	iscU protei	2138.703	1073.592	0.99429	0.005572	0.039333	1.9921	TRUE
Spy0246	-	253035	254453	472	ABC transp	10129.28	6464.98	0.64781	0.057535	0.1922	1.566788	FALSE
Spy0247	-	254605	255786	393	D-alanyl-D-	3314.818	4352.141	-0.3928	0.28992	0.51997	-1.31294	FALSE
Spy0248	dacA2	255954	257186	410	D-alanyl-D-	583.2466	727.867	-0.31957	0.46782	0.68594	-1.24796	FALSE
Spy0249	oppA	257517	259487	656	oligopeptid	15876.26	8288.011	0.93777	0.008289	0.050568	1.915565	FALSE
Spy0250	oppB	259540	261054	504	oligopeptid	7036.082	4873.265	0.52988	0.16194	0.36243	1.443809	FALSE
Spy0251	oppC	261054	261980	308	oligopeptid	4194.027	2941.051	0.512	0.28298	0.51462	1.426026	FALSE
Spy0252	oppD	261989	263059	356	oligopeptid	5875.017	4354.996	0.43192	0.29581	0.52646	1.349028	FALSE
Spy0253	oppF	263052	263975	307	oligopeptid	4867.819	4206.763	0.21056	0.6394	0.81583	1.157137	FALSE
Spy0254	-	264216	264362	48	transposase	NA	NA	NA	NA	NA	NA	Na
Spy0255	-	264737	264871	44	hypothetica	NA	NA	NA	NA	NA	NA	Na
Spy0256	comX1.1	270293	270844	183	competenc	4.624825	7.166556	-0.63188	0.74296	0.86481	-1.54958	FALSE
Spy0257	-	271455	272159	234	transposase	NA	NA	NA	NA	NA	NA	Na
Spy0258	-	272062	272577	171	transposase	NA	NA	NA	NA	NA	NA	Na
Spy0259	-	272561	272665	34	hypothetica	NA	NA	NA	NA	NA	NA	Na
Spy0260	-	272683	273267	194	lipase	1256.881	1088.991	0.20686	0.48636	0.69688	1.154173	FALSE
Spy0261	-	273267	274385	372	GTP-bindin	4310.439	2641.283	0.7066	0.032444	0.13209	1.631954	FALSE
Spy0262	-	274410	274718	102	RNA-bindin	874.8412	519.1899	0.75276	0.037402	0.14341	1.685013	FALSE
Spy0263	nadD	274787	275419	210	nicotinic ac	2510.582	1537.423	0.70751	0.037001	0.14269	1.632983	FALSE
Spy0264	-	275416	276009	197	HAD superf	1665.662	1127.638	0.56279	0.095953	0.26639	1.477123	FALSE
Spy0265	-	276009	276386	125	iojap super	467.7805	409.1945	0.19304	0.64668	0.8174	1.14317	FALSE
Spy0266	-	276428	277177	249	methyltran	1122.844	731.5731	0.61808	0.096236	0.26677	1.534831	FALSE

Spy0267	-	277431	278537	368	hypothetica	2154.126	1363.983	0.65928	0.058583	0.19464	1.579294	FALSE
Spy0268	-	278874	279029	51	hypothetica	76.73564	51.62212	0.57191	0.38987	0.60781	1.48649	FALSE
Spy0269	-	279019	279735	238	hypothetica	6212.99	6236.769	-0.00551	0.90483	0.96459	-1.00383	FALSE
Spy0270	-	279938	280780	280	ABC transp	11801.6	2680.996	2.1381	1.61E-09	3.68E-07	4.40182	TRUE
Spy0271	-	281109	281954	281	ABC transp	1732.779	965.9589	0.84305	0.025654	0.11168	1.793838	FALSE
Spy0272	-	282204	283268	354	ABC transp	2754.83	1744.326	0.65929	0.073695	0.22353	1.579305	FALSE
Spy0273	-	283269	283961	230	ABC transp	1104.604	826.0722	0.41919	0.28151	0.51387	1.337177	FALSE
Spy0274	braB	284015	285385	456	branched-c	3457.309	3832.882	-0.14878	0.71482	0.85433	-1.10863	FALSE
Spy0275	-	285619	286833	404	serine/thre	4558.287	5033.802	-0.14316	0.71508	0.85433	-1.10432	FALSE
Spy0276	-	286888	287562	224	potassium	3003.071	3239.329	-0.10926	0.85003	0.93683	-1.07867	FALSE
Spy0277	-	287572	288963	463	potassium	4531.6	4396.556	0.043647	0.79789	0.90115	1.030716	FALSE
Spy0278	gidB	289033	289746	237	16S rRNA m	1027.989	1258.099	-0.29142	0.55258	0.74549	-1.22384	FALSE
Spy0279	lemA	289896	290453	185	hypothetica	7234.644	3595.048	1.0089	0.002735	0.023868	2.012376	TRUE
Spy0280	-	290500	291396	298	heat shock	17587.36	11022.5	0.67409	0.040312	0.15067	1.59559	FALSE
Spy0281	-	291630	292163	177	hypothetica	4578.553	1736.78	1.3985	0.000111	0.002628	2.636273	TRUE
Spy0282	covR	292430	293116	228	response re	31037.32	52793.2	-0.76635	0.091601	0.25744	-1.70096	FALSE
Spy0283	covS	293259	294623	454	transmemb	4764.866	4182.898	0.18793	0.7224	0.85775	1.139128	FALSE
Spy0284	nrdR	294838	295332	164	NrdR family	675.1335	495.3991	0.44658	0.31069	0.53971	1.362806	FALSE
Spy0285	dnaB	295316	296491	391	replicative	2674.921	1721.646	0.63571	0.076802	0.22927	1.553702	FALSE
Spy0286	dnaI	296492	297394	300	primosoma	3834.322	2748.018	0.48058	0.19019	0.40212	1.395305	FALSE
Spy0287	engA	297457	298767	436	GTP-bindin	11687.27	8732.624	0.42045	0.25781	0.48783	1.338345	FALSE
Spy0288	snf	298974	302072	1032	SWF/SNF fa	6849.394	4918.513	0.47775	0.38149	0.60194	1.39257	FALSE
Spy0289	-	302315	302917	200	hypothetica	6084.114	7703.052	-0.34038	0.32869	0.55129	-1.26609	FALSE
Spy0290	murC	302957	304285	442	UDP-N-acet	8015.947	6416.748	0.32103	0.4068	0.62301	1.249222	FALSE
Spy0291	-	304331	304813	160	acetyltrans	1786.617	1292.92	0.4666	0.25551	0.48648	1.381849	FALSE
Spy0292	-	304931	306499	522	aminodeox	10233.06	8646.105	0.24311	0.48429	0.69554	1.183541	FALSE
Spy0293	greA	306524	307054	176	transcriptic	2415.541	2747.371	-0.18571	0.616	0.79849	-1.13738	FALSE
Spy0294	-	307269	307412	47	transposase	NA	NA	NA	NA	NA	NA	Na
Spy0295	-	307677	308600	307	OxaA-like p	11386.59	10623.11	0.10013	0.69737	0.84545	1.07187	FALSE
Spy0296	-	308682	308960	92	acylphosph	1017.233	820.3639	0.31031	0.3519	0.57638	1.239974	FALSE
Spy0297	-	309115	309630	171	transposase	NA	NA	NA	NA	NA	NA	Na
Spy0298	-	309533	310237	234	transposase	NA	NA	NA	NA	NA	NA	Na
Spy0299	-	310535	311272	245	23S rRNA m	1299.963	1491.977	-0.19875	0.61638	0.79849	-1.1477	FALSE
Spy0300	-	311311	311811	166	HAD superf	6088.742	2579.189	1.2392	0.000258	0.004568	2.360676	TRUE
Spy0301	-	311826	312515	229	hypothetica	20859.23	9007.928	1.2114	0.000265	0.004641	2.315622	TRUE
Spy0302	-	312693	312935	80	hypothetica	2458.627	2284.094	0.10623	0.69968	0.8463	1.076412	FALSE
Spy0303	glr	313113	313907	264	glutamate r	2724.437	1275.218	1.0952	0.002728	0.023868	2.136427	TRUE
Spy0304	-	313904	314890	328	deoxyribon	4256.245	1954.249	1.123	0.002254	0.020766	2.177994	TRUE

Spy0305	-	314869	315390	173	phosphoes	1433.303	724.2684	0.98475	0.011067	0.063278	1.97897	FALSE
Spy0306	-	315387	315848	153	hypothetica	1205.719	657.528	0.87477	0.02758	0.11699	1.833716	FALSE
Spy0307	xerD	315845	316591	248	site-specifi	2373.579	1190.247	0.9958	0.007296	0.04787	1.994186	TRUE
Spy0308	scpA	316591	317292	233	segregation	2802.557	1282.947	1.1273	0.002374	0.021435	2.184495	TRUE
Spy0309	scpB	317289	317840	183	segregation	3701.538	1835.527	1.0119	0.006756	0.04535	2.016565	TRUE
Spy0310	-	317939	318685	248	ribosomal l	4834.194	2702.254	0.83911	0.029782	0.12374	1.788946	FALSE
Spy0311	-	318682	318945	87	hypothetica	757.5394	797.3477	-0.07389	0.84635	0.93504	-1.05255	FALSE
Spy0312	-	319123	319671	182	23S rRNA m	1710.381	2843.706	-0.73345	0.042223	0.15622	-1.66261	FALSE
Spy0313	-	319982	320545	187	riboflavin t	1386.761	2027.166	-0.54775	0.49051	0.70006	-1.4618	FALSE
Spy0314	-	320547	321200	217	phosphatid	2895.944	3006.159	-0.05389	0.81113	0.91102	-1.03806	FALSE
Spy0315	-	321493	322413	306	Fe-S oxidor	2990.441	2655.917	0.17115	0.84288	0.9332	1.125956	FALSE
Spy0316	-	322452	323006	184	SAM-deper	1682.729	1639.868	0.037224	0.95563	0.99094	1.026137	FALSE
Spy0317	hlyX	323139	324473	444	hemolysin	6212.714	5900.577	0.074368	0.93151	0.98326	1.0529	FALSE
Spy0318	pflC	324479	325342	287	pyruvate fo	3207.331	4261.922	-0.41013	0.26928	0.50068	-1.32881	FALSE
Spy0319	ppaC	325473	326408	311	manganese	8917.434	13474.07	-0.59549	0.14132	0.33562	-1.51099	FALSE
Spy0320	-	326484	327137	217	hypothetica	3485.785	5808.884	-0.73678	0.045565	0.16328	-1.66645	FALSE
Spy0321	fhuG	327182	328108	308	ferrichrome	358.1173	892.8024	-1.3179	0.000862	0.010212	-2.49303	TRUE
Spy0322	fhuB	328180	329232	350	ferrichrome	314.1725	690.1944	-1.1354	0.004871	0.03547	-2.19679	TRUE
Spy0323	fhuD	329222	330154	310	ferrichrome	277.9466	579.1254	-1.0591	0.007735	0.048648	-2.08363	TRUE
Spy0324	fhuA	330180	330962	260	ferrichrome	143.1952	320.8738	-1.164	0.011777	0.065895	-2.24078	FALSE
Spy0325	murE	331208	332653	481	UDP-N-acet	4884.975	3839.44	0.34746	0.2879	0.51984	1.272319	FALSE
Spy0326	-	332741	334375	544	export prot	6031.701	4499.51	0.4228	0.18847	0.40019	1.340527	FALSE
Spy0327	upp	334543	335172	209	uracil phos	1548.511	936.5346	0.72548	0.048864	0.17273	1.653451	FALSE
Spy0328	clpP	335396	335986	196	ATP-depen	12956.49	9265.519	0.48373	0.1755	0.38108	1.398354	FALSE
Spy0329	-	336478	336753	91	hypothetica	1843.724	2498.877	-0.43866	0.2232	0.44836	-1.35534	FALSE
Spy0330	tmk	337002	337637	211	thymidylate	1136.067	1784.275	-0.65129	0.15308	0.35166	-1.57057	FALSE
Spy0331	dnaX	337655	338530	291	DNA polym	1654.909	2637.608	-0.67248	0.15729	0.35526	-1.59381	FALSE
Spy0332	-	338549	338788	79	tpl protein	642.9822	1148.391	-0.83676	0.038516	0.14636	-1.78603	FALSE
Spy0333	-	338877	339038	53	signal pept	NA	NA	NA	NA	NA	NA	Na
Spy0334	-	339193	339516	107	DNA replica	1184.476	1729.992	-0.54652	0.1146	0.30091	-1.46056	FALSE
Spy0335	-	339521	340384	287	corrin/porp	3224.841	4674.789	-0.53567	0.10768	0.28757	-1.44962	FALSE
Spy0336	-	340411	340803	130	hypothetica	1532.333	2339.439	-0.61043	0.087537	0.24948	-1.52671	FALSE
Spy0337	cutC	340850	341479	209	copper hon	1471.019	1520.869	-0.04808	0.96588	0.99479	-1.03389	FALSE
Spy0338	-	341778	342134	118	arsenate re	699.7428	1037.761	-0.56858	0.14108	0.33551	-1.48306	FALSE
Spy0339	exoA	342208	343119	303	exodeoxyri	2695.914	1872.006	0.52619	0.16759	0.36962	1.440121	FALSE
Spy0340	lctO	343269	344450	393	L-lactate ox	7886.846	13236.26	-0.74698	0.037332	0.14341	-1.67828	FALSE
Spy0341	-	344713	349656	1647	lactocepin	10431.56	35548.43	-1.7688	1.20E-05	0.000499	-3.4077	TRUE
Spy0343	-	350145	350324	59	hypothetica	805.6321	1509.896	-0.90626	0.013208	0.07255	-1.87418	FALSE

Spy0344	-	350427	351134	235	permease	403.5062	1654.84	-2.036	0.005598	0.039333	-4.10107	TRUE
Spy0345	metG	351377	353374	665	methionyl-t	10289.39	19406.57	-0.91539	0.006763	0.04535	-1.88608	TRUE
Spy0346	-	353705	353872	55	hypothetica	NA	NA	NA	NA	NA	NA	Na
Spy0347	nrdF	353869	354882	337	ribonucleot	585.0785	604.8662	-0.04799	0.91762	0.97554	-1.03382	FALSE
Spy0348	nrdI	354886	355374	162	ribonucleot	112.5389	132.9042	-0.23996	0.67994	0.83685	-1.18096	FALSE
Spy0349	nrdE.1	355341	357521	726	ribonucleot	2074.991	2082.255	-0.00504	0.98806	1	-1.0035	FALSE
Spy0350	-	357500	357718	72	hypothetica	1.392534	2.397593	-0.78387	0.8766	0.94808	-1.72174	FALSE
Spy0351	spyA	357724	358476	250	C3 family A	628.0785	1308.578	-1.059	0.003111	0.026272	-2.08349	TRUE
Spy0352	-	358935	359471	178	hypothetica	36.91018	64.04194	-0.795	0.26514	0.49704	-1.73508	FALSE
Spy0353	-	359613	359996	127	hypothetica	NA	NA	NA	NA	NA	NA	Na
Spy0354	-	360090	360722	210	hypothetica	233.8737	212.6588	0.13719	0.72675	0.85822	1.099761	FALSE
Spy0355	-	361401	361721	106	hypothetica	4.687086	57.15672	-3.6082	0.015853	0.079657	-12.1948	FALSE
Spy0356	speJ	362034	362732	232	exotoxin ty	212.9942	388.831	-0.86833	0.092207	0.25794	-1.82555	FALSE
Spy0357	-	362980	363594	204	hypothetica	251.3067	356.352	-0.50385	0.28826	0.51984	-1.41799	FALSE
Spy0358	-	363955	364191	78	hypothetica	946.5711	615.5299	0.62088	0.073794	0.22353	1.537813	FALSE
Spy0359	fabG	364175	364882	235	3-ketoacyl-	5623.884	3552.69	0.66265	0.039164	0.1479	1.582988	FALSE
Spy0360	-	364928	365917	329	NAD-deper	18211.48	12282.77	0.56821	0.083383	0.24065	1.482683	FALSE
Spy0361	-	366250	367587	445	phosphogly	7302.55	5025.783	0.53905	0.10131	0.27539	1.453015	FALSE
Spy0362	glmU	367760	369142	460	bifunctiona	6461.7	4441.877	0.54074	0.10341	0.27902	1.454718	FALSE
Spy0363	-	369173	369727	184	phosphohy	3078.451	1995.624	0.62536	0.076685	0.22927	1.542596	FALSE
Spy0364	-	369727	369978	83	hypothetica	173.1651	158.0282	0.13197	0.82496	0.9192	1.095789	FALSE
Spy0365	pfs	369998	370693	231	5\'-methylt	6753.355	5830.662	0.21194	0.50579	0.70791	1.158245	FALSE
Spy0366	-	370844	371185	113	hypothetica	6511.64	5719.79	0.18706	0.49973	0.70332	1.138441	FALSE
Spy0367	scaR	371286	371933	215	iron-depen	5771.266	4364.886	0.40294	0.22553	0.45011	1.3222	FALSE
Spy0368	mtsA	372091	373011	306	manganese	24652.56	17555.05	0.48985	0.11164	0.29511	1.404299	FALSE
Spy0369	mtsB	373075	373800	241	manganese	3056.447	2123.744	0.52525	0.12031	0.30728	1.439183	FALSE
Spy0370	mtsC	373801	374655	284	manganese	4478.931	4934.969	-0.13989	0.72407	0.85822	-1.10182	FALSE
Spy0371	-	374803	375609	268	peptidyl-pr	7070.576	4203.823	0.75013	0.022665	0.10284	1.681944	FALSE
Spy0372	ftsK	375826	378231	801	cell divisior	4604.942	5500.14	-0.25629	0.44632	0.66457	-1.1944	FALSE
Spy0373	-	378301	378654	117	hypothetica	2592.079	2548.734	0.024328	0.83055	0.92244	1.017006	FALSE
Spy0374	rplK	378898	379323	141	50S ribosor	22911.76	49947.52	-1.1243	0.082995	0.23991	-2.17996	FALSE
Spy0375	rplA	379429	380118	229	50S ribosor	38641.35	71069.47	-0.87908	0.098851	0.27032	-1.8392	FALSE
Spy0376	-	380469	381602	377	transposase	0	0	NA	NA	NA	NA	NA
Spy0377	-	381767	381937	56	uridylate ki	4517.287	3154.206	0.51818	0.1504	0.34724	1.432147	FALSE
Spy0378	pyrH	381912	382133	73	uridylate ki	NA	NA	NA	NA	NA	NA	Na
Spy0379	-	382121	382495	124	uridylate ki	NA	NA	NA	NA	NA	NA	Na
Spy0380	frr	382524	383081	185	ribosome re	6915.33	4349.554	0.66893	0.05325	0.18223	1.589893	FALSE
Spy0381	-	383190	384047	285	S1 RNA-bin	1949.233	1684.415	0.21066	0.70191	0.84791	1.157217	FALSE

Spy0382	msrA.2	384120	384629	169	methionine	808.6374	894.572	-0.1457	0.64235	0.81627	-1.10627	FALSE
Spy0383	-	384626	384841	71	hypothetica	274.7132	374.769	-0.44808	0.25744	0.48783	-1.36422	FALSE
Spy0384	-	384997	386166	389	surface ant	5628.776	7333.11	-0.3816	0.24553	0.478	-1.30279	FALSE
Spy0385	-	386441	388252	603	hypothetica	26239.18	27961.66	-0.09173	0.78735	0.8943	-1.06565	FALSE
Spy0386	phoH	388411	389463	350	phoH prote	1565.498	1233.177	0.34424	0.36071	0.58536	1.269482	FALSE
Spy0387	-	389509	390084	191	uracil DNA	1185.358	910.8616	0.38002	0.33173	0.55359	1.30136	FALSE
Spy0388	-	390192	390740	182	metallopro	1745.462	1228.922	0.50622	0.14926	0.34724	1.420324	FALSE
Spy0389	dgk	390721	391128	135	diacylglyce	982.8856	701.5205	0.48654	0.20778	0.42925	1.401081	FALSE
Spy0390	era	391248	392144	298	GTP-bindin	6130.05	3315.444	0.8867	0.010377	0.06028	1.848942	FALSE
Spy0391	-	392128	392640	170	phosphohy	3405.125	2100.884	0.69671	0.055669	0.18769	1.620804	FALSE
Spy0392	-	392945	393199	84	hypothetica	37.24486	77.99135	-1.0663	0.14035	0.33421	-2.09406	FALSE
Spy0393	-	393597	393842	81	hypothetica	51.86076	77.89559	-0.5869	0.43623	0.65389	-1.50202	FALSE
Spy0394	-	393857	394039	60	hypothetica	17.97505	41.54807	-1.2088	0.18438	0.3943	-2.31145	FALSE
Spy0395	-	394230	394409	59	transposase	NA	NA	NA	NA	NA	NA	Na
Spy0396	-	394348	394497	49	transposase	NA	NA	NA	NA	NA	NA	Na
Spy0397	-	394494	394916	140	transposase	112.7039	266.4971	-1.2416	0.011505	0.064858	-2.36461	FALSE
Spy0398	-	395189	395398	69	bacteriocin	NA	NA	NA	NA	NA	NA	Na
Spy0399	-	395463	395663	66	hypothetica	8.45692	8.00866	0.078572	1	1	1.055972	FALSE
Spy0400	silD	395957	396151	64	hypothetica	74.49835	121.7853	-0.70906	0.21061	0.43346	-1.63474	FALSE
Spy0401	-	396344	396460	38	hypothetica	NA	NA	NA	NA	NA	NA	Na
Spy0402	-	396644	397639	331	hypothetica	232.5369	341.9152	-0.55618	0.16232	0.36283	-1.47037	FALSE
Spy0403	-	397684	398130	148	hypothetica	131.6019	187.2797	-0.50901	0.27465	0.50704	-1.42307	FALSE
Spy0404	-	398478	398747	89	hypothetica	104.4407	106.2264	-0.02446	0.97794	0.99967	-1.0171	FALSE
Spy0405	-	398948	399055	35	hypothetica	NA	NA	NA	NA	NA	NA	Na
Spy0406	-	399269	399511	80	hypothetica	102.5051	118.0833	-0.20411	0.64855	0.81865	-1.15198	FALSE
Spy0407	mutR	399818	400684	288	transcriptio	314.959	332.6581	-0.07888	0.84919	0.93647	-1.05619	FALSE
Spy0408	fpg	400856	401683	275	formamido	604.503	578.6739	0.062999	0.94441	0.98689	1.044635	FALSE
Spy0409	coaE	401593	402273	226	dephospho	706.4711	858.0963	-0.28051	0.46992	0.68695	-1.21462	FALSE
Spy0410	-	402463	403965	500	ATPase	10829.04	13239.24	-0.28991	0.38003	0.60098	-1.22256	FALSE
Spy0411	-	404087	405280	397	multidrug r	7022.921	8982.494	-0.35504	0.28808	0.51984	-1.27902	FALSE
Spy0412	rpmG	405277	405423	48	50S riboson	19.8759	36.26806	-0.86768	0.36675	0.59252	-1.82473	FALSE
Spy0413	secG	405469	405705	78	preprotein	1571.422	3853.428	-1.2941	0.000228	0.004192	-2.45224	TRUE
Spy0414	-	405802	408132	776	exoribonuc	7609.652	14765.63	-0.95634	0.062197	0.20222	-1.94038	FALSE
Spy0415	smpB	408135	408602	155	SsrA-bindin	849.5574	1566.068	-0.88236	0.079943	0.23477	-1.84339	FALSE
Spy0416	-	408617	409327	236	glutaminyl-	2105.709	4740.977	-1.1709	0.000571	0.007602	-2.25152	TRUE
Spy0417	pcp	409443	410090	215	pyrrolidone	10864.55	6760.888	0.68434	0.03299	0.13248	1.606967	FALSE
Spy0418	-	410140	411066	308	permease	6455.632	3810.771	0.76048	0.020908	0.097057	1.694054	FALSE
Spy0419	-	411066	411749	227	permease	3530.682	2268.909	0.63795	0.04913	0.17333	1.556116	FALSE

Spy0420	-	411960	412886	308	glucosyltra	1839.462	965.7906	0.9295	0.008185	0.0501	1.904616	FALSE
Spy0421	gloA	413031	413408	125	lactoylgluta	3017.817	3384.859	-0.16559	0.73935	0.86377	-1.12162	FALSE
Spy0422	-	413419	414084	221	NAD(P)H-d	6023.491	7170.932	-0.25156	0.43533	0.65353	-1.19049	FALSE
Spy0423	pepQ	414133	415218	361	Xaa-Pro dip	10330.67	10772.04	-0.06036	0.87564	0.94808	-1.04272	FALSE
Spy0424	ccpA	415392	416393	333	catabolite c	13141.98	11604.69	0.17948	0.53229	0.73	1.132476	FALSE
Spy0425	-	416524	417522	332	glycosyltra	8239.436	7492.394	0.13712	0.68297	0.83909	1.099708	FALSE
Spy0426	-	417524	418858	444	1,2-diacylgl	18172.92	18070.27	0.008172	0.97884	0.99967	1.005681	FALSE
Spy0427	thrS	419280	421223	647	threonyl-tR	10505.24	11439.47	-0.12291	0.76298	0.87692	-1.08893	FALSE
Spy0428	drrA	421364	422356	330	daunorubic	225.1588	334.188	-0.56972	0.19329	0.40665	-1.48424	FALSE
Spy0429	-	422358	423176	272	daunorubic	159.4275	249.4621	-0.64592	0.16661	0.36882	-1.56474	FALSE
Spy0430	-	423178	423963	261	ABC transp	245.0445	383.7156	-0.64699	0.12833	0.31463	-1.5659	FALSE
Spy0431	-	424164	424313	49	dihydroxya	3.6744	10.07396	-1.4551	0.47189	0.68695	-2.74176	FALSE
Spy0432	-	424708	425856	382	acetyl-CoA	95.01279	248.0305	-1.3843	0.19569	0.41111	-2.61045	FALSE
Spy0433	-	425813	427060	415	long-chain-	132.5673	269.1762	-1.0218	0.21317	0.43443	-2.03045	FALSE
Spy0434	-	427116	428150	344	hypothetica	1395.805	1469.862	-0.07458	0.77897	0.88774	-1.05306	FALSE
Spy0435	vicR	428312	429022	236	two-compo	3229.527	2444.865	0.40157	0.26492	0.49704	1.320945	FALSE
Spy0436	vicK	429015	430367	450	two-compo	8285.863	6379.645	0.37718	0.26734	0.49887	1.298801	FALSE
Spy0437	vicX	430371	431180	269	Zn-depende	3089.945	2831.005	0.12627	0.72279	0.85775	1.091468	FALSE
Spy0438	rnc	431624	432316	230	ribonucleas	528.8944	316.0279	0.74293	0.086594	0.24834	1.673571	FALSE
Spy0439	smc	432317	435856	1179	chromoson	7050.523	4458.881	0.66105	0.067428	0.21315	1.581233	FALSE
Spy0440	-	436109	436960	283	transcriptio	209.1394	441.3825	-1.0776	0.014646	0.076989	-2.11052	FALSE
Spy0441	aroE	437234	438106	290	shikimate 5	27.31897	32.49318	-0.25023	0.75363	0.87149	-1.1894	FALSE
Spy0442	-	438205	438939	244	hypothetica	32.99419	39.45634	-0.25805	0.7486	0.86782	-1.19586	FALSE
Spy0443	-	438941	439675	244	hypothetica	49.60229	37.49147	0.40384	0.6579	0.82193	1.323025	FALSE
Spy0444	-	439668	440654	328	hypothetica	87.10042	52.56529	0.72857	0.30681	0.53707	1.656996	FALSE
Spy0445	metK1	440664	441863	399	S-adenosyl	181.755	169.0954	0.10416	0.91069	0.9697	1.074868	FALSE
Spy0446	-	441847	442815	322	hypothetica	134.2824	106.3031	0.33709	0.55618	0.749	1.263206	FALSE
Spy0447	-	442870	443859	329	cell wall bio	148.8978	131.6573	0.17753	0.74887	0.86782	1.130946	FALSE
Spy0448	-	444385	444528	47	hypothetica	NA	NA	NA	NA	NA	NA	Na
Spy0449	-	444552	445709	385	UDP-glucos	350.3939	307.5631	0.1881	0.64318	0.81627	1.139262	FALSE
Spy0450	mefE	445794	447002	402	macrolide-e	634.3725	712.8073	-0.16818	0.69091	0.84352	-1.12364	FALSE
Spy0451	-	447105	447314	69	transcriptio	3.052379	5.407064	-0.82491	0.72552	0.85822	-1.77142	FALSE
Spy0452	-	447304	448149	281	chromoson	98.51006	52.41103	0.9104	0.16353	0.36372	1.879567	FALSE
Spy0453	-	448234	448962	242	chromoson	87.58974	45.37195	0.94896	0.17011	0.37204	1.930481	FALSE
Spy0454	-	448953	449177	74	hypothetica	0.608868	0.612028	-0.00747	1	1	-1.00519	FALSE
Spy0455	-	449177	450271	364	hypothetica	173.6633	96.12468	0.85331	0.11123	0.29446	1.806641	FALSE
Spy0456	-	450317	450619	100	plasmid sta	446.434	452.3182	-0.01889	0.97495	0.99967	-1.01318	FALSE
Spy0457	-	450619	450924	101	plasmid sta	436.7327	502.3271	-0.20188	0.66329	0.82698	-1.1502	FALSE

Spy0458	-	450943	451215	90	hypothetica	802.5665	937.2612	-0.22383	0.52431	0.72393	-1.16783	FALSE
Spy0459	-	451320	451772	150	portal prot	25.16557	19.307	0.38233	0.84349	0.9332	1.303445	FALSE
Spy0460	-	451838	452152	104	hypothetica	26.67284	33.84463	-0.34356	0.74583	0.86704	-1.26888	FALSE
Spy0461	-	452569	452907	112	hypothetica	239.8314	171.8795	0.48062	0.30754	0.53783	1.395343	FALSE
Spy0462	-	452931	453755	274	asparagine	522.4504	420.4932	0.31321	0.43292	0.65044	1.242469	FALSE
Spy0463	-	453769	455016	415	hypothetica	1477.195	997.876	0.56593	0.25122	0.48336	1.480341	FALSE
Spy0464	mccF	455244	455960	238	microcin C7	1397.553	926.5112	0.59302	0.11649	0.30267	1.508401	FALSE
Spy0465	-	455973	456173	66	hypothetica	398.9671	392.3375	0.024175	0.99813	1	1.016898	FALSE
Spy0466	-	456293	457192	299	hypothetica	2382.134	2383.895	-0.00107	0.98195	1	-1.00074	FALSE
Spy0467	-	457645	458358	237	transposase	NA	NA	NA	NA	NA	NA	Na
Spy0468	-	458406	458681	91	transposase	NA	NA	NA	NA	NA	NA	Na
Spy0469	-	458701	458919	72	hypothetica	NA	NA	NA	NA	NA	NA	Na
Spy0470	-	458986	459783	265	HAD superf	1540.496	935.9117	0.71895	0.06111	0.19977	1.645984	FALSE
Spy0471	-	459787	460611	274	HAD superf	2418.747	1295.455	0.9008	0.016427	0.08164	1.867101	FALSE
Spy0472	ftsY	460611	462161	516	cell division	9252.513	7257.344	0.3504	0.31168	0.53975	1.274914	FALSE
Spy0473	-	462215	463582	455	multidrug r	2815.354	2224.848	0.33961	0.28638	0.51976	1.265414	FALSE
Spy0474	licT	463910	464752	280	BigG family	502.0145	441.2043	0.18628	0.54589	0.7392	1.137826	FALSE
Spy0475	-	464916	466616	566	PTS system	1243.81	1178.256	0.078113	0.77694	0.88683	1.055636	FALSE
Spy0476	bglA	466635	468059	474	6-phospho-	1393.798	1571.14	-0.17279	0.69139	0.84354	-1.12724	FALSE
Spy0477	-	468158	468973	271	hypothetica	4561.832	2045.801	1.1569	0.000953	0.011069	2.229778	TRUE
Spy0478	-	468973	469875	300	hypothetica	3370.645	1596.118	1.0785	0.002082	0.01947	2.111839	TRUE
Spy0479	-	470009	470206	65	hypothetica	NA	NA	NA	NA	NA	NA	Na
Spy0480	-	470412	472544	710	transcriptio	4913.404	3717.332	0.40246	0.24971	0.48249	1.32176	FALSE
Spy0481	-	470304	470432	42	hypothetica	NA	NA	NA	NA	NA	NA	Na
Spy0482	-	472486	472968	160	hypothetica	1083.204	1115.675	-0.04261	0.95021	0.98913	-1.02998	FALSE
Spy0483	-	473032	473304	90	stress-resp	5475.244	8503.349	-0.63511	0.11747	0.30392	-1.55306	FALSE
Spy0484	ptsK	473609	474601	330	HPr kinase/	6742.041	4147.067	0.70109	0.043524	0.15973	1.625733	FALSE
Spy0485	lgt	474598	475377	259	prolipoprot	4871.948	2780.489	0.80916	0.024261	0.1082	1.752191	FALSE
Spy0486	-	475399	475806	135	hypothetica	2440.304	1464.924	0.73624	0.034133	0.13476	1.665829	FALSE
Spy0487	-	475799	476227	142	hypothetica	7097.559	4056.155	0.80721	0.014302	0.076053	1.749824	FALSE
Spy0488	-	476386	476670	94	hypothetica	3011.639	7735.673	-1.361	0.011401	0.064662	-2.56863	FALSE
Spy0489	-	476869	477237	122	U32 family	1649.278	1767.74	-0.10007	0.85184	0.93826	-1.07183	FALSE
Spy0490	-	477209	477796	195	U32 family	NA	NA	NA	NA	NA	NA	Na
Spy0491	-	477887	479173	428	U32 family	4431.283	5303.907	-0.25933	0.49045	0.70006	-1.19692	FALSE
Spy0492	-	479186	479314	42	hypothetica	NA	NA	NA	NA	NA	NA	Na
Spy0493	-	479383	479595	70	hypothetica	1007.834	1691.34	-0.74691	0.4142	0.63164	-1.67819	FALSE
Spy0494	-	479692	479832	46	hypothetica	NA	NA	NA	NA	NA	NA	Na
Spy0495	lysS	479968	481461	497	lysyl-tRNA s	13722.5	12067.51	0.18542	0.49921	0.70332	1.137148	FALSE

Spy0496	-	481635	482537	300	HAD superf	966.9028	978.2678	-0.01686	0.92699	0.98183	-1.01175	FALSE
Spy0497	-	482645	483268	207	phosphogly	1879.34	1990.31	-0.08277	0.89174	0.95622	-1.05905	FALSE
Spy0498	-	483609	484088	159	transcriptio	1695.256	1952.618	-0.20391	0.63203	0.8118	-1.15182	FALSE
Spy0499	-	484179	484742	187	thiamine tr	1519.913	2889.855	-0.92701	0.006926	0.046095	-1.90133	TRUE
Spy0500	-	485011	485859	282	N-acetylmu	2691.706	1992.393	0.43402	0.19815	0.4143	1.350993	FALSE
Spy0501	-	486184	486687	167	hypothetica	910.2864	575.2753	0.66207	0.056095	0.18878	1.582351	FALSE
Spy0502	-	486671	487057	128	hypothetica	550.7071	295.6689	0.8973	0.021773	0.10003	1.862577	FALSE
Spy0503	-	487103	487582	159	glutathione	1251.44	1516.988	-0.27762	0.4253	0.64111	-1.21219	FALSE
Spy0504	pepF	487575	489374	599	oligoendop	4412.141	4497.018	-0.02749	0.92949	0.98271	-1.01924	FALSE
Spy0505	ppc	489518	492331	937	phosphoen	19586.52	13175.87	0.57196	0.10378	0.27937	1.486542	FALSE
Spy0506	ftsW	492501	493775	424	cell division	3959.775	3142.719	0.33341	0.31466	0.54244	1.259988	FALSE
Spy0507	-	493792	493932	46	hypothetica	NA	NA	NA	NA	NA	NA	Na
Spy0508	tuf	494129	495325	398	elongation	175359.7	138683.8	0.33852	0.28871	0.51984	1.264459	FALSE
Spy0509	tpiA	495566	496324	252	triosephosp	19784.32	21501.06	-0.12005	0.89157	0.95622	-1.08677	FALSE
Spy0510	-	496423	497658	411	factor esse	6059.71	4121.047	0.55624	0.090357	0.25613	1.470432	FALSE
Spy0511	murM	497645	498871	408	UDP-N-ace	5315.253	3319.911	0.67899	0.053829	0.18352	1.601019	FALSE
Spy0512	-	498871	499680	269	HAD superf	2941.738	1920.223	0.61539	0.064835	0.20711	1.531972	FALSE
Spy0513	-	499831	500064	77	hypothetica	158.5154	227.2735	-0.51981	0.24884	0.48133	-1.43377	FALSE
Spy0514	-	500136	501437	433	dGTP tripho	2534.249	2839.187	-0.16392	0.6955	0.8446	-1.12033	FALSE
Spy0515	-	501519	501905	128	hypothetica	852.283	1414.323	-0.73071	0.046187	0.16454	-1.65946	FALSE
Spy0516	pacL	502136	504817	893	calcium-tra	35976.42	35348.53	0.025401	0.93871	0.98523	1.017763	FALSE
Spy0517	regR	504901	505896	331	LacI family	1784.629	1735.261	0.040472	0.80359	0.90532	1.02845	FALSE
Spy0518	-	505960	507867	635	oligohyalur	94.36505	199.7715	-1.082	0.039883	0.14969	-2.11697	FALSE
Spy0519	agaD	507954	508775	273	PTS system	237.4959	516.5647	-1.121	0.00769	0.048627	-2.17498	TRUE
Spy0520	-	508762	509544	260	PTS system	NA	NA	NA	NA	NA	NA	Na
Spy0521	agaV	509563	510051	162	PTS system	117.4287	227.3957	-0.95342	0.057066	0.19134	-1.93646	FALSE
Spy0522	-	510087	511286	399	unsaturate	331.8386	543.0344	-0.71056	0.08781	0.24987	-1.63644	FALSE
Spy0523	-	511353	511883	176	hypothetica	NA	NA	NA	NA	NA	NA	Na
Spy0524	idnO	512059	512853	264	gluconate 5	340.0065	215.5338	0.65765	0.13888	0.33201	1.577511	FALSE
Spy0525	-	512878	513519	213	hypothetica	254.938	202.5297	0.33201	0.50489	0.70786	1.258766	FALSE
Spy0526	-	513548	514549	333	2-dehydro-	533.5151	450.2769	0.24472	0.55682	0.749	1.184863	FALSE
Spy0527	kgdA	514554	515189	211	keto-hydro	521.6488	465.2985	0.16492	0.66225	0.82679	1.121104	FALSE
Spy0528	-	515485	516135	216	beta-phosp	581.8305	411.895	0.49832	0.19645	0.41187	1.412568	FALSE
Spy0529	-	516776	517951	391	(Fe-S)-bind	4300.057	3027.553	0.5062	0.16293	0.36332	1.420304	FALSE
Spy0530	prfB	518105	519118	337	peptide cha	11581.4	4694.943	1.3026	0.000166	0.003507	2.46673	TRUE
Spy0531	ftsE	519137	519829	230	cell division	8211.951	3551.19	1.2094	0.000532	0.007268	2.312414	TRUE
Spy0532	ftsX	519822	520751	309	cell division	20397.55	10386.6	0.97367	0.00327	0.027264	1.96383	TRUE
Spy0533	-	521061	521696	211	hydroxyacy	4456.705	3447.013	0.37063	0.22417	0.44933	1.292917	FALSE

Spy0534	-	521927	522541	204	acetoin red	7395.377	4912.299	0.59023	0.065933	0.20988	1.505487	FALSE
Spy0535	-	522545	522691	48	acetoin deh	25.27533	23.10703	0.1294	0.96654	0.99491	1.093839	FALSE
Spy0536	dinG	522841	525300	819	bifunctiona	6584.929	3997.083	0.72022	0.032567	0.1323	1.647433	FALSE
Spy0537	aspC	525635	526828	397	aspartate a	7164.137	4964.678	0.52909	0.11071	0.29393	1.443019	FALSE
Spy0538	asnC	526849	528195	448	asparaginyl	10056.96	7114.85	0.49929	0.12571	0.3124	1.413518	FALSE
Spy0539	-	528609	529499	296	hypothetica	6289.805	5460.651	0.20394	0.58115	0.76846	1.15184	FALSE
Spy0540	-	529496	530473	325	transporter	6350.3	5268.862	0.26933	0.47171	0.68695	1.205248	FALSE
Spy0541	-	530470	531381	303	hypothetica	6385.949	5168.453	0.30517	0.39102	0.60907	1.235564	FALSE
Spy0542	pepD	531514	532911	465	dipeptidase	2664.659	2515.213	0.08327	0.77631	0.88673	1.059417	FALSE
Spy0543	-	533063	534610	515	high-affinit	3654.981	2158.039	0.76014	0.02743	0.11663	1.693655	FALSE
Spy0544	-	534758	535480	240	GntR family	2091.203	2134.363	-0.02947	0.96011	0.9922	-1.02064	FALSE
Spy0545	agaS	535499	536698	399	galactosam	8702.987	9464.446	-0.12101	0.77637	0.88673	-1.0875	FALSE
Spy0546	rpmE2	536795	537055	86	50S riboson	15454.4	33888.99	-1.1328	0.21141	0.43346	-2.19284	FALSE
Spy0547	-	537170	538111	313	phosphoes	5764.705	8331.999	-0.53142	0.31361	0.54165	-1.44535	FALSE
Spy0548	-	538505	538954	149	flavodoxin	2096.559	3117.944	-0.57257	0.11939	0.30585	-1.48717	FALSE
Spy0549	-	539130	539414	94	hypothetica	389.6503	908.5493	-1.2214	0.001546	0.015577	-2.33173	TRUE
Spy0550	-	539407	540669	420	chloride ch	2544.693	4694.312	-0.88342	0.009938	0.058288	-1.84474	FALSE
Spy0551	rplS	540784	541131	115	50S riboson	19189.74	56061.75	-1.5467	0.000172	0.003564	-2.92148	TRUE
Spy0552	-	542134	542703	189	DNA gyrase	1907.758	1718.612	0.15063	0.6836	0.83909	1.110054	FALSE
Spy0553	gyrB	542704	544656	650	DNA gyrase	18927.23	13131.43	0.52744	0.14522	0.3419	1.441369	FALSE
Spy0554	-	545024	546748	574	septation ri	25310.66	25335.98	-0.00144	0.9813	1	-1.001	FALSE
Spy0555	-	546880	547338	152	hypothetica	117.8212	98.80664	0.25392	0.60232	0.78812	1.192443	FALSE
Spy0556	eno	547565	548872	435	phosphopy	112892.5	68306.22	0.72486	0.024687	0.10899	1.65274	FALSE
Spy0557	-	549398	549988	196	transposas	NA	NA	NA	NA	NA	NA	Na
Spy0558	-	550037	550288	83	transposas	NA	NA	NA	NA	NA	NA	Na
Spy0559	-	550650	551306	218	transcriptio	NA	NA	NA	NA	NA	NA	Na
Spy0560	-	551303	552190	295	transcriptio	NA	NA	NA	NA	NA	NA	Na
Spy0561	epf	552662	558841	2059	extracellula	525.6474	579.768	-0.14138	0.71687	0.85518	-1.10296	FALSE
Spy0562	sagA	559706	559867	53	streptolysin	8921.213	20377.69	-1.1917	0.000499	0.006941	-2.28422	TRUE
Spy0563	sagB	560089	561039	316	streptolysin	3108.198	2819.244	0.14077	0.57985	0.76846	1.102493	FALSE
Spy0564	sagC	561036	562094	352	streptolysin	2697.539	2526.287	0.094626	0.69067	0.84352	1.067789	FALSE
Spy0565	sagD	562114	563472	452	streptolysin	3721.159	3649.474	0.028063	0.84367	0.9332	1.019642	FALSE
Spy0566	sagE	563447	564118	223	streptolysin	1265.618	1332.218	-0.07399	0.95099	0.98913	-1.05262	FALSE
Spy0567	sagF	564115	564798	227	streptolysin	1493.134	1400.368	0.092538	0.68304	0.83909	1.066244	FALSE
Spy0568	sagG	564821	565744	307	streptolysin	2615.247	2571.114	0.024554	0.85362	0.93908	1.017165	FALSE
Spy0569	sagH	565753	566880	375	streptolysin	3232.276	3206.284	0.011648	0.84735	0.93557	1.008106	FALSE
Spy0570	sagI	566877	567995	372	streptolysin	4026.013	4397.061	-0.12719	0.87687	0.94808	-1.09216	FALSE
Spy0571	-	568566	571298	910	hypothetica	14813.27	15669.25	-0.08105	0.89062	0.95615	-1.05778	FALSE

Spy0572	-	571576	572076	166	hypothetica	8224.159	13919.14	-0.75913	0.051898	0.17895	-1.69247	FALSE
Spy0573	ligA	572270	574228	652	NAD-deper	5883.394	5688.823	0.048519	0.978	0.99967	1.034203	FALSE
Spy0574	-	574242	575264	340	lipid kinase	4014.244	4142.41	-0.04534	0.88633	0.95435	-1.03193	FALSE
Spy0575	atpE	575657	575854	65	ATP syntha	1224.526	982.335	0.31794	0.3292	0.55129	1.246549	FALSE
Spy0576	atpB	575889	576605	238	ATP syntha	13188.24	6387.295	1.046	0.002046	0.019329	2.064797	TRUE
Spy0577	atpF	576623	577117	164	ATP syntha	9061.664	3822.003	1.2454	0.000328	0.005208	2.370843	TRUE
Spy0578	atpH	577117	577653	178	ATP syntha	9639.771	6090.959	0.66233	0.038738	0.1469	1.582637	FALSE
Spy0579	atpA	577669	579177	502	ATP syntha	75288.32	22805.47	1.723	9.09E-07	6.48E-05	3.301222	TRUE
Spy0580	atpG	579193	580068	291	ATP syntha	29188.85	9535.936	1.614	4.65E-06	0.00025	3.060994	TRUE
Spy0581	atpD	580230	581636	468	ATP syntha	89314.64	26953.8	1.7284	9.23E-07	6.48E-05	3.313601	TRUE
Spy0582	atpC	581649	582065	138	ATP syntha	23260.86	8198.663	1.5044	1.87E-05	0.000681	2.837067	TRUE
Spy0583	-	582331	582588	85	hypothetica	1838.207	1085.069	0.76051	0.030587	0.12622	1.694089	FALSE
Spy0584	murA	582815	583924	369	UDP-N-acet	16394.09	12757.58	0.36182	0.29109	0.52104	1.285046	FALSE
Spy0585	epuA	583928	584116	62	epuA prote	26.97858	41.80153	-0.63174	0.43113	0.64829	-1.54943	FALSE
Spy0586	endA	584152	584691	179	DNA-entry	1234.915	1342.581	-0.1206	0.63246	0.8118	-1.08719	FALSE
Spy0587	pheS	584974	586017	347	phenylalan	3173.784	2726.315	0.21925	0.56643	0.75691	1.164128	FALSE
Spy0588	pheT	586212	588632	806	phenylalan	13239.17	10799.12	0.2939	0.39398	0.61159	1.22595	FALSE
Spy0589	-	588742	589119	125	salt-stress i	120.4407	185.8762	-0.62602	0.16847	0.37058	-1.5433	FALSE
Spy0590	-	589112	589498	128	hypothetica	64.18282	126.8316	-0.98266	0.17457	0.37951	-1.97611	FALSE
Spy0591	-	589571	590647	358	ABC transp	4259.935	1738.893	1.2927	0.000253	0.004523	2.449861	TRUE
Spy0592	-	590657	591325	222	ABC transp	3065.012	1263.808	1.2781	0.0003	0.004976	2.425194	TRUE
Spy0593	-	591427	592344	305	neutral zinc	2004.248	3011.212	-0.58728	0.11067	0.29393	-1.50241	FALSE
Spy0594	rexB	592495	595710	1071	ATP-depen	6396.951	5842.467	0.13081	0.77993	0.88801	1.094908	FALSE
Spy0595	rexA	595671	599339	1222	ATP-depen	8699.726	7038.96	0.30561	0.44817	0.66569	1.235941	FALSE
Spy0596	-	599479	600291	270	arginine-bi	2315.609	2523.21	-0.12387	0.6651	0.8271	-1.08965	FALSE
Spy0597	rpsU	600432	600608	58	30S ribosor	1824.788	4696.995	-1.364	0.000101	0.002478	-2.57398	TRUE
Spy0598	mscL	600736	601098	120	large-condu	8165.576	5451.435	0.58292	0.080336	0.23483	1.497878	FALSE
Spy0599	dnaG	601229	603043	604	DNA prima	7238.497	12715.41	-0.81282	0.015702	0.079555	-1.75664	FALSE
Spy0600	rpoD	603052	604161	369	RNA polym	7768.325	10072.92	-0.37481	0.23576	0.46611	-1.29667	FALSE
Spy0601	-	604397	604735	112	hypothetica	2724.684	4025.403	-0.56304	0.086996	0.24911	-1.47738	FALSE
Spy0602	rmlD	604873	605727	284	dTDP-4-del	4833.868	7206.984	-0.57622	0.087424	0.24948	-1.49094	FALSE
Spy0603	rgpAc	605846	607000	384	alpha-(1,2)-	1884.721	2077.149	-0.14025	0.70429	0.84906	-1.1021	FALSE
Spy0604	rgpBc	606990	607922	310	alpha-L-Rha	1808.15	1956.885	-0.11404	0.70862	0.85091	-1.08225	FALSE
Spy0605	rgpCc	607925	608728	267	polysaccha	1282.947	1125.207	0.18927	0.65222	0.81935	1.140187	FALSE
Spy0606	rgpDc	608728	609933	401	polysaccha	3401.614	2710.574	0.32762	0.40031	0.61774	1.254941	FALSE
Spy0607	rgpEc	609958	610965	335	glycosyltrar	2783.186	1882.826	0.56384	0.12191	0.30888	1.478198	FALSE
Spy0608	rgpFc	610962	612707	581	alpha-L-Rha	5108.886	3511.846	0.54078	0.13142	0.32048	1.454759	FALSE
Spy0609	-	612704	615178	824	phosphogly	8618.31	7268.611	0.24573	0.47663	0.68865	1.185693	FALSE

Spy0610	-	615357	616052	231	glycosyltra	1736.081	1452.686	0.25711	0.47177	0.68695	1.195082	FALSE
Spy0611	-	616054	616395	113	hypothetica	214.5058	168.6917	0.34663	0.4502	0.66682	1.271587	FALSE
Spy0612	amrA	616388	617674	428	transcriptio	3618.843	3835.128	-0.08375	0.85894	0.93942	-1.05977	FALSE
Spy0613	-	617655	619151	498	hypothetica	4240.375	5009.641	-0.24052	0.54983	0.74344	-1.18142	FALSE
Spy0614	pepT	619245	620468	407	peptidase T	10092.33	7356.539	0.45616	0.15818	0.35621	1.371885	FALSE
Spy0615	ebsA	620509	620997	162	pore formi	5137.175	5174.169	-0.01035	0.95735	0.9916	-1.0072	FALSE
Spy0616	-	620984	621181	65	ferredoxin	13.71575	39.88881	-1.5402	0.097108	0.26833	-2.90835	FALSE
Spy0617	-	621230	621706	158	hypothetica	5868.889	4391.397	0.41841	0.18555	0.39583	1.336454	FALSE
Spy0618	cmk	621721	622401	226	cytidylate k	4827.366	3054.227	0.66043	0.049537	0.17376	1.580554	FALSE
Spy0619	infC	622563	623093	176	translation	3837.585	8655.583	-1.1734	0.000485	0.006881	-2.25543	TRUE
Spy0620	rpmI	623135	623332	65	50S riboso	1496.709	2908.968	-0.95871	0.007935	0.04938	-1.94357	TRUE
Spy0621	rplT	623391	623750	119	50S riboso	8832.894	18753.71	-1.0862	0.001869	0.018226	-2.12314	TRUE
Spy0622	-	624041	626212	723	phosphogly	10093.91	6541.648	0.62576	0.046478	0.16493	1.543023	FALSE
Spy0623	-	626359	627522	387	methyltran	3408.83	5501.832	-0.69064	0.060118	0.19793	-1.614	FALSE
Spy0624	aroD	627519	628205	228	3-dehydroc	2253.035	2818.718	-0.32317	0.37147	0.59484	-1.25108	FALSE
Spy0625	aroF	628299	629465	388	chorismate	5822.872	5264.097	0.14554	0.87253	0.94619	1.106145	FALSE
Spy0626	-	629526	629867	113	hypothetica	3186.365	3381.124	-0.08559	0.77819	0.88769	-1.06112	FALSE
Spy0627	gor	630088	631440	450	glutathione	3628.534	3556.389	0.028974	1	1	1.020286	FALSE
Spy0628	folC.2	631528	632796	422	folylpolyglu	6592.78	5556.458	0.24672	0.4481	0.66569	1.186506	FALSE
Spy0629	-	632826	633266	146	hypothetica	1887.826	1715.26	0.1383	0.61896	0.8007	1.100607	FALSE
Spy0630	nifS1	633501	634643	380	cysteine de	2833.152	2400.94	0.23881	0.58015	0.76846	1.180019	FALSE
Spy0631	thiI	634655	635869	404	thiamine bi	4272.528	3611.34	0.24256	0.51898	0.72042	1.18309	FALSE
Spy0632	capA	635907	637199	430	capsule bio	2496.457	2898.213	-0.21528	0.52052	0.72146	-1.16093	FALSE
Spy0633	rplU	637413	637727	104	50S riboso	7337.944	9623.075	-0.39112	0.27422	0.50682	-1.31141	FALSE
Spy0634	-	637739	638065	108	hypothetica	3241.469	4678.971	-0.52954	0.15554	0.35311	-1.44347	FALSE
Spy0635	rpmA	638093	638386	97	50S riboso	6718.528	10212.27	-0.60409	0.083868	0.24103	-1.52002	FALSE
Spy0636	-	638734	639648	304	LysR family	1702.662	2568.218	-0.59298	0.097907	0.26895	-1.50836	FALSE
Spy0637	lsp	639645	640103	152	lipoprotein	374.1092	575.865	-0.62227	0.10881	0.29016	-1.5393	FALSE
Spy0638	-	640093	640983	296	ribosomal l	2833.91	4029.969	-0.50797	0.149	0.3471	-1.42205	FALSE
Spy0639	pyrR	641379	641900	173	bifunctiona	1001.332	1376.923	-0.45953	0.21883	0.44252	-1.37509	FALSE
Spy0640	pyrP	641916	643175	419	uracil perm	2534.839	3542.933	-0.48305	0.16846	0.37058	-1.3977	FALSE
Spy0641	pyrB	643236	644171	311	aspartate c	2235.642	3058.64	-0.4522	0.28399	0.51593	-1.36812	FALSE
Spy0642	carA	644215	645297	360	carbamoyl	3420.182	4765.531	-0.47856	0.34616	0.56882	-1.39335	FALSE
Spy0643	carB	645523	648699	1058	carbamoyl	10078.39	14262.4	-0.50095	0.32469	0.54887	-1.41515	FALSE
Spy0644	-	648999	650177	392	periplasmic	14903.92	12890.82	0.20935	0.6102	0.79387	1.156167	FALSE
Spy0645	-	650177	650887	236	ABC transp	9373.553	8695.034	0.1084	0.89022	0.95615	1.078032	FALSE
Spy0646	-	650899	652119	406	ABC transp	21077.78	22839.46	-0.11581	0.64397	0.81627	-1.08358	FALSE
Spy0647	-	652373	654106	577	glyceropho	23987.44	23440.98	0.033246	0.90179	0.96409	1.023312	FALSE

Spy0648	rpsP	654233	654505	90	30S ribosor	3168.328	5960.557	-0.91173	0.17984	0.38774	-1.8813	FALSE
Spy0649	-	654515	654754	79	RNA bindin	907.4843	1849.178	-1.0269	0.004857	0.03547	-2.03764	TRUE
Spy0650	-	654763	654870	35	hypothetica	NA	NA	NA	NA	NA	NA	Na
Spy0651	-	655665	658691	1008	cell surface	2387.112	2520.265	-0.07831	0.77315	0.88415	-1.05578	FALSE
Spy0652	-	658711	659112	133	hypothetica	414.0666	424.2181	-0.03494	0.9528	0.9897	-1.02452	FALSE
Spy0653	czcD	659292	660167	291	cobalt-zinc-	2113.473	4914.606	-1.2175	0.025777	0.11168	-2.32543	FALSE
Spy0654	-	660303	660824	173	TetR family	183.6032	295.5251	-0.68669	0.11829	0.30402	-1.60959	FALSE
Spy0655	rimM	661039	661557	172	16S rRNA-p	702.974	813.7931	-0.21119	0.64989	0.81921	-1.15764	FALSE
Spy0656	trmD	661547	662278	243	tRNA (guan	665.9879	479.5353	0.47386	0.25283	0.48442	1.38882	FALSE
Spy0657	trxB	662278	663270	330	thioredoxir	1534.47	1095.567	0.48606	0.21994	0.44427	1.400615	FALSE
Spy0658	-	663448	664506	352	regulatory	7917.173	3772.601	1.0694	0.002237	0.020713	2.09856	TRUE
Spy0659	apbA	664519	665442	307	2-dehydrop	15967.78	7517.987	1.0867	0.001648	0.01634	2.123877	TRUE
Spy0660	fruR	665698	666411	237	fructose re	1549.691	782.5558	0.98571	0.006115	0.042249	1.980288	TRUE
Spy0661	fruB	666408	667319	303	1-phosphof	1871.648	1034.666	0.85514	0.01557	0.079555	1.808934	FALSE
Spy0662	fruA	667316	669262	648	PTS system	11173.2	7980.42	0.48551	0.13681	0.32975	1.400081	FALSE
Spy0663	mur1.1	669361	669954	197	autolysin	257.0393	286.694	-0.15752	0.74017	0.86377	-1.11537	FALSE
Spy0664	mur1.2	670106	670813	235	autolysin	2966.021	3979.531	-0.42407	0.42088	0.63709	-1.34171	FALSE
Spy0665	-	670870	671088	72	transposase	35.46737	47.10648	-0.40943	0.57296	0.76406	-1.32816	FALSE
Spy0666	-	671291	671410	39	hypothetica	NA	NA	NA	NA	NA	NA	Na
Spy0667	-	671385	671594	69	exotoxin ty	0.696267	17.41952	-4.6449	0.022592	0.10276	-25.0181	FALSE
Spy0668	mac	671769	672788	339	IgG-degrad	11.67094	71.1044	-2.607	0.001527	0.015543	-6.09235	TRUE
Spy0669	-	672944	673063	39	phage prot	NA	NA	NA	NA	NA	NA	Na
Spy0670	-	673287	673463	58	nucleoside	50.92744	34.30315	0.5701	0.5169	0.71971	1.484626	FALSE
Spy0671	-	673691	674071	126	transposase	1392.593	1665.188	-0.25791	0.53725	0.73459	-1.19575	FALSE
Spy0672	-	674071	674919	282	degV family	6720.769	7661.594	-0.18902	0.71391	0.85433	-1.13999	FALSE
Spy0673	papS	675044	676252	402	tRNA CCA-p	5103.308	2721.428	0.90707	0.011324	0.064548	1.875233	FALSE
Spy0674	-	676249	678126	625	ABC transp	11536.68	7116.066	0.69708	0.049802	0.17404	1.62122	FALSE
Spy0675	-	678383	678535	50	hypothetica	NA	NA	NA	NA	NA	NA	Na
Spy0676	-	678544	678762	72	hypothetica	901.9926	942.8981	-0.06399	1	1	-1.04535	FALSE
Spy0677	fms	679018	679428	136	peptide def	2717.046	2498.152	0.12118	0.69532	0.8446	1.087624	FALSE
Spy0678	-	679510	681522	670	5\'-nucleot	477.9792	831.1764	-0.79821	0.04548	0.16328	-1.73894	FALSE
Spy0679	-	681742	682392	216	GTP pyroph	3368.689	2100.864	0.6812	0.037909	0.14496	1.603473	FALSE
Spy0680	-	682395	683063	222	two-compo	2690.883	1643.257	0.71152	0.036635	0.14187	1.637528	FALSE
Spy0681	-	683072	684304	410	two-compo	9903.753	6249.462	0.66424	0.040525	0.15116	1.584733	FALSE
Spy0682	mvaK1	684583	685476	297	mevalonate	3590.399	2215.498	0.69651	0.042506	0.15694	1.62058	FALSE
Spy0683	mvaD	685458	686402	314	diphosphor	4345.002	2608.934	0.7359	0.033999	0.13452	1.665436	FALSE
Spy0684	mvaK2	686395	687402	335	phosphome	5090.124	2890.316	0.81647	0.018208	0.088288	1.761092	FALSE
Spy0685	-	687395	688384	329	isopenteny	7963.429	4670.088	0.76994	0.02486	0.109	1.705199	FALSE

Spy0686	-	688614	689891	425	3-hydroxy-3	5498.98	4466.285	0.30009	0.33878	0.55973	1.231221	FALSE
Spy0687	mvaS.1	689878	691053	391	hydroxyme	3414.737	2246.802	0.6039	0.062343	0.20234	1.51982	FALSE
Spy0688	thyA	691262	692101	279	thymidylate	1569.75	855.2116	0.87618	0.014842	0.077793	1.835509	FALSE
Spy0689	dyr	692181	692678	165	dihydrofola	2019.313	763.9292	1.4024	0.000102	0.002478	2.64341	TRUE
Spy0690	-	692698	692868	56	hypothetica	495.9224	194.8557	1.3477	0.002056	0.019329	2.545061	TRUE
Spy0691	clpX	692998	694227	409	ATP-depen	9989.78	4970.131	1.0072	0.004793	0.035254	2.010006	TRUE
Spy0692	engB	694237	694836	199	ribosome b	7006.411	4267.21	0.71538	0.037425	0.14341	1.641916	FALSE
Spy0693	-	694984	695727	247	hypothetica	4531.083	5107.936	-0.17288	0.73889	0.86377	-1.12731	FALSE
Spy0694	clpL	695785	697884	699	ATP-depen	15046.66	46057.37	-1.614	2.91E-06	0.000171	-3.06099	TRUE
Spy0695	rpiA	698263	698946	227	ribose-5-ph	11504.19	5681.042	1.0179	0.0033	0.027264	2.024969	TRUE
Spy0696	deoB	699023	700234	403	phosphope	23584.16	12641.72	0.89963	0.007927	0.04938	1.865587	TRUE
Spy0697	arsC	700253	700693	146	arsenate re	5467.371	2881.017	0.92427	0.007488	0.048432	1.897724	TRUE
Spy0698	punA	700677	701486	269	purine nucl	14568.57	7327.147	0.99154	0.003345	0.027264	1.988306	TRUE
Spy0699	deoD	702149	702862	237	purine nucl	17358.86	8938.307	0.9576	0.003758	0.029296	1.942076	TRUE
Spy0700	cpsX	702855	703643	262	LytR family	5799.548	3029.899	0.93667	0.005325	0.038023	1.914105	TRUE
Spy0701	cpsY	703722	704576	284	LysR family	2771.311	2384.616	0.21681	0.49699	0.70327	1.162161	FALSE
Spy0702	-	704849	705385	178	hypothetica	2991.092	5763.46	-0.94626	0.033953	0.13452	-1.92687	FALSE
Spy0703	pyrF	705662	706354	230	orotidine 5	387.809	1169.025	-1.5919	0.075822	0.22784	-3.01446	FALSE
Spy0704	pyrE	706412	707041	209	orotate pho	379.472	1277.274	-1.751	0.15013	0.34724	-3.36592	FALSE
Spy0705	amiC	707238	708692	484	amidase	1906.588	4206.078	-1.1415	0.090838	0.25648	-2.2061	FALSE
Spy0706	-	708810	709673	287	cystine-bin	2581.964	3489.05	-0.43437	0.34886	0.57223	-1.35132	FALSE
Spy0707	-	709702	710352	216	cystine tran	1879.894	2927.262	-0.6389	0.18684	0.39785	-1.55714	FALSE
Spy0708	ung	710485	711138	217	uracil-DNA	1388.236	1524.499	-0.13508	0.66597	0.82748	-1.09815	FALSE
Spy0709	pyrC	711270	712538	422	dihydrooro	4608.691	4556.537	0.016419	0.99845	1	1.011446	FALSE
Spy0710	-	712596	713237	213	glycerol-3-p	2497.834	4662.914	-0.90055	0.18693	0.39785	-1.86678	FALSE
Spy0711	parE	713372	715321	649	DNA topois	14321.06	8377.459	0.77355	0.036386	0.14151	1.709471	FALSE
Spy0712	parC	715412	717871	819	DNA topois	25817.99	18198.23	0.50458	0.17941	0.38772	1.41871	FALSE
Spy0713	bcaT	717994	719016	340	branched-c	2557.967	2227.173	0.19978	0.55243	0.74549	1.148523	FALSE
Spy0714	-	719080	719310	76	hypothetica	693.9388	4469.576	-2.6873	9.29E-06	0.000413	-6.44107	TRUE
Spy0715	rpsA	719701	720906	401	30S riboson	37794.28	31330.88	0.27058	0.41451	0.63164	1.206293	FALSE
Spy0716	-	721453	721755	100	hypothetica	3628.882	1241.356	1.5476	7.13E-06	0.000333	2.923304	TRUE
Spy0717	-	721954	722940	328	hypothetica	6625.945	2902.479	1.1908	0.000675	0.008414	2.282793	TRUE
Spy0718	-	723011	723202	63	hypothetica	74.1014	54.75624	0.43648	0.66477	0.8271	1.353298	FALSE
Spy0719	-	723251	723670	139	glutathione	621.0913	488.2287	0.34725	0.69491	0.8446	1.272133	FALSE
Spy0720	-	723907	724815	302	exfoliative	2839.121	3172.162	-0.16002	0.60388	0.78903	-1.1173	FALSE
Spy0721	-	724933	725109	58	hypothetica	4212.073	3252.154	0.37314	0.46058	0.67969	1.295169	FALSE
Spy0722	miaA	725243	726142	299	tRNA delta	697.4123	763.3423	-0.13032	0.66931	0.82936	-1.09454	FALSE
Spy0723	hflX	726215	727453	412	GTP-bindin	4848.956	3663.506	0.40445	0.40665	0.62301	1.323584	FALSE

Spy0724	-	727446	728081	211	hypothetica	1739.772	1370.049	0.34467	0.57354	0.76416	1.26986	FALSE
Spy0725	elaC	728096	729025	309	ribonucleas	3000.952	2369.624	0.34076	0.48344	0.69487	1.266424	FALSE
Spy0726	-	729025	729789	254	short chain	4200.534	3458.018	0.28063	0.49552	0.7022	1.214725	FALSE
Spy0727	recJ	729786	731996	736	single-strar	9598.169	8803.991	0.1246	0.90452	0.96459	1.090205	FALSE
Spy0728	apt	732147	732665	172	adenine ph	5756.775	4103.441	0.48843	0.1778	0.38516	1.402917	FALSE
Spy0729	dnaD	732746	733429	227	DNA replica	6040.832	3408.57	0.82558	0.022757	0.103	1.772247	FALSE
Spy0730	nth	733426	734082	218	endonuclea	5730.775	3397.047	0.75445	0.040821	0.1516	1.686988	FALSE
Spy0731	-	734154	734738	194	para-aminc	749.1335	828.1066	-0.14459	0.7294	0.85871	-1.10542	FALSE
Spy0732	-	734829	735617	262	NIF3-relate	1615	1429.861	0.17566	0.73136	0.85953	1.129481	FALSE
Spy0733	-	735657	736763	368	glycine/D-a	2724.382	2580.379	0.078346	0.99551	1	1.055807	FALSE
Spy0734	cpsFO	736821	737690	289	glucose-1-p	8785.205	6967.958	0.33434	0.32305	0.54887	1.2608	FALSE
Spy0735	cpsFP	737690	738283	197	dTDP-4-deh	6085.983	4609.51	0.40088	0.26224	0.49413	1.320313	FALSE
Spy0736	cpsFQ	738527	739567	346	dTDP-gluco	18293.78	12844.44	0.51021	0.14293	0.33813	1.424257	FALSE
Spy0737	-	739788	740264	158	7,8-dihydro	831.6946	963.6016	-0.21238	0.58817	0.77405	-1.1586	FALSE
Spy0738	-	740322	741503	393	hypothetica	2592.033	2430.576	0.092786	0.75463	0.87172	1.066428	FALSE
Spy0739	-	741493	742740	415	hypothetica	3798.054	3517.574	0.11068	0.72919	0.85871	1.079737	FALSE
Spy0740	fbp	742799	744085	428	fibronectin	2510.304	1749.095	0.52125	0.12666	0.31366	1.435198	FALSE
Spy0741	-	744178	744450	90	fibronectin	NA	NA	NA	NA	NA	NA	Na
Spy0742	-	744497	744622	41	hypothetica	NA	NA	NA	NA	NA	NA	Na
Spy0743	-	744804	745802	332	ABC transp	973.3965	628.644	0.63078	0.12014	0.30728	1.548402	FALSE
Spy0744	-	745792	745971	59	hypothetica	101.4261	82.22842	0.30272	0.69728	0.84545	1.233468	FALSE
Spy0745	-	746148	747017	289	ABC transp	1636.884	1334.619	0.29452	0.38359	0.60368	1.226477	FALSE
Spy0746	-	747014	747772	252	ABC transp	2276.748	2634.613	-0.21062	0.61478	0.79755	-1.15719	FALSE
Spy0747	-	747976	749637	553	Zn-depende	21034.51	13639.18	0.625	0.062875	0.20262	1.542211	FALSE
Spy0748	estA	749770	750555	261	acetyl ester	2869.851	2264.046	0.34207	0.32403	0.54887	1.267574	FALSE
Spy0749	-	750585	750911	108	hypothetica	396.9939	367.0603	0.1131	0.80407	0.90532	1.08155	FALSE
Spy0750	-	750957	752864	635	ABC transp	8422.199	5997.767	0.48977	0.15863	0.35676	1.404221	FALSE
Spy0751	acoA	753149	754117	322	pyruvate de	13006.5	5187.11	1.3262	0.000118	0.002734	2.507414	TRUE
Spy0752	acoB	754173	755174	333	pyruvate de	22551.65	10280.63	1.1333	0.000971	0.011211	2.193599	TRUE
Spy0753	acoC	755359	756768	469	branched-c	36077.06	16912	1.093	0.001328	0.01445	2.133172	TRUE
Spy0754	-	756823	757062	79	hypothetica	9.356486	18.26051	-0.96469	0.49924	0.70332	-1.95164	FALSE
Spy0755	acoL	757095	758858	587	dihydrolipo	51569.78	26286.94	0.97218	0.003847	0.029862	1.961803	TRUE
Spy0756	-	758874	759086	70	hypothetica	16.71332	9.258232	0.85219	0.56029	0.75083	1.805239	FALSE
Spy0757	hylA	759532	761949	805	hyaluronate	4159.131	3281.122	0.34209	0.32668	0.55071	1.267592	FALSE
Spy0758	lplB	762182	763171	329	lipoate-pro	1877.066	1664.328	0.17354	0.53439	0.73123	1.127822	FALSE
Spy0759	-	763280	764071	263	hypothetica	2604.319	2845.233	-0.12764	0.69203	0.84358	-1.09251	FALSE
Spy0760	-	764071	765414	447	UDP-N-acet	2652.187	2654.732	-0.00138	0.9772	0.99967	-1.00096	FALSE
Spy0761	-	765521	766372	283	hypothetica	981.4915	1054.399	-0.10337	0.85264	0.93857	-1.07428	FALSE

Spy0762	-	766369	767325	318	hypothetica	1576.625	1460.174	0.1107	0.75102	0.8692	1.079752	FALSE
Spy0763	glmM	767379	768734	451	phosphoglu	12777.17	12403.54	0.042817	0.81351	0.91243	1.030123	FALSE
Spy0764	-	768868	769509	213	hypothetica	276.6797	212.9597	0.37764	0.39295	0.61092	1.299215	FALSE
Spy0765	hemN	769506	770702	398	coproporph	3012.766	1232.629	1.2893	0.000195	0.003782	2.444094	TRUE
Spy0766	-	770712	771464	250	acyl-ACP th	2217.901	812.4557	1.4488	4.70E-05	0.00126	2.729809	TRUE
Spy0767	-	771464	772228	254	4-nitropher	2661.793	1328.769	1.0023	0.004354	0.032949	2.003191	TRUE
Spy0768	-	772228	772860	210	hypothetica	3169.137	1460.424	1.1177	0.001408	0.014936	2.170007	TRUE
Spy0769	-	773340	777446	1368	hypothetica	1066.76	1849.509	-0.79391	0.38294	0.60361	-1.73377	FALSE
Spy0770	-	777446	778315	289	hypothetica	215.9956	365.8629	-0.7603	0.33572	0.5577	-1.69384	FALSE
Spy0771	-	778312	778653	113	hypothetica	57.30594	84.41996	-0.5589	0.31388	0.54165	-1.47315	FALSE
Spy0772	-	778643	779305	220	hypothetica	222.9911	360.6105	-0.69345	0.12156	0.30888	-1.61715	FALSE
Spy0773	-	779352	779639	95	hypothetica	NA	NA	NA	NA	NA	NA	Na
Spy0774	-	779772	779951	59	nucleoside	842.2105	785.2449	0.10104	0.69889	0.8459	1.072546	FALSE
Spy0775	-	780015	780161	48	nucleoside	216.4432	327.4253	-0.59718	0.15737	0.35526	-1.51276	FALSE
Spy0776	lepA	780285	782117	610	GTP-bindin	8419.3	10671.95	-0.34205	0.29977	0.52935	-1.26756	FALSE
Spy0777	-	782375	783256	293	hypothetica	8244.801	11558.43	-0.48739	0.15058	0.34724	-1.40191	FALSE
Spy0778	msrB	783442	783879	145	methionine	677.6222	938.2031	-0.46942	0.19026	0.40212	-1.38455	FALSE
Spy0779	-	783994	785013	339	hypothetica	6876.934	7754.298	-0.17323	0.64666	0.8174	-1.12758	FALSE
Spy0780	-	785220	785645	141	PTS system	747.6949	2045.672	-1.4521	0.13326	0.32322	-2.73606	FALSE
Spy0781	ptsB	785664	786155	163	PTS system	3199.721	6945.688	-1.1182	0.1553	0.35311	-2.17076	FALSE
Spy0782	ptsC	786172	786981	269	PTS system	6540.495	14140.27	-1.1123	0.072395	0.22082	-2.1619	FALSE
Spy0783	ptsD	786978	787805	275	PTS system	6072.962	15074.93	-1.3117	0.068456	0.21491	-2.48234	FALSE
Spy0784	-	787941	789590	549	two-compo	1174.097	1950.478	-0.73228	0.11691	0.30334	-1.66126	FALSE
Spy0785	-	789594	790382	262	two-compo	480.3421	676.4881	-0.494	0.31494	0.54244	-1.40834	FALSE
Spy0786	-	790376	791422	348	iron(III)-bin	1009.543	1343.62	-0.41242	0.28738	0.51984	-1.33092	FALSE
Spy0787	-	791519	791866	115	hypothetica	NA	NA	NA	NA	NA	NA	Na
Spy0788	-	792201	792767	188	acetyltrans	478.9957	459.7938	0.059026	0.95971	0.9922	1.041762	FALSE
Spy0789	-	792783	793472	229	HAD superf	519.8958	435.4149	0.25583	0.6133	0.79676	1.194022	FALSE
Spy0790	gabD	793567	794964	465	succinate-s	3046.706	2680.491	0.18475	0.63289	0.8118	1.13662	FALSE
Spy0791	uvrC	795066	796862	598	excinucleas	4567.305	3897.477	0.2288	0.53073	0.72877	1.17186	FALSE
Spy0792	-	797047	797649	200	NAD(P)H-de	3552.052	2756.967	0.36557	0.29545	0.52646	1.288391	FALSE
Spy0793	-	797774	799183	469	dipeptidase	10474.2	9351.408	0.16358	0.65414	0.81947	1.120063	FALSE
Spy0794	trmE	799251	800627	458	tRNA modi	2476.946	3461.328	-0.48276	0.19197	0.40469	-1.39741	FALSE
Spy0795	rplJ	800960	801460	166	50S ribosor	20034.32	40933.89	-1.0308	0.076593	0.22927	-2.04316	FALSE
Spy0796	rplL	801525	801890	121	50S ribosor	14886.91	27049.36	-0.86155	0.0174	0.085086	-1.81699	FALSE
Spy0797	-	802286	802426	46	hypothetica	NA	NA	NA	NA	NA	NA	Na
Spy0798	-	802420	802788	122	IFN-respon	7.385922	71.05535	-3.2661	0.1277	0.31447	-9.62042	FALSE
Spy0799	-	802767	802904	45	hypothetica	NA	NA	NA	NA	NA	NA	Na

Spy0800	-	802963	803286	107	DNA-cytosi	15.61836	13.61964	0.19755	0.99425	1	1.146749	FALSE
Spy0801	-	803344	803466	40	relaxase	NA	NA	NA	NA	NA	NA	Na
Spy0802	-	803599	803811	70	relaxase	NA	NA	NA	NA	NA	NA	Na
Spy0803	srtI	804076	804771	231	lantibiotic p	3.227175	12.1653	-1.9144	0.27773	0.50965	-3.76957	FALSE
Spy0804	srtR	804922	805608	228	nisin biosyr	427.5607	427.9762	-0.0014	0.90278	0.96409	-1.00097	FALSE
Spy0805	srtK	805601	806947	448	nisin biosyr	720.4185	739.5221	-0.03776	0.95474	0.99058	-1.02652	FALSE
Spy0806	srtA	807095	807235	46	lantibiotic p	59.86301	65.80193	-0.13647	0.88737	0.95484	-1.09921	FALSE
Spy0807	srtT	807350	807967	205	lantibiotic A	175.2138	83.65771	1.0665	0.033937	0.13452	2.094346	FALSE
Spy0808	srtF	808049	808738	229	lantibiotic A	359.4226	221.7388	0.69682	0.077284	0.22997	1.620928	FALSE
Spy0809	srtE	808744	809493	249	lantibiotic t	278.9066	196.3403	0.50643	0.18892	0.40069	1.420531	FALSE
Spy0810	srtG	809496	810218	240	lantibiotic t	359.2196	318.8386	0.17204	0.56107	0.75083	1.12665	FALSE
Spy0811	-	810410	810628	72	Cro/CI fami	250.3519	215.5152	0.21617	0.52497	0.72393	1.161646	FALSE
Spy0812	-	810737	810952	71	hypothetica	16.19522	19.20457	-0.24588	0.83048	0.92244	-1.18582	FALSE
Spy0813	-	811119	811250	43	integrase p	NA	NA	NA	NA	NA	NA	Na
Spy0814	-	811272	811493	73	integrase p	NA	NA	NA	NA	NA	NA	Na
Spy0815	-	811566	811787	73	integrase p	NA	NA	NA	NA	NA	NA	Na
Spy0816	-	811818	812201	127	integrase p	NA	NA	NA	NA	NA	NA	Na
Spy0817	dacA1	812371	813699	442	D-alanyl-D-	3717.77	1907.487	0.96276	0.006605	0.044956	1.949035	TRUE
Spy0818	-	813816	814778	320	polysaccha	2518.734	3369.93	-0.42002	0.58109	0.76846	-1.33795	FALSE
Spy0819	-	814866	815021	51	hypothetica	NA	NA	NA	NA	NA	NA	Na
Spy0820	folC.1	815110	816387	425	folylpolyglu	31126.52	42069.17	-0.43462	0.23076	0.459	-1.35155	FALSE
Spy0821	folE	816434	817000	188	GTP cycloh	3917.5	3583.276	0.12865	0.7206	0.85775	1.09327	FALSE
Spy0822	folP	817009	817809	266	dihydropte	5576.375	4544.471	0.29522	0.38026	0.60098	1.227072	FALSE
Spy0823	folQ	817816	818175	119	dihydroned	1098.112	868.3301	0.33871	0.31549	0.54289	1.264625	FALSE
Spy0824	folK	818172	818672	166	2-amino-4-	4602.007	4513.998	0.027857	0.90386	0.96459	1.019497	FALSE
Spy0825	murB	818822	819709	295	UDP-N-ace	2724.34	2497.515	0.12541	0.74014	0.86377	1.090818	FALSE
Spy0826	potA	819755	820909	384	spermidine	4197.791	3463.334	0.27747	0.41747	0.63402	1.212067	FALSE
Spy0827	potB	820893	821687	264	spermidine	2327.557	1914.106	0.28215	0.41671	0.63392	1.216006	FALSE
Spy0828	potC	821684	822460	258	spermidine	2172.043	1675.73	0.37426	0.29957	0.52935	1.296175	FALSE
Spy0829	-	822453	823526	357	spermidine	3979.382	2811.297	0.50131	0.13812	0.33096	1.415498	FALSE
Spy0830	dpiA	823581	824246	221	transcriptio	243.1634	250.271	-0.04157	0.94267	0.98648	-1.02923	FALSE
Spy0831	dpiB	824227	825768	513	sensor kina	528.6261	668.5978	-0.33889	0.38888	0.60729	-1.26478	FALSE
Spy0832	malP	825929	827260	443	malate-sod	286.2299	339.7505	-0.2473	0.51219	0.71479	-1.18698	FALSE
Spy0833	-	827291	828457	388	NAD-deper	201.6264	266.6742	-0.40339	0.3039	0.53505	-1.32261	FALSE
Spy0834	-	828540	829631	363	Zn-depend	671.2812	1054.473	-0.65153	0.09136	0.25738	-1.57083	FALSE
Spy0835	-	829825	830187	120	class B acid	1723.591	4285.641	-1.3141	0.10944	0.29142	-2.48647	FALSE
Spy0836	-	830130	830555	141	acid phospl	NA	NA	NA	NA	NA	NA	Na
Spy0837	-	830733	832265	510	chloride ch	1053.567	783.4327	0.4274	0.22471	0.44991	1.344808	FALSE

Spy0838	-	832428	833042	204	lipase/acylh	1028.516	712.5675	0.52947	0.12429	0.31056	1.443399	FALSE
Spy0839	-	833222	834349	375	hypothetica	4786.644	4509.481	0.086053	0.73239	0.8602	1.061462	FALSE
Spy0840	radC	834398	835078	226	DNA repair	31.43314	37.26266	-0.24544	0.70194	0.84791	-1.18545	FALSE
Spy0841	-	835080	835775	231	glutamine a	2972.761	1943.057	0.61348	0.062696	0.20262	1.529945	FALSE
Spy0842	-	835785	836429	214	redox-sens	7232.481	5300.629	0.44833	0.14822	0.34617	1.36446	FALSE
Spy0843	-	836681	837028	115	hypothetica	1362.9	995.9769	0.4525	0.18418	0.3943	1.368409	FALSE
Spy0844	nifS2	837018	838145	375	cysteine de	6397.166	3886.23	0.71906	0.034332	0.13525	1.646109	FALSE
Spy0845	-	838142	839122	326	ribose-phos	5427.121	3149.626	0.78501	0.018692	0.089723	1.723104	FALSE
Spy0846	-	839262	839840	192	adenylate c	559.917	480.0931	0.2219	0.47001	0.68695	1.166269	FALSE
Spy0847	-	839928	840599	223	GTP pyroph	2038.792	1214.234	0.74767	0.032178	0.1316	1.679079	FALSE
Spy0848	ppnK	840574	841410	278	inorganic p	2067.292	1290.07	0.68029	0.052397	0.18027	1.602462	FALSE
Spy0849	-	841407	842312	301	ribosomal l	2102.96	1458.639	0.5278	0.13686	0.32975	1.441729	FALSE
Spy0850	pta	842316	842492	58	phosphotra	NA	NA	NA	NA	NA	NA	Na
Spy0851	-	842489	843310	273	phosphotra	21733.44	12411.21	0.80827	0.014147	0.075452	1.75111	FALSE
Spy0852	-	843437	844054	205	short chain	NA	NA	NA	NA	NA	NA	Na
Spy0853	-	844051	844209	52	short chain	NA	NA	NA	NA	NA	NA	Na
Spy0854	-	844403	845092	229	Na+ driven	5703.544	989.4672	2.5271	5.15E-12	1.88E-09	5.764119	TRUE
Spy0855	proV	845512	846240	242	glycine beta	4705.571	2654.699	0.82582	0.01525	0.078922	1.772542	FALSE
Spy0856	-	846233	847759	508	glycine beta	11965.46	6371.202	0.90924	0.006788	0.045353	1.878056	TRUE
Spy0857	guaC	848037	849020	327	guanosine !	1218.442	1089.42	0.16148	0.67738	0.83426	1.118434	FALSE
Spy0858	xpt	849325	849906	193	xanthine ph	200.9511	674.4408	-1.7468	2.15E-05	0.000738	-3.35613	TRUE
Spy0859	-	849906	851189	427	xanthine pe	955.6979	3276.941	-1.7777	7.53E-07	5.73E-05	-3.42879	TRUE
Spy0860	apbE	851253	852191	312	thiamine bi	2030.979	1756.97	0.20909	0.48094	0.69291	1.155959	FALSE
Spy0861	-	852244	852429	61	4-oxalocrot	181.3791	169.3768	0.098772	0.75589	0.87235	1.070862	FALSE
Spy0862	tdk2	852567	853136	189	thymidine k	1107.532	632.9666	0.80715	0.035063	0.13724	1.749751	FALSE
Spy0863	prfA	853171	854250	359	peptide cha	3114.549	1048.919	1.5701	2.12E-05	0.000738	2.969253	TRUE
Spy0864	hemK	854250	855089	279	peptide rel	2977.151	1091.032	1.4482	8.63E-05	0.002187	2.728674	TRUE
Spy0865	-	855073	855663	196	SUA5 prote	2717.132	1046.25	1.3769	0.000179	0.003609	2.597097	TRUE
Spy0866	-	855681	856133	150	phosphinot	2299.159	881.5151	1.383	0.000185	0.00362	2.608101	TRUE
Spy0867	glyA	856123	857379	418	serine hydr	11417.23	4680.325	1.2865	0.000324	0.005179	2.439355	TRUE
Spy0868	-	857386	858363	325	hypothetica	9421.77	3349.993	1.4918	3.61E-05	0.001046	2.812396	TRUE
Spy0869	-	858364	858963	199	hypothetica	4824.802	1829.912	1.3987	0.000117	0.002734	2.636639	TRUE
Spy0870	-	858973	860697	574	multidrug r	14032.15	4883.177	1.5228	1.65E-05	0.00064	2.873482	TRUE
Spy0871	-	860694	862421	575	multidrug r	13251.76	4816.934	1.46	3.34E-05	0.000999	2.751084	TRUE
Spy0872	nox	862962	864032	356	NADH oxida	NA	NA	NA	NA	NA	NA	Na
Spy0873	ldh	864191	865174	327	L-lactate de	20639.35	15060.74	0.4546	0.17452	0.37951	1.370403	FALSE
Spy0874	gyrA	865365	867851	828	DNA gyrase	12928.41	7815.507	0.72613	0.029872	0.12383	1.654196	FALSE
Spy0875	-	867871	868620	249	sortase	4679.258	3229.218	0.53509	0.093422	0.26055	1.449033	FALSE

Spy0876	-	868700	869116	138	lactoylgluta	570.672	417.7942	0.44987	0.21452	0.43586	1.365917	FALSE
Spy0877	-	869603	869722	39	hypothetica	1540.954	1592.475	-0.04745	0.98496	1	-1.03343	FALSE
Spy0878	-	869730	870098	122	hypothetica	22892.67	21761.11	0.073134	0.77193	0.88331	1.051999	FALSE
Spy0879	-	870148	871059	303	hypothetica	4898.781	6911.448	-0.49657	0.15396	0.35269	-1.41086	FALSE
Spy0880	-	871176	871826	216	hypothetica	1638.997	2587.774	-0.6589	0.059383	0.19622	-1.57888	FALSE
Spy0881	-	871823	872263	146	hypothetica	373.8833	551.3056	-0.56026	0.18053	0.38878	-1.47453	FALSE
Spy0882	rbgA	872482	873330	282	ribosomal b	2434.256	2778.587	-0.19087	0.66522	0.8271	-1.14145	FALSE
Spy0883	rnhB	873320	874111	263	ribonucleas	2306.131	2672.767	-0.21286	0.47241	0.68695	-1.15898	FALSE
Spy0884	smf	874176	875012	278	hypothetica	110.626	512.7165	-2.2125	0.14947	0.34724	-4.63478	FALSE
Spy0885	-	875119	877248	709	DNA topois	4322.522	7732.577	-0.83908	0.018478	0.088929	-1.78891	FALSE
Spy0886	-	877323	877805	160	LysR family	26.80832	31.1137	-0.21487	0.82314	0.91865	-1.1606	FALSE
Spy0887	-	877812	877934	40	LysR family	1.330273	1.83649	-0.46523	0.96563	0.99479	-1.38054	FALSE
Spy0888	-	878080	878205	41	LysR family	13.89275	23.28521	-0.74508	0.55921	0.75055	-1.67607	FALSE
Spy0889	-	878218	878763	181	regulatory	30.49982	145.1203	-2.2504	0.15182	0.34964	-4.75815	FALSE
Spy0890	ddh	878778	879770	330	D-lactate d	52.50734	257.926	-2.2964	0.15546	0.35311	-4.9123	FALSE
Spy0891	satD	879875	880540	221	hypothetica	159.6097	269.2981	-0.75466	0.12745	0.31447	-1.68723	FALSE
Spy0892	satE	880533	881249	238	hypothetica	368.1861	488.8916	-0.40908	0.3424	0.56469	-1.32784	FALSE
Spy0893	gid	881400	882746	448	tRNA (uraci	2034.336	1796.591	0.1793	0.57548	0.76618	1.132334	FALSE
Spy0894	oadA2	882899	884305	468	oxaloacetat	212.5951	314.7744	-0.56621	0.21684	0.43947	-1.48063	FALSE
Spy0895	-	884348	884662	104	hypothetica	13.63522	23.84621	-0.80642	0.47697	0.68865	-1.74887	FALSE
Spy0896	-	884662	885012	116	biotin carbo	19.06081	37.26223	-0.9671	0.31242	0.54014	-1.95491	FALSE
Spy0897	-	885022	886152	376	oxaloacetat	260.9201	443.9273	-0.76672	0.070406	0.21729	-1.7014	FALSE
Spy0898	-	886352	887236	294	2-(5\'\'-trip	649.7297	862.2799	-0.40832	0.27078	0.50296	-1.32714	FALSE
Spy0899	citG	887229	887924	231	GntR family	406.5342	649.4717	-0.67589	0.082787	0.23969	-1.59758	FALSE
Spy0900	-	888082	889488	468	Mg2+/citra	198.6909	1529.875	-2.9448	1.17E-13	5.36E-11	-7.69969	TRUE
Spy0901	-	889865	890188	107	hypothetica	25.58649	63.04405	-1.301	0.09857	0.27032	-2.464	FALSE
Spy0902	-	890242	890640	132	acetyl-CoA	48.69877	96.17599	-0.98179	0.11581	0.30267	-1.97491	FALSE
Spy0903	oadB	890685	891806	373	oxaloacetat	341.5539	541.9122	-0.66595	0.09981	0.27213	-1.58661	FALSE
Spy0904	-	891824	891958	44	hypothetica	NA	NA	NA	NA	NA	NA	Na
Spy0905	citD	891994	892302	102	citrate lyas	92.43058	147.0795	-0.67015	0.22251	0.44747	-1.59124	FALSE
Spy0906	citE	892320	893177	285	citrate lyas	481.9649	674.7604	-0.48545	0.21263	0.43443	-1.40002	FALSE
Spy0907	citF	893180	894712	510	citrate lyas	865.0343	1547.626	-0.83923	0.025274	0.11055	-1.7891	FALSE
Spy0908	citX	894642	895283	213	2-(5\'\'-trip	221.8169	453.4659	-1.0316	0.016758	0.083063	-2.04429	FALSE
Spy0909	oadA1	895298	896692	464	oxaloacetat	951.9071	1888.184	-0.98811	0.028384	0.11902	-1.98358	FALSE
Spy0910	citC	896802	897854	350	(citrate (pro	4615	13978.23	-1.5988	3.71E-06	0.000205	-3.02891	TRUE
Spy0911	-	897943	898425	160	hypothetica	1333.94	5247.224	-1.9759	2.73E-08	4.14E-06	-3.93374	TRUE
Spy0912	-	898910	899014	34	hypothetica	NA	NA	NA	NA	NA	NA	Na
Spy0913	xerS	899125	900039	304	site-specifi	2420.562	11918.61	-2.2998	9.10E-11	2.37E-08	-4.9239	TRUE

Spy0914	-	900733	901362	209	phage trans	335.8469	2476.066	-2.8822	9.00E-14	5.36E-11	-7.37274	TRUE
Spy0915	ffh	901479	903041	520	signal recog	5388.323	6563.751	-0.28468	0.44117	0.65862	-1.21814	FALSE
Spy0916	ylxM	903056	903397	113	DNA-bindir	358.1379	524.2435	-0.54972	0.18107	0.38947	-1.4638	FALSE
Spy0917	-	903486	904184	232	GntR family	1374.1	2294.328	-0.73959	0.03586	0.14006	-1.6697	FALSE
Spy0918	-	904267	905700	477	hypothetica	424.1662	731.5687	-0.78636	0.044612	0.16145	-1.72472	FALSE
Spy0919	guaA	905867	907429	520	GMP synth	6662.338	5968.102	0.15876	0.57304	0.76406	1.116327	FALSE
Spy0920	-	907471	908694	407	UDP-N-acet	136.0561	764.8338	-2.4909	3.98E-05	0.001099	-5.62129	TRUE
Spy0921	-	909059	910603	514	ABC transp	512.1825	1978.529	-1.9497	1.74E-07	2.27E-05	-3.86294	TRUE
Spy0922	pdxK	910737	911294	185	hypothetica	72.83397	246.9353	-1.7614	0.008101	0.049748	-3.39027	TRUE
Spy0923	-	911272	912138	288	pyridoxami	229.4528	744.243	-1.6976	2.44E-05	0.000808	-3.24361	TRUE
Spy0924	-	912228	913496	422	GntR family	2874.682	803.9801	1.8382	2.61E-07	2.64E-05	3.575636	TRUE
Spy0925	rnhB	913894	914250	118	anaerobic r	5721.792	11723.72	-1.0349	0.003196	0.026859	-2.04897	TRUE
Spy0926	-	914572	916149	525	cardiolipin	9366.63	5577.008	0.74804	0.028308	0.11897	1.67951	FALSE
Spy0927	fhs.1	916234	917904	556	formate--te	10561.8	5803.56	0.86385	0.014553	0.076735	1.819888	FALSE
Spy0928	lplA	918032	919051	339	lipoate-pro	6586.1	3390.163	0.95807	0.007554	0.048517	1.942709	TRUE
Spy0929	-	919098	919979	293	SIR2 family	4660.64	2221.673	1.0689	0.002538	0.022579	2.097833	TRUE
Spy0930	-	919972	920784	270	hypothetica	4908.688	2503.965	0.97112	0.007227	0.047758	1.960362	TRUE
Spy0931	-	920777	921109	110	glycine clea	1479.708	595.3545	1.3135	0.000396	0.005964	2.485438	TRUE
Spy0932	-	921151	922149	332	luciferase-l	7088.224	2948.062	1.2657	0.000297	0.00497	2.404438	TRUE
Spy0933	-	922146	923345	399	NADH-depe	8411.474	3460.525	1.2814	0.000244	0.004452	2.430747	TRUE
Spy0934	-	923342	924178	278	lipoate-pro	7526.788	2979.759	1.3368	0.000125	0.002847	2.525904	TRUE
Spy0935	dpfB	924374	925066	230	phosphopa	937.2633	669.8504	0.48462	0.21388	0.43539	1.399217	FALSE
Spy0936	dfp	925059	925604	181	phosphopa	847.9626	631.6404	0.4249	0.26748	0.49887	1.342479	FALSE
Spy0937	-	925662	926231	189	transporter	2054.219	1864.587	0.13973	0.65386	0.81947	1.101699	FALSE
Spy0938	pgmA	926407	928125	572	phosphoglu	9418.044	8812.723	0.09584	0.74096	0.86414	1.068687	FALSE
Spy0939	-	928338	929294	318	nucleoside	4423.081	4862.748	-0.13672	0.78532	0.89303	-1.0994	FALSE
Spy0940	-	929296	930360	354	nucleoside	4069.745	3584.3	0.18325	0.53322	0.73072	1.135439	FALSE
Spy0941	-	930353	931903	516	nucleoside	5837.072	3747.439	0.63934	0.0519	0.17895	1.557616	FALSE
Spy0942	-	932024	933076	350	nucleoside-	15037.85	12437.75	0.27387	0.36733	0.59293	1.209047	FALSE
Spy0943	cdd	933170	933559	129	cytidine de	7754.28	5731.893	0.43598	0.16371	0.36372	1.352829	FALSE
Spy0944	-	934218	934805	195	16S rRNA m	3756.617	2472.398	0.60352	0.062008	0.20197	1.519419	FALSE
Spy0945	coaA	935073	935993	306	pantothena	432.8211	394.7623	0.13279	0.62337	0.80469	1.096412	FALSE
Spy0946	rpsT	936062	936295	77	30S ribosor	2346.525	5970.953	-1.3474	0.024799	0.10899	-2.54453	FALSE
Spy0947	ciaH	936420	937730	436	sensor prot	6554.542	2780.079	1.2374	0.000226	0.004192	2.357732	TRUE
Spy0948	ciaR	937723	938397	224	transcriptic	3167.869	1124.366	1.4944	1.76E-05	0.000656	2.81747	TRUE
Spy0949	pepN	938743	941280	845	lysyl amino	29639.03	22220.01	0.41564	0.17348	0.37805	1.33389	FALSE
Spy0950	phoU	941485	942138	217	phosphate	4368.092	2512.537	0.79786	0.017656	0.086109	1.73852	FALSE
Spy0951	pstB	942206	942964	252	phosphate	5943.235	3332.521	0.83463	0.013403	0.073197	1.7834	FALSE

Spy0952	pstB2	942977	943780	267	phosphate	6558.704	3263.864	1.0068	0.003384	0.02743	2.009449	TRUE
Spy0953	pstA	943796	944683	295	phosphate	6351.774	3331.576	0.93095	0.00666	0.045162	1.906531	TRUE
Spy0954	pstC	944673	945491	272	phosphate	5921.326	3323.165	0.83336	0.016212	0.080796	1.78183	FALSE
Spy0955	pstS	945618	946484	288	phosphate-	4707.106	2249.92	1.065	0.002752	0.023901	2.09217	TRUE
Spy0956	-	946623	947933	436	16S rRNA m	2871.924	1653.855	0.79618	0.029211	0.12192	1.736497	FALSE
Spy0957	-	947936	948724	262	myo-inosito	1496.733	1059.722	0.49813	0.1764	0.38258	1.412382	FALSE
Spy0958	-	948714	948992	92	hypothetica	65.17957	41.57276	0.64878	0.36881	0.59458	1.567842	FALSE
Spy0959	spxA	948994	949398	134	Spx family t	3785.836	6185.514	-0.70828	0.2891	0.51997	-1.63386	FALSE
Spy0960	mreA	949441	950373	310	bifunctiona	3607.475	1464.979	1.3001	0.000322	0.005179	2.46246	TRUE
Spy0961	truB	950402	951286	294	tRNA pseud	3407.847	1417.496	1.2655	0.000424	0.006243	2.404105	TRUE
Spy0962	-	951402	952742	446	hypothetica	842.1462	594.2297	0.50305	0.28254	0.51433	1.417207	FALSE
Spy0963	-	952839	953795	318	hypothetica	888.6031	2608.548	-1.5536	9.20E-05	0.002299	-2.93549	TRUE
Spy0964	-	953806	954402	198	type I restri	464.9749	1246.736	-1.4229	0.003682	0.02901	-2.68124	TRUE
Spy0965	-	954494	955669	391	ABC transp	1576.164	2422.189	-0.61989	0.24812	0.48044	-1.53676	FALSE
Spy0966	-	955689	957131	480	ABC transp	NA	NA	NA	NA	NA	NA	Na
Spy0967	-	957146	957847	233	ABC transp	273.1653	292.8689	-0.10048	0.80189	0.90399	-1.07213	FALSE
Spy0968	-	957965	958507	180	TetR family	56.23027	90.61874	-0.68846	0.28201	0.51387	-1.61156	FALSE
Spy0969	-	958504	958647	47	hypothetica	NA	NA	NA	NA	NA	NA	Na
Spy0970	-	958650	959291	213	NAD-deper	10388.37	8300.481	0.3237	0.32887	0.55129	1.251536	FALSE
Spy0971	-	959454	959942	162	Gls24 famil	26822.69	21888.41	0.29329	0.37984	0.60098	1.225432	FALSE
Spy0972	-	959953	960153	66	hypothetica	1679.309	1235.246	0.44307	0.22043	0.44477	1.359494	FALSE
Spy0973	-	960194	960733	179	Gls24 famil	25165.73	17414.16	0.5312	0.12391	0.31056	1.445131	FALSE
Spy0974	-	960746	960934	62	small integ	268.2192	171.721	0.64335	0.15338	0.3519	1.561952	FALSE
Spy0975	-	960945	961532	195	hypothetica	26921.48	15405.7	0.80529	0.015636	0.079555	1.747497	FALSE
Spy0976	-	961593	961799	68	hypothetica	13787.08	9084.433	0.60185	0.061114	0.19977	1.517661	FALSE
Spy0977	pcrA	962205	964523	772	DNA helica	10862.23	9721.611	0.16005	0.62776	0.80822	1.117326	FALSE
Spy0978	-	965052	966374	440	Na(+)-linke	1013.486	3964.27	-1.9677	0.015049	0.07865	-3.91144	FALSE
Spy0979	-	964891	965061	56	hypothetica	NA	NA	NA	NA	NA	NA	Na
Spy0980	-	966494	967729	411	cobalt-zinc-	4254.403	5068.9	-0.25272	0.4771	0.68865	-1.19145	FALSE
Spy0981	cfa	968098	968871	257	cAMP facto	7532.074	6268.677	0.26489	0.41342	0.63102	1.201544	FALSE
Spy0982	-	969241	970077	278	histidine-bi	1978.074	2935.448	-0.56948	0.28033	0.51349	-1.48399	FALSE
Spy0983	-	970093	970722	209	histidine tra	1066.629	1504.088	-0.49583	0.243	0.47557	-1.41013	FALSE
Spy0984	-	970732	971373	213	histidine tra	1152.574	2425.634	-1.0735	0.10244	0.27805	-2.10453	FALSE
Spy0985	-	971479	971814	111	phnA prote	522.5492	1705.88	-1.7069	0.015694	0.079555	-3.26459	FALSE
Spy0986	glmS	972010	973824	604	glucosamin	9974.483	34481.07	-1.7895	3.68E-07	3.53E-05	-3.45695	TRUE
Spy0987	sipC	974000	974557	185	signal pepti	4031.892	5154.806	-0.35446	0.36993	0.59458	-1.27851	FALSE
Spy0988	pyk	974775	976277	500	pyruvate ki	91686.17	51158.9	0.84172	0.011521	0.064858	1.792186	FALSE
Spy0989	pfkA	976340	977353	337	6-phosphof	41826	19950.4	1.068	0.001467	0.015207	2.096525	TRUE

Spy0990	dnaE	977433	980543	1036	DNA polym	9863.519	4446.323	1.1495	0.001433	0.01502	2.21837	TRUE
Spy0991	-	980728	981099	123	GntR family	1636.617	1368.397	0.25823	0.40315	0.62019	1.19601	FALSE
Spy0992	-	981099	981797	232	ABC transp	5591.998	3646.464	0.61687	0.049824	0.17404	1.533544	FALSE
Spy0993	-	981807	982592	261	ABC transp	11145.93	9171.163	0.28134	0.32463	0.54887	1.215323	FALSE
Spy0994	-	982719	983333	204	membrane	3380.028	4919.467	-0.54147	0.46472	0.68354	-1.45545	FALSE
Spy0995	-	983924	984112	62	phage prot	9.847702	3.41754	1.5268	0.49471	0.7022	2.88146	FALSE
Spy0996	speA2	984332	985087	251	enterotoxin	326.1278	863.8996	-1.4054	0.069384	0.21593	-2.64891	FALSE
Spy0997	-	985209	985868	219	phage prot	35.33876	17.82581	0.98728	0.32012	0.54758	1.982444	FALSE
Spy0998	-	985868	986089	73	phage prot	0.391833	0.943505	-1.2678	0.94577	0.98689	-2.40794	FALSE
Spy0999	-	986099	986872	257	phage prot	34.42838	10.25226	1.7477	0.11106	0.29443	3.358228	FALSE
Spy1000	-	986883	987485	200	phage prot	17.40097	7.192116	1.2747	0.39466	0.61212	2.419485	FALSE
Spy1001	-	987497	988261	254	phage-asso	68.07381	26.34572	1.3695	0.092066	0.25794	2.58381	FALSE
Spy1002	-	988263	988595	110	N-acetylmu	3.978834	4.0809	-0.03654	0.99194	1	-1.02565	FALSE
Spy1003	-	988595	988963	122	phage prot	14.71821	4.616486	1.6727	0.35764	0.58245	3.188107	FALSE
Spy1004	-	988932	989096	54	phage prot	NA	NA	NA	NA	NA	NA	Na
Spy1005	-	989068	989415	115	phage prot	36.44688	11.6295	1.648	0.11236	0.29626	3.133989	FALSE
Spy1006	-	989426	991177	583	phage struc	118.2585	54.1695	1.1264	0.064105	0.20586	2.183133	FALSE
Spy1007	-	991293	994733	1146	phage prot	278.6353	97.34959	1.5171	0.001973	0.018842	2.862151	TRUE
Spy1008	-	994734	996218	494	hypothetica	124.7856	36.47182	1.7746	0.006159	0.042343	3.421431	TRUE
Spy1009	-	996219	998024	601	phage prot	124.5382	54.93651	1.1808	0.063427	0.20404	2.267025	FALSE
Spy1010	-	998017	998475	152	phage prot	16.6727	6.044352	1.4638	0.37523	0.59906	2.758339	FALSE
Spy1011	-	998448	998765	105	phage prot	4.270116	2.397187	0.83293	0.82396	0.91865	1.781299	FALSE
Spy1012	-	998778	999284	168	antigen A	25.93725	13.49161	0.94296	0.47392	0.68715	1.922469	FALSE
Spy1013	-	999296	999706	136	antigen B	15.38758	4.055234	1.9239	0.28697	0.51984	3.794474	FALSE
Spy1014	-	999708	1000103	131	antigen C	11.25105	5.27929	1.0916	0.58057	0.76846	2.131103	FALSE
Spy1015	-	1000100	1000411	103	phage prot	6.434329	1.734233	1.8915	0.53958	0.73641	3.710208	FALSE
Spy1016	-	1000408	1000752	114	phage prot	8.650571	4.234083	1.0307	0.71569	0.85433	2.043015	FALSE
Spy1017	-	1000766	1001059	97	phage prot	7.552683	2.091376	1.8525	0.47828	0.68963	3.611254	FALSE
Spy1018	-	1001072	1001962	296	phage prot	67.70024	20.73378	1.7072	0.030884	0.12716	3.265265	FALSE
Spy1019	-	1001981	1002550	189	phage scaff	46.68027	20.58239	1.1814	0.24241	0.47544	2.267968	FALSE
Spy1020	-	1002795	1003064	89	phage prot	14.15274	8.237835	0.78074	0.72695	0.85822	1.718012	FALSE
Spy1021	-	1003071	1003979	302	phage prot	41.34588	18.9748	1.1237	0.22403	0.44933	2.179051	FALSE
Spy1022	-	1003948	1005273	441	portal prot	75.24197	22.80092	1.7224	0.028115	0.11844	3.299849	FALSE
Spy1023	-	1005273	1006547	424	terminase l	57.67307	31.06487	0.89261	0.27575	0.50819	1.856532	FALSE
Spy1024	-	1006537	1006917	126	phage prot	20.99601	13.90013	0.59502	0.65034	0.81921	1.510494	FALSE
Spy1025	-	1007527	1007961	144	ArpU family	71.9502	87.93735	-0.28948	0.6554	0.82048	-1.2222	FALSE
Spy1026	-	1008247	1008513	88	phage prot	42.54594	25.09615	0.76156	0.39321	0.61092	1.695323	FALSE
Spy1027	-	1008510	1009034	174	phage prot	44.31993	21.19339	1.0643	0.23865	0.46907	2.091155	FALSE

Spy1028	-	1009037	1009669	210	phage prot	0	0	NA	NA	NA	NA	NA
Spy1029	-	1009671	1009955	94	phage prot	39.68151	17.24078	1.2026	0.23469	0.46581	2.301541	FALSE
Spy1030	-	1009952	1010146	64	phage prot	NA	NA	NA	NA	NA	NA	Na
Spy1031	-	1010119	1010358	79	phage prot	26.32222	17.85321	0.5601	0.66892	0.82936	1.474371	FALSE
Spy1032	-	1010355	1010600	81	phage prot	1.684983	2.703404	-0.68204	0.88595	0.95435	-1.60441	FALSE
Spy1033	-	1010597	1010953	118	phage prot	15.04603	7.906658	0.92824	0.57727	0.76745	1.902953	FALSE
Spy1034	-	1010950	1011390	146	phage prot	25.0371	16.47556	0.60374	0.67656	0.83385	1.519651	FALSE
Spy1035	-	1011390	1011593	67	phage prot	1.622722	0	Inf	0.71529	0.85433	NA	FALSE
Spy1036	ssb2	1011599	1012024	141	phage singl	3.773783	4.080643	-0.11279	0.98793	1	-1.08132	FALSE
Spy1037	ssb1	1012017	1012691	224	phage singl	87.01185	29.73881	1.5489	0.036481	0.14158	2.92594	FALSE
Spy1038	-	1012692	1013174	160	phage prot	29.33747	17.03656	0.78411	0.49041	0.70006	1.72203	FALSE
Spy1039	-	1013196	1013450	84	phage prot	4.128493	3.315582	0.31635	0.9376	0.98523	1.245176	FALSE
Spy1040	-	1013431	1013784	117	phage prot	50.57682	24.96833	1.0184	0.26333	0.49474	2.025671	FALSE
Spy1041	-	1013797	1013934	45	phage prot	NA	NA	NA	NA	NA	NA	Na
Spy1042	-	1013925	1014707	260	phage repli	110.9818	57.41084	0.95093	0.13774	0.3306	1.933118	FALSE
Spy1043	-	1014694	1015524	276	phage prot	71.63392	44.35337	0.6916	0.33713	0.55903	1.615074	FALSE
Spy1044	-	1015738	1016199	153	phage prot	32.42814	109.1075	-1.7504	0.00611	0.042249	-3.36452	TRUE
Spy1045	-	1016649	1016849	66	transcriptio	65.72954	64.42464	0.028929	0.95356	0.98992	1.020254	FALSE
Spy1046	-	1016923	1017309	128	phage prot	1538.396	3052.627	-0.98862	0.11785	0.30402	-1.98429	FALSE
Spy1047	-	1017298	1017507	69	phage prot	104.7446	179.5773	-0.77773	0.13585	0.32821	-1.71443	FALSE
Spy1048	-	1017561	1018160	199	phage prot	776.1391	775.3095	0.001543	0.92378	0.97963	1.00107	FALSE
Spy1049	-	1018190	1018348	52	phage prot	81.16974	122.3683	-0.59222	0.27694	0.50922	-1.50756	FALSE
Spy1050	-	1018705	1019529	274	phage trans	1498.15	1753.373	-0.22695	0.51981	0.72102	-1.17036	FALSE
Spy1051	-	1019565	1020458	297	phage prot	1814.035	2120.251	-0.22503	0.5296	0.72873	-1.1688	FALSE
Spy1052	int.1	1020579	1021667	362	integrase	621.0729	1191.005	-0.93935	0.082447	0.23908	-1.91766	FALSE
Spy1053	-	1021799	1021912	37	hypothetica	NA	NA	NA	NA	NA	NA	Na
Spy1054	-	1022030	1022650	206	hypothetica	393.5702	392.1283	0.005295	0.86944	0.94509	1.003677	FALSE
Spy1055	glgP	1022907	1025171	754	glycogen pl	1955.025	1517.775	0.36523	0.26874	0.50019	1.288087	FALSE
Spy1056	malM	1025206	1026699	497	4-alpha-glu	619.1257	565.3804	0.13101	0.69475	0.8446	1.09506	FALSE
Spy1057	malR	1026814	1027833	339	LacI family	1526.431	1637.628	-0.10145	0.9567	0.99149	-1.07285	FALSE
Spy1058	malE	1027874	1029325	483	maltose/ma	2523.072	3711.709	-0.5569	0.30507	0.53659	-1.4711	FALSE
Spy1059	malF	1029830	1030960	376	maltose AB	1261.695	1462.586	-0.21316	0.72792	0.85842	-1.15922	FALSE
Spy1060	malG	1030960	1031796	278	maltose AB	694.6547	801.9281	-0.20718	0.7352	0.86073	-1.15443	FALSE
Spy1061	-	1031951	1032751	266	LacI family	620.4129	633.6062	-0.03036	0.92719	0.98183	-1.02127	FALSE
Spy1062	malA	1032867	1033682	271	maltodextr	7208.263	19676.18	-1.4487	0.026676	0.11395	-2.72962	FALSE
Spy1063	malD	1033704	1034564	286	maltodextr	6836.378	15392.21	-1.1709	0.097534	0.26833	-2.25152	FALSE
Spy1064	malC	1034561	1035868	435	maltose AB	9780.865	21702.46	-1.1498	0.071936	0.22053	-2.21883	FALSE
Spy1065	amyA	1035943	1038078	711	alpha-amyl	22593.05	48455.06	-1.1008	0.068328	0.21488	-2.14474	FALSE

Spy1066	amyB	1038104	1039807	567	neopullular	13908.04	25625.05	-0.88164	0.12245	0.30888	-1.84247	FALSE
Spy1067	malX	1039973	1041232	419	maltose/m	14655.78	21392	-0.5456	0.33244	0.55427	-1.45963	FALSE
Spy1068	-	1041558	1041782	74	transposase	9.513013	5.075737	0.90629	0.68611	0.84104	1.87422	FALSE
Spy1069	-	1041847	1042830	327	esterase	20659.49	14397.25	0.52101	0.12259	0.30888	1.434959	FALSE
Spy1070	dltD	1042861	1044111	416	hypothetica	15384.04	10270.09	0.58299	0.093687	0.26089	1.497951	FALSE
Spy1071	-	1044104	1044343	79	D-alanine--	478.6832	351.9863	0.44355	0.33785	0.55964	1.359947	FALSE
Spy1072	-	1044361	1045617	418	protein Dlt	18682.9	12480.08	0.58209	0.097265	0.26833	1.497016	FALSE
Spy1073	dltA	1045614	1047152	512	D-alanine--	20632.9	14017.8	0.55769	0.099635	0.27206	1.471911	FALSE
Spy1074	-	1047164	1047307	47	hypothetica	400.653	236.8983	0.75809	0.13262	0.32254	1.69125	FALSE
Spy1075	uvrB	1047571	1049562	663	excinucleas	12051.25	8448.718	0.51238	0.12759	0.31447	1.426401	FALSE
Spy1076	glnH	1049755	1051929	724	transporter	24091.39	7281.102	1.7263	3.94E-07	3.59E-05	3.308781	TRUE
Spy1077	glnQ.2	1051929	1052669	246	glutamine A	11551.22	5048.25	1.1942	0.00037	0.005665	2.288179	TRUE
Spy1078	-	1052817	1052969	50	hypothetica	NA	NA	NA	NA	NA	NA	Na
Spy1079	-	1052966	1054345	459	PTS system	502.6046	884.6691	-0.81571	0.033042	0.13248	-1.76016	FALSE
Spy1080	-	1054523	1054972	149	hypothetica	100.2031	199.8248	-0.99581	0.049347	0.17375	-1.9942	FALSE
Spy1081	-	1054969	1055304	111	PTS system	25.57464	40.70514	-0.6705	0.39949	0.617	-1.59162	FALSE
Spy1082	-	1055307	1055618	103	PTS system	23.69879	44.37943	-0.90508	0.31788	0.54596	-1.87265	FALSE
Spy1083	-	1055641	1057635	664	PTS system	367.1597	814.4092	-1.1493	0.002967	0.025409	-2.21806	TRUE
Spy1084	-	1057741	1058835	364	outer surfa	152.0086	340.1263	-1.1619	0.007959	0.04938	-2.23752	TRUE
Spy1085	bglA.2	1058844	1060244	466	beta-glucos	226.8261	401.6928	-0.82451	0.043457	0.15973	-1.77093	FALSE
Spy1086	-	1060469	1061164	231	nicotinamid	529.972	1367.642	-1.3677	0.000307	0.005041	-2.58059	TRUE
Spy1087	-	1061169	1061327	52	hypothetica	29.21412	53.22809	-0.86552	0.25007	0.48267	-1.822	FALSE
Spy1088	obgE	1061396	1062703	435	GTPase Obg	3471.853	3775.368	-0.12091	0.73397	0.86046	-1.08742	FALSE
Spy1089	-	1062766	1062894	42	hypothetica	4.966383	14.74148	-1.5696	0.3221	0.54806	-2.96822	FALSE
Spy1090	-	1063136	1063651	171	transposase	NA	NA	NA	NA	NA	NA	Na
Spy1091	-	1063824	1064480	218	transposase	8.398609	9.436675	-0.16813	0.93677	0.98523	-1.1236	FALSE
Spy1092	rsuA	1064529	1065263	244	ribosomal s	927.1793	2067.138	-1.1567	0.001361	0.014628	-2.22947	TRUE
Spy1093	-	1065375	1065740	121	hypothetica	395.6308	1397.18	-1.8203	0.078558	0.23218	-3.53155	FALSE
Spy1094	-	1065860	1067080	406	major facili	495.2504	2613.316	-2.3997	0.054998	0.18681	-5.27693	FALSE
Spy1095	-	1067382	1068938	518	hypothetica	7370.184	6897.816	0.095561	0.68576	0.84104	1.068481	FALSE
Spy1096	-	1069045	1069446	133	thioesteras	1263.567	896.6354	0.49491	0.18786	0.39936	1.409233	FALSE
Spy1097	-	1069531	1070043	170	phosphoryl	NA	NA	NA	NA	NA	NA	Na
Spy1098	-	1070348	1071703	451	tRNA (uraci	86.15555	53.78827	0.67965	0.31164	0.53975	1.601751	FALSE
Spy1099	-	1071785	1073236	483	LytR family	5256.228	6901.747	-0.39293	0.23659	0.46652	-1.31306	FALSE
Spy1100	aroK	1073444	1073935	163	shikimate k	983.8348	958.6744	0.037375	0.95997	0.9922	1.026245	FALSE
Spy1101	-	1073928	1075220	430	3-phosphos	3287.699	2701.901	0.2831	0.72557	0.85822	1.216807	FALSE
Spy1102	-	1075322	1076287	321	ribonucleas	6386.897	5779.75	0.14411	0.70553	0.84999	1.105049	FALSE
Spy1103	map	1076289	1077149	286	methionine	6971.701	5451.093	0.35497	0.33512	0.5572	1.278959	FALSE

Spy1104	-	1077165	1078448	427	hypothetica	3295.883	3572.974	-0.11646	0.72074	0.85775	-1.08407	FALSE
Spy1105	-	1078457	1078999	180	ribosomal-p	1048.055	1470.57	-0.48866	0.33833	0.55964	-1.40314	FALSE
Spy1106	grab	1079237	1079890	217	protein G-r	5947.829	9825.206	-0.72412	0.033497	0.1337	-1.65189	FALSE
Spy1107	murZ	1080248	1081507	419	UDP-N-acet	15897.95	11083.26	0.52046	0.11623	0.30267	1.434413	FALSE
Spy1108	metK2	1081681	1082877	398	S-adenosyl	7359.663	5424.445	0.44016	0.19214	0.40469	1.356755	FALSE
Spy1109	inlA	1083414	1085792	792	internalin p	384.8863	578.4154	-0.58767	0.32648	0.55071	-1.50282	FALSE
Spy1110	birA	1085996	1086937	313	biotin--prot	3704.632	2431.323	0.60759	0.057476	0.1922	1.523712	FALSE
Spy1111	-	1086912	1087220	102	hypothetica	867.4601	988.0991	-0.18786	0.64186	0.81627	-1.13907	FALSE
Spy1112	-	1087210	1088880	556	DNA polym	8585.986	6455.456	0.41146	0.20954	0.43186	1.330031	FALSE
Spy1113	-	1088880	1089377	165	GAF domai	1471.186	1271.382	0.21058	0.54325	0.73672	1.157153	FALSE
Spy1114	-	1089522	1090331	269	hypothetica	700.1459	682.9106	0.035959	0.80598	0.90692	1.025238	FALSE
Spy1115	-	1090384	1090647	87	hypothetica	112.3633	137.6989	-0.29335	0.58032	0.76846	-1.22548	FALSE
Spy1116	udk	1090727	1091353	208	uridine kina	839.4882	1715.486	-1.031	0.044087	0.15987	-2.04344	FALSE
Spy1117	deaD2	1091451	1092536	361	ATP-depen	6484.405	5860.803	0.14588	0.59311	0.77839	1.106405	FALSE
Spy1118	-	1092700	1093920	406	peptidoglyc	5113.732	6533.015	-0.35337	0.47301	0.68695	-1.27754	FALSE
Spy1119	gapN	1094015	1095442	475	NADP-depe	24428.22	9566.048	1.3526	7.40E-05	0.001929	2.553719	TRUE
Spy1120	pstI	1095627	1097360	577	phosphoen	53447.26	40917.69	0.38539	0.20512	0.42613	1.306213	FALSE
Spy1121	ptsH	1097365	1097628	87	phosphoca	32950.35	38604.73	-0.22848	0.82824	0.92172	-1.1716	FALSE
Spy1122	nrdH	1098021	1098239	72	glutaredoxi	268.321	230.0499	0.22201	0.54064	0.73641	1.166357	FALSE
Spy1123	nrdE.2	1098259	1100418	719	ribonucleot	12194.29	8912.97	0.45223	0.16436	0.36472	1.368153	FALSE
Spy1124	nrdF	1100751	1101710	319	ribonucleot	4555.376	2747.224	0.7296	0.026669	0.11395	1.658179	FALSE
Spy1125	-	1101685	1102998	437	chloride ch	1229.394	1591.191	-0.37216	0.3054	0.53667	-1.29429	FALSE
Spy1126	-	1103214	1103429	71	transposase	NA	NA	NA	NA	NA	NA	Na
Spy1127	-	1103462	1103758	98	transposase	NA	NA	NA	NA	NA	NA	Na
Spy1128	-	1104071	1104292	73	transposase	NA	NA	NA	NA	NA	NA	Na
Spy1129	-	1104384	1105079	231	CAAX amin	370.9192	1027.285	-1.4697	0.000183	0.003619	-2.76964	TRUE
Spy1130	-	1105098	1105859	253	hypothetica	415.1512	1061.057	-1.3538	0.000543	0.007335	-2.55584	TRUE
Spy1131	-	1105856	1106077	73	Cro/CI fami	127.4124	425.129	-1.7384	0.055404	0.18714	-3.33665	FALSE
Spy1132	alaS	1106432	1109050	872	alanyl-tRNA	11631.71	7989.27	0.54193	0.12261	0.30888	1.455919	FALSE
Spy1133	prsA	1109437	1110492	351	foldase Prs	6345.76	3266.195	0.95818	0.004261	0.032383	1.942857	TRUE
Spy1134	-	1110555	1111262	235	O-methyltr	1084.492	505.3403	1.1017	0.003522	0.028178	2.146074	TRUE
Spy1135	-	1111328	1112524	398	oxalate/for	424.3325	2193.575	-2.37	0.000611	0.008019	-5.16941	TRUE
Spy1136	pepB	1112900	1114705	601	oligoendop	2427.765	7958.587	-1.7129	0.11647	0.30267	-3.27819	FALSE
Spy1137	-	1114718	1115680	320	competenc	738.9474	4182.143	-2.5007	0.069452	0.21593	-5.6596	FALSE
Spy1138	-	1115975	1116691	238	ribosomal s	1743.332	11462.86	-2.717	0.043706	0.15987	-6.57504	FALSE
Spy1139	nagB	1116810	1117514	234	glucosamin	964.9295	7206.538	-2.9008	0.033411	0.13364	-7.4684	FALSE
Spy1140	queA	1117716	1118744	342	S-adenosyl	1865.624	1278.409	0.54531	0.12481	0.31144	1.459334	FALSE
Spy1141	-	1118751	1119971	406	hypothetica	3607.487	2525.414	0.51447	0.12995	0.3173	1.428469	FALSE

Spy1142	-	1120085	1120675	196	hypothetica	1338.991	1655.712	-0.3063	0.43963	0.65728	-1.23653	FALSE
Spy1143	-	1120672	1120920	82	hypothetica	25.11543	37.26268	-0.56916	0.52359	0.72354	-1.48366	FALSE
Spy1144	-	1120905	1121132	75	hypothetica	110.0138	138.8213	-0.33554	0.55861	0.7503	-1.26185	FALSE
Spy1145	sodA	1121299	1121904	201	superoxide	44008.87	16836.82	1.3862	2.91E-05	0.000922	2.613893	TRUE
Spy1146	holA	1122001	1123041	346	DNA polym	1354.127	1017.811	0.41189	0.27694	0.50922	1.330428	FALSE
Spy1147	comEC	1123112	1125355	747	competenc	95.32131	174.6298	-0.87343	0.50115	0.70373	-1.83201	FALSE
Spy1148	-	1125336	1125998	220	competenc	19.60976	42.69434	-1.1225	0.48685	0.69703	-2.17724	FALSE
Spy1149	-	1126198	1126938	246	1-acyl-sn-gl	1577.247	1725.293	-0.12943	0.64984	0.81921	-1.09386	FALSE
Spy1150	-	1127056	1127832	258	methyltran	528.1598	829.6582	-0.65154	0.16261	0.36303	-1.57084	FALSE
Spy1151	-	1127822	1128100	92	hypothetica	50.52026	84.54639	-0.74288	0.24761	0.48044	-1.67351	FALSE
Spy1152	-	1128124	1129107	327	kup system	2425.906	5245.299	-1.1125	0.001012	0.011613	-2.1622	TRUE
Spy1153	-	1129059	1130123	354	kup system	NA	NA	NA	NA	NA	NA	Na
Spy1154	deaD	1130251	1131870	539	ATP-depen	27038.96	31082.91	-0.20108	0.59396	0.77885	-1.14956	FALSE
Spy1155	prfC	1132177	1133721	514	peptide cha	10442.66	14196.91	-0.44309	0.1637	0.36372	-1.35951	FALSE
Spy1156	-	1133969	1134664	231	hypothetica	4996.965	5072.155	-0.02155	0.92884	0.98271	-1.01505	FALSE
Spy1157	murF	1134744	1136135	463	UDP-N-ace	10999.49	6237.414	0.81842	0.017014	0.083883	1.763474	FALSE
Spy1158	ddl	1136326	1137372	348	D-alanyl-ala	3947.534	2978.078	0.40657	0.25508	0.4864	1.325531	FALSE
Spy1159	recR	1137473	1138069	198	recombinat	1184.234	709.0953	0.7399	0.050736	0.17647	1.67006	FALSE
Spy1160	-	1138116	1138307	63	penicillin-b	292.0623	228.4696	0.35427	0.44125	0.65862	1.278339	FALSE
Spy1161	-	1138868	1139647	259	formate tra	988.5434	1795.359	-0.8609	0.019473	0.092017	-1.81617	FALSE
Spy1162	-	1139771	1140313	180	hypothetica	1902.668	3988.476	-1.0678	0.006752	0.04535	-2.09623	TRUE
Spy1163	-	1140474	1140995	173	biotin repre	695.828	1098.729	-0.65903	0.081602	0.23701	-1.57902	FALSE
Spy1164	gpmA	1141102	1141797	231	phosphogly	8290.751	10471.57	-0.3369	0.47074	0.68695	-1.26304	FALSE
Spy1165	pyrD	1142036	1142971	311	dihydrooro	2887.909	2148.212	0.42689	0.17839	0.38598	1.344332	FALSE
Spy1166	-	1143026	1143199	57	hypothetica	NA	NA	NA	NA	NA	NA	Na
Spy1167	-	1143271	1145133	620	lead, cadmi	41401.26	65263.9	-0.65661	0.060701	0.19913	-1.57637	FALSE
Spy1168	-	1145664	1145846	60	phage prot	3.773783	5.151922	-0.4491	0.85528	0.93921	-1.36519	FALSE
Spy1169	spd3	1146085	1146885	266	streptodorn	1647.223	2913.146	-0.82254	0.024744	0.10899	-1.76852	FALSE
Spy1170	-	1147096	1147590	164	hypothetica	511.9646	1179.062	-1.2035	0.001925	0.01868	-2.30298	TRUE
Spy1171	-	1147660	1148865	401	phage-asso	39.80953	55.77773	-0.48658	0.47722	0.68865	-1.40112	FALSE
Spy1172	-	1148981	1149208	75	holin	0.696267	3.264506	-2.2292	0.63733	0.81579	-4.68874	FALSE
Spy1173	-	1149205	1149480	91	phage prot	0.391833	1.989674	-2.3442	0.76955	0.8828	-5.07779	FALSE
Spy1174	-	1149490	1150107	205	phage prot	5.720961	3.723501	0.6196	0.86693	0.94348	1.536449	FALSE
Spy1175	-	1150104	1150541	145	phage prot	1.989417	2.321001	-0.2224	0.97486	0.99967	-1.16667	FALSE
Spy1176	-	1150553	1152421	622	phage infec	20.51561	32.44114	-0.6611	0.44913	0.66658	-1.58129	FALSE
Spy1177	-	1152418	1153113	231	phage prot	3.114639	10.4057	-1.7402	0.37273	0.59584	-3.34081	FALSE
Spy1178	-	1153110	1155467	785	phage prot	33.34203	53.53508	-0.68314	0.38988	0.60781	-1.60563	FALSE
Spy1179	-	1155467	1155838	123	phage prot	0.913303	1.249722	-0.45244	1	1	-1.36835	FALSE

Spy1180	-	1155853	1156116	87	phage prot	3.351697	6.529268	-0.96203	0.61226	0.79599	-1.94805	FALSE
Spy1181	-	1156127	1156720	197	major tail p	11.33845	14.74184	-0.37869	0.76612	0.87998	-1.30016	FALSE
Spy1182	-	1156732	1157067	111	phage prot	0.304434	0	Inf	0.99805	1	NA	FALSE
Spy1183	-	1157068	1157304	78	phage prot	0	0.280551	#NAME?	1	1	NA	FALSE
Spy1184	-	1157297	1157635	112	phage prot	0	0.714286	#NAME?	0.93359	0.98399	NA	FALSE
Spy1185	-	1157595	1158017	140	phage prot	4.92926	5.049814	-0.03486	1	1	-1.02446	FALSE
Spy1186	-	1158027	1158227	66	phage prot	0	0.331477	#NAME?	1	1	NA	FALSE
Spy1187	-	1158227	1159138	303	phage struc	17.37072	20.42905	-0.23397	0.78892	0.89504	-1.17607	FALSE
Spy1188	-	1159163	1159624	153	phage prot	3.344828	5.381141	-0.68598	0.78904	0.89504	-1.60879	FALSE
Spy1189	-	1159705	1161120	471	phage term	30.1663	48.38215	-0.68154	0.50585	0.70791	-1.60385	FALSE
Spy1190	-	1161230	1161496	88	phage prot	0	0.637694	#NAME?	0.94016	0.98611	NA	FALSE
Spy1191	-	1161535	1161750	71	phage prot	NA	NA	NA	NA	NA	NA	Na
Spy1192	-	1161719	1161952	77	phage prot	35.22811	68.37598	-0.95676	0.19118	0.40361	-1.94095	FALSE
Spy1193	-	1161949	1163442	497	phage prot	1981.744	2192.52	-0.14582	0.62654	0.80764	-1.10636	FALSE
Spy1194	-	1163435	1164703	422	phage prot	25.6448	35.91064	-0.48574	0.58951	0.77525	-1.4003	FALSE
Spy1195	-	1164700	1165056	118	phage prot	5.041796	16.27216	-1.6904	0.26284	0.49474	-3.22746	FALSE
Spy1196	-	1165205	1165549	114	HNH endor	5.755164	27.67137	-2.2655	0.07847	0.23218	-4.80821	FALSE
Spy1197	-	1165658	1166077	139	phage prot	25.81901	121.9111	-2.2393	0.000688	0.008475	-4.72168	TRUE
Spy1198	-	1166345	1166980	211	phage prot	1.268012	1.402756	-0.14569	1	1	-1.10626	FALSE
Spy1199	-	1166982	1167251	89	phage prot	8.423746	16.88378	-1.0031	0.44705	0.6651	-2.0043	FALSE
Spy1200	-	1167335	1167847	170	phage prot	14.2875	43.20383	-1.5964	0.078463	0.23218	-3.02388	FALSE
Spy1201	-	1167844	1168257	137	phage prot	28.0243	65.39232	-1.2224	0.094281	0.26215	-2.33335	FALSE
Spy1202	-	1168363	1168530	55	phage prot	NA	NA	NA	NA	NA	NA	Na
Spy1203	-	1168540	1169337	265	phage prot	40.10301	117.0633	-1.5455	0.15024	0.34724	-2.91905	FALSE
Spy1204	-	1169334	1170263	309	recT protei	26.96134	54.98754	-1.0282	0.24453	0.478	-2.03948	FALSE
Spy1205	-	1170266	1170595	109	phage prot	5.129194	13.03276	-1.3453	0.3914	0.60914	-2.54083	FALSE
Spy1206	-	1170651	1170857	68	phage prot	3.619008	10.83887	-1.5825	0.37097	0.59484	-2.99488	FALSE
spy1207	-	1170866	1171006	46	phage prot	NA	NA	NA	NA	NA	NA	Na
Spy1208	-	1171003	1171236	77	phage prot	1.772381	5.789616	-1.7078	0.54118	0.73641	-3.26662	FALSE
Spy1209	-	1171217	1171606	129	DNA replica	8.623681	19.86814	-1.2041	0.32363	0.54887	-2.30393	FALSE
Spy1210	-	1171751	1171990	79	phage repli	5.97512	16.09357	-1.4294	0.32498	0.54887	-2.69335	FALSE
Spy1211	-	1172090	1172275	61	phage prot	1.50507	2.499444	-0.73178	0.87122	0.94534	-1.66069	FALSE
Spy1212	xis	1172277	1172588	103	excisionase	2.747944	5.432067	-0.98315	0.70712	0.85056	-1.97678	FALSE
Spy1213	-	1172666	1172851	61	phage prot	10.159	27.74918	-1.4497	0.19877	0.41482	-2.73151	FALSE
Spy1214	-	1173018	1173257	79	phage prot	1376.128	14153.65	-3.3625	6.18E-06	0.000305	-10.2852	TRUE
Spy1215	-	1173399	1174205	268	phage prot	656.1812	4109.876	-2.6469	1.66E-06	0.000104	-6.2632	TRUE
Spy1216	-	1174140	1174406	88	phage prot	12.88868	43.84117	-1.7662	0.055148	0.18693	-3.40157	FALSE
Spy1217	-	1174438	1175154	238	phage antir	23.30184	76.15712	-1.7085	0.018841	0.08973	-3.26821	FALSE

Spy1218	-	1175166	1175357	63	phage prot	3.127792	25.55691	-3.0305	0.030954	0.12716	-8.17093	FALSE
Spy1219	-	1176511	1176858	115	Cro/CI fami	142.3426	418.5398	-1.556	0.000504	0.006968	-2.94037	TRUE
Spy1220	-	1176862	1177242	126	phage prot	100.2095	254.893	-1.3469	0.005081	0.036629	-2.54365	TRUE
Spy1221	-	1177254	1177520	88	phage prot	142.5423	302.2544	-1.0844	0.020822	0.097057	-2.12049	FALSE
Spy1222	int.2	1177644	1178786	380	integrase	294.2644	1333.078	-2.1796	2.21E-08	3.67E-06	-4.53028	TRUE
Spy1223	-	1178876	1179151	91	DNA-bindir	22588.16	30087.3	-0.41359	0.65213	0.81935	-1.332	FALSE
Spy1224	-	1179250	1179837	195	hypothetica	3583.314	2522.413	0.50649	0.11597	0.30267	1.42059	FALSE
Spy1225	-	1179815	1180657	280	lipase/acylh	5012.647	3244.842	0.62742	0.06278	0.20262	1.5448	FALSE
Spy1226	-	1180650	1181501	283	degV family	6199.265	3514.255	0.81888	0.015124	0.078746	1.764036	FALSE
Spy1227	-	1181717	1182658	313	hypothetica	5619.12	4906.421	0.19567	0.55204	0.74549	1.145256	FALSE
Spy1228	recN	1182830	1184491	553	DNA repair	5823.347	3814.719	0.61027	0.077286	0.22997	1.526545	FALSE
Spy1229	argR1	1184513	1184983	156	arginine re	682.4286	458.9312	0.5724	0.14774	0.34594	1.486995	FALSE
Spy1230	-	1184970	1185797	275	hemolysin	2065.235	1424.365	0.53599	0.14478	0.34162	1.449937	FALSE
Spy1231	fps	1185790	1186662	290	dimethylall	1527.989	1116.235	0.45299	0.23115	0.45928	1.368874	FALSE
Spy1232	xseB	1186662	1186877	71	exodeoxyri	3.190052	5.917134	-0.89132	0.71254	0.85393	-1.85487	FALSE
Spy1233	xseA	1186855	1188195	446	exodeoxyri	2058.395	1964.333	0.06748	0.88907	0.95561	1.047885	FALSE
Spy1234	folD	1188348	1189202	284	bifunctiona	697.7119	561.2462	0.314	0.44239	0.65978	1.24315	FALSE
Spy1235	-	1189410	1191113	567	phosphoglu	17798.27	6886.619	1.3699	0.000105	0.002513	2.584527	TRUE
Spy1236	phr	1191282	1192691	469	deoxyribod	774.3343	561.5061	0.46365	0.3754	0.59906	1.379026	FALSE
Spy1237	artP	1192840	1193574	244	arginine tra	253.7219	1158.312	-2.1907	0.000433	0.006315	-4.56527	TRUE
Spy1238	artQ	1193574	1194260	228	arginine tra	176.6885	755.3423	-2.0959	8.09E-05	0.00208	-4.27493	TRUE
Spy1239	-	1194387	1194617	76	hypothetica	824.489	1159.465	-0.49189	0.40167	0.61879	-1.40629	FALSE
Spy1240	clpE	1194915	1197197	760	ATP-depen	38852.44	26274.48	0.56434	0.11468	0.30091	1.478711	FALSE
Spy1241	mutT	1197325	1197780	151	7,8-dihydro	529.0071	452.5445	0.22523	0.58505	0.7716	1.168964	FALSE
Spy1242	-	1197831	1198133	100	hypothetica	4755.854	4429.778	0.10247	0.74696	0.86725	1.07361	FALSE
Spy1243	ileS	1198398	1201199	933	isoleucyl-tR	33067.55	17167.83	0.94571	0.004944	0.035786	1.926137	TRUE
Spy1244	divIVAS	1201472	1202230	252	cell divisior	15481.13	8838.568	0.80863	0.017016	0.083883	1.751547	FALSE
Spy1245	-	1202240	1203031	263	RNA bindin	10952.3	5791.305	0.91927	0.008062	0.049748	1.891158	TRUE
Spy1246	-	1203031	1203285	84	hypothetica	847.9438	386.3944	1.1339	0.003502	0.028138	2.194512	TRUE
Spy1247	-	1203290	1203958	222	hypothetica	12101.01	6190.62	0.96697	0.006175	0.042343	1.954731	TRUE
Spy1248	-	1203958	1204629	223	pyridoxal-5	7447.82	4272.023	0.8019	0.028089	0.11844	1.743396	FALSE
Spy1249	ftsZ	1204632	1205951	439	cell divisior	18289.17	8991.85	1.0243	0.003678	0.02901	2.033972	TRUE
Spy1250	ftsA	1205975	1207339	454	cell divisior	12671.19	4615.823	1.4569	3.21E-05	0.000992	2.745179	TRUE
Spy1251	divIB	1207551	1208699	382	cell divisior	8316.114	6970.389	0.25467	0.46267	0.68113	1.193063	FALSE
Spy1252	murG	1208700	1209803	367	undecaprer	8087.454	6696.67	0.27224	0.49326	0.7018	1.207681	FALSE
Spy1253	murD	1209782	1211140	452	UDP-N-acet	7292.778	5196.905	0.48882	0.16998	0.37204	1.403297	FALSE
Spy1254	-	1211510	1211761	83	hypothetica	3374.953	2133.544	0.66162	0.050792	0.17647	1.581858	FALSE
Spy1255	typA	1211883	1213724	613	GTP-bindin	37614.29	17009.33	1.145	0.000796	0.009612	2.211461	TRUE

Spy1256	-	1213907	1214296	129	rhodanese-	1306.15	1360.469	-0.05878	0.85447	0.93921	-1.04159	FALSE
Spy1257	glcK	1214306	1215277	323	glucokinase	2283.049	2340.347	-0.03576	0.86225	0.94124	-1.0251	FALSE
Spy1258	-	1215282	1215485	67	hypothetica	28.90968	66.46406	-1.201	0.27379	0.50682	-2.29899	FALSE
Spy1259	dpr	1215627	1216154	175	non-specifi	25848.39	37397.76	-0.53288	0.28939	0.51997	-1.44681	FALSE
Spy1260	-	1216382	1217008	208	prepilin pe	10.79988	22.13882	-1.0356	0.37633	0.60002	-2.04997	FALSE
Spy1261	-	1217090	1218169	359	ribosomal P	8163.921	5355.324	0.60829	0.079729	0.23456	1.524451	FALSE
Spy1262	-	1218173	1218793	206	transcriptio	3497.018	1595.856	1.1318	0.001751	0.017268	2.19132	TRUE
Spy1263	-	1219088	1219207	39	hypothetica	NA	NA	NA	NA	NA	NA	Na
Spy1264	-	1219241	1219942	233	ribose oper	NA	NA	NA	NA	NA	NA	Na
Spy1265	-	1220035	1220265	76	ribose oper	NA	NA	NA	NA	NA	NA	Na
Spy1266	-	1220631	1221668	345	ATP-depen	7880.969	3626.718	1.1197	0.001133	0.012679	2.173018	TRUE
Spy1267	coaD	1221655	1222146	163	phosphopa	1838.984	1059.119	0.79604	0.022588	0.10276	1.736329	FALSE
Spy1268	-	1222136	1222675	179	methyltran	2637.073	1566.91	0.75102	0.032361	0.13205	1.682982	FALSE
Spy1269	asnA	1222798	1223790	330	asparagine	3073.847	2026.831	0.60082	0.071859	0.22053	1.516578	FALSE
Spy1270	arcC	1224103	1225053	316	carbamate	14696.95	30491.8	-1.0529	0.15509	0.35311	-2.0747	FALSE
Spy1271	-	1225073	1226404	443	hypothetica	16787.28	40211.4	-1.2602	0.033048	0.13248	-2.39529	FALSE
Spy1272	-	1226421	1227914	497	arginine/or	23043.97	40637.58	-0.81842	0.1594	0.35806	-1.76347	FALSE
Spy1273	arcB	1228084	1229097	337	ornithine ca	41751.56	66128.07	-0.66343	0.24806	0.48044	-1.58384	FALSE
Spy1274	-	1229137	1229565	142	acetyltrans	12968.28	24093.55	-0.89366	0.25633	0.48653	-1.85788	FALSE
Spy1275	arcA	1229665	1230900	411	arginine de	44105.83	50755.36	-0.20259	0.75395	0.87149	-1.15076	FALSE
Spy1276	-	1231174	1231854	226	Crp/Fnr fan	1240.98	1551.034	-0.32175	0.60706	0.79222	-1.24985	FALSE
Spy1277	ahrC.2	1231996	1232469	157	arginine re	809.1044	873.1597	-0.10992	0.85907	0.93942	-1.07917	FALSE
Spy1278	-	1232635	1233351	238	hypothetica	5331.892	4443.789	0.26286	0.38321	0.60361	1.199855	FALSE
Spy1279	-	1233365	1234444	359	hypothetica	1611.164	1333.672	0.2727	0.49777	0.70332	1.208067	FALSE
Spy1280	-	1234517	1236250	577	two-compo	2265.991	1808.102	0.32567	0.42344	0.63989	1.253246	FALSE
Spy1281	-	1236247	1236987	246	two-compo	541.7372	442.3947	0.29226	0.51605	0.71963	1.224557	FALSE
Spy1282	msrA	1237075	1238181	368	bifunctiona	67.74015	79.11298	-0.2239	0.65661	0.82088	-1.16789	FALSE
Spy1283	tlpA	1238224	1238847	207	thiol:disulfi	22.19884	27.87599	-0.32854	0.72632	0.85822	-1.25574	FALSE
Spy1284	ccdA	1238860	1239570	236	cytochrome	13.78196	27.90217	-1.0176	0.31614	0.54349	-2.02455	FALSE
Spy1285	-	1240173	1240466	97	hypothetica	100.7052	128.159	-0.3478	0.5223	0.72282	-1.27262	FALSE
Spy1286	-	1240477	1241502	341	DNA polym	423.7831	429.3672	-0.01889	0.87623	0.94808	-1.01318	FALSE
Spy1287	-	1241499	1242173	224	hypothetica	206.7697	203.8561	0.020474	0.91895	0.97622	1.014293	FALSE
Spy1288	-	1242175	1243023	282	hypothetica	203.4278	130.6597	0.6387	0.25498	0.4864	1.556926	FALSE
Spy1289	-	1243028	1244923	631	hypothetica	318.1211	326.8911	-0.03923	0.8585	0.93942	-1.02757	FALSE
Spy1290	-	1244923	1245651	242	hypothetica	75.89703	93.67695	-0.30365	0.65225	0.81935	-1.23426	FALSE
Spy1291	-	1245784	1248186	800	ATP-depen	167.403	407.9959	-1.2852	0.27737	0.50949	-2.43716	FALSE
Spy1292	valS	1248346	1250751	801	valyl-tRNA	8188.506	8951.018	-0.12845	0.65621	0.82088	-1.09312	FALSE
Spy1293	-	1250995	1251558	187	hypothetica	326.7666	455.969	-0.48068	0.22143	0.44629	-1.3954	FALSE

Spy1294	-	1251555	1251734	59	ribosomal-|	211.3643	329.1112	-0.63884	0.12674	0.31366	-1.55708	FALSE
Spy1295	-	1252159	1252554	131	hypothetica	1027.619	1695.578	-0.72247	0.21174	0.43346	-1.65	FALSE
Spy1296	-	1252572	1252826	84	hypothetica	198.0734	519.4061	-1.3908	0.10678	0.28601	-2.62224	FALSE
Spy1297	-	1253305	1254057	250	3-deoxy-7-|	2229.458	3816.961	-0.77573	0.023651	0.10626	-1.71206	FALSE
Spy1298	aroB	1254113	1255186	357	3-dehydroc	8671.542	10815.98	-0.3188	0.32062	0.54758	-1.24729	FALSE
Spy1299	-	1255455	1255613	52	hypothetica	NA	NA	NA	NA	NA	NA	Na
Spy1300	-	1255621	1255926	101	hypothetica	601.3811	602.2571	-0.0021	0.94226	0.98648	-1.00146	FALSE
Spy1301	-	1255928	1256266	112	hypothetica	1587.083	1125.009	0.49644	0.15565	0.35311	1.410728	FALSE
Spy1302	-	1256319	1257074	251	SAM-deper	2384.714	2158.345	0.14389	0.63924	0.81583	1.10488	FALSE
Spy1303	aroE	1257309	1258187	292	shikimate 5	1374.501	1369.252	0.005521	0.98862	1	1.003834	FALSE
Spy1304	lacZ	1258325	1261741	1138	beta-galact	4692.987	6176.315	-0.39624	0.26337	0.49474	-1.31607	FALSE
Spy1305	-	1261761	1263245	494	two-compo	1292.746	1784.488	-0.46507	0.20657	0.42817	-1.38038	FALSE
Spy1306	-	1263245	1264969	574	two-compo	1603.194	2111.42	-0.39727	0.28189	0.51387	-1.31701	FALSE
Spy1307	-	1264959	1265564	201	hypothetica	396.8091	689.0761	-0.79622	0.046889	0.16607	-1.73655	FALSE
Spy1308	-	1265870	1267315	481	sugar-bindi	959.2603	1245.643	-0.3769	0.30103	0.53052	-1.29855	FALSE
Spy1309	-	1267396	1268322	308	sugar trans	341.6344	451.0418	-0.40081	0.34414	0.56601	-1.32025	FALSE
Spy1310	-	1268332	1269282	316	sugar trans	487.8313	714.5835	-0.55072	0.15421	0.35269	-1.46482	FALSE
Spy1311	-	1269478	1270356	292	glucokinase	61.04187	77.81269	-0.35021	0.53017	0.72873	-1.27475	FALSE
Spy1312	-	1270500	1270622	40	hypothetica	NA	NA	NA	NA	NA	NA	Na
Spy1313	-	1270968	1272410	480	beta-glucos	4658.619	5541.648	-0.25041	0.45288	0.66996	-1.18955	FALSE
Spy1314	hyl	1272434	1274128	564	hyaluronog	4845.232	4708.666	0.041247	0.89572	0.9588	1.029003	FALSE
Spy1315	-	1274179	1275219	346	GntR family	2062.948	1938.508	0.08976	0.78979	0.89533	1.064193	FALSE
Spy1316	-	1275352	1276638	428	hypothetica	2291.501	2509.68	-0.13121	0.66376	0.82698	-1.09521	FALSE
Spy1317	-	1276653	1279358	901	alpha-manr	7824.25	10226.08	-0.38623	0.24563	0.478	-1.30697	FALSE
Spy1318	rocA	1279459	1280814	451	sensory tra	2216.186	4376.666	-0.98175	0.015274	0.078922	-1.97486	FALSE
Spy1319	-	1281479	1282834	451	tRNA (uraci	891.2266	1031.35	-0.21067	0.52361	0.72354	-1.15723	FALSE
Spy1320	recX	1282949	1283725	258	recombinat	319.001	305.871	0.060637	0.82317	0.91865	1.042926	FALSE
Spy1321	-	1283805	1284338	177	hypothetica	6881.565	8818.622	-0.35782	0.64711	0.8174	-1.28149	FALSE
Spy1322	-	1284437	1284586	49	hypothetica	NA	NA	NA	NA	NA	NA	Na
Spy1323	-	1284708	1284986	92	transposase	55.22503	64.19502	-0.21714	0.77105	0.88331	-1.16243	FALSE
Spy1324	-	1291239	1291373	44	hypothetica	NA	NA	NA	NA	NA	NA	Na
Spy1325	-	1292030	1292578	182	ribosome-a	23446.74	35829.54	-0.61176	0.37031	0.59458	-1.52812	FALSE
Spy1326	comFC	1292658	1293323	221	competenc	54.27344	113.7498	-1.0675	0.068795	0.21524	-2.0958	FALSE
Spy1327	comFA	1293295	1294509	404	competenc	145.5032	282.744	-0.95845	0.18381	0.3943	-1.94322	FALSE
Spy1328	-	1294676	1295308	210	Xaa-Pro dip	1644.385	617.3589	1.4134	0.000134	0.002973	2.663642	TRUE
Spy1329	cysM	1295436	1296377	313	cysteine sy	5346.401	6320.197	-0.2414	0.50349	0.70643	-1.18214	FALSE
Spy1330	-	1296395	1296772	125	hypothetica	3257.463	2916.97	0.15928	0.67659	0.83385	1.11673	FALSE
Spy1331	-	1296772	1298172	466	peptidyl-pr	12152.24	7940.49	0.61392	0.067706	0.21366	1.530412	FALSE

Spy1332	yvqC	1298209	1298850	213	two-compo	1360.254	1033.387	0.3965	0.28984	0.51997	1.316311	FALSE
Spy1333	yvqE	1298843	1299847	334	two-compo	1500.242	1158.055	0.37349	0.30661	0.53707	1.295483	FALSE
Spy1334	yvqF	1299844	1300536	230	transporter	635.8327	595.4047	0.094777	0.85693	0.93942	1.0679	FALSE
Spy1335	-	1300659	1302557	632	serine/thre	32500.16	17726.71	0.87452	0.01392	0.074898	1.833398	FALSE
Spy1336	pppL	1302554	1303294	246	protein pho	7664.103	3967.408	0.94992	0.008537	0.051903	1.931766	FALSE
Spy1337	sunL	1303332	1304654	440	16S rRNA m	5127.018	3861.451	0.40898	0.35202	0.57638	1.327747	FALSE
Spy1338	fmt	1304644	1305579	311	methionyl-t	2929.862	3237.818	-0.14419	0.64353	0.81627	-1.10511	FALSE
Spy1339	priA	1305641	1308025	794	primosome	1542.946	2387.552	-0.62984	0.37925	0.60098	-1.54739	FALSE
Spy1340	-	1308090	1308407	105	DNA-direct	1397.5	1915.125	-0.45459	0.22749	0.45349	-1.37039	FALSE
Spy1341	gmk	1308423	1309058	211	guanylate k	2140.387	3730.634	-0.80155	0.022494	0.10276	-1.74297	FALSE
Spy1342	-	1309168	1310775	535	hypothetica	21037.93	18800.39	0.16223	0.64512	0.81715	1.119015	FALSE
Spy1343	-	1310905	1311801	298	LysR family	1273.796	1087.303	0.22838	0.55663	0.749	1.171519	FALSE
Spy1344	atoB	1312003	1313190	395	acetyl-CoA	1854.698	831.3145	1.1577	0.001331	0.01445	2.231015	TRUE
Spy1345	atoD.1	1313214	1313864	216	acetate Co/	713.0809	284.7537	1.3244	0.000905	0.010646	2.504287	TRUE
Spy1346	atoA	1313866	1314525	219	acetate Co/	966.9087	435.5138	1.1507	0.00281	0.024293	2.220216	TRUE
Spy1347	-	1314558	1315337	259	3-hydroxyb	1433.107	697.2848	1.0393	0.004537	0.033915	2.05523	TRUE
Spy1348	-	1315408	1316739	443	D-beta-hyd	1773.712	989.2656	0.84234	0.016001	0.08018	1.792956	FALSE
Spy1349	luxS	1316723	1317295	190	S-ribosylho	2261.373	1713.153	0.40055	0.21171	0.43346	1.320011	FALSE
Spy1350	-	1317440	1318909	489	hypothetica	15147.84	13919.4	0.12202	0.73403	0.86046	1.088258	FALSE
Spy1351	-	1318923	1320077	384	methyltran	6020.433	4705.333	0.35557	0.32031	0.54758	1.279491	FALSE
Spy1352	-	1320522	1320848	108	cell division	1775.494	2398.897	-0.43415	0.46224	0.68104	-1.35111	FALSE
Spy1353	-	1320970	1321485	171	hypothetica	462.9668	297.3884	0.63856	0.083911	0.24103	1.556775	FALSE
Spy1354	recU	1321566	1322165	199	Holliday jun	3513.044	4109.686	-0.22631	0.58224	0.76846	-1.16984	FALSE
Spy1355	pbp1A	1322152	1324317	721	multimodu	28716.19	26112.97	0.1371	0.63662	0.81544	1.099692	FALSE
Spy1356	pepC	1324784	1326121	445	aminopept	20459.86	14595.95	0.48723	0.14528	0.3419	1.401751	FALSE
Spy1357	nadE	1326306	1327130	274	NAD synthe	4674.274	2478.062	0.91553	0.008723	0.052686	1.886262	FALSE
Spy1358	nadE	1327132	1328586	484	nicotinate p	5625.235	2770.478	1.0218	0.003077	0.026103	2.030451	TRUE
Spy1359	-	1328757	1330136	459	amino acid	7671.915	8265.157	-0.10746	0.7399	0.86377	-1.07733	FALSE
Spy1360	-	1330305	1331222	305	thioredoxin	3020.774	2917.706	0.050084	0.94779	0.9873	1.035325	FALSE
Spy1361	aapA	1331286	1331510	74	hypothetica	155.1089	251.0454	-0.69467	0.12045	0.30728	-1.61851	FALSE
Spy1362	-	1331614	1332360	248	transporter	2726.475	3897.173	-0.51539	0.12198	0.30888	-1.42938	FALSE
Spy1363	-	1332357	1333160	267	amino acid	2367.475	3288.021	-0.47387	0.13759	0.3306	-1.38883	FALSE
Spy1364	-	1333355	1334698	447	ATP-depen	5550.076	4748.044	0.22517	0.51784	0.71992	1.168915	FALSE
Spy1365	mraY	1334856	1335866	336	phospho-N	4247.717	4514.758	-0.08796	0.79203	0.89719	-1.06287	FALSE
Spy1366	-	1335868	1338123	751	division spe	6856.414	6380.859	0.1037	0.86658	0.94348	1.074526	FALSE
Spy1367	ftsL	1338127	1338450	107	cell division	187.7591	194.395	-0.05011	0.86144	0.94124	-1.03534	FALSE
Spy1368	mraW	1338455	1339468	337	S-adenosyl-	1910.776	2263.371	-0.24431	0.45039	0.66682	-1.18453	FALSE
Spy1369	-	1339492	1339599	35	hypothetica	NA	NA	NA	NA	NA	NA	Na

Spy1370	proA	1339942	1341192	416	gamma-glu	2339.857	2673.417	-0.19226	0.49585	0.7022	-1.14255	FALSE
Spy1371	-	1341185	1342006	273	gamma-glu	659.7722	720.5769	-0.12718	0.63856	0.81583	-1.09216	FALSE
Spy1372	proB	1342071	1343699	542	ABC transp	22342.19	19825.61	0.17241	0.67632	0.83385	1.126939	FALSE
Spy1373	-	1343704	1344438	244	ABC transp	4154.973	3197.623	0.37784	0.29722	0.52788	1.299395	FALSE
Spy1374	-	1344472	1344738	88	hypothetica	1152.379	1054.028	0.1287	0.72183	0.85775	1.093308	FALSE
Spy1375	tkt	1344931	1346916	661	transketola	17667.32	17951.37	-0.02301	1	1	-1.01608	FALSE
Spy1376	-	1347134	1347778	214	transladola	536.7812	664.8911	-0.30878	0.38658	0.60649	-1.23866	FALSE
Spy1377	-	1347904	1349403	499	trans-acting	659.9203	960.7476	-0.54187	0.12295	0.30933	-1.45586	FALSE
Spy1378	-	1349393	1350739	448	NADH pero	567.9334	781.9166	-0.46129	0.23587	0.46611	-1.37677	FALSE
Spy1379	glpF	1350848	1351549	233	glycerol up	596.3221	784.9602	-0.39653	0.24287	0.47557	-1.31634	FALSE
Spy1380	glpO	1351551	1353389	612	alpha-glyce	1354.272	1267.594	0.095424	0.97876	0.99967	1.068379	FALSE
Spy1381	glpK	1353405	1354931	508	glycerol kin	792.6393	658.654	0.26714	0.81321	0.91243	1.20342	FALSE
Spy1382	-	1355292	1355684	130	hypothetica	19984	15184.26	0.39627	0.27583	0.50819	1.316101	FALSE
Spy1383	-	1355811	1356068	85	hypothetica	1990.378	1804.797	0.14121	0.70634	0.8504	1.10283	FALSE
Spy1384	glyS	1356222	1358261	679	glycyl-tRNA	11284.76	11307.4	-0.00289	0.88305	0.9525	-1.00201	FALSE
Spy1385	glyQ	1358639	1359556	305	glycyl-tRNA	3067.575	2660.969	0.20515	0.61752	0.7994	1.152806	FALSE
Spy1386	-	1359928	1360461	177	hypothetica	5692.663	10243.45	-0.84753	0.015345	0.079063	-1.79942	FALSE
Spy1387	-	1360593	1361432	279	aldo/keto r	9065.939	10595.85	-0.22497	0.48485	0.6958	-1.16875	FALSE
Spy1388	nagA	1361554	1362702	382	N-acetylglu	8926.981	10392.5	-0.2193	0.46544	0.68354	-1.16417	FALSE
Spy1389	-	1362820	1364469	549	sodium-dep	3132.915	4024.184	-0.36119	0.31189	0.53975	-1.28448	FALSE
Spy1390	-	1364653	1365375	240	hypothetica	2406.391	1885.588	0.35186	0.29622	0.52662	1.276205	FALSE
Spy1391	-	1365504	1366346	280	degV family	1470.424	1422.96	0.047336	0.81897	0.91588	1.033355	FALSE
Spy1392	-	1366639	1367196	185	TetR family	1070.593	1714.368	-0.67927	0.06989	0.21607	-1.60133	FALSE
Spy1393	-	1367232	1368056	274	HAD superf	1482.622	2465.741	-0.73387	0.060476	0.19875	-1.66309	FALSE
Spy1394	-	1368058	1368675	205	hypothetica	779.4901	1575.636	-1.0153	0.1124	0.29626	-2.02132	FALSE
Spy1395	lacD.1	1368872	1369849	325	tagatose 1,	518.7298	8739.316	-4.0745	0.003414	0.027554	-16.8479	TRUE
Spy1396	-	1369999	1370349	116	tagatose-6-	131.8538	2315.523	-4.1343	0.002432	0.021743	-17.561	TRUE
Spy1397	lacB.1	1370359	1370874	171	galactose-6	154.2995	2615.917	-4.0835	0.002573	0.022735	-16.9534	TRUE
Spy1398	lacA.1	1370889	1371314	141	galactose-6	263.5966	3699.087	-3.8108	0.001943	0.01875	-14.0335	TRUE
Spy1399	-	1371554	1373002	482	PTS system	1802.02	21331.43	-3.5653	0.002333	0.021278	-11.8376	TRUE
Spy1400	-	1373031	1373336	101	PTS system	70.94569	766.1137	-3.4328	0.003306	0.027264	-10.7988	TRUE
Spy1401	-	1373329	1373802	157	PTS system	217.0919	2114.016	-3.2836	0.007517	0.048447	-9.73783	TRUE
Spy1402	lacR.1	1374039	1374809	256	lactose pho	959.5385	2792.435	-1.5411	0.029469	0.12272	-2.91016	FALSE
Spy1403	-	1374836	1375006	56	copper cha	3.015256	15.68565	-2.3791	0.15504	0.35311	-5.20212	FALSE
Spy1404	copZ	1375013	1375216	67	copper cha	140.8891	177.0036	-0.32922	0.52986	0.72873	-1.25633	FALSE
Spy1405	copA	1375230	1377461	743	copper-exp	25914.88	19866.84	0.38342	0.40845	0.62502	1.30443	FALSE
Spy1406	copY	1377461	1377895	144	copAB ATPa	1497.733	1661.904	-0.15006	0.63243	0.8118	-1.10962	FALSE
Spy1407	-	1378067	1379053	328	esterase	203.5882	457.2666	-1.1674	0.005185	0.037237	-2.24607	TRUE

Spy1408	rbfA	1379187	1379543	118	ribosome-b	NA	NA	NA	NA	NA	NA	Na
Spy1409	infB	1379742	1382603	953	translation	44883.89	43556.66	0.043304	0.97774	0.99967	1.030471	FALSE
Spy1410	-	1382623	1382925	100	hypothetica	676.6025	673.2484	0.00717	0.91777	0.97554	1.004982	FALSE
Spy1411	-	1382918	1383214	98	hypothetica	334.4495	482.8809	-0.52988	0.56067	0.75083	-1.44381	FALSE
Spy1412	nusA	1383230	1384387	385	transcriptio	8457.145	8792.024	-0.05602	0.79247	0.89719	-1.0396	FALSE
Spy1413	-	1384562	1385098	178	hypothetica	2335.286	3503.092	-0.58503	0.073898	0.22353	-1.50007	FALSE
Spy1414	-	1385343	1385522	59	phage prot	14.77696	35.24732	-1.2542	0.19775	0.41412	-2.38535	FALSE
Spy1415	sdaD2	1385761	1386933	390	phage-enco	610.4115	986.5129	-0.69256	0.056941	0.19127	-1.61615	FALSE
Spy1416	-	1387049	1388245	398	phage-asso	159.4441	350.0936	-1.1347	0.01022	0.059747	-2.19573	FALSE
Spy1417	-	1388356	1388541	61	phage prot	10.47542	24.20361	-1.2082	0.32192	0.54806	-2.31049	FALSE
Spy1418	-	1388538	1388837	99	phage prot	4.202739	12.21641	-1.5394	0.3867	0.60649	-2.90674	FALSE
Spy1419	-	1388848	1389468	206	phage prot	37.65452	76.9975	-1.032	0.11865	0.30439	-2.04486	FALSE
Spy1420	-	1389471	1389632	53	phage prot	0	0.612028	#NAME?	0.94241	0.98648	NA	FALSE
Spy1421	-	1389641	1391548	635	phage infe	143.4242	484.1967	-1.7553	4.39E-05	0.001195	-3.37597	TRUE
Spy1422	-	1391559	1392194	211	phage prot	20.98505	74.72742	-1.8323	0.019002	0.090258	-3.56104	FALSE
Spy1423	-	1392194	1393249	351	hyaluronog	61.5765	156.5174	-1.3459	0.015686	0.079555	-2.54189	FALSE
Spy1424	-	1393246	1395228	660	phage endo	99.70338	302.66	-1.602	0.000625	0.008131	-3.03564	TRUE
Spy1425	-	1395238	1396080	280	phage prot	41.22545	146.8544	-1.8328	0.001961	0.018823	-3.56228	TRUE
Spy1426	-	1396092	1400474	1460	phage prot	351.5292	1092.136	-1.6354	1.76E-05	0.000656	-3.10674	TRUE
Spy1427	-	1400489	1400722	77	phage prot	6.025395	12.29311	-1.0287	0.49971	0.70332	-2.04019	FALSE
Spy1428	-	1400797	1401252	151	phage prot	22.49421	61.43891	-1.4496	0.066794	0.21183	-2.73132	FALSE
Spy1429	-	1401306	1401905	199	phage prot	33.80752	87.27364	-1.3682	0.04352	0.15973	-2.58148	FALSE
Spy1430	-	1401917	1402276	119	phage prot	6.042497	13.33847	-1.1424	0.47712	0.68865	-2.20748	FALSE
Spy1431	-	1402280	1402606	108	phage prot	5.943113	24.58612	-2.0486	0.12179	0.30888	-4.13704	FALSE
Spy1432	-	1402621	1402899	92	phage prot	3.331675	11.22117	-1.7519	0.38039	0.60098	-3.36802	FALSE
Spy1433	-	1402910	1403266	118	phage prot	10.32577	39.76163	-1.9451	0.058265	0.19429	-3.85064	FALSE
Spy1434	-	1403278	1404165	295	phage prot	37.8264	97.19838	-1.3615	0.03396	0.13452	-2.56952	FALSE
Spy1435	-	1404178	1404747	189	phage scaff	58.23575	143.9472	-1.3056	0.018839	0.08973	-2.47187	FALSE
Spy1436	-	1404903	1405169	88	phage prot	0.634006	2.856587	-2.1717	0.71252	0.85393	-4.50554	FALSE
Spy1437	-	1405172	1405360	62	hypothetica	0.329572	0.280551	0.23233	1	1	1.174731	FALSE
Spy1438	-	1405391	1406830	479	phage prot	56.3599	148.7158	-1.3998	0.013869	0.074842	-2.63865	FALSE
Spy1439	-	1406796	1408328	510	portal prot	40.42176	129.4323	-1.679	0.005337	0.038023	-3.20206	TRUE
Spy1440	-	1408344	1409621	425	terminase l	30.57348	95.71869	-1.6465	0.015153	0.078746	-3.13073	FALSE
Spy1441	-	1409611	1410105	164	phage term	11.84457	32.41689	-1.4525	0.14988	0.34724	-2.73682	FALSE
Spy1442	-	1410153	1410569	138	phage trans	18.19779	37.74698	-1.0526	0.40119	0.61858	-2.07426	FALSE
Spy1443	-	1410566	1410757	63	phage prot	0	0	NA	NA	NA	NA	NA
Spy1444	-	1410747	1411598	283	adenine-sp	33.01991	44.40239	-0.4273	0.6508	0.81922	-1.34471	FALSE
Spy1445	-	1411607	1411873	88	phage prot	12.58308	17.57251	-0.48184	0.62788	0.80822	-1.39652	FALSE

Spy1446	-	1411870	1412037	55	phage prot	NA	NA	NA	NA	NA	NA	Na
Spy1447	-	1412038	1413360	440	phage-relat	87.90616	279.9335	-1.671	0.11773	0.30402	-3.18435	FALSE
Spy1448	-	1413357	1413632	91	hypothetica	11.50755	49.02055	-2.0908	0.15247	0.35071	-4.25984	FALSE
Spy1449	-	1414019	1416403	794	DNA prima	197.4179	720.4974	-1.8677	0.12199	0.30888	-3.6495	FALSE
Spy1450	-	1416408	1418330	640	phage-enco	193.2967	596.8272	-1.6265	0.25157	0.48353	-3.08763	FALSE
Spy1451	-	1418373	1418930	185	phage prot	80.99435	273.0196	-1.7531	0.001098	0.01236	-3.37082	TRUE
Spy1452	-	1418941	1419339	132	phage prot	18.52049	99.54545	-2.4262	0.087373	0.24948	-5.37476	FALSE
Spy1453	-	1419343	1420497	384	phage prot	132.6131	443.3115	-1.7411	0.072028	0.22053	-3.3429	FALSE
Spy1454	-	1420497	1420796	99	phage prot	22.6769	49.12264	-1.1152	0.18194	0.39089	-2.16625	FALSE
Spy1455	-	1420884	1421087	67	phage prot	15.12539	37.5428	-1.3116	0.16066	0.36025	-2.48217	FALSE
Spy1456	-	1421084	1421236	50	phage prot	NA	NA	NA	NA	NA	NA	Na
Spy1457	-	1421233	1421619	128	phage prot	20.07072	48.61216	-1.2762	0.11728	0.30386	-2.422	FALSE
Spy1458	-	1421616	1421819	67	phage prot	0	0.662954	#NAME?	0.93797	0.98523	NA	FALSE
Spy1459	-	1421812	1421982	56	phage prot	0	0	NA	NA	NA	NA	NA
Spy1460	-	1421979	1422254	91	phage prot	5.650664	20.63325	-1.8685	0.18461	0.3943	-3.65153	FALSE
Spy1461	-	1422316	1422531	71	phage prot	17.49977	84.1664	-2.2659	0.00695	0.046095	-4.80954	TRUE
Spy1462	-	1422579	1422992	137	phage prot	227.6566	465.0497	-1.0305	0.018309	0.088349	-2.04273	FALSE
Spy1463	-	1422973	1423128	51	phage prot	NA	NA	NA	NA	NA	NA	Na
Spy1464	-	1423403	1423804	133	Cro/CI fami	118.7921	323.046	-1.4433	0.003348	0.027264	-2.71942	TRUE
Spy1465	-	1423818	1424201	127	phage prot	170.7609	319.6436	-0.90449	0.036863	0.14245	-1.87188	FALSE
Spy1466	-	1424212	1424763	183	phage prot	910.5779	1219.281	-0.42118	0.24553	0.478	-1.33902	FALSE
Spy1467	int.3	1424880	1425959	359	integrase	271.3956	1394.028	-2.3608	0.00016	0.003429	-5.13655	TRUE
Spy1468	trmB	1426157	1426792	211	tRNA (guan	2772.163	3459.432	-0.31952	0.40902	0.62535	-1.24792	FALSE
Spy1469	-	1426792	1427583	263	phosphotra	2582.631	2693.617	-0.0607	0.85514	0.93921	-1.04297	FALSE
Spy1470	-	1427647	1428681	344	protein ecs	1291.346	1250.269	0.046638	0.82294	0.91865	1.032855	FALSE
Spy1471	-	1428684	1429409	241	ABC transp	1121.239	1129.456	-0.01054	0.95117	0.98913	-1.00733	FALSE
Spy1472	hit	1429481	1429900	139	bis(5\'-nucl	765.1757	1351.279	-0.82046	0.052479	0.18027	-1.76597	FALSE
Spy1473	-	1429897	1430253	118	hypothetica	901.0114	1583.743	-0.81372	0.029056	0.12156	-1.75774	FALSE
Spy1474	lytR	1430364	1431638	424	LytR family	5598.133	7727.215	-0.465	0.17084	0.37318	-1.38032	FALSE
Spy1475	-	1431647	1432171	174	acetyltrans	872.0637	1069.38	-0.29427	0.42887	0.64543	-1.22626	FALSE
Spy1476	-	1432146	1432607	153	ATP/GTP hy	2.38125	536.0534	-7.8145	4.80E-07	3.98E-05	-225.112	TRUE
Spy1477	-	1432761	1434221	486	guanine-hy	1355.983	5682.743	-2.0672	2.26E-05	0.000763	-4.19073	TRUE
Spy1478	-	1434485	1435297	270	HAD superf	3772.545	5078.555	-0.42888	0.25365	0.48496	-1.34619	FALSE
Spy1479	manL	1435649	1436641	330	PTS system	15830.99	9965.329	0.66776	0.039514	0.14861	1.588605	FALSE
Spy1480	manM	1436730	1437539	269	PTS system	13555.67	8099.15	0.74305	0.023801	0.10667	1.67371	FALSE
Spy1481	manN	1437556	1438467	303	PTS system	26602.3	16077.23	0.72653	0.024619	0.10899	1.654654	FALSE
Spy1482	manO	1438581	1438940	119	hypothetica	526.8854	793.7608	-0.59122	0.2078	0.42925	-1.50652	FALSE
Spy1483	serS	1439332	1440609	425	seryl-tRNA	8662.392	7120.09	0.28287	0.38507	0.60497	1.216613	FALSE

Spy1484	accD	1440831	1441601	256	acetyl-CoA	8265.639	5363.556	0.62394	0.050894	0.17648	1.541078	FALSE
Spy1485	accA	1441598	1442464	288	acetyl-CoA	7042.274	4503.311	0.64506	0.04394	0.15987	1.563804	FALSE
Spy1486	accC	1442473	1443837	454	acetyl-CoA	12368.45	7437.839	0.73371	0.025926	0.11206	1.66291	FALSE
Spy1487	fabZ	1443869	1444291	140	(3R)-hydrox	2236.121	1422.473	0.6526	0.05168	0.17887	1.571999	FALSE
Spy1488	accB	1444288	1444788	166	acetyl-CoA	2715.359	1472.044	0.88332	0.009468	0.055886	1.844615	FALSE
Spy1489	fabF	1444790	1446022	410	3-oxoacyl-A	9354.612	4784.166	0.96741	0.003585	0.028556	1.955327	TRUE
Spy1490	fabG	1446037	1446771	244	3-ketoacyl-	3411.585	1622.415	1.0723	0.001587	0.015816	2.102783	TRUE
Spy1491	fabD	1446761	1447699	312	ACP S-malo	3415.6	1816.863	0.91069	0.007654	0.048627	1.879944	TRUE
Spy1492	fabK	1447724	1448695	323	enoyl-ACP r	3904.148	1727.643	1.1762	0.000633	0.008131	2.259808	TRUE
Spy1493	acpP	1448897	1449121	74	acyl carrier	1860.287	2402.618	-0.36908	0.64088	0.81627	-1.29153	FALSE
Spy1494	fabH	1449182	1450156	324	3-oxoacyl-A	10449.06	9490.654	0.13879	0.64006	0.81584	1.100981	FALSE
Spy1495	-	1450157	1450591	144	MarR famil	4843.346	4764.154	0.023784	0.84822	0.93597	1.016622	FALSE
Spy1496	phaB	1450668	1451459	263	enoyl-CoA I	9679.336	10612.05	-0.13272	0.83975	0.93056	-1.09636	FALSE
Spy1497	dnaJ	1451679	1452836	385	molecular c	17710.96	41448.38	-1.2267	0.000283	0.004786	-2.34031	TRUE
Spy1498	dnaK	1453117	1454943	608	molecular c	148106.3	169601	-0.19551	0.51783	0.71992	-1.14513	FALSE
Spy1499	grpE	1455124	1455657	177	heat shock	20346.03	29245.87	-0.52349	0.097492	0.26833	-1.43743	FALSE
Spy1500	hrcA	1455699	1456733	344	heat-induci	28936.6	47896.48	-0.72702	0.032986	0.13248	-1.65522	FALSE
Spy1501	-	1456868	1457437	189	N-acetylmu	1647.623	1763.434	-0.098	0.74942	0.8679	-1.07029	FALSE
Spy1502	-	1457434	1458177	247	D-alanyl-D-	1521.798	1390.571	0.1301	0.75661	0.87235	1.09437	FALSE
Spy1503	-	1458167	1458874	235	phosphogly	1504.853	1012.652	0.57148	0.12871	0.31512	1.486047	FALSE
Spy1504	-	1459265	1459513	82	hypothetica	616.3408	899.66	-0.54565	0.16077	0.36025	-1.45968	FALSE
Spy1505	-	1459723	1459938	71	hypothetica	245.5813	379.7892	-0.629	0.13294	0.32287	-1.54649	FALSE
Spy1506	gatB	1459935	1461374	479	aspartyl/glu	11468.79	8504.671	0.43139	0.20464	0.42562	1.348532	FALSE
Spy1507	gatA	1461374	1462840	488	aspartyl/glu	8464.164	6367.001	0.41075	0.21709	0.43948	1.329377	FALSE
Spy1508	gatC	1462840	1463142	100	aspartyl/glu	859.6952	842.4618	0.029214	0.96932	0.99664	1.020456	FALSE
Spy1509	-	1463374	1463583	69	pyruvate, p	20.76582	57.28268	-1.4639	0.26807	0.49945	-2.75853	FALSE
Spy1510	-	1463555	1463671	38	pyruvate, p	30.27591	70.74997	-1.2246	0.090571	0.25613	-2.33691	FALSE
Spy1511	-	1464245	1464691	148	pyrazinami	438.6937	843.8962	-0.94385	0.097339	0.26833	-1.92365	FALSE
Spy1512	codY	1464838	1465620	260	transcriptio	13381.17	10933.9	0.2914	0.37162	0.59484	1.223827	FALSE
Spy1513	-	1465838	1467052	404	aminotrans	14268.9	10067.14	0.50322	0.16863	0.37058	1.417374	FALSE
Spy1514	-	1467283	1467735	150	universal st	6908.884	7030.804	-0.02524	0.93382	0.98399	-1.01765	FALSE
Spy1515	-	1467858	1469135	425	HAD superf	2651.744	2821.989	-0.08977	0.79291	0.89719	-1.0642	FALSE
Spy1516	asnB	1469318	1470283	321	L-asparagin	1084.862	934.3626	0.21546	0.53795	0.735	1.161074	FALSE
Spy1517	-	1470632	1471336	234	lantibiotic t	599.1881	844.3917	-0.4949	0.25782	0.48783	-1.40922	FALSE
Spy1518	-	1471349	1472251	300	transporter	488.3146	699.1848	-0.51786	0.20636	0.42817	-1.43183	FALSE
Spy1519	recG	1472544	1474559	671	ATP-depen	6877.476	6346.588	0.1159	0.75769	0.87249	1.083651	FALSE
Spy1520	-	1474652	1474852	66	hypothetica	18.48337	27.36673	-0.5662	0.53988	0.73641	-1.48062	FALSE
Spy1521	-	1474934	1476388	484	cobalt ABC	6347.939	5472.67	0.21404	0.55316	0.74572	1.159932	FALSE

Spy1522	-	1476325	1477005	226	cobalt trans	1837.673	1697.779	0.11423	0.73374	0.86046	1.082397	FALSE
Spy1523	-	1477002	1477595	197	permease	1086.107	1043.054	0.058353	0.86994	0.94509	1.041276	FALSE
Spy1524	-	1477592	1479262	556	ABC transp	1457.506	1567.976	-0.1054	0.75639	0.87235	-1.07579	FALSE
Spy1525	-	1479255	1481018	587	ABC transp	1010.148	1155.475	-0.19392	0.54548	0.73919	-1.14387	FALSE
Spy1526	fhuC	1481015	1481851	278	ferrichrome	376.5146	478.767	-0.34662	0.32721	0.5511	-1.27158	FALSE
Spy1527	-	1481848	1482870	340	ferrichrome	401.6902	527.2556	-0.39242	0.28068	0.51349	-1.31259	FALSE
Spy1528	-	1482872	1483756	294	ferrichrome	363.0615	404.8815	-0.15729	0.64571	0.81733	-1.11519	FALSE
Spy1529	shp	1483740	1484615	291	heme bindi	371.5732	431.7894	-0.21668	0.54101	0.73641	-1.16206	FALSE
Spy1530	-	1484812	1488639	1275	Fe3+-sidero	2789.867	2999.877	-0.10471	0.72806	0.85842	-1.07528	FALSE
Spy1531	isp2	1489128	1490639	503	hypothetica	23996.51	27997.39	-0.22247	0.49307	0.7018	-1.16673	FALSE
Spy1532	alr	1490726	1491826	366	alanine rac	4765.599	4822.243	-0.01705	0.86262	0.94124	-1.01189	FALSE
Spy1533	acpS	1491823	1492179	118	4\'-phosph	1299.684	1338.937	-0.04293	0.8309	0.92244	-1.0302	FALSE
Spy1534	secA	1492295	1494814	839	preprotein	41947.54	36553.09	0.19859	0.60023	0.78595	1.147576	FALSE
Spy1535	-	1494892	1494996	34	hypothetica	NA	NA	NA	NA	NA	NA	Na
Spy1536	-	1494980	1495495	171	transposase	NA	NA	NA	NA	NA	NA	Na
Spy1537	-	1495398	1496102	234	transposase	NA	NA	NA	NA	NA	NA	Na
Spy1538	pmi	1496264	1497217	317	mannose-6	3066.019	3496.068	-0.18937	0.59237	0.77839	-1.14027	FALSE
Spy1539	scrK	1497312	1498268	318	fructokinas	275.0908	829.5325	-1.5924	0.003925	0.030337	-3.01551	TRUE
Spy1540	endoS	1498460	1501447	995	endo-beta-	1968.623	6698.064	-1.7666	0.004081	0.031411	-3.40251	TRUE
Spy1541	-	1501471	1501635	54	hypothetica	NA	NA	NA	NA	NA	NA	Na
Spy1542	scrA	1501678	1503561	627	PTS system	1446.755	6099.138	-2.0758	0.000247	0.004466	-4.21578	TRUE
Spy1543	scrB	1503803	1505242	479	sucrose-6-p	604.0688	1355.925	-1.1665	0.001576	0.015791	-2.24466	TRUE
Spy1544	scrR	1505247	1506212	321	sucrose ope	807.1978	1970.771	-1.2878	0.000487	0.006881	-2.44155	TRUE
Spy1545	nusB	1506353	1506805	150	transcriptio	6642.265	6211.426	0.096751	0.7074	0.85056	1.069363	FALSE
Spy1546	-	1506798	1507187	129	Gls24 famil	1962.075	1510.223	0.37762	0.30848	0.53806	1.299197	FALSE
Spy1547	efp	1507233	1507790	185	elongation	7099.131	5124.365	0.47027	0.1543	0.35269	1.385369	FALSE
Spy1548	comEB	1507886	1508347	153	competenc	908.9223	1077.275	-0.24516	0.45015	0.66682	-1.18522	FALSE
Spy1549	-	1508382	1509455	357	Xaa-Pro dip	2048.118	2627.804	-0.35956	0.28177	0.51387	-1.28303	FALSE
Spy1550	uvrA	1509570	1512428	952	excinucleas	16594.35	12550.52	0.40294	0.30885	0.53806	1.3222	FALSE
Spy1551	corA	1512601	1513545	314	magnesium	4986.188	5148.949	-0.04634	0.99342	1	-1.03264	FALSE
Spy1552	-	1513678	1514334	218	hypothetica	5479.707	4718.167	0.21587	0.50609	0.70791	1.161404	FALSE
Spy1553	rpsR	1514467	1514706	79	30S riboson	7523.859	11209.95	-0.57524	0.10325	0.27902	-1.48993	FALSE
Spy1554	ssb3	1514871	1515362	163	single-stran	10250.72	16027.77	-0.64485	0.07206	0.22053	-1.56358	FALSE
Spy1555	rpsF	1515384	1515674	96	30S riboson	8177.049	13902.51	-0.76569	0.10315	0.27902	-1.70018	FALSE
Spy1556	-	1515847	1516140	97	hypothetica	336.2821	992.9043	-1.562	6.03E-05	0.001594	-2.95263	TRUE
Spy1557	mutY	1516461	1517462	333	A/G-specifi	1468.878	785.939	0.90223	0.012226	0.067576	1.868953	FALSE
Spy1558	-	1517538	1518125	195	transcriptio	87.53727	112.1442	-0.35739	0.51833	0.72006	-1.28111	FALSE
Spy1559	trx	1518177	1518491	104	thioredoxin	5925.443	4132.502	0.51991	0.16755	0.36962	1.433866	FALSE

Spy1560	-	1518572	1519075	167	phosphatid	1459.545	993.6851	0.55466	0.12902	0.31545	1.468822	FALSE
Spy1561	mutS2	1519076	1521415	779	DNA misma	7091.049	4602.489	0.62358	0.091439	0.25738	1.540694	FALSE
Spy1562	-	1521564	1522109	181	colicin V pr	2048.822	2535.863	-0.30768	0.44328	0.66057	-1.23772	FALSE
Spy1563	-	1522112	1522420	102	hypothetica	818.6798	1290.681	-0.65676	0.069815	0.21607	-1.57654	FALSE
Spy1564	-	1522577	1523479	300	ribonucleas	2104.123	2090.322	0.009494	0.94244	0.98648	1.006602	FALSE
Spy1565	spi	1523490	1524083	197	signal pept	2402.478	2479.627	-0.0456	0.99179	1	-1.03211	FALSE
Spy1566	recD	1524141	1526594	817	exodeoxyri	4231.63	3560.254	0.24923	0.49259	0.7018	1.188573	FALSE
Spy1567	-	1526685	1527167	160	hypothetica	2796.199	4490.433	-0.68339	0.2986	0.52879	-1.60591	FALSE
Spy1568	dinP	1527260	1528354	364	DNA polym	1750.492	1562.453	0.16395	0.67326	0.832	1.12035	FALSE
Spy1569	pfl	1528563	1530890	775	formate ac	21046.71	33866.41	-0.68626	0.25626	0.48653	-1.60911	FALSE
Spy1570	-	1531068	1532018	316	penicillin-b	12608.95	16128.16	-0.35513	0.48332	0.69487	-1.2791	FALSE
Spy1571	-	1532003	1532755	250	c3-degradir	8986.279	13472.35	-0.58421	0.30953	0.53872	-1.49922	FALSE
Spy1572	-	1533053	1533949	298	hypothetica	1407.035	3934.538	-1.4835	3.77E-05	0.001074	-2.79626	TRUE
Spy1573	glpF.2	1534285	1535133	282	aquaporin	4351.327	10399.16	-1.2569	0.090557	0.25613	-2.38982	FALSE
Spy1574	-	1535303	1535449	48	universal st	836.4223	1495.88	-0.83869	0.25974	0.49095	-1.78843	FALSE
Spy1575	norA	1535635	1536831	398	quinolone r	5213.962	7597.17	-0.54308	0.49383	0.70195	-1.45708	FALSE
Spy1576	srv	1536937	1537656	239	Crp/Fnr fan	779.7964	1010.616	-0.37406	0.31111	0.53975	-1.29599	FALSE
Spy1577	pepXP	1537678	1539960	760	x-prolyl-dip	8120.442	8136.761	-0.0029	0.94428	0.98689	-1.00201	FALSE
Spy1578	-	1540040	1540261	73	Cro/CI fami	11.43097	41.31727	-1.8538	0.064277	0.20605	-3.61451	FALSE
Spy1579	-	1540431	1540805	124	transcriptic	329.9648	537.8072	-0.70478	0.13539	0.32793	-1.6299	FALSE
Spy1580	-	1540998	1541297	99	hypothetica	1400.415	2213.773	-0.66065	0.41949	0.63656	-1.58079	FALSE
Spy1581	-	1541354	1542091	245	MerR famil	647.5734	829.5661	-0.35731	0.29584	0.52646	-1.28104	FALSE
Spy1582	dnaQ	1542240	1542827	195	DNA polym	399.5975	550.0545	-0.46103	0.2669	0.49887	-1.37652	FALSE
Spy1583	-	1542876	1543406	176	hypothetica	1294.433	1640.627	-0.34193	0.37794	0.60098	-1.26745	FALSE
Spy1584	-	1543612	1544781	389	NAD(FAD)-(	2418.765	2394.079	0.0148	0.93049	0.98276	1.010311	FALSE
Spy1585	deoC	1544876	1545547	223	deoxyribos	4343.058	3071.066	0.49997	0.1319	0.32121	1.414184	FALSE
Spy1586	nupC	1545577	1546779	400	nucleoside	5754.639	4641.922	0.31	0.35801	0.58253	1.239708	FALSE
Spy1587	udp	1546800	1547579	259	uridine pho	3200.124	1982.378	0.6909	0.039481	0.14861	1.61429	FALSE
Spy1588	-	1547644	1547745	33	hypothetica	NA	NA	NA	NA	NA	NA	Na
Spy1589	crgR	1547819	1548562	247	GntR family	1641.522	2045.014	-0.31708	0.38015	0.60098	-1.24581	FALSE
Spy1590	rpsN	1548798	1549067	89	30S ribosor	572.318	2078.15	-1.8604	0.06575	0.20966	-3.63108	FALSE
Spy1591	gcp	1549238	1550266	342	DNA-bindir	5278.05	1727.334	1.6115	6.82E-06	0.000328	3.055694	TRUE
Spy1592	-	1550256	1550711	151	ribosomal-|	889.4312	378.2086	1.2337	0.001814	0.017785	2.351693	TRUE
Spy1593	-	1550683	1551381	232	glycoprotea	3134.493	1422.837	1.1395	0.002123	0.01976	2.203047	TRUE
Spy1594	-	1551666	1551896	76	hypothetica	2520.257	1860.725	0.43771	0.25264	0.48442	1.354453	FALSE
Spy1595	-	1551898	1553580	560	Zn-depend	36704.19	28451.2	0.36745	0.22178	0.4465	1.290071	FALSE
Spy1596	glnA	1553807	1555153	448	glutamine s	19166.86	8709.397	1.138	0.00061	0.008019	2.200757	TRUE
Spy1597	-	1555191	1555562	123	MerR famil	3750.945	1342.131	1.4827	1.64E-05	0.00064	2.794713	TRUE

Spy1598	-	1555629	1556180	183	hypothetica	359.7729	451.0706	-0.32627	0.47539	0.68865	-1.25377	FALSE
Spy1599	pgk	1556443	1557639	398	phosphogly	23132.69	26870.09	-0.21607	0.81388	0.91243	-1.16157	FALSE
Spy1600	lppC	1557832	1558686	284	acid phospl	9904.865	8257.731	0.26239	0.35302	0.57656	1.199464	FALSE
Spy1601	-	1558916	1559806	296	membrane	6940.913	4976.945	0.47987	0.16964	0.37204	1.394618	FALSE
Spy1602	-	1560043	1561707	554	kinase	16644.64	11447.99	0.53996	0.1043	0.28018	1.453932	FALSE
Spy1603	asp	1561707	1562072	121	alkaline-sho	1295.458	1008.843	0.36076	0.30858	0.53806	1.284102	FALSE
Spy1604	-	1562097	1562234	45	hypothetica	0	0	NA	NA	NA	NA	NA
Spy1605	-	1562239	1563372	377	transposase	0	0	NA	NA	NA	NA	NA
Spy1606	rpmB	1563550	1563738	62	50S ribosor	4016.669	12723.41	-1.6634	0.007483	0.048432	-3.16762	TRUE
Spy1607	fba	1564120	1565001	293	fructose-bis	37295.26	20930.81	0.83336	0.011932	0.066355	1.78183	FALSE
Spy1608	-	1565347	1566273	308	alpha/beta	4108.844	7879.227	-0.93932	0.005922	0.041225	-1.91762	TRUE
Spy1609	-	1566441	1567895	484	CTP synthe	NA	NA	NA	NA	NA	NA	Na
Spy1610	pyrG	1567892	1568044	50	CTP syntha	4923.575	11783.69	-1.259	0.003282	0.027264	-2.3933	TRUE
Spy1611	rpoE	1568301	1568876	191	DNA-direct	4413.848	5184.034	-0.23204	0.76211	0.87659	-1.17449	FALSE
Spy1612	tig	1569093	1570376	427	trigger fact	18113.08	13906.6	0.38126	0.27425	0.50682	1.302479	FALSE
Spy1613	-	1570697	1571542	281	mechanose	414.761	347.3124	0.25605	0.46737	0.68583	1.194205	FALSE
Spy1614	-	1571607	1572167	186	hypothetica	1967.432	1242.903	0.6626	0.068995	0.21549	1.582933	FALSE
Spy1615	-	1572181	1572651	156	hypothetica	693.6386	335.8951	1.0462	0.008892	0.053529	2.065083	FALSE
Spy1616	thiD	1572641	1573405	254	phosphome	1407.935	970.525	0.53674	0.14232	0.33758	1.450691	FALSE
Spy1617	truA	1573395	1574144	249	tRNA pseuc	1894.297	1064.623	0.83132	0.01934	0.091627	1.779313	FALSE
Spy1618	comX.2	1574329	1574880	183	competenc	1.60957	0.280551	2.5203	0.8186	0.91588	5.737014	FALSE
Spy1619	-	1580302	1580436	44	hypothetica	NA	NA	NA	NA	NA	NA	Na
Spy1620	-	1580598	1581740	380	glycerate ki	512.6024	846.0062	-0.72283	0.049903	0.17404	-1.65042	FALSE
Spy1621	hsdR	1582022	1585000	992	type I restri	2692.044	1865.475	0.52916	0.15759	0.3553	1.443089	FALSE
Spy1622	hsdS	1585013	1586212	399	type I restri	1073.02	933.7012	0.20064	0.63287	0.8118	1.149208	FALSE
Spy1623	hsdM	1586225	1587805	526	type I restri	2780.118	2757.517	0.011776	0.91466	0.97336	1.008196	FALSE
Spy1624	-	1588015	1588206	63	hypothetica	376.1501	699.0871	-0.89416	0.040161	0.15042	-1.85853	FALSE
Spy1625	salR	1588358	1588963	201	transcriptio	666.7045	1132.623	-0.76455	0.07674	0.22927	-1.69884	FALSE
Spy1626	-	1588944	1590506	520	sensory tra	983.9528	1533.999	-0.64064	0.13775	0.3306	-1.55902	FALSE
Spy1627	salY	1590546	1592453	635	ABC transp	652.7173	1428.442	-1.1299	0.008936	0.053613	-2.18844	FALSE
Spy1628	-	1592455	1593192	245	ABC transp	34.85558	186.9736	-2.4234	3.86E-05	0.001083	-5.36434	TRUE
Spy1629	salX	1593189	1593650	153	lantibiotic t	18.48337	103.2173	-2.4814	0.000677	0.008414	-5.58439	TRUE
Spy1630	salB	1593724	1595349	541	serine (thre	61.45139	454.9481	-2.8882	1.90E-09	3.85E-07	-7.40346	TRUE
Spy1631	salA	1595432	1595578	48	lantibiotic s	248.9203	2422.738	-3.2829	0.010297	0.060003	-9.7331	FALSE
Spy1632	lacG	1596090	1597496	468	6-phospho-	1601.101	13807.2	-3.1083	0.008672	0.05255	-8.62366	FALSE
Spy1633	lacE	1597584	1599281	565	PTS system	3745.921	34594.47	-3.2071	0.012901	0.071094	-9.23492	FALSE
Spy1634	lacF	1599281	1599598	105	PTS system	187.4976	1224.534	-2.7073	0.010828	0.062301	-6.53098	FALSE
Spy1635	lacD.2	1599622	1600605	327	tagatose 1,	1325.582	6838.631	-2.3671	0.011654	0.065405	-5.15903	FALSE

Spy1636	lacC.2	1600609	1601538	309	tagatose-6-	954.1652	3151.007	-1.7235	0.001534	0.015543	-3.30237	TRUE
Spy1637	lacB.2	1601586	1602101	171	galactose-6	819.8826	2846.668	-1.7958	0.014445	0.076593	-3.47208	FALSE
Spy1638	lacA.2	1602136	1602564	142	galactose-6	651.5443	2425.974	-1.8966	0.01878	0.08973	-3.72335	FALSE
Spy1639	lacR.2	1603011	1603784	257	lactose pho	790.7521	1311.755	-0.7302	0.18997	0.40212	-1.65887	FALSE
Spy1640	-	1604445	1604732	95	DNA-damag	135.811	262.7694	-0.9522	0.049438	0.17375	-1.93482	FALSE
Spy1641	-	1604722	1605057	111	hypothetica	267.316	472.2637	-0.82105	0.055239	0.18693	-1.76669	FALSE
Spy1642	-	1605209	1605391	60	DNA integr	0.608868	3.494131	-2.5207	0.60806	0.79222	-5.7386	FALSE
Spy1643	-	1605479	1605679	66	DNA integr	NA	NA	NA	NA	NA	NA	Na
Spy1644	-	1605380	1605565	61	hypothetica	NA	NA	NA	NA	NA	NA	Na
Spy1645	-	1606013	1606162	49	DNA integr	NA	NA	NA	NA	NA	NA	Na
Spy1646	rpsI	1606282	1606674	130	30S ribosor	13575.59	14129.97	-0.05774	0.86507	0.94258	-1.04084	FALSE
Spy1647	rplM	1606695	1607141	148	50S ribosor	12143.41	13833.11	-0.18795	0.61373	0.79676	-1.13914	FALSE
Spy1648	-	1607359	1607565	68	Cro/CI fami	187.6129	269.3033	-0.52147	0.2385	0.46907	-1.43542	FALSE
Spy1649	-	1607562	1608068	168	hypothetica	346.9984	578.3346	-0.73698	0.066827	0.21183	-1.66668	FALSE
Spy1650	-	1608204	1609064	286	degV family	2805.658	3739.789	-0.41462	0.32205	0.54806	-1.33295	FALSE
Spy1651	-	1609161	1609679	172	hypothetica	2555.175	2342.338	0.12547	0.68917	0.84309	1.090863	FALSE
Spy1652	-	1609683	1610429	248	23S rRNA m	3856.52	3713.989	0.05433	0.83659	0.92762	1.038377	FALSE
Spy1653	-	1610476	1611273	265	hypothetica	778.6989	765.1695	0.025286	0.95845	0.99217	1.017681	FALSE
Spy1654	-	1611469	1611882	137	hypothetica	1341.514	1211.05	0.1476	0.69302	0.84383	1.107725	FALSE
Spy1655	cysS	1611875	1613218	447	cysteinyl-tR	3902.993	3178.82	0.29609	0.42489	0.64111	1.227812	FALSE
Spy1656	-	1613246	1613476	76	hypothetica	159.3094	113.8304	0.48495	0.37902	0.60098	1.399537	FALSE
Spy1657	-	1613489	1613761	90	hypothetica	2399.064	1598.441	0.58581	0.11834	0.30402	1.500881	FALSE
Spy1658	cysE	1613955	1614536	193	serine acet	6392.022	4115.122	0.63534	0.081244	0.23635	1.553304	FALSE
Spy1659	-	1614545	1615297	250	hypothetica	3833.707	2251.018	0.76816	0.034961	0.13714	1.703096	FALSE
Spy1660	-	1615290	1617422	710	polynucleo	17486.77	9119.521	0.93923	0.009269	0.055429	1.917505	FALSE
Spy1661	-	1617703	1618431	242	translaldola	89.33743	224.5121	-1.3295	0.007637	0.048627	-2.51316	TRUE
Spy1662	ulaA	1618587	1619822	411	PTS system	126.4836	280.111	-1.147	0.024411	0.1086	-2.21453	FALSE
Spy1663	-	1619849	1620133	94	PTS system	6.123026	8.824497	-0.52727	0.79543	0.89893	-1.4412	FALSE
Spy1664	-	1620126	1622186	686	PTS system	155.2716	421.6889	-1.4414	0.024092	0.10771	-2.71584	FALSE
Spy1665	-	1622411	1622554	47	hypothetica	NA	NA	NA	NA	NA	NA	Na
Spy1666	rpsO	1622538	1622807	89	30S ribosor	9277.159	18130.32	-0.96665	0.011415	0.064662	-1.9543	FALSE
Spy1667	-	1622965	1623120	51	hypothetica	39.08813	214.4926	-2.4561	0.21154	0.43346	-5.48731	FALSE
Spy1668	-	1623246	1623515	89	transcriptio	1.392534	4.973223	-1.8365	0.576	0.76632	-3.57143	FALSE
Spy1669	def	1623616	1624230	204	peptide def	2344.647	2272.994	0.044777	0.8163	0.91401	1.031524	FALSE
Spy1670	-	1624264	1624806	180	oxidoreduc	2666.085	2825.375	-0.08372	0.77179	0.88331	-1.05975	FALSE
Spy1671	-	1624937	1625365	142	MarR famil	2121.334	1643.59	0.36812	0.32454	0.54887	1.29067	FALSE
Spy1672	polC	1625475	1629872	1465	DNA polym	18594.53	14911.9	0.31841	0.42542	0.64111	1.246956	FALSE
Spy1673	proS	1630127	1631983	618	prolyl-tRNA	23061.08	19763.67	0.22261	0.52973	0.72873	1.166843	FALSE

Spy1674	-	1632181	1633440	419	pheromone	10863.45	8254.894	0.39616	0.23552	0.46611	1.316	FALSE
Spy1675	cdsA	1633513	1634307	264	phosphatid	3423.39	3237.941	0.080349	0.79902	0.90186	1.057274	FALSE
Spy1676	uppS	1634320	1635069	249	undecaprer	3269.687	2797.221	0.22516	0.47299	0.68695	1.168907	FALSE
Spy1677	yajC	1635288	1635653	121	preprotein	7428.694	6318.923	0.23343	0.483	0.69487	1.175627	FALSE
Spy1678	-	1635769	1636116	115	thioredoxir	4569.176	1898.305	1.2672	0.00018	0.003609	2.40694	TRUE
Spy1679	-	1636258	1638939	893	pullulanase	NA	NA	NA	NA	NA	NA	Na
Spy1680	pulA	1638902	1639783	293	pullulanase	2591.797	3246.131	-0.32477	0.38737	0.60653	-1.25246	FALSE
Spy1681	dexB	1639954	1641567	537	glucan 1,6-	1230.377	1751.178	-0.50923	0.21297	0.43443	-1.42329	FALSE
Spy1682	msmK	1641696	1642829	377	multiple su	4994.841	4020.674	0.313	0.34371	0.56582	1.242288	FALSE
Spy1683	lrp	1643127	1643975	282	hypothetica	1697.388	1999.171	-0.23609	0.49926	0.70332	-1.1778	FALSE
Spy1684	ska	1644335	1645657	440	streptokina	1093.04	2001.682	-0.87287	0.015772	0.079648	-1.8313	FALSE
Spy1685	-	1645755	1646198	147	D-tyrosyl-tF	2457.219	2072.426	0.24571	0.49205	0.70172	1.185676	FALSE
Spy1686	relA	1646213	1648432	739	GTP pyroph	10239.43	7886.572	0.37666	0.29818	0.52855	1.298333	FALSE
Spy1687	sclA	1648684	1648827	47	hypothetica	32.05121	128.492	-2.0032	0.010665	0.061559	-4.00888	FALSE
Spy1688	-	1649246	1649473	75	immunoglo	NA	NA	NA	NA	NA	NA	Na
Spy1689	-	1649525	1649731	68	hypothetica	NA	NA	NA	NA	NA	NA	Na
Spy1690	-	1650113	1650595	160	flavoprotei	4897.509	4609.951	0.087297	0.71026	0.85232	1.062378	FALSE
Spy1691	-	1650989	1651807	272	exodeoxyri	169.9972	659.43	-1.9557	3.09E-06	0.000176	-3.87904	TRUE
Spy1692	-	1651890	1653785	631	PTS system	1810.418	4121.952	-1.187	0.000821	0.009789	-2.27679	TRUE
Spy1693	-	1653782	1654075	97	PTS system	NA	NA	NA	NA	NA	NA	Na
Spy1694	-	1654433	1655182	249	16S ribosor	1367.733	1718.873	-0.32968	0.31855	0.5466	-1.25673	FALSE
Spy1695	prmA	1655182	1656135	317	50S ribosor	1766.861	1673.182	0.078594	0.96082	0.9922	1.055988	FALSE
Spy1696	-	1656206	1656676	156	hypothetica	931.2953	683.0554	0.44724	0.2552	0.4864	1.363429	FALSE
Spy1697	-	1656876	1658633	585	para-aminc	3921.708	2385.247	0.71734	0.053807	0.18352	1.644148	FALSE
Spy1698	trpG	1658666	1659232	188	anthranilat	1137.274	583.8879	0.96182	0.01614	0.080657	1.947766	FALSE
Spy1699	-	1659265	1660533	422	recombinat	3452.222	2337.057	0.56283	0.13947	0.33297	1.477164	FALSE
Spy1700	-	1661030	1661470	146	acetyltrans	231.596	681.5537	-1.5572	0.013245	0.07255	-2.94282	FALSE
Spy1701	flaR	1661525	1662031	168	topology m	418.5873	1442.217	-1.7847	1.51E-06	9.84E-05	-3.44547	TRUE
Spy1702	smeZ	1662280	1662981	233	mitogenic e	205.0561	327.7359	-0.67651	0.10545	0.28287	-1.59827	FALSE
Spy1703	-	1663265	1663531	88	hypothetica	5.900874	11.85938	-1.007	0.52109	0.7217	-2.00973	FALSE
Spy1704	dppA	1663710	1665338	542	dipeptide-b	3649.41	3482.775	0.067426	0.87048	0.94509	1.047845	FALSE
Spy1705	dppB	1665451	1666428	325	dipeptide t	311.7919	414.8803	-0.41211	0.2724	0.50493	-1.33063	FALSE
Spy1706	dppC	1666425	1667246	273	dipeptide t	244.3373	410.8257	-0.74965	0.069787	0.21607	-1.68138	FALSE
Spy1707	dppD	1667258	1668061	267	dipeptide t	321.6783	434.7523	-0.43457	0.38055	0.60098	-1.35151	FALSE
Spy1708	dppE	1668045	1668671	208	dipeptide t	289.1933	501.2401	-0.79347	0.043826	0.15987	-1.73324	FALSE
Spy1709	-	1668752	1668952	66	hypothetica	597.3465	433.0812	0.46393	0.3534	0.57656	1.379294	FALSE
Spy1710	-	1669121	1671598	825	histidine tri	2837.68	836.1146	1.7629	1.49E-06	9.84E-05	3.393796	TRUE
Spy1711	lmb	1671611	1672531	306	laminin bin	785.755	232.6521	1.7559	1.10E-05	0.000474	3.377369	TRUE

Spy1712	-	1672709	1673842	377	transposase	0	0	NA	NA	NA	NA	NA
Spy1713	-	1673896	1674048	50	hypothetica	NA	NA	NA	NA	NA	NA	Na
Spy1714	-	1674093	1675160	355	cell surface	2438.264	4890.635	-1.0042	0.046345	0.16478	-2.00583	FALSE
Spy1715	scpA	1675257	1678751	1164	C5A peptid	4205.383	8625.823	-1.0364	0.19829	0.4143	-2.0511	FALSE
Spy1716	-	1679185	1680390	401	transposase	1834.851	1561.139	0.23306	0.47118	0.68695	1.175325	FALSE
Spy1717	-	1680369	1680551	60	transposase	NA	NA	NA	NA	NA	NA	Na
Spy1718	sic1.01	1680969	1681910	313	inhibitor of	2524.471	7205.937	-1.5132	3.60E-05	0.001046	-2.85442	TRUE
Spy1719	emm1.0	1682099	1683553	484	M protein	2170.981	4759.528	-1.1325	0.044009	0.15987	-2.19238	FALSE
Spy1720	mga	1683738	1685327	529	trans-acting	2004.715	3316.62	-0.72632	0.031046	0.12725	-1.65441	FALSE
Spy1721	-	1685712	1685897	61	hypothetica	2.685684	9.207456	-1.7775	0.42565	0.64111	-3.42832	FALSE
Spy1722	-	1686003	1686254	83	hypothetica	633.5548	1137.498	-0.84432	0.36486	0.58999	-1.79542	FALSE
Spy1723	isp	1686333	1687934	533	hypothetica	6719.599	5979.404	0.16837	0.82651	0.92036	1.123788	FALSE
Spy1724	ihk	1688036	1689424	462	two compo	4141.727	3160.268	0.39019	0.5464	0.73934	1.310566	FALSE
Spy1725	irr	1689421	1690074	217	two-compo	1812.246	1417.097	0.35484	0.59715	0.78247	1.278844	FALSE
Spy1726	-	1690168	1691385	405	ABC transp	3167.691	3238.465	-0.03188	0.85724	0.93942	-1.02234	FALSE
Spy1727	-	1691398	1692072	224	ABC transp	792.3172	627.3369	0.33684	0.73515	0.86073	1.262987	FALSE
Spy1728	-	1692059	1693327	422	periplasmic	2321.944	1519.006	0.61221	0.40326	0.62019	1.528599	FALSE
Spy1729	-	1693750	1694154	134	hypothetica	NA	NA	NA	NA	NA	NA	Na
Spy1730	-	1694181	1694477	98	hypothetica	259.6827	260.3994	-0.00398	0.96119	0.9922	-1.00276	FALSE
Spy1731	-	1694721	1694957	78	hypothetica	78.71015	920.6253	-3.548	5.93E-06	0.0003	-11.6965	TRUE
Spy1732	prsA	1695233	1696162	309	foldase Prs	123314.1	35170.29	1.8099	1.05E-07	1.47E-05	3.50618	TRUE
Spy1733	-	1696218	1696535	105	hypothetica	20783.96	6288.268	1.7247	6.29E-07	4.99E-05	3.305114	TRUE
Spy1734	-	1696642	1696947	101	hypothetica	56653.46	30102.72	0.91227	0.005512	0.039123	1.882004	TRUE
Spy1735	speB	1696949	1698145	398	exotoxin B	310490.7	139038.2	1.1591	0.000473	0.006846	2.233181	TRUE
Spy1736	-	1698670	1698801	43	hypothetica	75.99772	177.8404	-1.2266	0.025559	0.11153	-2.34015	FALSE
Spy1737	rgg	1699085	1699927	280	transcriptio	2851.335	2701.288	0.07799	0.70333	0.8486	1.055546	FALSE
Spy1738	spd	1700168	1700983	271	phage-asso	73034.45	58783.23	0.31317	0.33165	0.55359	1.242435	FALSE
Spy1739	-	1701067	1701195	42	hypothetica	NA	NA	NA	NA	NA	NA	Na
Spy1740	-	1701347	1701856	169	low temper	604.1001	622.1195	-0.0424	0.96523	0.99479	-1.02983	FALSE
Spy1741	gldA	1701938	1703026	362	glycerol de	2922.829	3953.232	-0.43567	0.22555	0.45011	-1.35254	FALSE
Spy1742	mipB	1703083	1703751	222	fructose-6-	990.9175	1345.215	-0.441	0.21298	0.43443	-1.35754	FALSE
Spy1743	pflD	1703764	1706136	790	formate ac	3686.277	5215.377	-0.50061	0.12418	0.31056	-1.41481	FALSE
Spy1744	-	1706391	1707695	434	PTS system	406.7933	1023.874	-1.3317	0.000566	0.007597	-2.51699	TRUE
Spy1745	-	1707705	1708013	102	PTS system	14.31147	50.29408	-1.8132	0.044851	0.162	-3.51421	FALSE
Spy1746	-	1708041	1708361	106	PTS system	56.00928	183.0968	-1.7089	0.002013	0.019124	-3.26911	TRUE
Spy1747	-	1708649	1709629	326	sorbitol op	2440.452	5885.174	-1.2699	0.000274	0.004758	-2.41145	TRUE
Spy1748	-	1709645	1710394	249	DeoR famil	1793.957	3541.834	-0.98135	0.00453	0.033915	-1.97431	TRUE
Spy1749	-	1710517	1711290	257	pyruvate fo	1346.046	4866.659	-1.8542	2.23E-07	2.60E-05	-3.61551	TRUE

Spy1750	-	1711325	1711525	66	hypothetica	58.64864	418.3477	-2.8345	2.71E-05	0.000881	-7.13296	TRUE
Spy1751	secE	1711527	1711703	58	preprotein	447.7955	832.972	-0.89543	0.013803	0.07478	-1.86016	FALSE
Spy1752	rpmG	1711717	1711869	50	50S ribosor	133.9131	162.5158	-0.27928	0.60774	0.79222	-1.21359	FALSE
Spy1753	pbp2A	1711918	1714254	778	multimodu	23101.91	13859.98	0.73709	0.02721	0.11596	1.66681	FALSE
Spy1754	-	1714293	1714673	126	translation	413.4427	208.726	0.98608	0.020015	0.093848	1.980796	FALSE
Spy1755	-	1715164	1715355	63	hypothetica	693.0242	810.9049	-0.22663	0.74238	0.86481	-1.1701	FALSE
Spy1756	-	1715249	1716250	333	ribosomal l	3552.761	3622.213	-0.02793	0.97796	0.99967	-1.01955	FALSE
Spy1757	-	1716339	1717979	546	hypothetica	3962.33	8698.13	-1.1344	0.064603	0.20673	-2.19527	FALSE
Spy1758	-	1718226	1719722	498	dipeptidase	4707.811	6825.909	-0.53596	0.13977	0.33324	-1.44991	FALSE
Spy1759	-	1720207	1720371	54	MutR famil	NA	NA	NA	NA	NA	NA	Na
Spy1760	-	1720350	1720532	60	MutR famil	NA	NA	NA	NA	NA	NA	Na
Spy1761	groEL	1720925	1722556	543	molecular c	69661.7	104988.5	-0.59179	0.068719	0.21524	-1.50712	FALSE
Spy1762	groES	1722592	1722882	96	co-chapero	11345.26	20090.37	-0.82441	0.014046	0.075134	-1.77081	FALSE
Spy1763	clpC	1723060	1725504	814	negative re	10218.77	16766.62	-0.71437	0.24564	0.478	-1.64077	FALSE
Spy1764	ctsR	1725504	1725965	153	transcriptic	1036.263	1363.057	-0.39546	0.42087	0.63709	-1.31536	FALSE
Spy1765	csp	1726161	1726370	69	cold shock	6355.805	9742.988	-0.61629	0.08086	0.2356	-1.53293	FALSE
Spy1766	-	1726507	1726611	34	hypothetica	NA	NA	NA	NA	NA	NA	Na
Spy1767	-	1726595	1727728	377	transposase	0	0	NA	NA	NA	NA	NA
Spy1768	ahpC	1728685	1729245	186	peroxiredo:	18598.05	19595.58	-0.07538	0.96823	0.99609	-1.05364	FALSE
Spy1769	ahpF	1729266	1730798	510	peroxiredo:	74274.16	66204.5	0.16593	0.55761	0.74951	1.121889	FALSE
Spy1770	hutI	1730856	1732121	421	imidazolon	228.168	210.1332	0.11879	0.94497	0.98689	1.085824	FALSE
Spy1771	hutU	1732413	1734443	676	urocanate l	53.48524	171.8241	-1.6837	0.28843	0.51984	-3.21251	FALSE
Spy1772	-	1734532	1735431	299	glutamate f	15.80047	53.73707	-1.766	0.26723	0.49887	-3.4011	FALSE
Spy1773	-	1735442	1736068	208	formiminot	10.61193	38.84321	-1.872	0.32909	0.55129	-3.6604	FALSE
Spy1774	fhs.2	1736086	1737759	557	formate--te	100.034	169.0669	-0.7571	0.40952	0.6256	-1.69009	FALSE
Spy1775	-	1737781	1738377	198	hypothetica	54.98622	77.04942	-0.48671	0.52509	0.72393	-1.40125	FALSE
Spy1776	-	1738597	1739940	447	amino acid	147.8195	174.9587	-0.24318	0.67553	0.83385	-1.1836	FALSE
Spy1777	hutH	1739951	1741492	513	histidine ar	253.0108	254.5832	-0.00894	0.87396	0.94718	-1.00621	FALSE
Spy1778	hutG	1741678	1742664	328	formimidoy	2929.257	2773.434	0.078862	0.80059	0.90308	1.056185	FALSE
Spy1779	-	1742695	1745769	1024	LuxR family	770.8424	1512.581	-0.9725	0.009901	0.058258	-1.96224	FALSE
Spy1780	rpsB	1746073	1746840	255	30S ribosor	32123.69	35543.7	-0.14596	0.726	0.85822	-1.10647	FALSE
Spy1781	tsf	1746974	1748014	346	elongation	35299.64	28934.15	0.28688	0.35331	0.57656	1.219999	FALSE
Spy1782	pepO	1748180	1750075	631	neutral enc	3213.114	3984.182	-0.31031	0.38392	0.60368	-1.23997	FALSE
Spy1783	dexS	1750283	1751911	542	trehalose-6	535.2764	5933.094	-3.4704	1.57E-11	4.78E-09	-11.0839	TRUE
Spy1784	-	1751978	1754002	674	PTS system	1174.115	12187.54	-3.3758	2.49E-17	2.27E-14	-10.3805	TRUE
Spy1785	-	1754213	1754926	237	trehalose o	102.413	218.6228	-1.094	0.032672	0.13243	-2.13465	FALSE
Spy1786	-	1755205	1755372	55	MarR famil	NA	NA	NA	NA	NA	NA	Na
Spy1787	-	1755730	1756587	285	glyoxalase	121.8891	168.914	-0.47072	0.35889	0.58344	-1.3858	FALSE

Spy1788	yaaA	1756629	1757360	243	hypothetica	2570.196	2070.949	0.31159	0.34327	0.5656	1.241075	FALSE
Spy1789	nrdG	1757534	1758148	204	anaerobic r	2989.934	4090.11	-0.45203	0.20719	0.42895	-1.36796	FALSE
Spy1790	-	1758148	1758657	169	acetyltrans	1305.681	1518.551	-0.21789	0.54181	0.73641	-1.16303	FALSE
Spy1791	-	1758666	1759601	311	virulence fa	4275.902	5266.516	-0.30062	0.39795	0.6159	-1.23167	FALSE
Spy1792	-	1759630	1759776	48	hypothetica	NA	NA	NA	NA	NA	NA	Na
Spy1793	nrdD	1759958	1762156	732	anaerobic r	19296.09	29488.46	-0.61184	0.079017	0.23284	-1.52821	FALSE
Spy1794	-	1762253	1763812	519	hypothetica	3470.356	10078.03	-1.5381	1.32E-05	0.000537	-2.90412	TRUE
Spy1795	-	1764225	1764530	101	hypothetica	4289.027	4829.828	-0.17132	0.68352	0.83909	-1.12609	FALSE
Spy1796	-	1764542	1764961	139	Holliday jur	4102.945	5045.454	-0.29832	0.42236	0.6388	-1.22971	FALSE
Spy1797	-	1764958	1765227	89	hypothetica	1224.714	1560.335	-0.34941	0.32128	0.54806	-1.27404	FALSE
Spy1798	spxA	1765340	1765738	132	Spx family t	10945.04	21557.87	-0.97794	0.058467	0.19461	-1.96965	FALSE
Spy1799	recA	1766029	1767165	378	recombinas	9278.938	7176.737	0.37063	0.30674	0.53707	1.292917	FALSE
Spy1800	cinA	1767254	1768525	423	competenc	1085.447	995.2877	0.1251	0.80944	0.90969	1.090583	FALSE
Spy1801	tag	1768594	1769154	186	DNA-3-met	1803.048	1475.156	0.28957	0.41503	0.63191	1.222276	FALSE
Spy1802	ruvA	1769164	1769760	198	Holliday jur	1517.488	1203.037	0.335	0.37876	0.60098	1.261377	FALSE
Spy1803	lmrP	1769762	1770982	406	multidrug r	3363.969	3137.742	0.10044	0.83084	0.92244	1.0721	FALSE
Spy1804	mutL	1770993	1772975	660	DNA misma	5752.675	4060.446	0.50259	0.14424	0.34081	1.416755	FALSE
Spy1805	mutS	1773104	1775659	851	DNA misma	4896.247	4988.226	-0.02685	0.93747	0.98523	-1.01879	FALSE
Spy1806	-	1775646	1775852	68	hypothetica	137.0539	248.6139	-0.85916	0.073898	0.22353	-1.81398	FALSE
Spy1807	argR2	1775995	1776432	145	arginine rep	629.2313	1186.623	-0.9152	0.014556	0.076735	-1.88583	FALSE
Spy1808	argS	1776723	1778414	563	arginyl-tRN	7995.879	7359.524	0.11964	0.71846	0.85652	1.086464	FALSE
Spy1809	uviB	1778502	1778810	102	bacteriocin	1021.761	1021.465	0.000418	0.89403	0.95812	1.00029	FALSE
Spy1810	-	1778837	1779709	290	hypothetica	3590.248	3129.262	0.19826	0.54278	0.73663	1.147314	FALSE
Spy1811	-	1779752	1780687	311	hypothetica	3690.087	2836.787	0.3794	0.25612	0.48653	1.300801	FALSE
Spy1812	-	1780650	1781591	313	hypothetica	4132.313	3751.113	0.13963	0.69235	0.84358	1.101623	FALSE
Spy1813	aspS	1781584	1783332	582	aspartyl-tR	7974.681	7292.885	0.12894	0.72484	0.85822	1.09349	FALSE
Spy1814	hisS	1783670	1784950	426	histidyl-tRN	2896.435	3961.22	-0.45167	0.32775	0.55129	-1.36762	FALSE
Spy1815	rpmF	1785170	1785352	60	50S ribosor	1603.243	2942.829	-0.87621	0.28838	0.51984	-1.83555	FALSE
Spy1816	rpmG	1785368	1785517	49	50S ribosor	1502.393	2906.884	-0.95221	0.015616	0.079555	-1.93483	FALSE
Spy1817	cadD	1785810	1786424	204	cadmium re	150.4851	306.9989	-1.0286	0.28064	0.51349	-2.04004	FALSE
Spy1818	cadC	1786406	1786774	122	cadmium e	59.43522	106.5854	-0.84262	0.40652	0.62301	-1.7933	FALSE
Spy1819	-	1786825	1787748	307	hypothetica	395.16	618.0038	-0.64518	0.35953	0.58395	-1.56393	FALSE
Spy1820	-	1787814	1788566	250	DNA transl	485.8159	732.8631	-0.59313	0.30818	0.53806	-1.50852	FALSE
Spy1821	-	1788563	1789165	200	hypothetica	623.8819	984.0148	-0.65741	0.24581	0.478	-1.57725	FALSE
Spy1822	-	1789641	1789925	94	transcriptio	NA	NA	NA	NA	NA	NA	Na
Spy1823	-	1790376	1791218	280	hypothetica	2006.846	2742.267	-0.45044	0.53382	0.73099	-1.36646	FALSE
Spy1824	-	1791265	1791906	213	phosphohy	523.953	423.5234	0.307	0.39516	0.61239	1.237132	FALSE
Spy1825	-	1792113	1792439	108	PadR family	465.6186	712.566	-0.61387	0.13826	0.33096	-1.53036	FALSE

Spy1826	-	1792426	1793013	195	hypothetica	2139.791	5183.994	-1.2766	0.000179	0.003609	-2.42267	TRUE
Spy1827	-	1793010	1794089	359	hypothetica	9476.581	15929.25	-0.74924	0.023115	0.10436	-1.68091	FALSE
Spy1828	-	1794207	1796075	622	phage infec	1861.943	4508.002	-1.2757	0.020948	0.097057	-2.42116	FALSE
Spy1829	-	1796108	1796479	123	phage infec	NA	NA	NA	NA	NA	NA	Na
Spy1830	-	1796614	1797147	177	TetR family	145.1143	443.8795	-1.613	0.000342	0.005279	-3.05887	TRUE
Spy1831	rpsD	1797301	1797912	203	30S ribosor	14246.67	27144.45	-0.93003	0.11821	0.30402	-1.90532	FALSE
Spy1832	-	1798115	1798219	34	hypothetica	NA	NA	NA	NA	NA	NA	Na
Spy1833	-	1798203	1799336	377	transposase	0	0	NA	NA	NA	NA	NA
Spy1834	-	1799968	1800240	90	hypothetica	624.6522	412.612	0.59827	0.14872	0.34689	1.5139	FALSE
Spy1835	holB	1800257	1801624	455	replicative	9742.182	6222.01	0.64686	0.059019	0.19537	1.565757	FALSE
Spy1836	rplI	1801654	1802106	150	50S ribosor	1354.169	853.8568	0.66534	0.078666	0.23218	1.585942	FALSE
Spy1837	-	1802103	1804079	658	phosphoes	8837.116	6032.846	0.55074	0.092344	0.25794	1.464837	FALSE
Spy1838	gidA	1804170	1806068	632	tRNA uridir	8983.612	7005.123	0.35889	0.31067	0.53971	1.282439	FALSE
Spy1839	-	1806192	1806509	105	phosphohy	1640.401	1390.84	0.23809	0.531	0.72877	1.17943	FALSE
Spy1840	mnmA	1807286	1808407	373	tRNA-speci	12731.78	8496.696	0.58346	0.10335	0.27902	1.498439	FALSE
Spy1841	sdhB	1808705	1809376	223	L-serine de	1240.266	2174.265	-0.80988	0.020806	0.097057	-1.75307	FALSE
Spy1842	sdhA	1809388	1810260	290	L-serine de	2083.175	4301.516	-1.0461	0.002273	0.020837	-2.06494	TRUE
Spy1843	-	1810673	1811287	204	transglycos	2751.907	2777.642	-0.01343	0.92304	0.97942	-1.00935	FALSE
Spy1844	cbiQ	1811659	1812459	266	cobalt trans	1474.76	1710.595	-0.21402	0.64356	0.81627	-1.15992	FALSE
Spy1845	cbiO	1812452	1813294	280	cobalt ABC	937.1891	1053.2	-0.16837	0.71337	0.85433	-1.12379	FALSE
Spy1846	cbiO	1813270	1814160	296	cobalt ABC	1885.18	2366.771	-0.32822	0.50027	0.70355	-1.25546	FALSE
Spy1847	-	1814111	1814653	180	CDP-diacylg	703.9967	821.6729	-0.223	0.60772	0.79222	-1.16716	FALSE
Spy1848	-	1814667	1815692	341	hypothetica	2875.09	2406.123	0.2569	0.39811	0.6159	1.194908	FALSE
Spy1849	-	1815742	1817031	429	zinc protea	1376.679	545.2105	1.3363	0.000277	0.004761	2.525029	TRUE
Spy1850	-	1817033	1818277	414	zinc protea	1202.277	542.816	1.1472	0.001481	0.01524	2.214836	TRUE
Spy1851	hasA	1818708	1819967	419	hyaluronan	143.3187	661.4086	-2.2063	0.038395	0.14621	-4.6149	FALSE
Spy1852	hasB	1820003	1821211	402	UDP-glucos	126.3293	295.983	-1.2283	0.025772	0.11168	-2.34291	FALSE
Spy1853	hasC	1821393	1822307	304	UTP-glucos	348.9751	415.8679	-0.253	0.49949	0.70332	-1.19168	FALSE
Spy1854	-	1822615	1823028	137	hypothetica	1060.888	398.2776	1.4134	0.000141	0.003089	2.663642	TRUE
Spy1855	recF	1823030	1824136	368	recombinat	4040.766	1770.605	1.1904	0.000482	0.006881	2.28216	TRUE
Spy1856	-	1824191	1825024	277	glucose upt	3871.66	4339.352	-0.16453	0.79534	0.89893	-1.1208	FALSE
Spy1857	guaB	1825256	1826737	493	inosine 5\'-	17425.33	13072.66	0.41463	0.19586	0.41111	1.332957	FALSE
Spy1858	trsA	1827045	1828067	340	tryptophan	2606.723	1912.374	0.44687	0.157	0.35526	1.36308	FALSE
Spy1859	-	1828159	1828284	41	hypothetica	NA	NA	NA	NA	NA	NA	Na
Spy1860	-	1828486	1829358	290	hypothetica	1065.353	710.8875	0.58364	0.089385	0.25395	1.498626	FALSE
Spy1861	-	1829437	1831056	539	ABC transp	10582.68	6042.577	0.80847	0.013476	0.073375	1.751353	FALSE
Spy1862	-	1831139	1833715	858	ABC transp	1629.879	1485.98	0.13335	0.62076	0.80246	1.096838	FALSE
Spy1863	-	1834881	1835162	93	transposase	NA	NA	NA	NA	NA	NA	Na

Spy1864	-	1835744	1836223	159	rRNA large	926.7595	1719.722	-0.89191	0.22528	0.45011	-1.85563	FALSE
Spy1865	htrA	1836435	1837658	407	protease D	37227.8	21579.48	0.78672	0.014014	0.075134	1.725148	FALSE
Spy1866	parB	1837717	1838523	268	chromosom	11753.05	6147.9	0.93487	0.005607	0.039333	1.911718	TRUE
