# Supplementary material for: Novel Tyrosine Kinase-Mediated Phosphorylation With Dual Specificity Plays a Key Role in the Modulation of Streptococcus pyogenes Physiology and Virulence
Source: Front Microbiol. 2021 Dec 7;12:689246. doi: 10.3389/fmicb.2021.689246 (PMC8689070; doi:10.3389/fmicb.2021.689246)
Supplement: Supplementary file 8 [file Data_Sheet_8.PDF]

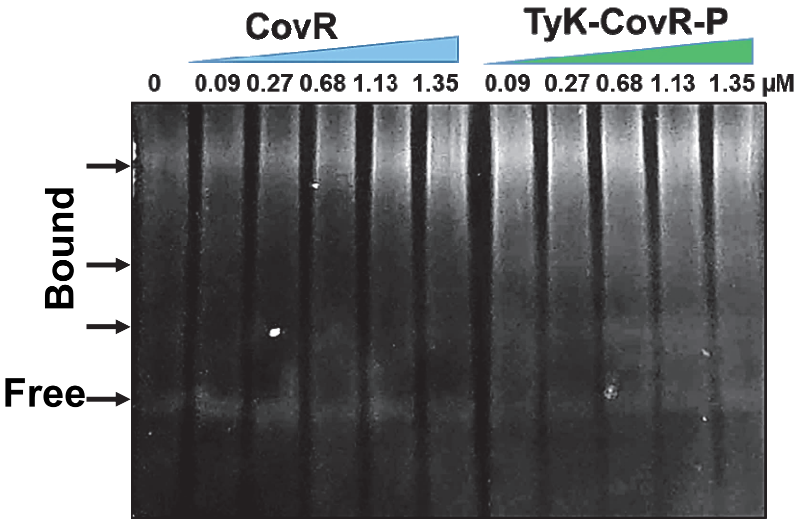

Figure S4: EMSA-based profile of the 6% native gel-resolved bound and free P*covR* preincubated with different concentrations of purified non-phosphorylated CovR and SP-TyK-phosphorylated CovR-P as revealed by SYBR-green stain. (Supporting figure for Fig. 6D).
