# Supplementary material for: Novel Tyrosine Kinase-Mediated Phosphorylation With Dual Specificity Plays a Key Role in the Modulation of Streptococcus pyogenes Physiology and Virulence
Source: Front Microbiol. 2021 Dec 7;12:689246. doi: 10.3389/fmicb.2021.689246 (PMC8689070; doi:10.3389/fmicb.2021.689246)
Supplement: Supplementary file 9 [file Data_Sheet_9.PDF]

Table-S3-Upregulated-DEGS in M1T1 ΔTyK mutant

Gene_id	readcount_D	readcount_W	log2Fold	Linear fold	pval	padj	**Locus tag**	**Locus**	**Start**	**Stop**	**Length**	**Protein name**
Spy0047	30495.872	15002.929	1.0234	2.0327038	0.0044118	0.033252	M5005_Spy	rplB	66412	67245	277	50S ribosomal protein L
Spy0048	9114.2653	3736.4506	1.2865	2.4393555	0.0003127	0.0050922	M5005_Spy	rpsS	67384	67662	92	30S ribosomal protein S1
Spy0049	4172.2156	1666.5657	1.3239	2.5034194	0.0003318	0.0052171	M5005_Spy	rplV	67678	68022	114	50S ribosomal protein L2
Spy0050	23509.501	9612.9932	1.2902	2.4456196	0.0002824	0.0047858	M5005_Spy	rpsC	68035	68688	217	30S ribosomal protein S
Spy0051	11591.794	5358.2566	1.1133	2.1633993	0.0023869	0.021446	M5005_Spy	rplP	68692	69105	137	50S ribosomal protein L1
Spy0053	4117.2527	1909.0913	1.1088	2.1566619	0.0029429	0.02532	M5005_Spy	rpsQ	69347	69607	86	30S ribosomal protein S1
Spy0054	13725.91	6270.1705	1.1303	2.1890426	0.0014873	0.01524	M5005_Spy	rplN	69632	70000	122	50S ribosomal protein L1
Spy0055	9707.8913	4678.5615	1.0531	2.0749837	0.0036899	0.02901	M5005_Spy	rplX	70079	70384	101	50S ribosomal protein L2
Spy0056	26731.751	10708.658	1.3198	2.496315	0.0002002	0.0038061	M5005_Spy	rplE	70408	70950	180	50S ribosomal protein L
Spy0057	7000.4433	3127.4298	1.1625	2.2384499	0.0010531	0.011931	M5005_Spy	rpsN	70966	71151	61	30S ribosomal protein S1
Spy0058	26739.86	11687.423	1.194	2.2878619	0.0006923	0.0084754	M5005_Spy	rpsH	71302	71700	132	30S ribosomal protein S
Spy0059	31366.461	13290.6	1.2388	2.3600215	0.0003846	0.0058461	M5005_Spy	rplF	71903	72439	178	50S ribosomal protein L
Spy0061	17948.056	9469.8198	0.92242	1.8952918	0.0077046	0.048627	M5005_Spy	rpsE	72919	73413	164	30S ribosomal protein S
Spy0065	3834.1856	1830.1183	1.067	2.0950723	0.0023603	0.021419	M5005_Spy	adk	75735	76373	212	adenylate kinase
Spy0068	6037.9232	3185.7698	0.92241	1.8952787	0.0078695	0.049326	M5005_Spy	rpsM	76869	77234	121	30S ribosomal protein S1
Spy0069	11669.025	6227.3416	0.906	1.8738429	0.0080817	0.049748	M5005_Spy	rpsK	77252	77635	127	30S ribosomal protein S1
Spy0070	33538.759	17903.978	0.90555	1.8732585	0.0073937	0.048204	M5005_Spy	rpoA	77681	78619	312	DNA-directed RNA polymerase subunit alph
Spy0083	41481.136	16468.142	1.3328	2.5189107	0.0001673	0.0035069	M5005_Spy	rpoB	94142	97708	1188	DNA-directed RNA polymerase subunit bet
Spy0084	78787.148	36521.076	1.1092	2.1572599	0.0014243	0.015017	M5005_Spy	rpoC	97799	101440	1213	DNA-directed RNA polymerase subunit beta
Spy0085	2184.5805	685.1	1.673	3.1887699	7.47E-06	0.0003405	M5005_Spy	-	101592	101957	121	DNA binding protei
Spy0093	2360.998	1078.596	1.1302	2.1888908	0.0013892	0.014818	M5005_Spy	-	105765	106718	317	adenine-specific methyltransferas
Spy0228	4404.7931	2218.6398	0.9894	1.9853591	0.0046045	0.034141	M5005_Spy	purR	232835	233674	279	pur operon represso
Spy0233	170262.11	92897.332	0.87405	1.8328008	0.0073997	0.048204	M5005_Spy	plr	240191	241201	336	glyceraldehyde-3-phosphate dehydrogenas
Spy0242	4188.0439	1686.3653	1.3124	2.4835435	0.0002101	0.0039509	M5005_Spy	-	249192	249962	256	ABC transporter ATP-binding prote
Spy0243	9125.8418	4096.3413	1.1556	2.2277695	0.0010527	0.011931	M5005_Spy	-	250057	251319	420	ABC transporte
Spy0244	9538.0236	4810.4081	0.98753	1.9827874	0.0041859	0.031946	M5005_Spy	nifS3	251350	252576	408	cysteine desulfhydrase
Spy0245	2138.703	1073.5921	0.99429	1.9920999	0.0055724	0.039333	M5005_Spy	nifU	252563	253042	159	iscU protei
Spy0270	11801.596	2680.9963	2.1381	4.4018195	1.61E-09	3.68E-07	M5005_Spy	-	279938	280780	280	ABC transporter substrate-binding prote
Spy0279	7234.6442	3595.0485	1.0089	2.0123762	0.0027349	0.023868	M5005_Spy	lemA	289896	290453	185	hypothetical protein M5005_Spy_027
Spy0281	4578.5525	1736.78	1.3985	2.6362734	0.0001109	0.0026278	M5005_Spy	-	291630	292163	177	hypothetical protein M5005_Spy_028
Spy0300	6088.7424	2579.1893	1.2392	2.3606759	0.000258	0.0045683	M5005_Spy	-	311311	311811	166	HAD superfamily hydrolase
Spy0301	20859.229	9007.928	1.2114	2.3156224	0.0002646	0.0046414	M5005_Spy	-	311826	312515	229	hypothetical protein M5005_Spy_030
Spy0303	2724.4371	1275.2179	1.0952	2.136427	0.0027281	0.023868	M5005_Spy	glr	313113	313907	264	glutamate racemase
Spy0304	4256.2454	1954.249	1.123	2.177994	0.0022542	0.020766	M5005_Spy	-	313904	314890	328	deoxyribonucleotide triphosphate pyrophosphat
Spy0307	2373.5794	1190.2467	0.9958	1.994186	0.007296	0.04787	M5005_Spy	xerD	315845	316591	248	site-specific tyrosine recombinase Xer
Spy0308	2802.5571	1282.9465	1.1273	2.1844953	0.0023738	0.021435	M5005_Spy	scpA	316591	317292	233	segregation and condensation protein
Spy0309	3701.5384	1835.5268	1.0119	2.0165651	0.0067563	0.04535	M5005_Spy	scpB	317289	317840	183	segregation and condensation protein
Spy0477	4561.8324	2045.801	1.1569	2.2297779	0.0009528	0.011069	M5005_Spy	-	468158	468973	271	hypothetical protein M5005_Spy_047
Spy0478	3370.6448	1596.1176	1.0785	2.1118392	0.0020815	0.01947	M5005_Spy	-	468973	469875	300	hypothetical protein M5005_Spy_047
Spy0530	11581.404	4694.9431	1.3026	2.4667303	0.0001664	0.0035069	M5005_Spy	prfB	518105	519118	337	peptide chain release factor
Spy0531	8211.9512	3551.1904	1.2094	2.3124145	0.0005316	0.0072679	M5005_Spy	ftsE	519137	519829	230	cell division ATP-binding protei
Spy0532	20397.55	10386.601	0.97367	1.9638299	0.0032702	0.027264	M5005_Spy	ftsX	519822	520751	309	cell division protei

Spy0576	13188.236	6387.2953	1.046	2.0647971	0.0020456	0.019329	M5005_Spy_	atpB	575889	576605	238	ATP synthase F0F1 subunit A
Spy0577	9061.6638	3822.003	1.2454	2.3708428	0.0003284	0.0052084	M5005_Spy_	atpF	576623	577117	164	ATP synthase F0F1 subunit B
Spy0579	75288.321	22805.468	1.723	3.3012216	9.09E-07	6.48E-05	M5005_Spy_	atpA	577669	579177	502	ATP synthase F0F1 subunit alpha
Spy0580	29188.851	9535.9357	1.614	3.0609935	4.65E-06	0.0002495	M5005_Spy_	atpG	579193	580068	291	ATP synthase F0F1 subunit gamma
Spy0581	89314.642	26953.801	1.7284	3.3136012	9.23E-07	6.48E-05	M5005_Spy_	atpD	580230	581636	468	ATP synthase F0F1 subunit beta
Spy0582	23260.862	8198.6626	1.5044	2.8370666	1.87E-05	0.0006815	M5005_Spy_	atpC	581649	582065	138	ATP synthase F0F1 subunit epsilon
Spy0591	4259.9346	1738.8932	1.2927	2.4498612	0.0002529	0.0045229	M5005_Spy_	-	589571	590647	358	ABC transporter permease
Spy0592	3065.0123	1263.8076	1.2781	2.4251937	0.0003001	0.0049757	M5005_Spy_	-	590657	591325	222	ABC transporter ATP-binding prote
Spy0658	7917.1727	3772.6014	1.0694	2.0985604	0.0022371	0.020713	M5005_Spy_	-	663448	664506	352	regulatory protein
Spy0659	15967.784	7517.9874	1.0867	2.1238767	0.0016483	0.01634	M5005_Spy_	apbA	664519	665442	307	2-dehydropantoate 2-reductase
Spy0660	1549.6907	782.55579	0.98571	1.9802876	0.006115	0.042249	M5005_Spy_	fruR	665698	666411	237	fructose repressor
Spy0689	2019.3131	763.92923	1.4024	2.6434096	0.0001019	0.0024782	M5005_Spy_	dyr	692181	692678	165	dihydrofolate reductase
Spy0690	495.92239	194.85573	1.3477	2.5450606	0.0020559	0.019329	M5005_Spy_	-	692698	692868	56	hypothetical protein M5005_Spy_0690
Spy0691	9989.7798	4970.1305	1.0072	2.0100063	0.0047933	0.035254	M5005_Spy_	clpX	692998	694227	409	ATP-dependent protease ATP-binding subunit Cl
Spy0695	11504.192	5681.0423	1.0179	2.0249692	0.0032997	0.027264	M5005_Spy_	rpiA	698263	698946	227	ribose-5-phosphate isomerase A
Spy0696	23584.161	12641.717	0.89963	1.8655875	0.0079267	0.04938	M5005_Spy_	deoB	699023	700234	403	phosphopentomutase
Spy0697	5467.3715	2881.0171	0.92427	1.8977238	0.0074878	0.048432	M5005_Spy_	arsC	700253	700693	146	arsenate reductase
Spy0698	14568.567	7327.1469	0.99154	1.9883063	0.0033447	0.027264	M5005_Spy_	punA	700677	701486	269	purine nucleoside phosphorylas
Spy0699	17358.864	8938.3068	0.9576	1.9420765	0.0037584	0.029296	M5005_Spy_	deoD	702149	702862	237	purine nucleoside phosphorylas
Spy0700	5799.5477	3029.8986	0.93667	1.914105	0.005325	0.038023	M5005_Spy_	cpsX	702855	703643	262	LytR family transcriptional regulato
Spy0716	3628.8819	1241.3557	1.5476	2.9233043	7.13E-06	0.0003334	M5005_Spy_	-	721453	721755	100	hypothetical protein M5005_Spy_0716
Spy0717	6625.9448	2902.479	1.1908	2.2827929	0.0006754	0.0084143	M5005_Spy_	-	721954	722940	328	hypothetical protein M5005_Spy_0717
Spy0751	13006.5	5187.11	1.3262	2.5074136	0.0001184	0.0027344	M5005_Spy_	acoA	753149	754117	322	pyruvate dehydrogenase E1 component subunit
Spy0752	22551.65	10280.627	1.1333	2.1935993	0.0009711	0.011211	M5005_Spy_	acoB	754173	755174	333	pyruvate dehydrogenase E1 component subunit
Spy0753	36077.064	16911.999	1.093	2.1331716	0.0013284	0.01445	M5005_Spy_	acoC	755359	756768	469	branched-chain alpha-keto acid dehydrogenase s
Spy0755	51569.778	26286.936	0.97218	1.9618028	0.0038474	0.029862	M5005_Spy_	acoL	757095	758858	587	dihydrolipoamide dehydrogenase
Spy0765	3012.7657	1232.6292	1.2893	2.4440944	0.0001949	0.0037817	M5005_Spy_	hemN	769506	770702	398	coproporphyrinogen III oxidase
Spy0766	2217.901	812.45574	1.4488	2.729809	4.70E-05	0.0012599	M5005_Spy_	-	770712	771464	250	acyl-ACP thioesterase
Spy0767	2661.7931	1328.7691	1.0023	2.003191	0.0043535	0.032949	M5005_Spy_	-	771464	772228	254	4-nitrophenylphosphatase
Spy0768	3169.1375	1460.4237	1.1177	2.1700075	0.0014084	0.014936	M5005_Spy_	-	772228	772860	210	hypothetical protein M5005_Spy_0768
Spy0817	3717.7703	1907.4873	0.96276	1.949035	0.0066053	0.044956	M5005_Spy_	dacA1	812371	813699	442	D-alanyl-D-alanine carboxypeptidase
Spy0854	5703.5444	989.46724	2.5271	5.7641185	5.15E-12	1.88E-09	M5005_Spy_	-	844403	845092	229	Na+ driven multidrug efflux pum
Spy0856	11965.461	6371.2018	0.90924	1.8780559	0.0067881	0.045353	M5005_Spy_	-	846233	847759	508	glycine betaine transporter permeas
Spy0863	3114.5492	1048.9185	1.5701	2.9692529	2.12E-05	0.0007384	M5005_Spy_	prfA	853171	854250	359	peptide chain release factor
Spy0864	2977.1506	1091.0322	1.4482	2.7286739	8.63E-05	0.0021868	M5005_Spy_	hemK	854250	855089	279	peptide release factor-glutamine N5-methyltrans
Spy0865	2717.1323	1046.2504	1.3769	2.5970972	0.000179	0.0036094	M5005_Spy_	-	855073	855663	196	SUA5 protein
Spy0866	2299.1586	881.51512	1.383	2.6081015	0.0001846	0.0036201	M5005_Spy_	-	855681	856133	150	phosphinothricin N-acetyltransferas
Spy0867	11417.228	4680.3247	1.2865	2.4393555	0.0003237	0.0051788	M5005_Spy_	glyA	856123	857379	418	serine hydroxymethyltransferase
Spy0868	9421.7704	3349.9931	1.4918	2.8123965	3.61E-05	0.001046	M5005_Spy_	-	857386	858363	325	hypothetical protein M5005_Spy_0868
Spy0869	4824.8024	1829.9125	1.3987	2.6366389	0.0001171	0.0027344	M5005_Spy_	-	858364	858963	199	hypothetical protein M5005_Spy_0869
Spy0870	14032.148	4883.1772	1.5228	2.873482	1.65E-05	0.0006397	M5005_Spy_	-	858973	860697	574	multidrug resistance ABC transporter ATP-bindin
Spy0871	13251.765	4816.9343	1.46	2.7510836	3.34E-05	0.0009988	M5005_Spy_	-	860694	862421	575	multidrug resistance ABC transporter ATP-bindin
Spy0924	2874.6824	803.98005	1.8382	3.5756363	2.61E-07	2.64E-05	M5005_Spy_	-	912228	913496	422	GntR family transcriptional regulato
Spy0928	6586.1002	3390.1627	0.95807	1.9427092	0.0075542	0.048517	M5005_Spy_	lplA	918032	919051	339	lipoate-protein ligase A

Spy0929	4660.64	2221.6726	1.0689	2.0978332	0.0025377	0.022579	M5005_Spy_	-	919098	919979	293	SIR2 family proteir
Spy0930	4908.6883	2503.965	0.97112	1.9603619	0.0072266	0.047758	M5005_Spy_	-	919972	920784	270	hypothetical protein M5005_Spy_093(
Spy0931	1479.7081	595.35448	1.3135	2.4854378	0.0003957	0.0059642	M5005_Spy_	-	920777	921109	110	glycine cleavage system protein H
Spy0932	7088.2235	2948.0622	1.2657	2.4044385	0.000297	0.0049695	M5005_Spy_	-	921151	922149	332	luciferase-like monooxygenase
Spy0933	8411.4744	3460.5247	1.2814	2.4307474	0.0002441	0.0044516	M5005_Spy_	-	922146	923345	399	NADH-dependent flavin oxidoreductas
Spy0934	7526.7877	2979.759	1.3368	2.5259043	0.0001249	0.0028466	M5005_Spy_	-	923342	924178	278	lipoate-protein ligase A
Spy0947	6554.5415	2780.0795	1.2374	2.3577324	0.0002256	0.0041923	M5005_Spy_	ciaH	936420	937730	436	sensor proteir
Spy0948	3167.8693	1124.3664	1.4944	2.8174695	1.76E-05	0.0006564	M5005_Spy_	ciaR	937723	938397	224	transcriptional regulatory prote
Spy0952	6558.7042	3263.8639	1.0068	2.0094491	0.0033836	0.02743	M5005_Spy_	pstB2	942977	943780	267	phosphate transporter ATP-binding prote
Spy0953	6351.774	3331.5756	0.93095	1.906531	0.0066604	0.045162	M5005_Spy_	pstA	943796	944683	295	phosphate transporter permeas
Spy0955	4707.1065	2249.9203	1.065	2.0921699	0.0027518	0.023901	M5005_Spy_	pstS	945618	946484	288	phosphate-binding protei
Spy0960	3607.4749	1464.9787	1.3001	2.4624595	0.0003225	0.0051788	M5005_Spy_	mreA	949441	950373	310	bifunctional riboflavin kinase/FMN adenylyltrans
Spy0961	3407.8474	1417.4958	1.2655	2.4041052	0.0004244	0.006243	M5005_Spy_	truE	950402	951286	294	tRNA pseudouridine synthase
Spy0989	41826.004	19950.399	1.068	2.096525	0.0014674	0.015207	M5005_Spy_	pfkA	976340	977353	337	6-phosphofructokinase
Spy0990	9863.519	4446.3232	1.1495	2.21837	0.0014328	0.01502	M5005_Spy_	dnaE	977433	980543	1036	DNA polymerase III DnaE
Spy1007	278.63532	97.349594	1.5171	2.8621514	0.0019731	0.018842	M5005_Spy_	-	991293	994733	1146	phage proteir
Spy1008	124.78564	36.471825	1.7746	3.4214313	0.006159	0.042343	M5005_Spy_	-	994734	996218	494	hypothetical protein M5005_Spy_100
Spy1076	24091.393	7281.1015	1.7263	3.3087814	3.94E-07	3.59E-05	M5005_Spy_	glnH	1E+06	1051929	724	transporter
Spy1077	11551.218	5048.2497	1.1942	2.2881791	0.0003696	0.0056652	M5005_Spy_	glnQ.2	1E+06	1052669	246	glutamine ABC transporter ATP-binding prote
Spy1119	24428.224	9566.0483	1.3526	2.5537194	7.40E-05	0.0019289	M5005_Spy_	gapN	1E+06	1095442	475	NADP-dependent glyceraldehyde-3-phosphate d
Spy1133	6345.76	3266.1953	0.95818	1.9428574	0.0042609	0.032383	M5005_Spy_	prsA	1E+06	1110492	351	foldase PrsA
Spy1134	1084.4919	505.34026	1.1017	2.1460743	0.0035223	0.028178	M5005_Spy_	-	1E+06	1111262	235	O-methyltransferase
Spy1145	44008.865	16836.823	1.3862	2.6138928	2.91E-05	0.0009219	M5005_Spy_	sodA	1E+06	1121904	201	superoxide dismutase
Spy1235	17798.27	6886.6194	1.3699	2.5845265	0.0001047	0.0025131	M5005_Spy_	-	1E+06	1191113	567	phosphoglucomutase
Spy1243	33067.549	17167.831	0.94571	1.9261366	0.0049441	0.035786	M5005_Spy_	ileS	1E+06	1201199	933	isoleucyl-tRNA synthetase
Spy1245	10952.302	5791.3046	0.91927	1.8911581	0.0080622	0.049748	M5005_Spy_	-	1E+06	1203031	263	RNA binding protei
Spy1246	847.94385	386.39443	1.1339	2.1945118	0.0035018	0.028138	M5005_Spy_	-	1E+06	1203285	84	hypothetical protein M5005_Spy_124(
Spy1247	12101.006	6190.6204	0.96697	1.9547309	0.0061751	0.042343	M5005_Spy_	-	1E+06	1203958	222	hypothetical protein M5005_Spy_124
Spy1249	18289.168	8991.8497	1.0243	2.0339723	0.0036775	0.02901	M5005_Spy_	ftsZ	1E+06	1205951	439	cell division protein Fts
Spy1250	12671.185	4615.8233	1.4569	2.7451786	3.21E-05	0.0009916	M5005_Spy_	ftsA	1E+06	1207339	454	cell division protei
Spy1255	37614.286	17009.332	1.145	2.2114613	0.0007957	0.0096117	M5005_Spy_	typA	1E+06	1213724	613	GTP-binding protei
Spy1262	3497.0183	1595.8556	1.1318	2.1913197	0.0017514	0.017268	M5005_Spy_	-	1E+06	1218793	206	transcriptional regulato
Spy1266	7880.9686	3626.718	1.1197	2.1730178	0.0011331	0.012679	M5005_Spy_	-	1E+06	1221668	345	ATP-dependent protease La
Spy1328	1644.3854	617.3589	1.4134	2.6636416	0.0001337	0.0029729	M5005_Spy_	-	1E+06	1295308	210	Xaa-Pro dipeptidase
Spy1344	1854.6982	831.31453	1.1577	2.2310147	0.0013309	0.01445	M5005_Spy_	atoB	1E+06	1313190	395	acetyl-CoA acetyltransferase
Spy1345	713.08091	284.75373	1.3244	2.5042872	0.0009047	0.010646	M5005_Spy_	atoD.1	1E+06	1313864	216	acetate CoA-transferase subunit alph
Spy1346	966.90873	435.51377	1.1507	2.2202159	0.0028103	0.024293	M5005_Spy_	atoA	1E+06	1314525	219	acetate CoA-transferase subunit bet
Spy1347	1433.1074	697.28481	1.0393	2.0552302	0.0045368	0.033915	M5005_Spy_	-	1E+06	1315337	259	3-hydroxybutyrate dehydrogenase
Spy1358	5625.2345	2770.4775	1.0218	2.0304507	0.0030768	0.026103	M5005_Spy_	nadE	1E+06	1328586	484	nicotinate phosphoribosyltransferas
Spy1489	9354.6125	4784.1659	0.96741	1.9553271	0.0035851	0.028556	M5005_Spy_	fabF	1E+06	1446022	410	3-oxoacyl-ACP synthase
Spy1490	3411.5845	1622.4152	1.0723	2.102783	0.0015868	0.015816	M5005_Spy_	fabG	1E+06	1446771	244	3-ketoacyl-ACP reductase
Spy1491	3415.6005	1816.8629	0.91069	1.8799444	0.0076536	0.048627	M5005_Spy_	fabD	1E+06	1447699	312	ACP S-malonyltransferase
Spy1492	3904.1484	1727.6432	1.1762	2.2598077	0.0006334	0.0081307	M5005_Spy_	fabK	1E+06	1448695	323	enoyl-ACP reductase
Spy1591	5278.0502	1727.3342	1.6115	3.0556938	6.82E-06	0.0003276	M5005_Spy_	gcp	2E+06	1550266	342	DNA-binding/iron metalloprotein/AP endonuclea

Spy1592	889.43122	378.2086	1.2337	2.3516934	0.0018136	0.017785	M5005_Spy_	-	2E+06	1550711	151	ribosomal-protein-alanine acetyltransferas
Spy1593	3134.4933	1422.8366	1.1395	2.2030466	0.0021233	0.01976	M5005_Spy_	-	2E+06	1551381	232	glycoprotease
Spy1596	19166.863	8709.3969	1.138	2.2007572	0.0006103	0.008019	M5005_Spy_	glnA	2E+06	1555153	448	glutamine synthetase
Spy1597	3750.9445	1342.1305	1.4827	2.7947127	1.64E-05	0.0006397	M5005_Spy_	-	2E+06	1555562	123	MerR family transcriptional regulatc
Spy1678	4569.1757	1898.3046	1.2672	2.4069397	0.0001801	0.0036094	M5005_Spy_	-	2E+06	1636116	115	thioredoxir
Spy1710	2837.6797	836.11464	1.7629	3.3937964	1.49E-06	9.84E-05	M5005_Spy_	-	2E+06	1671598	825	histidine triad protei
Spy1711	785.75501	232.65208	1.7559	3.3773694	1.10E-05	0.0004744	M5005_Spy_	lmb	2E+06	1672531	306	laminin binding protei
Spy1732	123314.06	35170.294	1.8099	3.5061798	1.05E-07	1.47E-05	M5005_Spy_	prsA	2E+06	1696162	309	foldase PrsA
Spy1733	20783.961	6288.2676	1.7247	3.3051139	6.29E-07	4.99E-05	M5005_Spy_	-	2E+06	1696535	105	hypothetical protein M5005_Spy_173
Spy1734	56653.46	30102.72	0.91227	1.8820044	0.0055124	0.039123	M5005_Spy_	-	2E+06	1696947	101	hypothetical protein M5005_Spy_173
Spy1735	310490.71	139038.21	1.1591	2.2331807	0.0004729	0.0068463	M5005_Spy_	speB	2E+06	1698145	398	exotoxin E
Spy1849	1376.6787	545.21053	1.3363	2.5250291	0.0002767	0.0047609	M5005_Spy_	-	2E+06	1817031	429	zinc protease
Spy1850	1202.2767	542.81598	1.1472	2.2148362	0.0014812	0.01524	M5005_Spy_	-	2E+06	1818277	414	zinc protease
Spy1854	1060.8878	398.27756	1.4134	2.6636416	0.0001406	0.0030891	M5005_Spy_	-	2E+06	1823028	137	hypothetical protein M5005_Spy_185
Spy1855	4040.766	1770.605	1.1904	2.2821601	0.0004823	0.0068809	M5005_Spy_	recF	2E+06	1824136	368	recombination protein
Spy1866	11753.052	6147.9001	0.93487	1.9117184	0.0056067	0.039333	M5005_Spy_	parB	2E+06	1838523	268	chromosome partitioning protei

ase/unknown domain fusion prot		

p		
alph		
bet		
subunit E		
sferas		
g protein/permea		
g protein/permea		

feras		
ehydrogenas		
[illegible]		

