# Supplementary material for: Novel Tyrosine Kinase-Mediated Phosphorylation With Dual Specificity Plays a Key Role in the Modulation of Streptococcus pyogenes Physiology and Virulence
Source: Front Microbiol. 2021 Dec 7;12:689246. doi: 10.3389/fmicb.2021.689246 (PMC8689070; doi:10.3389/fmicb.2021.689246)
Supplement: Supplementary file 10 [file Data_Sheet_10.PDF]

Table-S4-Down-regulated DEGS in SP-TyK mutant

Gene_id	**Locus**	readcount_\	log2FoldCha	pval	padj	**Start**	**Stop**	**Length**	**Protein name**
Spy0014	-	5024.2009	-1.2302	0.0006374	0.0081307	15079	16470	463	amino acid permease
Spy0113	-	29.380898	-3.9532	0.004743	0.035025	124370	124720	116	transposase
Spy0115	-	282.5548	-4.1128	3.34E-05	0.0009988	125973	126638	221	hypothetical protein M5005_Spy_0115
Spy0124	sloR	2892.8369	-1.6846	5.24E-06	0.000273	133375	134394	339	transcriptional regulato
Spy0125	-	332.27231	-2.152	0.0004904	0.0068809	134849	135169	106	hypothetical protein M5005_Spy_0125
Spy0126	ntpI	2273.7405	-2.5024	0.0013634	0.014628	135159	137180	673	V-type ATP synthase subunit
Spy0127	ntpK	464.12295	-2.4527	0.0006476	0.0082029	137182	137661	159	V-type ATP synthase subunit K
Spy0128	ntpE	767.2457	-2.7724	0.0005339	0.0072679	137729	138313	194	V-type sodium ATP synthase subunit I
Spy0129	ntpC	1627.0262	-2.9325	0.0045902	0.034141	138329	139327	332	V-type ATP synthase subunit C
Spy0130	ntpF	509.24212	-2.6003	0.0006351	0.0081307	139324	139644	106	V-type ATP synthase subunit F
Spy0131	ntpA	3655.0792	-2.4719	0.0025801	0.022735	139845	141620	591	V-type ATP synthase subunit A
Spy0132	ntpB	2636.1632	-2.4977	0.0041006	0.031426	141621	143036	471	V-type ATP synthase subunit B
Spy0133	ntpD	1365.9422	-2.7435	0.0030566	0.026053	143081	143707	208	V-type ATP synthase subunit D
Spy0138	nusG	4117.7258	-1.1423	0.0009215	0.010774	149253	149792	179	transcription antitermination protein Nu
Spy0141	slo	1380.7077	-1.4722	0.0072909	0.04787	152032	153747	571	streptolysin C
Spy0142	-	107.72917	-2.1587	0.0012305	0.013521	154002	154433	143	hypothetical protein M5005_Spy_0142
Spy0143	-	71.51179	-3.4202	0.000408	0.0060508	154619	154855	78	hypothetical protein M5005_Spy_0143
Spy0144	-	34.38028	-4.9487	0.0006984	0.0084922	155265	155432	55	hypothetical protein M5005_Spy_0144
Spy0150	-	81.078033	-2.4934	0.0014578	0.015194	162449	162934	161	PTS system 3-keto-L-gulonate specific transporter subunit II
Spy0151	ulaD	111.6872	-1.7989	0.0057393	0.040109	163025	163687	220	3-keto-L-gulonate-6-phosphate decarboxylas
Spy0152	-	143.00043	-2.1999	0.0003359	0.0052358	163692	164555	287	L-xylulose 5-phosphate 3-epimeras
Spy0153	araD	218.89925	-2.3136	2.12E-05	0.0007384	164557	165261	234	L-ribulose-5-phosphate 4-epimeras
Spy0157	opuAA	29370.102	-1.4616	0.0011953	0.013213	169064	170260	398	glycine betaine transport ATP-binding prote
Spy0158	opuABC	72719.867	-1.446	0.001164	0.012946	170276	172003	575	glycine betaine-binding protein/glycine betaine transporter permea
Spy0164	-	8948.9175	-0.9645	0.004881	0.03547	177942	178472	176	parB-like nuclease
Spy0177	-	1887.4125	-2.8723	0.0062146	0.042455	186919	187458	179	bioY protein
Spy0211	rpmH	2559.3555	-1.5062	2.93E-05	0.0009219	217191	217325	44	50S ribosomal protein L34
Spy0213	-	2236.8051	-1.5166	0.0001281	0.0028854	218352	219671	439	N-acetylneuraminate-binding protei
Spy0214	-	739.58529	-1.9678	1.12E-05	0.0004744	219774	220661	295	N-acetylneuraminate transporter permeas
Spy0215	-	784.36032	-2.098	4.64E-07	3.98E-05	220674	221504	276	N-acetylneuraminate transporter permeas
Spy0216	-	580.99712	-2.2867	2.28E-07	2.60E-05	221661	222323	220	hypothetical protein M5005_Spy_0216
Spy0217	nanH	867.82029	-2.1783	2.27E-06	0.0001382	222335	223249	304	N-acetylneuraminate lyase
Spy0218	-	1015.7689	-2.2241	2.49E-07	2.64E-05	223271	224209	312	N-acetylmannosamine kinase
Spy0321	fhuG	892.80243	-1.3179	0.0008622	0.010212	327182	328108	308	ferrichrome transporter permeas
Spy0322	fhuB	690.1944	-1.1354	0.0048709	0.03547	328180	329232	350	ferrichrome transporter permeas
Spy0323	fhuD	579.12544	-1.0591	0.0077346	0.048648	329222	330154	310	ferrichrome-binding protei
Spy0341	-	35548.434	-1.7688	1.20E-05	0.0004991	344713	349656	1647	lactocepir
Spy0344	-	1654.8397	-2.036	0.0055982	0.039333	350427	351134	235	permease
Spy0345	metG	19406.573	-0.91539	0.0067627	0.04535	351377	353374	665	methionyl-tRNA synthetase
Spy0351	spyA	1308.5784	-1.059	0.0031112	0.026272	357724	358476	250	C3 family ADP-ribosyltransferase
Spy0413	secG	3853.4283	-1.2941	0.0002275	0.0041923	405469	405705	78	preprotein translocase subunit Sec
Spy0416	-	4740.9766	-1.1709	0.000571	0.0076016	408617	409327	236	glutaminyl-peptide cyclotransferase

Spy0499	-	2889.8547	-0.92701	0.006926	0.046095	484179	484742	187	thiamine transporte
Spy0519	agaD	516.56474	-1.121	0.0076898	0.048627	507954	508775	273	PTS system N-acetylgalactosamine-specific transporter subunit II
Spy0549	-	908.54934	-1.2214	0.0015458	0.015577	539130	539414	94	hypothetical protein M5005_Spy_054
Spy0551	rplS	56061.751	-1.5467	0.0001719	0.0035639	540784	541131	115	50S ribosomal protein L1
Spy0562	sagA	20377.692	-1.1917	0.0004985	0.0069414	559706	559867	53	streptolysin S
Spy0597	rpsU	4696.9954	-1.364	0.0001011	0.0024782	600432	600608	58	30S ribosomal protein S2
Spy0619	infC	8655.5829	-1.1734	0.0004847	0.0068809	622563	623093	176	translation initiation factor IF-
Spy0620	rpmI	2908.9677	-0.95871	0.007935	0.04938	623135	623332	65	50S ribosomal protein L3
Spy0621	rplT	18753.706	-1.0862	0.0018685	0.018226	623391	623750	119	50S ribosomal protein L2
Spy0649	-	1849.1784	-1.0269	0.0048565	0.03547	654515	654754	79	RNA binding protei
Spy0668	mac	71.104403	-2.607	0.0015266	0.015543	671769	672788	339	IgG-degrading protease
Spy0694	clpL	46057.366	-1.614	2.91E-06	0.0001711	695785	697884	699	ATP-dependent protease ATP-binding subun
Spy0714	-	4469.5758	-2.6873	9.29E-06	0.0004134	719080	719310	76	hypothetical protein M5005_Spy_071
Spy0858	xpt	674.44081	-1.7468	2.15E-05	0.0007384	849325	849906	193	xanthine phosphoribosyltransferas
Spy0859	-	3276.9407	-1.7777	7.53E-07	5.73E-05	849906	851189	427	xanthine permease
Spy0900	-	1529.8753	-2.9448	1.17E-13	5.36E-11	888082	889488	468	Mg2+/citrate complex secondary transporte
Spy0910	citC	13978.227	-1.5988	3.71E-06	0.0002052	896802	897854	350	(citrate (pro-3S)-lyase) ligase
Spy0911	-	5247.2235	-1.9759	2.73E-08	4.14E-06	897943	898425	160	hypothetical protein M5005_Spy_091
Spy0913	xerS	11918.605	-2.2998	9.10E-11	2.37E-08	899125	900039	304	site-specific tyrosine recombinase Xer
Spy0914	-	2476.0659	-2.8822	9.00E-14	5.36E-11	900733	901362	209	phage transcriptional represso
Spy0920	-	764.83377	-2.4909	3.98E-05	0.0010994	907471	908694	407	UDP-N-acetylmuramoylpentapeptide-lysine N(6)-alanyltransferas
Spy0921	-	1978.5288	-1.9497	1.74E-07	2.27E-05	909059	910603	514	ABC transporter ATP-binding prote
Spy0922	pdxK	246.9353	-1.7614	0.0081005	0.049748	910737	911294	185	hypothetical protein M5005_Spy_092
Spy0923	-	744.24297	-1.6976	2.44E-05	0.0008077	911272	912138	288	pyridoxamine kinase
Spy0925	rnhB	11723.72	-1.0349	0.0031955	0.026859	913894	914250	118	anaerobic ribonucleoside-triphosphate reducta
Spy0963	-	2608.5485	-1.5536	9.20E-05	0.0022994	952839	953795	318	hypothetical protein M5005_Spy_096
Spy0964	-	1246.7364	-1.4229	0.003682	0.02901	953806	954402	198	type I restriction-modification system specificity subur
Spy0986	glmS	34481.067	-1.7895	3.68E-07	3.53E-05	972010	973824	604	glucosamine--fructose-6-phosphate aminotransferas
Spy1044	-	109.10748	-1.7504	0.0061104	0.042249	1015738	1016199	153	phage proteir
Spy1083	-	814.4092	-1.1493	0.0029671	0.025409	1055641	1057635	664	PTS system, mannitol (cryptic)-specific IIA componer
Spy1084	-	340.12634	-1.1619	0.0079592	0.04938	1057741	1058835	364	outer surface protei
Spy1086	-	1367.6417	-1.3677	0.0003068	0.0050413	1060469	1061164	231	nicotinamide mononucleotide transporte
Spy1092	rsuA	2067.1383	-1.1567	0.0013606	0.014628	1064529	1065263	244	ribosomal small subunit pseudouridine synthase
Spy1129	-	1027.2851	-1.4697	0.0001825	0.0036188	1104384	1105079	231	CAAX amino protease
Spy1130	-	1061.0574	-1.3538	0.0005429	0.0073351	1105098	1105859	253	hypothetical protein M5005_Spy_113
Spy1135	-	2193.5753	-2.37	0.0006111	0.008019	1111328	1112524	398	oxalate/formate antiporte
Spy1152	-	5245.2989	-1.1125	0.0010123	0.011613	1128124	1129107	327	kup system potassium uptake protein, partia
Spy1162	-	3988.4758	-1.0678	0.0067517	0.04535	1139771	1140313	180	hypothetical protein M5005_Spy_116
Spy1170	-	1179.0616	-1.2035	0.0019254	0.01868	1147096	1147590	164	hypothetical protein M5005_Spy_117
Spy1197	-	121.9111	-2.2393	0.0006881	0.0084754	1165658	1166077	139	phage proteir
Spy1214	-	14153.648	-3.3625	6.18E-06	0.0003046	1173018	1173257	79	phage proteir
Spy1215	-	4109.8763	-2.6469	1.66E-06	0.0001042	1173399	1174205	268	phage proteir
Spy1219	-	418.53979	-1.556	0.0005043	0.0069678	1176511	1176858	115	Cro/CI family phage transcriptional regulato
Spy1220	-	254.893	-1.3469	0.0050807	0.036629	1176862	1177242	126	phage proteir

Spy1222	int.2	1333.0784	-2.1796	2.21E-08	3.67E-06	1177644	1178786	380	integrase
Spy1237	artP	1158.3119	-2.1907	0.0004328	0.0063153	1192840	1193574	244	arginine transport ATP-binding prote
Spy1238	artQ	755.3423	-2.0959	8.09E-05	0.0020796	1193574	1194260	228	arginine transporter permeas
Spy1395	lacD.1	8739.3164	-4.0745	0.0034141	0.027554	1368872	1369849	325	tagatose 1,6-diphosphate aldolase
Spy1396	-	2315.5232	-4.1343	0.0024318	0.021743	1369999	1370349	116	tagatose-6-phosphate kinase
Spy1397	lacB.1	2615.9167	-4.0835	0.0025733	0.022735	1370359	1370874	171	galactose-6-phosphate isomerase subunit Lac
Spy1398	lacA.1	3699.0871	-3.8108	0.0019429	0.01875	1370889	1371314	141	galactose-6-phosphate isomerase subunit Lac
Spy1399	-	21331.425	-3.5653	0.0023331	0.021278	1371554	1373002	482	PTS system galactose-specific transporter subunit II
Spy1400	-	766.11374	-3.4328	0.0033064	0.027264	1373031	1373336	101	PTS system galactose-specific transporter subunit II
Spy1401	-	2114.0158	-3.2836	0.0075167	0.048447	1373329	1373802	157	PTS system galactose-specific transporter subunit II
Spy1407	-	457.26659	-1.1674	0.0051854	0.037237	1378067	1379053	328	esterase
Spy1421	-	484.19673	-1.7553	4.39E-05	0.0011954	1389641	1391548	635	phage infection protei
Spy1424	-	302.66002	-1.602	0.0006254	0.0081307	1393246	1395228	660	phage endopeptidase
Spy1425	-	146.8544	-1.8328	0.0019607	0.018823	1395238	1396080	280	phage protein
Spy1426	-	1092.1365	-1.6354	1.76E-05	0.0006564	1396092	1400474	1460	phage protein
Spy1439	-	129.43227	-1.679	0.0053366	0.038023	1406796	1408328	510	portal protein
Spy1451	-	273.01957	-1.7531	0.0010977	0.01236	1418373	1418930	185	phage protein
Spy1461	-	84.166402	-2.2659	0.0069496	0.046095	1422316	1422531	71	phage protein
Spy1464	-	323.04599	-1.4433	0.0033482	0.027264	1423403	1423804	133	Cro/CI family phage transcriptional regulato
Spy1467	int.3	1394.0283	-2.3608	0.0001598	0.0034291	1424880	1425959	359	integrase
Spy1476	-	536.05337	-7.8145	4.80E-07	3.98E-05	1432146	1432607	153	ATP/GTP hydrolase
Spy1477	-	5682.7428	-2.0672	2.26E-05	0.0007625	1432761	1434221	486	guanine-hypoxanthine permease
Spy1497	dnaJ	41448.376	-1.2267	0.0002834	0.0047858	1451679	1452836	385	molecular chaperone Dna
Spy1539	scrK	829.53251	-1.5924	0.0039252	0.030337	1497312	1498268	318	fructokinase
Spy1540	endoS	6698.064	-1.7666	0.0040813	0.031411	1498460	1501447	995	endo-beta-N-acetylglucosaminidase F2
Spy1542	scrA	6099.1375	-2.0758	0.0002473	0.0044663	1501678	1503561	627	PTS system sucrose-specific transporter subunit IIAB
Spy1543	scrB	1355.9253	-1.1665	0.0015757	0.015791	1503803	1505242	479	sucrose-6-phosphate hydrolase
Spy1544	scrR	1970.7713	-1.2878	0.0004868	0.0068809	1505247	1506212	321	sucrose operon represso
Spy1556	-	992.90434	-1.562	6.03E-05	0.0015935	1515847	1516140	97	hypothetical protein M5005_Spy_155
Spy1572	-	3934.5375	-1.4835	3.77E-05	0.0010736	1533053	1533949	298	hypothetical protein M5005_Spy_157
Spy1606	rpmB	12723.414	-1.6634	0.007483	0.048432	1563550	1563738	62	50S ribosomal protein L2
Spy1608	-	7879.2269	-0.93932	0.0059216	0.041225	1565347	1566273	308	alpha/beta hydrolase
Spy1610	pyrG	11783.686	-1.259	0.0032816	0.027264	1567892	1568044	50	CTP synthase, partia
Spy1628	-	186.97361	-2.4234	3.86E-05	0.0010825	1592455	1593192	245	ABC transporter ATP-binding prote
Spy1629	salX	103.21728	-2.4814	0.0006774	0.0084143	1593189	1593650	153	lantibiotic transport ATP-binding prote
Spy1630	salB	454.94813	-2.8882	1.90E-09	3.85E-07	1593724	1595349	541	serine (threonine) dehydratase
Spy1636	lacC.2	3151.007	-1.7235	0.0015339	0.015543	1600609	1601538	309	tagatose-6-phosphate kinase
Spy1661	-	224.51213	-1.3295	0.0076371	0.048627	1617703	1618431	242	translaldolase
Spy1691	-	659.42995	-1.9557	3.09E-06	0.0001762	1650989	1651807	272	exodeoxyribonuclease II
Spy1692	-	4121.9518	-1.187	0.0008211	0.0097888	1651890	1653785	631	PTS system glucose-specific transporter subunit IIAB
Spy1701	flaR	1442.2175	-1.7847	1.51E-06	9.84E-05	1661525	1662031	168	topology modulation protei
Spy1718	sic1.01	7205.9369	-1.5132	3.60E-05	0.001046	1680969	1681910	313	inhibitor of complement prote
Spy1731	-	920.62534	-3.548	5.93E-06	0.0003004	1694721	1694957	78	hypothetical protein M5005_Spy_173
Spy1744	-	1023.8742	-1.3317	0.0005664	0.007597	1706391	1707695	434	PTS system cellobiose-specific transporter subunit I

Spy1746	-	183.09677	-1.7089	0.002013	0.019124	1708041	1708361	106	PTS system cellobiose-specific transporter subunit II
Spy1747	-	5885.1738	-1.2699	0.0002739	0.0047578	1708649	1709629	326	sorbitol operon regulatc
Spy1748	-	3541.8343	-0.98135	0.0045296	0.033915	1709645	1710394	249	DeoR family transcriptional regulatc
Spy1749	-	4866.6586	-1.8542	2.23E-07	2.60E-05	1710517	1711290	257	pyruvate formate-lyase activating enzyme
Spy1750	-	418.34774	-2.8345	2.71E-05	0.0008813	1711325	1711525	66	hypothetical protein M5005_Spy_175[illegible]
Spy1783	dexS	5933.0942	-3.4704	1.57E-11	4.78E-09	1750283	1751911	542	trehalose-6-phosphate hydrolas[illegible]
Spy1784	-	12187.54	-3.3758	2.49E-17	2.27E-14	1751978	1754002	674	PTS system, trehalose-specific IIBC componen
Spy1794	-	10078.031	-1.5381	1.32E-05	0.0005368	1762253	1763812	519	hypothetical protein M5005_Spy_179[illegible]
Spy1826	-	5183.9935	-1.2766	0.000179	0.0036094	1792426	1793013	195	hypothetical protein M5005_Spy_182[illegible]
Spy1830	-	443.87946	-1.613	0.0003415	0.0052787	1796614	1797147	177	TetR family transcriptional regulatc
Spy1842	sdhA	4301.5164	-1.0461	0.0022733	0.020837	1809388	1810260	290	L-serine dehydratase
