# Supplementary material for: Novel Tyrosine Kinase-Mediated Phosphorylation With Dual Specificity Plays a Key Role in the Modulation of Streptococcus pyogenes Physiology and Virulence
Source: Front Microbiol. 2021 Dec 7;12:689246. doi: 10.3389/fmicb.2021.689246 (PMC8689070; doi:10.3389/fmicb.2021.689246)
Supplement: Supplementary file 11 [file Data_Sheet_11.PDF]

**Table-S5.** Phosphoproteomic analysis of the SP-TyK phosphorylation and its phosphorylated substrates

## A. *In vitro* autophosphorylation of SP-TyK

| Peptide Sequence | Theoretical m/z | Observed m/z | Mass Error | Scan in Control | Scan # in TK | Modification |
|---|---|---|---|---|---|---|
| 56SPTY(PO4)TIVR63 | 1055.4737 | 1015.4740 | -0.22 | No MS | #1165 | Phosphorylation on Tyr 59 |
| 69 LPLYHLDIY(PO4)R78 | 1935.9832 | 1935.9843 | -o.052 | Second rerun, low abundance #4400 | #4402 | Phosphorylation on Tyr77 |

## B. *In vitro* phosphorylation of WalR by SP-TyK

| Peptide Sequence | Theoretical m/z | Observed m/z | Mass Error | Retention time | Scan # in Control | Scan # in TK | Modification |
|---|---|---|---|---|---|---|---|
| 19FNLTKEGY (PO4)DIVTAFDGR35 | 676.3101 | 676.3112 | 1.63 | 55.63 | No MSMS | #26888 | Phosphorylation on Y26 |
| 24EGY(PO4)DIVTAFDGR35 | 711.8005 | 711.7999 | -0.84 | 45.32 | No MSMS | #19684 | Phosphorylation on Y26 |
| 82DSEFDKVIGLEIGADDY(PO4)VTKPFSNR106 | 965.7865 | 965.7861 | -0.41 | 54.59 | No MSMS | #28176 | Phosphorylation on Y98 |
| 118RTETIETAVAEENASSGTQELT(PO4)IGNLQILPDAFVAK135 | 1299.644 | 1299.6442 | 0.38 | 56.86 | No MSMS | #28108; 28145 | Phosphorylation on T139, low abundance T135 |
| 212IEDTPS(PO4)RPEYILTR225 | 885.4271 | 885.4274 | 0.34 | 32.81 | No MSMS | #13367; or #14981 | Phosphorylation on S217 |
| 212IEDTPSRPEY(PO4)ILTR225 | 885.4271 | 885.4274 | 0.34 | 31.31 | No MSMS | #12591; or #14091 | Phosphorylation on Y221 |

## C. *In vitro* phosphorylation of CovR by SP-TyK

| Peptide Sequence | Theoretical m/z | Observed m/z | Mass Error | Rentention time | Scan # in Control | Scan # in TK | Modification |
|---|---|---|---|---|---|---|---|
| 127KVPS(PO4)QGIYR135 | 564.2842 | 564.2842 | 0.08 | 16.73 | No MSMS | #5395 | Phosphorylation on S130 |
| 145S(PO4)VNRGDDEISLTKR158 | 557.2684 | 557.2687 | 0.54 | 17.97 | No MSMS | #6028 | Phosphorylation on S145 |
| 145SVNRGDDEIS(PO4)LTKR158 | 557.2684 | 557.2687 | 0.54 | 17.9 | No MSMS | #5522 | Phosphorylation on S154 |

Continue..

D. *In vitro* phosphorylation of SP-STP by SP-TyK

| Peptide Sequence | Theoretical m/z | Observed m/z | Mass Error | Rentention time | Scan # in Control | Scan # in TK | Modification |
|---|---|---|---|---|---|---|---|
| 3IS(PO4)LKTDIGQKR13 | 446.9095 | 446.9097 | 0.45 | 19.1 | No MSMS | #8187 in STP_TK | Phosphorylation on S4 |
| 14S(PO4)NNQDFINKFDNK26 | 832.3592 | 832.359 | -0.24 | 29.36 | #12568 | #14170 | Phosphorylation on S14 |
| 43AGNIAS(PO4)EMTVTDLGR57 | 807.8633 | 807.864 | 0.87 | 45.6 | #22540 Rerun | #24067 in Rerun | Phosphorylation on S48 |
| 145AGQIT(PO4)EEEAASHPQR159 | 852.3729 | 852.373 | 0.12 | 16.05 | No Peak | #6496 in TK, #6506 in TK_rerun | Phosphorylation on S149 |
| 145AGQITEEEAAS(PO4)HPQR159 | 852.3729 | 852.373 | 0.12 | 13.09 | #3223 in Ctrl; #4606 in Ctrl_rerun | No Peak | Phosphorylation on S155 |
| 160NIITQSIGQAS(PO4)PVEPDLGVR179 | 1087.546 | 1087.547 | 0.55 | 51.97 | #25202; 25227 | #27181 | Phosphorylation on S170 |
| 180VLEPGDYLVINSDGLT(PO4)NMISNDEIVTILGSK220 | 1133.893 | 1133.894 | 0.53 | 62.05 | No MSMS | #38656 rerun | Phosphorylation on T195 |

Continue

## E. *In vitro* phosphorylation of SDH/GAPDH by SP-TyK

| Peptide Sequence | Theoretical m/z | Observed m/z | Mass Error | Rentention time | Scan # in Control | Scan # in TK | Modification |
|---|---|---|---|---|---|---|---|
| 32INDLT(PO4)DPNMLAHLLK46 | 596.6302 | 596.6307 | 0.84 | 53.91 | #26309 (not good) | didn't see | Phosphorylation on T37 |
| 130TVVFNTNHDILDGT(PO4)ETVISGASC(CAM)TTNC(CAM)LAPMAK162 | 1206.549 | 1206.55 | 0.91 | 54 | #26053; 27417 not very good | didn't see | Phosphorylation on S143 |
| 202AGAANIVPNST(PO4)GAAK216 | 711.3429 | 711.3428 | -0.14 | 22.9 | #8571 | #8411 and #9790 in rerun | Phosphorylation on T212 |
| 265AASNDSFGYTEDPIVSSDIVGVS(PO4)YGSLFDATQTK298 | 1207.877 | 1207.879 | 1.82 | 59.46 | #33358 | #34481 in Rerun | Phosphorylation on S287 |
| 299VMEVDGS(PO4)QLVK309 | 642.7989 | 642.7991 | 0.31 | 37.09 | No MSMS | #17410 in rerun | Phosphorylation on S305 |
| 310VVS(PO4)WYDNEMSYTAQLVR326 | 1070.974 | 1070.973 | -0.65 | 55.66 | #28023; 28046 in Rerun | #31659 in Rerun | Phosphorylation on S313 |
