# Supplementary material for: Novel Tyrosine Kinase-Mediated Phosphorylation With Dual Specificity Plays a Key Role in the Modulation of Streptococcus pyogenes Physiology and Virulence
Source: Front Microbiol. 2021 Dec 7;12:689246. doi: 10.3389/fmicb.2021.689246 (PMC8689070; doi:10.3389/fmicb.2021.689246)
Supplement: Supplementary file 12 [file Data_Sheet_12.PDF]

Table- S6: Mass-spectrometric analysis of *in vivo* phosphorylated WalR and CovR

A. *In vivo* phosphorylation of WalR:

MKKILIVDDEKPISDIIKFNLTKEGY^{26}DIVT^{30}AFDGREAVTIFEEEKPDLIILDLMLPELDGLEVAKEIRKT
SHVPIIMLSAKDSEFDKVIGLEIGADDYVTKPFSNRELLARVKAHLRRTETIETAVAEENASSGTQELTI
GNLQILPDAFVAKKHGQEVELTHREFELLHHLANHMGQVMTREHLLEIVWGYDYFGDVRTVDVTVRRLRE
KIEDTPS^{217}RPEY^{221}ILT^{224}RRGVGYY^{231}MKS^{234}Y^{235}D

	Query	Observed	Mr(expt)	Mr(calc)	Delta	Miss	Score	Expect	Rank		Peptide
1.	834	550.0800	1647.2182	1648.5435	-1.3253	2	13	0.047	1	U	RRGVGYYMKSYDA.- + Phospho (ST); 2 Phospho (Y)
2.	2470	834.7900	2501.3482	2501.0375	0.3107	3	17	0.031	1	U	RPEYILTRRGVGYYMKSYD - + Oxidation (M); Phospho (ST); Phospho (Y)
3.	4700	1451	2899.9874	2899.2418	0.7438	3	13	0.08	1		IEDTPSRPEYILTRRGVGYYMK + + Oxidation (M); Phospho (ST); Phospho (Y)
4.	592	502.09	1503.2482	1501.5528	1.6854	0	11	0.086			EGYDIVTAFDGR + Phospho (ST); Phospho (Y)

B. *In vivo* phosphorylation of CovR:

MTKKILIIEDEKNLARFVSLELQHEGYEVIVEVNGREGLETALEKEFDLILLDLMLPEMDGFEVT**RRLQT^{70}EKT^{73}T^{74}Y^{75}IMMMT^{80}ARDS^{84}IMDVVAGLDRGADDY^{99}**IVKPF AIEELLARIRAIFRRQDIESEKK**VPSQGIYRDLVLNPQNRSVNRGDDEISLTKREYDLLNILMTNMNRVMTR**EELLSNVWKYDEAVETNVVDVYIRYLRGKIDIPGKESYIQT VRGMGYVIREK

	Query	Observed	Mr(expt)	Mr(calc)	Delta	Miss	Score	Expect	Rank		sequence
1.	2568	853.2800	852.2727	853.4059	-1.1331	1	16	0.027	1	U	RRLQTEKT + Phospho (ST)
2.	1739	723.2300	2166.6682	2164.9121	1.7561	2	14	0.038	1	U	RRLQTEKTTYIMMMTARD + 2 Oxidation (M); 2 Phospho (ST)
3.	3829	1133.0800	2264.1454	2264.7049	-0.5594	1	17	0.028	1	U	RLQTEKTTYIMMMTARD + 3 Oxidation (M); 4 Phospho (ST); Phospho (Y)
4.	3291	974.9200	2921.7382	2921.0001	0.7381	1	19	0.012	1	U	KTTYIMMMTARDSIMDVVAGLDRG + 2 Oxidation (M); 4 Phospho (ST); Phospho (Y)
5.	3994	1256.6600	3766.9582	3766.4284	0.5298	2	14	0.035	1	U	KTTYIMMMTARDSIMDVVAGLDRGADDYIVKP + Oxidation (M); 4 Phospho (ST); Phospho (Y)
6.	4218	1460.7600	4379.2582	4377.9197	1.3385	4	13	0.072	1		RLQTEKTTYIMMMTARDSIMDVVAGLDRGADDYIVK + 2 Oxidation (M); 2 Phospho (ST); Phospho (Y)
7.	3852	1146.4300	3436.2682	3437.5141	-1.2459	3	11	0.078	1		RLQTEKTTYIMMMTARDSIMDVVAGLDR + Deamidated (NQ); 2 Oxidation (M); 2 Phospho (ST)
8.	4517	1314.23	3939.6682	3938.6920	0.9761	4	13	0.052	1		VPSQGIYRDLVLNPQNRSVNRGDDEISLTKR 4 Phospho (ST); 2 Phospho (Y)
9.	4596	1364.08	4089.2182	4086.7539	2.4602	4	13	0.07	1		SVNRGDDEISLTKREYDLLNILMTNMNRVMTR + 2 Deamidated (NQ); 3 Oxidation (M); 2 Phospho (ST); Phospho (Y)
