# Supplementary material for: Novel Tyrosine Kinase-Mediated Phosphorylation With Dual Specificity Plays a Key Role in the Modulation of Streptococcus pyogenes Physiology and Virulence
Source: Front Microbiol. 2021 Dec 7;12:689246. doi: 10.3389/fmicb.2021.689246 (PMC8689070; doi:10.3389/fmicb.2021.689246)
Supplement: Supplementary file 13 [file Data_Sheet_13.PDF]

Table- S7 Genomic environment of TsaE homologs

←	←	←upstream	TsaE homologs	Downstream→	→	→
S. pyogenes	HAD-hydrolase	Permease	SP-TyK/ spy1476	acetyltransferase	LTR/LCP	hypothetical
S. pneumoniae	transcriptional regulator	hypothetical	spd1743/UbK	acetyltransferase	LTR/LCP	competence inducible protei
S. agalactiae	DUF1361-protein	Permease	TsaE	acetyltransferase	LTR/LCP	hypothetical
S. salivarius	HAD-hydrolase	Permease	TsaE	acetyltransferase	LTR/LCP	ribosome assembly cofactor
S. aureus	dihydroxy acid hdeydratase	hypothetical	TsaE	tsaB	S18-alaninie acetyl	TsaD
Enterococcus faecalis	uracil glycosylase	pta	TsaE	acetyltransferase	hypothetical	LacI family regualtor BopD
Enterococcus faecium	uracil glycosylase	pta	TsaE	acetyltransferase	exonuclease	Exonuclease III
B. subtilis	tRNA	thiamine mono-P-kina	TsaE	tsaB	S18-alaninie acetyl	TsaD
B. anthracis	tRNA	tRNA	TsaE	tsaB	S18-alaninie acetyl	TsaD
Lactobacillus	uracil glycosylase	pta	TsaE	αβ hydrolase/ exonucle	hypothetical /murB	exonuclease
Listeria	hypothetical	hpothetical	TsaE	tsaB	TsaD	α-β hydrolase
Myocbactrium avium	alanine recemase	αβhydrolase	TsaE	tsaB	S18-alaninie acetyl	TsaD
Nocardia	alanine recemase	αβhydrolase	TsaE	tsaB	S18-alaninie acetyl	TsaD
Corynebacterium	dinelactone hydrolase	alanine recemase	TsaE	transporter	hypothetical	tsaB
Clostridium difficile	ECF transporter	DUF4116 protein	TsaE	TsaB	S18-alaninie acetyl	TsaD
rickettsia	RPE1 domain protein	transporter	TsaE	abc-transporter	hypothetical	hypothetical
Treponema pallidum	hypothetical	hypothetical	TsaE	TsaB	HD-GYP-protein	GldG family protein
Borrelia burgdofferri	ribosomal L20	hypotheticsl	TsaE	TsaB	carbon storage reg	FliW
helicobacter suis	uracil glycosylase	PTA	TsaE	Exonuclease	MurB	ABC transporter
Chlamidiae	hypothetical	DUF2709 protein	TsaE	DUF-3820 protein	acetyl coA thioeste	hypothetical
Mycoplasma pneumoniae	permease	lipoprotein	TsaD	Met-adenosyl transfera	hypothetical	signal recognition particle pr
Mycoplasma hypopneumoniae	rpo-Cβ subunit	rpoC	tsaE	DNA-Cyt-methytransfer	Ig-blocking virulenc	FUf31 -family protein
Mycoplasma capricolum	Ser/thr phosphatase	hypothetical	tsaE	tsaB	leucine -rich repeat	leucine -rich repeat surface p
Mycoplasma hominis	23s ribosomal RNA	metallopeptidase	tsaE	Hypothetical	tsaD	GTP-binding protein
E.coli	Epoxy-queuosine reductase	epimerase dehydratas	TsaE	muramyl-alanine amida	repir protein MutL	methyl transferase
Haemophilus influeanzae	polunucleotide adenyl transferas	phosphokinase	TsaE	muramyl-alanine amida	repir protein MutL	methyl transferase
Pseudomonas	Epoxy-queuosine reductase	NADPH-dehydratase	TsaE	AmiB	mutL	MiaA tRna transferase
Vibrio colarae	t-RNA	Epoxy-queuosine redu	TsaE	AmiB	mutL	MiaA tRna transferase
salmonella typhimurium	hypothetical	epimerase dehydratas	TsaE	amiB	MiaA tRna transfer	HFQ chapeon
shigella flexneri	hexaqueuosine reductase	NADPH-dehydratase	TsaE	AmiB	mutL	MiaA tRna transferase
Klebsiella pnemoniae	hexaqueuosine reductase	NADPH-dehydratase	TsaE	AmiB	mutL	MiaA tRna transferase
Enterobacter cloacae	hexaqueuosine reductase	NADPH-dehydratase	TsaE	AmiB	mutL	MiaA tRna transferase
Acinetobacter	relB/DinJ toxin-detoxin protein	RluA uridine syntase	TsaE	mutL	MiaA tRna transfer	HFQ chapeon
yersinia pestis	hexaqueuosine reductase	NADPH-dehydratase	TsaE	AmiB	mutL	MiaA tRna transferase
Burkholderia cenopecia	methyl transferase	QueG	TsaE	n acetyl muramyl amida	transporter	Pirin family protein
Neisseria gonorhoea/meningitid	FiC protein	gltamate recemase	TsaE	muramyl-alanine amida	Fc protein	methyl transferase

Bacteroides fragilis	Hypothetical	metal ABC transporter	TsaE	immunity 17 protein	DUF58	MoxR family ATPase
Bifidobacterium breve	ComE	DNApolymerase	TsaE	TsaB	S18-alaninie acetyl	TsaD
fusobacterium nucleatum	NUDIX domain protein	adenylyl transferase/r	TsaE	TsaB	duf4357-protein	hypothetical
porphromonas gingivalis	atp-binding protein	pglZ-protein	TsaE	immunity 17	thimidine kinase	lipoprotein
